# Supplementary material for: Osteoarthritis in older adults: A global health challenge and the role of high BMI in shaping disease trends
Source: PLoS One. 2026 Mar 30;21(3):e0344284. doi: 10.1371/journal.pone.0344284 (PMC13035164; doi:10.1371/journal.pone.0344284)
Supplement: S1 File — (DOCX) [file pone.0344284.s001.docx]

**Supplementary Materials**

**Osteoarthritis in older adults: a global health challenge and the role of high BMI in shaping disease trends**

Yan Gao^1^, Hailong Yu^2^, Wenfeng Han^2^, Ning Wang^2^, Bin Zheng^2^, Aoxiang Yang^2^, Yu Wang^2^*

^1^ Department of Disease Prevention and Control, General Hospital of Northern Theater Command, Shenyang, China

^2^ Department of Orthopedics, General Hospital of Northern Theater Command, Shenyang, China

* Corresponding author

Email: wangyu110016@163.com (YW)

**Contents**

[Table S1. Incidence and DALYs of hand, hip, knee, and other site osteoarthritis among older adults in 1990 and 2021, and their estimated annual percentage changes from 1990 to 2021. 1](#_Toc192609812)

[Table S2. Age-standardised incidence rates and DALY rates in 1990 and 2021, and estimated annual percentage changes for osteoarthritis in older adults, by country. 14](#_Toc192609813)

[Table S3. Age-standardised incidence rates and DALY rates in 1990 and 2021, and estimated annual percentage changes for hand osteoarthritis in older adults, by country. 31](#_Toc192609814)

[Table S4. Age-standardised incidence rates and DALY rates in 1990 and 2021, and estimated annual percentage changes for hip osteoarthritis in older adults, by country. 41](#_Toc192609815)

[Table S5. Age-standardised incidence rates and DALY rates in 1990 and 2021, and estimated annual percentage changes for knee osteoarthritis in older adults, by country. 51](#_Toc192609816)

[Table S6. Age-standardised incidence rates and DALY rates in 1990 and 2021, and estimated annual percentage changes for other sites osteoarthritis in older adults, by country. 61](#_Toc192609817)

[Table S7. Age-standardised DALYs attributable to high BMI for osteoarthritis, hip osteoarthritis, and knee osteoarthritis among older adults in 1990 and 2021, and their proportions. 71](#_Toc192609818)

[Table S8. Age-standardised DALYs attributable to high BMI for osteoarthritis, hip osteoarthritis, and knee osteoarthritis among in 1990 and 2021, and their proportions, by country. 75](#_Toc192609819)

[Table S9. Projected age-standardised incidence and DALY rates for osteoarthritis and its four anatomical sites by sex from 2022 to 2040 based on the BAPC model. 85](#_Toc192609820)

[Figure S1. Age-standardised incidence and DALY rates (per 100,000 population) and estimated annual percentage change. 94](#_Toc192609821)

[Figure S2. Age-standardised incidence and DALY rates and estimated annual percentage change in hand osteoarthritis 95](#_Toc192609822)

[Figure S3. Age-standardised incidence and DALY rates and estimated annual percentage change in hip osteoarthritis 96](#_Toc192609823)

[Figure S4. Age-standardised incidence and DALY rates and estimated annual percentage change in knee osteoarthritis 97](#_Toc192609824)

[Figure S5. Age-standardised incidence and DALY rates and estimated annual percentage change in other sites osteoarthritis 98](#_Toc192609825)

[Figure S6. EAPCs in age-standardised incidence and DALY rates for osteoarthritis and its four anatomical sites across different age groups of older adults globally and in 21 GBD regions from 1990 to 2021. 99](#_Toc192609826)

[Figure S7. Age-standardised incidence and DALY rates of OA and its four anatomical sites by SDI 100](#_Toc192609827)

[Figure S8. Age-standardised incidence and DALY rates for osteoarthritis among older adults in 204 countries and territories in 2021, and their estimated annual percentage changes (1990-2021), by SDI in 2021. 101](#_Toc192609828)

[Figure S9. Age-standardised incidence and DALY rates for hand osteoarthritis among older adults in 204 countries and territories in 2021, and their estimated annual percentage changes (1990-2021), by SDI in 2021. 102](#_Toc192609829)

[Figure S10. Age-standardised incidence and DALY rates for hip osteoarthritis among older adults in 204 countries and territories in 2021, and their estimated annual percentage changes (1990-2021), by SDI in 2021. 103](#_Toc192609830)

[Figure S11. Age-standardised incidence and DALY rates for knee osteoarthritis among older adults in 204 countries and territories in 2021, and their estimated annual percentage changes (1990-2021), by SDI in 2021. 104](#_Toc192609831)

[Figure S12. Age-standardised incidence and DALY rates for other sites osteoarthritis among older adults in 204 countries and territories in 2021, and their estimated annual percentage changes (1990-2021), by SDI in 2021. 105](#_Toc192609832)

[Figure S13. Proportion and trend of the osteoarthritis burden attributable to high BMI among older adults in 1990 and 2021, globally and across 21 GBD regions. 106](#_Toc192609833)

[Figure S14. Proportion and trend of the osteoarthritis burden attributable to high BMI among older adults in 1990 and 2021, at the national level. 107](#_Toc192609834)

**Table** **S1. Incidence and DALYs of hand, hip, knee, and other site osteoarthritis among older adults in 1990 and 2021, and their estimated annual percentage changes from 1990 to 2021.**

| **Characteristics** | | **Incidence** | | | | | **DALYs** | | | | |
| --- | --- | --- | --- | --- | --- | --- | --- | --- | --- | --- | --- |
|  | **Site of osteoarthritis** | **Number of cases, 1990** | **Age- standardised rate per 100,000 population, 1990** | **Number of cases, 2021** | **Age- standardised rate per 100,000 population, 2021** | **Estimated annual percentage change, 1990–2021** | **Number of cases, 1990** | **Age- standardised rate per 100,000 population, 1990** | **Number of cases, 2021** | **Age- standardised rate per 100,000 population, 2021** | **Estimated annual percentage change, 1990–2021** |
| Global | Hand | 801425.19 (397196.3 to 1415309.2) | 246.13 (121.6 to 435.37) | 2144066.61 (1066018.85 to 3775070.13) | 278.37 (138.14 to 490.63) | 0.29 (0.22 to 0.35) | 1139897.44 (524804.01 to 2348554.24) | 350.07 (160.74 to 722.19) | 3071942.05 (1418456.93 to 6339314.89) | 398.84 (183.86 to 823.7) | 0.49 (0.41 to 0.58) |
| Global | Hip | 212792.59 (113611.5 to 363518.2) | 65.35 (34.69 to 112) | 540023.21 (283400.49 to 933698.04) | 70.11 (36.66 to 121.47) | 0.16 (0.12 to 0.19) | 231709.29 (110148 to 468279.12) | 71.16 (33.63 to 144.23) | 576557.75 (272978.39 to 1165460.74) | 74.86 (35.31 to 151.59) | 0.24 (0.21 to 0.27) |
| Global | Knee | 3054148.64 (2200172.57 to 4146258.76) | 937.96 (674.8 to 1274.58) | 7478349.27 (5369036.97 to 10147887.85) | 970.95 (696.5 to 1318.36) | 0.02 (-0.02 to 0.07) | 2060496.14 (995729.28 to 4121883.72) | 632.8 (305.2 to 1267.09) | 5200673.76 (2515827.52 to 10394733.38) | 675.23 (326.24 to 1350.42) | 0.23 (0.21 to 0.25) |
| Global | Other | 411984.49 (238905.7 to 558049.51) | 126.52 (73.08 to 171.83) | 975487.36 (566006.35 to 1319345.37) | 126.65 (73.3 to 171.59) | 0.00 (0.00 to 0.00) | 408320.4 (189685.34 to 868619.58) | 125.4 (57.99 to 267.32) | 1024865.88 (478103.81 to 2176080.16) | 133.06 (61.9 to 282.91) | 0.22 (0.20 to 0.23) |
|  |  |  |  |  |  |  |  |  |  |  |  |
| **Sex** |  |  |  |  |  |  |  |  |  |  |  |
| Male | Hand | 255859.42 (123351.82 to 459176.18) | 182.69 (87.59 to 328.82) | 740907.1 (362686.16 to 1320572.65) | 212.14 (103.51 to 378.76) | 0.21 (0.09 to 0.32) | 338191.91 (154070.77 to 696923.88) | 241.48 (109.46 to 498.8) | 990995.08 (452913.54 to 2044856.18) | 283.74 (129.3 to 586.29) | 0.38 (0.28 to 0.48) |
| Male | Hip | 90904.88 (47517.34 to 158413.9) | 64.91 (33.62 to 113.67) | 249082.28 (128591.73 to 435170.48) | 71.32 (36.62 to 124.97) | 0.26 (0.23 to 0.29) | 93902.2 (44437.64 to 191146.48) | 67.05 (31.44 to 137.1) | 256859.5 (121078.98 to 522412.66) | 73.54 (34.47 to 149.98) | 0.35 (0.33 to 0.38) |
| Male | Knee | 1136797.88 (814372.66 to 1545832.5) | 811.71 (580.23 to 1105.52) | 2981133.05 (2133412.62 to 4051949.95) | 853.57 (610.02 to 1161.29) | 0.07 (0.02 to 0.12) | 709716.96 (341549.11 to 1421072.45) | 506.76 (243.06 to 1016.36) | 1907126.65 (918273.16 to 3821342.18) | 546.05 (262.38 to 1095.23) | 0.24 (0.22 to 0.27) |
| Male | Other | 179037.25 (104511.47 to 241030.01) | 127.84 (74.17 to 172.79) | 446324.4 (260044.02 to 601290.9) | 127.79 (74.17 to 172.6) | -0.01 (-0.01 to 0.00) | 179435.86 (83194.62 to 382106.64) | 128.12 (59 to 273.7) | 481068 (223770.83 to 1024608.82) | 137.74 (63.81 to 293.94) | 0.26 (0.25 to 0.28) |
|  |  |  |  |  |  |  |  |  |  |  |  |
| Female | Hand | 545565.76 (269793.71 to 957676.51) | 294 (144.84 to 517.12) | 1403159.51 (698626.4 to 2449401.12) | 333.33 (165.57 to 582.6) | 0.37 (0.32 to 0.43) | 801705.52 (370656.38 to 1650884.59) | 432.03 (199.1 to 891) | 2080946.96 (963208.74 to 4291577.28) | 494.34 (228.36 to 1020.45) | 0.60 (0.47 to 0.72) |
| Female | Hip | 121887.72 (66376.69 to 205057.29) | 65.68 (35.5 to 110.98) | 290940.92 (154674.54 to 496880.64) | 69.11 (36.56 to 118.37) | 0.07 (0.02 to 0.13) | 137807.09 (65303.01 to 277759.02) | 74.26 (34.92 to 150.24) | 319698.25 (151577.19 to 644014.03) | 75.95 (35.83 to 153.36) | 0.16 (0.13 to 0.20) |
| Female | Knee | 1917350.76 (1384898.82 to 2597333.7) | 1033.24 (745.06 to 1401.38) | 4497216.22 (3235002.79 to 6084022.83) | 1068.34 (767.66 to 1446.44) | 0.02 (-0.02 to 0.07) | 1350779.18 (653949.64 to 2702039.58) | 727.92 (351.55 to 1457.84) | 3293547.11 (1597059.63 to 6574893.77) | 782.4 (378.8 to 1563.1) | 0.26 (0.25 to 0.27) |
| Female | Other | 232947.24 (134223.78 to 317228.28) | 125.53 (71.95 to 171.55) | 529162.96 (305422.03 to 719938.77) | 125.71 (72.3 to 171.42) | 0.00 (0.00 to 0.00) | 228884.54 (106526.46 to 486292.27) | 123.34 (57.06 to 262.79) | 543797.88 (255077.9 to 1153949.13) | 129.18 (60.36 to 274.63) | 0.18 (0.16 to 0.20) |
|  |  |  |  |  |  |  |  |  |  |  |  |
| **GBD regions** | | |  |  |  |  |  |  |  |  |  |
| East Asia | Hand | 136685.72 (66871.2 to 241426.96) | 203.34 (98.73 to 360.6) | 569311.82 (285656.58 to 998101.66) | 280.88 (140.42 to 493.4) | 0.82 (0.65 to 1.00) | 122768.38 (54904.06 to 255214.7) | 182.64 (81 to 381.15) | 597231.17 (271828.18 to 1240245.65) | 294.66 (133.61 to 612.98) | 1.52 (1.34 to 1.71) |
| East Asia | Hip | 25789.64 (12854.73 to 46039.21) | 38.37 (18.79 to 69.12) | 105688.96 (52381.75 to 188811.96) | 52.14 (25.62 to 93.58) | 1.23 (1.13 to 1.33) | 21687.96 (10028.19 to 44217.92) | 32.26 (14.63 to 66.4) | 88134.06 (40709.88 to 180360.4) | 43.48 (19.89 to 89.4) | 1.18 (1.08 to 1.27) |
| East Asia | Knee | 576785.87 (404318.08 to 801227.25) | 858.07 (599.64 to 1194.57) | 1846023.85 (1296251.47 to 2553529.86) | 910.77 (638.43 to 1261.38) | 0.07 (-0.06 to 0.20) | 439060.24 (212479.2 to 870205.41) | 653.18 (314.76 to 1297.3) | 1427590.67 (689476.04 to 2839966.71) | 704.33 (339.36 to 1402.78) | 0.41 (0.30 to 0.52) |
| East Asia | Other | 85443.61 (49685.72 to 115647.49) | 127.11 (73.27 to 173.04) | 258052.38 (150798.95 to 348079.34) | 127.32 (74.02 to 172.3) | 0.00 (0.00 to 0.01) | 82564.72 (37821.01 to 176755.46) | 122.83 (55.7 to 264.18) | 268848.32 (124791.19 to 573544.5) | 132.64 (61.23 to 283.7) | 0.27 (0.25 to 0.29) |
| South Asia | Hand | 93886.02 (47317.72 to 164114.34) | 232.61 (116.49 to 407.98) | 334641.78 (167674.35 to 579462.05) | 279.8 (139.72 to 485.38) | 0.62 (0.60 to 0.65) | 101009.35 (46593.31 to 207811.55) | 249.54 (114.59 to 514.36) | 420872.5 (194552.69 to 866246.01) | 351.34 (162.02 to 724.01) | 1.15 (1.10 to 1.19) |
| South Asia | Hip | 14426.02 (7115.21 to 25839.64) | 35.74 (17.34 to 64.57) | 54257.3 (26959.61 to 96844.65) | 45.37 (22.35 to 81.34) | 0.73 (0.60 to 0.87) | 13865.91 (6415.71 to 27989.98) | 34.39 (15.72 to 69.82) | 53445.27 (24777.55 to 108222.23) | 44.66 (20.58 to 90.68) | 0.95 (0.84 to 1.06) |
| South Asia | Knee | 362646.49 (262304.14 to 491146.7) | 898.43 (648.1 to 1219.18) | 1149947.97 (832579.39 to 1555107.51) | 961.47 (695.06 to 1301.66) | 0.20 (0.18 to 0.23) | 208229.67 (100158.34 to 414587.58) | 514.7 (246.75 to 1026.28) | 690800.17 (334561.7 to 1375762.41) | 576.32 (278.64 to 1148.75) | 0.35 (0.32 to 0.39) |
| South Asia | Other | 51160.41 (29923.81 to 69193.22) | 126.75 (73.55 to 172.33) | 151343.24 (88239.06 to 204788.41) | 126.54 (73.44 to 171.75) | -0.01 (-0.01 to 0.00) | 48072.81 (22321.35 to 102575.86) | 119.09 (54.95 to 254.86) | 151086.34 (70108.13 to 323943.26) | 126.29 (58.39 to 271.25) | 0.22 (0.20 to 0.24) |
| Southeast Asia | Hand | 41203.94 (20322.83 to 72778.62) | 222.54 (108.26 to 395.94) | 141101.46 (70707.46 to 246838.97) | 275.81 (137.2 to 484.41) | 0.67 (0.65 to 0.69) | 43473.09 (19928.77 to 89695.1) | 234.8 (106.14 to 487.62) | 171847.99 (78613.89 to 353874.1) | 335.91 (152.6 to 694.01) | 1.17 (1.14 to 1.19) |
| Southeast Asia | Hip | 9129.84 (4604.96 to 16172.62) | 49.31 (24.16 to 88.7) | 28735.87 (14418.02 to 50934.87) | 56.17 (27.72 to 100.43) | 0.49 (0.45 to 0.52) | 7490.48 (3500.49 to 15350.73) | 40.46 (18.28 to 84.23) | 23922.79 (11108.73 to 48946.89) | 46.76 (21.31 to 96.53) | 0.58 (0.54 to 0.61) |
| Southeast Asia | Knee | 122264.61 (86521.13 to 168666.76) | 660.34 (464.19 to 915.32) | 371752.66 (264086.02 to 510530) | 726.67 (514.25 to 1000.68) | 0.35 (0.33 to 0.36) | 77480.48 (37191.87 to 153936.38) | 418.47 (198.83 to 835.57) | 241736.83 (116202.01 to 483811.62) | 472.53 (225.84 to 948.38) | 0.46 (0.44 to 0.48) |
| Southeast Asia | Other | 23310.09 (13440.53 to 31681.59) | 125.9 (71.37 to 173.01) | 64751.72 (37450.97 to 87654.88) | 126.57 (72.47 to 172.48) | 0.02 (0.01 to 0.02) | 22229.32 (10290.64 to 47727.25) | 120.06 (54.51 to 260.1) | 64587.3 (29683.22 to 138144.21) | 126.25 (57.36 to 271.46) | 0.23 (0.21 to 0.25) |
| Central Asia | Hand | 8438.63 (4013.24 to 15071.44) | 241.54 (111.34 to 438.33) | 15174.25 (7353.14 to 26736.86) | 252.04 (119.36 to 449.45) | 0.00 (-0.11 to 0.10) | 19547.23 (8965.36 to 40052.18) | 559.49 (251.33 to 1157.68) | 39884.58 (18287.25 to 82016.12) | 662.47 (299.36 to 1371.62) | 0.68 (0.56 to 0.80) |
| Central Asia | Hip | 4170.02 (2009.21 to 7502.6) | 119.36 (55.02 to 219.66) | 7525.48 (3687.88 to 13495.65) | 125 (59.29 to 227.97) | 0.30 (0.19 to 0.41) | 2747.84 (1276.83 to 5666.55) | 78.65 (34.57 to 166.47) | 5095.22 (2380.67 to 10487.49) | 84.63 (37.97 to 177.56) | 0.45 (0.36 to 0.54) |
| Central Asia | Knee | 26125.1 (18675.57 to 35415.08) | 747.77 (526.91 to 1024.29) | 48162.21 (34714.52 to 64925.31) | 799.96 (570.55 to 1086.72) | 0.13 (0.10 to 0.16) | 15146.94 (7200.5 to 30654.28) | 433.55 (201.36 to 887.28) | 27172.55 (13033.83 to 54760.35) | 451.33 (212.79 to 917.21) | 0.23 (0.19 to 0.26) |
| Central Asia | Other | 4382.74 (2519 to 5964.8) | 125.45 (69.31 to 175.12) | 7631.06 (4439.32 to 10318.26) | 126.75 (71.58 to 174.72) | 0.00 (-0.01 to 0.01) | 4560.32 (2109.09 to 9625.93) | 130.53 (57.82 to 281.08) | 7886.63 (3697.61 to 16773.04) | 131 (59.45 to 282.84) | 0.16 (0.11 to 0.21) |
| High-income Asia Pacific | Hand | 59938.26 (30119.8 to 104169.22) | 345 (171.42 to 603.24) | 148042.35 (73076.53 to 261682.8) | 320.87 (157.24 to 569.35) | -0.45 (-0.57 to -0.34) | 81914.24 (37660.69 to 168800.21) | 471.49 (214.59 to 976.25) | 261304.34 (120650.67 to 535682.02) | 566.35 (260.03 to 1164.16) | 1.16 (0.60 to 1.73) |
| High-income Asia Pacific | Hip | 9330.75 (5131.28 to 15608.26) | 53.71 (28.73 to 91.26) | 23754.78 (12683.16 to 40529.81) | 51.49 (27.01 to 88.7) | -0.16 (-0.20 to -0.13) | 13032.81 (6207 to 26573.65) | 75.02 (34.84 to 154.81) | 39308.08 (18684.6 to 79886.28) | 85.2 (39.92 to 174.35) | 0.47 (0.39 to 0.55) |
| High-income Asia Pacific | Knee | 226326.27 (165112.29 to 302807.87) | 1302.72 (945.8 to 1749.16) | 559674.85 (405509.02 to 751054.25) | 1213.04 (876.2 to 1631.53) | -0.33 (-0.39 to -0.27) | 166130.98 (80513.24 to 331736.88) | 956.24 (460.23 to 1915.97) | 450474.52 (218020.3 to 906075.46) | 976.36 (470.56 to 1967.88) | 0.12 (0.09 to 0.16) |
| High-income Asia Pacific | Other | 21763.2 (12584.1 to 29745.83) | 125.27 (71.17 to 173.17) | 57614.34 (33041.96 to 78773.71) | 124.87 (70.85 to 171.93) | -0.01 (-0.02 to -0.01) | 21483.15 (10043.87 to 45968.59) | 123.66 (56.69 to 267.02) | 62675.04 (29536.57 to 132392.8) | 135.84 (63.29 to 288.5) | 0.31 (0.30 to 0.32) |
| Oceania | Hand | 381.03 (191.02 to 667.24) | 193.89 (83.9 to 366.3) | 1094.71 (542.93 to 1909.72) | 223.95 (101.92 to 408.61) | 0.51 (0.48 to 0.53) | 443.07 (202.3 to 918.32) | 225.46 (89.24 to 498.52) | 1399.04 (642.07 to 2898.42) | 286.21 (121.39 to 614.94) | 0.81 (0.78 to 0.84) |
| Oceania | Hip | 107.15 (54.61 to 190.05) | 54.53 (20.91 to 111.48) | 286.84 (145.9 to 503.57) | 58.68 (25.2 to 112.42) | 0.22 (0.18 to 0.27) | 90.76 (42.4 to 187.67) | 46.19 (15.57 to 110.18) | 245.04 (113.29 to 501.25) | 50.13 (19.11 to 111.93) | 0.20 (0.10 to 0.30) |
| Oceania | Knee | 1523.87 (1081.89 to 2082.09) | 775.43 (518.21 to 1105.99) | 4022.28 (2862.65 to 5513.63) | 822.87 (564.37 to 1158.14) | 0.16 (0.15 to 0.18) | 1027.03 (490.57 to 2052.72) | 522.61 (228.03 to 1090.72) | 2759.17 (1331.94 to 5484.02) | 564.46 (258.05 to 1152) | 0.22 (0.19 to 0.26) |
| Oceania | Other | 247.39 (144.77 to 336.37) | 125.89 (62.16 to 190.47) | 616.33 (359.58 to 836.21) | 126.09 (66.15 to 183.07) | 0.00 (-0.01 to 0.01) | 233.49 (108.27 to 495.59) | 118.81 (45.21 to 275.4) | 607.53 (279.92 to 1293.03) | 124.29 (50.75 to 279.34) | 0.10 (0.05 to 0.16) |
| Australasia | Hand | 5486.61 (2676.56 to 9722.6) | 245.9 (115.46 to 444.5) | 12832.95 (6227.22 to 22729.98) | 246.97 (116.88 to 443.16) | 0.05 (0.03 to 0.06) | 9302.94 (4272.93 to 19222.79) | 416.94 (185.81 to 873.81) | 22692.2 (10506.21 to 46542.03) | 436.7 (198.34 to 903.86) | 0.12 (0.09 to 0.15) |
| Australasia | Hip | 1502.57 (854.43 to 2469.98) | 67.34 (35.77 to 115.15) | 4188.31 (2316.87 to 6942.08) | 80.6 (42.79 to 136.78) | 0.65 (0.59 to 0.72) | 2289.82 (1078.53 to 4590.42) | 102.63 (45.5 to 211.78) | 7049.18 (3330.59 to 14333.07) | 135.66 (61.94 to 280.39) | 0.89 (0.81 to 0.98) |
| Australasia | Knee | 22998.43 (16676.09 to 31184.93) | 1030.76 (736.1 to 1413.27) | 56983.05 (40830.37 to 76923.27) | 1096.62 (778.16 to 1490.86) | 0.07 (0.01 to 0.13) | 16195.04 (7854.31 to 32446.73) | 725.84 (344.28 to 1470.13) | 41636.14 (20202.6 to 83826.4) | 801.27 (383.45 to 1624.17) | 0.26 (0.23 to 0.30) |
| Australasia | Other | 2812.3 (1628.46 to 3842.55) | 126.04 (69.48 to 177.75) | 6601.19 (3816.73 to 8941.32) | 127.04 (71.14 to 175.68) | 0.02 (0.02 to 0.02) | 2824.67 (1299.87 to 5978.78) | 126.6 (55.13 to 274.84) | 7340.64 (3429.87 to 15518.57) | 141.27 (63.82 to 303.39) | 0.32 (0.26 to 0.37) |
| Eastern Europe | Hand | 80532.59 (39997.86 to 139774.72) | 344.36 (169.36 to 600.82) | 119233.79 (59239.72 to 206371.97) | 356.37 (175.63 to 619.48) | 0.04 (-0.02 to 0.10) | 155025.06 (71530.12 to 319185.31) | 662.89 (303.63 to 1369.59) | 230152.52 (106700.14 to 473712.79) | 687.89 (317 to 1419.88) | 0.39 (0.30 to 0.48) |
| Eastern Europe | Hip | 29490.52 (14318.03 to 52937.75) | 126.1 (60.23 to 228.3) | 50572.84 (24710.35 to 89711.86) | 151.15 (72.94 to 269.89) | 0.79 (0.66 to 0.91) | 18902.89 (8782.91 to 38652.18) | 80.83 (36.77 to 166.93) | 32209.68 (14948.98 to 65831.13) | 96.27 (43.97 to 198.27) | 0.81 (0.72 to 0.91) |
| Eastern Europe | Knee | 206708.59 (149326.92 to 279797.17) | 883.89 (635.29 to 1200.86) | 309843.86 (225116.73 to 420934.53) | 926.07 (670.06 to 1261.91) | 0.03 (-0.02 to 0.08) | 124725.95 (60195.15 to 251621.55) | 533.33 (255.34 to 1080.15) | 190587.2 (91965.03 to 380653.08) | 569.63 (273.09 to 1141.33) | 0.31 (0.28 to 0.34) |
| Eastern Europe | Other | 29480.53 (17162.55 to 40038.18) | 126.06 (72.29 to 172.89) | 42453.17 (24709.06 to 57339.19) | 126.89 (72.93 to 172.79) | -0.01 (-0.02 to 0.00) | 30908.82 (14314.85 to 65725.18) | 132.17 (60.21 to 283.2) | 46456.69 (21701.67 to 98624.88) | 138.85 (64 to 296.62) | 0.32 (0.26 to 0.37) |
| Western Europe | Hand | 121194.97 (58362.95 to 216378.2) | 217.43 (103.86 to 389.83) | 196112.25 (93324.74 to 351237.77) | 217.95 (103.05 to 391.65) | -0.01 (-0.05 to 0.04) | 214734.1 (98535.42 to 442399.14) | 385.24 (175.67 to 796.03) | 360341.24 (165829.55 to 739971.09) | 400.47 (183.41 to 824.25) | 0.10 (0.06 to 0.14) |
| Western Europe | Hip | 45305.88 (25806.99 to 73936.67) | 81.28 (45.74 to 133.61) | 78206.76 (43802.15 to 129293.23) | 86.92 (48.23 to 144.48) | 0.17 (0.13 to 0.20) | 66520.05 (31476.42 to 134838.67) | 119.34 (55.85 to 243.2) | 125119.6 (59434.94 to 253322.01) | 139.05 (65.52 to 282.63) | 0.52 (0.45 to 0.58) |
| Western Europe | Knee | 585383.06 (424409.79 to 787021.91) | 1050.2 (759.12 to 1415.08) | 960107.83 (692652.02 to 1286972.17) | 1067.03 (767.98 to 1432.77) | -0.12 (-0.17 to -0.07) | 394715.37 (190209.16 to 799818.73) | 708.14 (339.71 to 1438.06) | 667595.04 (321969.35 to 1350380.62) | 741.94 (356.59 to 1503.3) | 0.14 (0.11 to 0.16) |
| Western Europe | Other | 69784.15 (40512.75 to 95318.97) | 125.2 (71.98 to 172.1) | 112675.87 (64896.87 to 154131.2) | 125.22 (71.57 to 172.15) | 0.00 (-0.01 to 0.00) | 71572.83 (33401.79 to 151199.19) | 128.4 (59.28 to 272.63) | 124823.78 (58654.52 to 262502.14) | 138.72 (64.66 to 292.85) | 0.25 (0.24 to 0.27) |
| Central Europe | Hand | 37249.31 (18176.11 to 66105.03) | 283.48 (136.32 to 506.94) | 65879.4 (32377.42 to 116759.07) | 295.09 (143.45 to 526.01) | 0.12 (0.09 to 0.16) | 60834.05 (28116.46 to 125681.39) | 462.97 (211.48 to 961.79) | 121325.65 (56018.8 to 250906.67) | 543.45 (248.85 to 1128.29) | 0.61 (0.56 to 0.65) |
| Central Europe | Hip | 16412.19 (7977.58 to 29584.86) | 124.9 (59.39 to 227.73) | 33251.73 (16082.25 to 59370.22) | 148.94 (70.93 to 268.08) | 0.70 (0.63 to 0.76) | 10813.21 (5043.59 to 22262.46) | 82.29 (37.33 to 171.67) | 21950.19 (10221.34 to 44809.03) | 98.32 (44.9 to 202.58) | 0.72 (0.68 to 0.76) |
| Central Europe | Knee | 111559.91 (80502.33 to 150376.78) | 849.02 (608.43 to 1150.23) | 195120.43 (140537.73 to 264203.44) | 874 (626.22 to 1187.96) | 0.02 (-0.02 to 0.05) | 65714.11 (31536.47 to 132178.63) | 500.11 (237.36 to 1011.37) | 120633.62 (58055.65 to 242456.68) | 540.35 (257.94 to 1090.36) | 0.29 (0.27 to 0.30) |
| Central Europe | Other | 16638.19 (9762.48 to 22515.84) | 126.62 (72.83 to 173.61) | 28243.41 (16423.39 to 38215.48) | 126.51 (72.44 to 172.9) | -0.01 (-0.01 to 0.00) | 17177.5 (8014.47 to 36582.19) | 130.73 (59.67 to 281.27) | 31423.38 (14683.16 to 66699.14) | 140.75 (64.71 to 301.04) | 0.31 (0.28 to 0.34) |
| High-income North America | Hand | 102717.93 (50174.02 to 180144.95) | 298.95 (144.75 to 526.71) | 205995.08 (102122.39 to 361094.49) | 321.1 (158.21 to 564.7) | 0.02 (-0.09 to 0.13) | 178823.93 (82600.81 to 369355.63) | 520.44 (238.76 to 1078.43) | 349847.64 (161675.19 to 719679.75) | 545.33 (250.79 to 1124.41) | 0.05 (-0.26 to 0.35) |
| High-income North America | Hip | 26160.99 (14757.75 to 43367.49) | 76.14 (42.26 to 127.41) | 60498.08 (33827.61 to 98992.13) | 94.3 (52.17 to 155.27) | 0.00 (-0.34 to 0.35) | 47560.45 (22849.07 to 96051.73) | 138.42 (65.64 to 281.32) | 98939.43 (47656.43 to 199263.51) | 154.22 (73.62 to 311.97) | 0.37 (0.25 to 0.48) |
| High-income North America | Knee | 333006.37 (238081.91 to 454492.79) | 969.17 (690.13 to 1326.59) | 659368.22 (472104.68 to 897115.51) | 1027.81 (733.81 to 1401.3) | 0.01 (-0.20 to 0.21) | 254105.09 (122735.39 to 509139.67) | 739.54 (355.21 to 1485.86) | 484011.54 (234598.69 to 966650.5) | 754.46 (364.21 to 1509.8) | -0.32 (-0.53 to -0.10) |
| High-income North America | Other | 44279.39 (25671.14 to 59326.81) | 128.87 (73.8 to 174.06) | 81925 (47452.93 to 110160.59) | 127.7 (73.3 to 172.73) | -0.03 (-0.04 to -0.02) | 43940.61 (21155.39 to 91970.35) | 127.88 (60.74 to 269.4) | 84993.54 (41236.72 to 177528.08) | 132.49 (63.66 to 278.02) | 0.10 (0.05 to 0.16) |
| Andean Latin America | Hand | 3615.11 (1742.18 to 6456.73) | 225.79 (103.76 to 413.22) | 11857.23 (5733.38 to 21088.34) | 236.3 (111.32 to 425.98) | 0.08 (0.03 to 0.14) | 5941.29 (2729.81 to 12257.44) | 371.07 (164.16 to 779.23) | 20938.91 (9585.81 to 43187.59) | 417.29 (187.23 to 868.85) | 0.33 (0.24 to 0.42) |
| Andean Latin America | Hip | 1001.06 (495.47 to 1798.86) | 62.52 (28.28 to 117.66) | 3694.71 (1820.83 to 6601.19) | 73.63 (34.64 to 134.77) | 0.58 (0.56 to 0.61) | 872.52 (404.11 to 1807.3) | 54.49 (22.84 to 118.2) | 3278.88 (1537.56 to 6632.53) | 65.35 (29.13 to 135.4) | 0.67 (0.64 to 0.69) |
| Andean Latin America | Knee | 16849.29 (12276.19 to 22670.36) | 1052.35 (753.22 to 1434.46) | 55815.54 (40365.26 to 75187.64) | 1112.35 (796.61 to 1509.17) | 0.16 (0.14 to 0.17) | 10809.46 (5175.18 to 21906.57) | 675.12 (314.48 to 1386.44) | 37730.73 (18370.53 to 75524.7) | 751.94 (360.83 to 1515.91) | 0.39 (0.37 to 0.41) |
| Andean Latin America | Other | 2007.47 (1167.08 to 2732.4) | 125.38 (68.77 to 177.18) | 6342.88 (3700.32 to 8594.27) | 126.41 (71.39 to 174.94) | 0.01 (0.01 to 0.02) | 2042.2 (945.64 to 4324.77) | 127.55 (55.36 to 278.28) | 6863.12 (3175.23 to 14756.36) | 136.78 (61.1 to 298.86) | 0.26 (0.25 to 0.28) |
| Central Latin America | Hand | 15772.88 (7686.81 to 27853.43) | 245.78 (117.12 to 439.15) | 57184.7 (28220.63 to 101136.69) | 269.43 (131.42 to 479.47) | 0.29 (0.27 to 0.31) | 23125.61 (10628.52 to 47725.58) | 360.35 (162.48 to 750.38) | 96644.91 (44894.03 to 199266.98) | 455.36 (209.57 to 943.01) | 0.84 (0.80 to 0.87) |
| Central Latin America | Hip | 3922.06 (1952.58 to 6972.32) | 61.12 (29.09 to 111.23) | 14785.69 (7311.78 to 26474.52) | 69.66 (33.67 to 126.25) | 0.32 (0.25 to 0.38) | 3388.67 (1579.08 to 6920.26) | 52.8 (23.41 to 110.41) | 12798.52 (5981.05 to 26218.81) | 60.3 (27.47 to 125.04) | 0.33 (0.26 to 0.40) |
| Central Latin America | Knee | 68241.51 (49625.86 to 91965.86) | 1063.37 (766.5 to 1442.35) | 232611.99 (168532.89 to 313225.98) | 1095.99 (790.28 to 1480.99) | 0.09 (0.07 to 0.10) | 43378.49 (20917.3 to 87125.18) | 675.94 (321.54 to 1366.67) | 155510.22 (75336.11 to 311854.35) | 732.71 (352.43 to 1474.51) | 0.27 (0.26 to 0.28) |
| Central Latin America | Other | 8110.41 (4726.59 to 11003.3) | 126.38 (71.57 to 174.69) | 26849.67 (15646.05 to 36349.19) | 126.51 (72.57 to 173.03) | 0.00 (0.00 to 0.00) | 8484.83 (3968.11 to 18052.51) | 132.21 (59.92 to 285.44) | 29416.66 (13634.32 to 62790.88) | 138.6 (63.17 to 298.17) | 0.17 (0.16 to 0.18) |
| Caribbean | Hand | 4907.49 (2385.15 to 8789.04) | 217.28 (101.41 to 397.36) | 11020.22 (5350.72 to 19689.29) | 231.77 (109.54 to 419.92) | 0.22 (0.20 to 0.23) | 7853.9 (3589.27 to 16162.19) | 347.73 (153.76 to 726.71) | 19049.69 (8817.8 to 39335.08) | 400.65 (181.6 to 835.5) | 0.45 (0.45 to 0.46) |
| Caribbean | Hip | 1437.42 (716.65 to 2553.91) | 63.64 (29.45 to 117.55) | 3549.24 (1751.48 to 6352.96) | 74.65 (35.13 to 136.94) | 0.58 (0.54 to 0.63) | 1292.26 (597.99 to 2647.61) | 57.22 (24.4 to 121.78) | 3163.95 (1480.23 to 6452.48) | 66.54 (29.57 to 139.06) | 0.58 (0.54 to 0.61) |
| Caribbean | Knee | 23529.93 (17083.02 to 31812.48) | 1041.8 (745.06 to 1424.07) | 50686.85 (36580.61 to 68483.05) | 1066.02 (761.48 to 1451.13) | 0.12 (0.10 to 0.13) | 15391.93 (7372.79 to 31076.58) | 681.48 (319.02 to 1391.31) | 34944.38 (16916.14 to 70446.32) | 734.94 (350.43 to 1492.58) | 0.28 (0.27 to 0.30) |
| Caribbean | Other | 2842.78 (1640.92 to 3854.25) | 125.87 (69.18 to 176.12) | 5985.83 (3462.64 to 8105.98) | 125.89 (70.42 to 174.23) | 0.00 (0.00 to 0.01) | 2955.8 (1383.4 to 6291.21) | 130.87 (58.06 to 285.52) | 6554.14 (3064.87 to 13974.02) | 137.84 (62.2 to 298.81) | 0.19 (0.17 to 0.21) |
| Tropical Latin America | Hand | 21049.28 (10532.6 to 36964.47) | 296.02 (145.31 to 525.16) | 73070.63 (36143.48 to 127137.07) | 328.47 (160.8 to 574.66) | 0.33 (0.30 to 0.35) | 26700.17 (12300.78 to 55352.56) | 375.49 (169.94 to 784.94) | 102392.32 (47464.62 to 210928.58) | 460.27 (211.45 to 952.22) | 0.66 (0.64 to 0.69) |
| Tropical Latin America | Hip | 4760.32 (2395.09 to 8416.08) | 66.94 (32.35 to 120.91) | 18054.65 (8962.56 to 32090.32) | 81.16 (39.46 to 145.84) | 0.68 (0.64 to 0.72) | 3910.05 (1831.88 to 8001.9) | 54.99 (24.6 to 115.02) | 15001.39 (6970.03 to 30790.92) | 67.43 (30.6 to 139.97) | 0.74 (0.70 to 0.78) |
| Tropical Latin America | Knee | 75867.45 (55209.26 to 102363.2) | 1066.93 (769.95 to 1448.39) | 244557.95 (176454.66 to 330945.64) | 1099.34 (789.5 to 1492.75) | 0.10 (0.09 to 0.12) | 46762.36 (22669.9 to 93844.89) | 657.62 (314.67 to 1328.22) | 161522.19 (78024.68 to 322241.27) | 726.08 (348.28 to 1453.55) | 0.35 (0.33 to 0.37) |
| Tropical Latin America | Other | 9049.69 (5269.19 to 12161.02) | 127.27 (72.11 to 174.09) | 28255.1 (16593.89 to 38186.87) | 127.01 (73.46 to 173.39) | -0.01 (-0.01 to -0.01) | 9173.73 (4234 to 19703.56) | 129.01 (57.76 to 280.99) | 31035.02 (14422.36 to 65927.98) | 139.51 (63.78 to 298.63) | 0.27 (0.25 to 0.30) |
| Southern Latin America | Hand | 9561.41 (4623.32 to 16714.99) | 233.25 (109.56 to 413.99) | 19386.75 (9385 to 34582.76) | 239.06 (113.4 to 430.97) | 0.04 (0.03 to 0.06) | 17078.32 (7785.3 to 34752.67) | 416.63 (185.73 to 856.75) | 35468.86 (16332.25 to 72758.81) | 437.37 (198.32 to 903.75) | 0.14 (0.12 to 0.16) |
| Southern Latin America | Hip | 2637.68 (1487.24 to 4338.39) | 64.35 (34.46 to 109.03) | 6187.53 (3450.18 to 10226.17) | 76.3 (41.14 to 128.57) | 0.54 (0.52 to 0.57) | 3788.62 (1765.76 to 7676.77) | 92.42 (41.09 to 191.51) | 9752.18 (4609.57 to 19740.57) | 120.26 (55.21 to 246.85) | 0.88 (0.78 to 0.98) |
| Southern Latin America | Knee | 41958.11 (30495.42 to 56579.33) | 1023.57 (735.61 to 1391.68) | 88641.62 (63628.85 to 119275.02) | 1093.06 (778.54 to 1479.18) | 0.04 (-0.01 to 0.09) | 28788.83 (13915.11 to 57514.62) | 702.3 (333.84 to 1414.59) | 63072.25 (30248.04 to 127253.28) | 777.76 (368.8 to 1577.84) | 0.31 (0.27 to 0.35) |
| Southern Latin America | Other | 5105.18 (2935.5 to 7014.77) | 124.54 (69.04 to 175.18) | 10199.72 (5825.18 to 13889.17) | 125.78 (70 to 174.14) | 0.03 (0.02 to 0.03) | 4967.59 (2303.25 to 10529.25) | 121.18 (53.92 to 261.82) | 10901.17 (5062.86 to 22717.99) | 134.42 (60.72 to 283.81) | 0.34 (0.29 to 0.38) |
| Eastern Sub-Saharan Africa | Hand | 10807.29 (5370.61 to 19115.48) | 199.89 (96.69 to 358.6) | 30155.37 (15060.86 to 53119.31) | 254.68 (125.17 to 452.45) | 0.81 (0.79 to 0.84) | 11904.4 (5411.5 to 24598.09) | 220.18 (97.44 to 460.68) | 37944.82 (17462.18 to 78499.47) | 320.47 (145.3 to 667.63) | 1.27 (1.25 to 1.29) |
| Eastern Sub-Saharan Africa | Hip | 3786.61 (1894.55 to 6752.3) | 70.04 (33.48 to 127.9) | 9350.84 (4632.73 to 16745.73) | 78.97 (38.01 to 143.59) | 0.44 (0.42 to 0.46) | 2920.96 (1355.9 to 5988.71) | 54.02 (23.76 to 113.61) | 7307.21 (3410.1 to 14866.54) | 61.71 (27.84 to 127.59) | 0.50 (0.47 to 0.52) |
| Eastern Sub-Saharan Africa | Knee | 47486.28 (34456.87 to 64133.64) | 878.28 (630.59 to 1195.4) | 108579.62 (79013.5 to 146446.56) | 917.02 (662.67 to 1243.17) | 0.14 (0.13 to 0.16) | 28023.55 (13395.74 to 56238.8) | 518.31 (243.58 to 1048.8) | 66026 (31838.1 to 132066.23) | 557.63 (265.94 to 1121.4) | 0.27 (0.26 to 0.28) |
| Eastern Sub-Saharan Africa | Other | 6793.1 (3930.75 to 9225.81) | 125.64 (70.45 to 174.15) | 14920.2 (8632.27 to 20264.15) | 126.01 (71.37 to 173.51) | 0.01 (0.00 to 0.01) | 6300.74 (2893.7 to 13405.03) | 116.54 (51.59 to 252.17) | 14532.57 (6742.43 to 30994.82) | 122.74 (55.59 to 264.7) | 0.19 (0.18 to 0.21) |
| Southern Sub-Saharan Africa | Hand | 6643.07 (3336.87 to 11555.81) | 314.01 (152.42 to 556.28) | 15647.62 (7844.02 to 27146.5) | 348.53 (170.87 to 611.9) | 0.33 (0.30 to 0.36) | 8921.93 (4103.75 to 18386.6) | 421.73 (188.09 to 881.76) | 21525.28 (9973.8 to 44358) | 479.45 (217.82 to 997.27) | 0.47 (0.41 to 0.53) |
| Southern Sub-Saharan Africa | Hip | 1896.45 (934.84 to 3399.2) | 89.64 (41.4 to 166.17) | 4401.4 (2174.77 to 7893.67) | 98.04 (46.43 to 179.75) | 0.36 (0.34 to 0.38) | 1551.47 (715.65 to 3187.66) | 73.34 (31.39 to 156) | 3607.75 (1674.79 to 7350.72) | 80.36 (35.54 to 167.52) | 0.39 (0.36 to 0.42) |
| Southern Sub-Saharan Africa | Knee | 19609.2 (14153.7 to 26643) | 926.9 (658.05 to 1274.59) | 44203.05 (32146.99 to 59644.74) | 984.58 (708.23 to 1339.23) | 0.18 (0.16 to 0.20) | 12431.94 (5981.3 to 24922.21) | 587.64 (275.61 to 1192.75) | 28057.57 (13581.37 to 56160.83) | 624.95 (297.44 to 1261.31) | 0.22 (0.21 to 0.22) |
| Southern Sub-Saharan Africa | Other | 2675.64 (1568.58 to 3608.2) | 126.47 (70.52 to 176.21) | 5687.19 (3339.45 to 7648.36) | 126.68 (71.88 to 174.22) | 0.01 (0.00 to 0.01) | 2841.79 (1319.77 to 6037.45) | 134.33 (59.06 to 292.67) | 6124.05 (2824.32 to 13060.66) | 136.41 (60.61 to 295.95) | 0.11 (0.09 to 0.12) |
| Western Sub-Saharan Africa | Hand | 14887.62 (7405.72 to 26420.09) | 222.2 (108.03 to 399.1) | 33503.44 (16673.87 to 59251.84) | 245.08 (120.13 to 436.93) | 0.42 (0.31 to 0.53) | 16755.83 (7663.3 to 34517.75) | 250.08 (111.83 to 520.64) | 41305.71 (18964.5 to 85202.59) | 302.15 (136.76 to 627.46) | 0.76 (0.61 to 0.91) |
| Western Sub-Saharan Africa | Hip | 4544.31 (2253.32 to 8106.65) | 67.82 (32.26 to 123.65) | 10040.35 (4961.92 to 17994.79) | 73.45 (35.29 to 133.57) | 0.16 (0.08 to 0.24) | 3548.7 (1640.78 to 7330.21) | 52.96 (23.32 to 111.94) | 8003.85 (3704.13 to 16431.22) | 58.55 (26.23 to 122.05) | 0.24 (0.17 to 0.32) |
| Western Sub-Saharan Africa | Knee | 59757.82 (43193.18 to 80996.45) | 891.88 (638.59 to 1217.22) | 128276.13 (92675.45 to 173443.41) | 938.34 (673.57 to 1274.73) | 0.14 (0.13 to 0.15) | 37408.12 (18064.64 to 74824.28) | 558.31 (265.7 to 1124.78) | 82766.2 (39941.99 to 165214.93) | 605.44 (289.32 to 1214.39) | 0.26 (0.23 to 0.29) |
| Western Sub-Saharan Africa | Other | 8472.44 (4918.44 to 11462.56) | 126.45 (71.37 to 174.24) | 17328.98 (10089.03 to 23484.01) | 126.76 (72.37 to 174) | 0.01 (0.01 to 0.01) | 8215.59 (3820.16 to 17552.35) | 122.62 (55.22 to 265.87) | 17496.55 (8123.74 to 37389.41) | 127.99 (58.14 to 276.29) | 0.11 (0.09 to 0.14) |
| North Africa and Middle East | Hand | 23181.28 (11294.66 to 41724.9) | 187.42 (89.64 to 340.6) | 74583.23 (36523.6 to 132699.02) | 218.32 (105.82 to 390.53) | 0.49 (0.43 to 0.56) | 29263.81 (13405.45 to 60404.24) | 236.6 (106.56 to 492.28) | 107964.91 (49474.72 to 222200.47) | 316.03 (143.55 to 653.13) | 0.93 (0.79 to 1.07) |
| North Africa and Middle East | Hip | 5965.15 (2888.99 to 10821.81) | 48.23 (22.51 to 89.16) | 20487.84 (9926.66 to 37047.72) | 59.97 (28.49 to 109.55) | 0.80 (0.76 to 0.84) | 4636.31 (2132.14 to 9492.79) | 37.48 (16.51 to 78.31) | 16253.54 (7551.57 to 33200.68) | 47.58 (21.61 to 98.23) | 0.82 (0.80 to 0.85) |
| North Africa and Middle East | Knee | 111800.96 (80402.24 to 151303.19) | 903.91 (645.57 to 1229.47) | 331628.05 (241432.13 to 449022.33) | 970.73 (703.89 to 1318.21) | 0.18 (0.16 to 0.20) | 67043.31 (32111.67 to 134599.05) | 542.05 (256.79 to 1094.07) | 206876.75 (99762.41 to 413643.42) | 605.56 (290.21 to 1214.5) | 0.38 (0.36 to 0.39) |
| North Africa and Middle East | Other | 15711.9 (9159.93 to 21170.44) | 127.03 (72.55 to 173.49) | 43637.3 (25747.18 to 58814.07) | 127.73 (74.45 to 173.56) | 0.01 (0.01 to 0.02) | 16050.25 (7415.52 to 34393) | 129.77 (58.6 to 281.02) | 47140.22 (21880.71 to 100480.42) | 137.99 (63.2 to 295.95) | 0.24 (0.23 to 0.26) |
| Central Sub-Saharan Africa | Hand | 3284.72 (1621.84 to 5839.65) | 216.08 (101.56 to 394.13) | 8237.58 (4085.18 to 14625.58) | 235.97 (113.46 to 425.8) | 0.25 (0.19 to 0.30) | 4476.74 (2030.09 to 9291.11) | 294.49 (127.8 to 623.74) | 11807.76 (5446.39 to 24561.58) | 338.24 (151.9 to 712.44) | 0.41 (0.29 to 0.52) |
| Central Sub-Saharan Africa | Hip | 1015.93 (516.33 to 1814.26) | 66.83 (31.1 to 124.97) | 2504 (1251.92 to 4494.58) | 71.73 (33.9 to 132.57) | 0.25 (0.22 to 0.28) | 797.54 (360.08 to 1638.43) | 52.46 (21.3 to 113.13) | 1971.93 (909.77 to 4071.79) | 56.49 (24.39 to 120.28) | 0.26 (0.22 to 0.30) |
| Central Sub-Saharan Africa | Knee | 13719.52 (9925.81 to 18475.16) | 902.5 (640.16 to 1232.99) | 32341.28 (23303.31 to 43257.81) | 926.44 (658.99 to 1250.88) | 0.04 (0.01 to 0.06) | 7927.26 (3790.85 to 15908.15) | 521.47 (241.49 to 1062.86) | 19170.03 (9132.11 to 38673.73) | 549.14 (256.26 to 1118.93) | 0.15 (0.14 to 0.16) |
| Central Sub-Saharan Africa | Other | 1913.85 (1106.97 to 2607.31) | 125.9 (68.59 to 178.23) | 4372.79 (2522.59 to 5979.88) | 125.26 (69.47 to 175.69) | -0.02 (-0.04 to -0.01) | 1719.64 (785.98 to 3652.79) | 113.12 (48.15 to 248.21) | 4073.19 (1868.47 to 8763.72) | 116.68 (51.12 to 256.35) | 0.12 (0.10 to 0.13) |

**Abbreviations: DALY=disability-adjusted life-years.**

**Table S2.** **Age-standardised incidence rates and DALY rates in 1990 and 2021, and estimated annual percentage changes for osteoarthritis in older adults, by country.**

| **Countries** | **Age-standardised incidence rate, 1990** | **Age-standardised incidence rate, 2021** | **EAPC of incidence rate No. (95% CI)** | **Age-standardised DALYs rate, 1990** | **Age-standardised DALYs rate, 2021** | **EAPC of DALYs rate No. (95% CI)** |
| --- | --- | --- | --- | --- | --- | --- |
| Afghanistan | 1155.26 (857.85 to 1502.25) | 1242.55 (925.47 to 1618.49) | 0.28 (0.25 to 0.30) | 780.28 (362.06 to 1605.75) | 881.84 (413.38 to 1820.58) | 0.47 (0.40 to 0.53) |
| Albania | 1253.68 (908.11 to 1651.13) | 1331.96 (994.46 to 1723.64) | 0.19 (0.18 to 0.20) | 936.84 (428.31 to 1950.94) | 1085.73 (505.74 to 2250.99) | 0.60 (0.56 to 0.64) |
| Algeria | 1268.90 (946.50 to 1646.09) | 1363.33 (1025.18 to 1753.07) | 0.23 (0.22 to 0.24) | 945.15 (440.53 to 1946.62) | 1128.98 (544.38 to 2337.59) | 0.61 (0.58 to 0.63) |
| American Samoa | 1302.05 (548.05 to 2456.55) | 1366.62 (729.81 to 2255.63) | 0.12 (0.10 to 0.14) | 1200.54 (265.24 to 3378.11) | 1291.71 (397.43 to 3216.34) | 0.20 (0.14 to 0.25) |
| Andorra | 1473.75 (859.61 to 2276.62) | 1502.15 (979.46 to 2154.40) | -0.07 (-0.13 to -0.01) | 1272.41 (423.48 to 3071.78) | 1376.69 (545.43 to 3071.41) | 0.26 (0.21 to 0.31) |
| Angola | 1315.93 (956.28 to 1734.53) | 1409.89 (1055.92 to 1822.69) | 0.21 (0.19 to 0.22) | 1085.74 (500.68 to 2267.29) | 1205.00 (569.47 to 2490.10) | 0.37 (0.35 to 0.38) |
| Antigua and Barbuda | 1430.47 (821.72 to 2224.26) | 1541.55 (964.41 to 2275.39) | 0.27 (0.25 to 0.28) | 1310.49 (433.48 to 3164.45) | 1359.25 (503.50 to 3117.47) | 0.10 (0.08 to 0.11) |
| Argentina | 1447.57 (1094.20 to 1866.93) | 1533.11 (1159.36 to 1965.30) | 0.06 (0.02 to 0.10) | 1340.48 (637.85 to 2749.96) | 1473.08 (708.91 to 3013.95) | 0.29 (0.25 to 0.33) |
| Armenia | 1202.40 (880.44 to 1591.74) | 1285.46 (951.72 to 1666.30) | 0.14 (0.10 to 0.17) | 1096.67 (500.96 to 2288.49) | 1308.88 (612.77 to 2679.13) | 0.87 (0.76 to 0.98) |
| Australia | 1454.27 (1094.88 to 1867.65) | 1535.40 (1159.11 to 1978.73) | 0.08 (0.04 to 0.13) | 1366.80 (652.59 to 2788.24) | 1511.24 (735.83 to 3079.17) | 0.28 (0.25 to 0.32) |
| Austria | 1449.81 (1098.05 to 1873.56) | 1471.58 (1115.39 to 1889.92) | -0.09 (-0.12 to -0.05) | 1324.32 (635.60 to 2713.50) | 1396.70 (672.79 to 2857.23) | 0.14 (0.12 to 0.16) |
| Azerbaijan | 1224.17 (901.42 to 1596.25) | 1316.56 (984.21 to 1703.34) | 0.17 (0.14 to 0.20) | 1261.05 (584.18 to 2590.08) | 1326.05 (620.86 to 2716.65) | 0.38 (0.24 to 0.52) |
| Bahamas | 1514.84 (969.58 to 2198.18) | 1576.82 (1087.03 to 2176.74) | 0.14 (0.13 to 0.16) | 1355.62 (517.02 to 3085.19) | 1407.52 (601.27 to 3078.08) | 0.12 (0.10 to 0.13) |
| Bahrain | 1357.76 (842.48 to 2017.07) | 1455.53 (1033.98 to 1983.43) | 0.20 (0.18 to 0.21) | 1085.84 (396.93 to 2519.93) | 1145.13 (495.21 to 2450.07) | 0.21 (0.17 to 0.24) |
| Bangladesh | 1181.28 (887.29 to 1517.77) | 1281.57 (970.80 to 1656.18) | 0.29 (0.28 to 0.30) | 870.56 (411.42 to 1772.49) | 998.49 (475.31 to 2033.54) | 0.49 (0.43 to 0.54) |
| Barbados | 1465.86 (978.11 to 2041.75) | 1549.99 (1096.29 to 2098.58) | 0.19 (0.18 to 0.20) | 1355.23 (558.05 to 2956.52) | 1417.02 (613.46 to 3078.72) | 0.15 (0.11 to 0.18) |
| Belarus | 1369.11 (1024.45 to 1768.78) | 1440.52 (1087.01 to 1840.09) | 0.07 (0.04 to 0.10) | 1283.47 (612.58 to 2638.50) | 1434.17 (681.69 to 2925.30) | 0.51 (0.46 to 0.56) |
| Belgium | 1432.10 (1082.87 to 1846.17) | 1468.85 (1106.86 to 1886.39) | -0.13 (-0.19 to -0.06) | 1313.56 (629.95 to 2669.91) | 1384.14 (664.01 to 2839.11) | 0.16 (0.14 to 0.19) |
| Belize | 1430.50 (871.78 to 2139.43) | 1552.17 (1045.02 to 2175.50) | 0.22 (0.19 to 0.25) | 1207.99 (426.43 to 2799.90) | 1350.73 (555.59 to 2991.38) | 0.34 (0.28 to 0.40) |
| Benin | 1239.71 (899.19 to 1634.06) | 1367.57 (1005.57 to 1782.11) | 0.30 (0.29 to 0.32) | 937.34 (421.43 to 1957.97) | 1119.51 (519.03 to 2309.24) | 0.58 (0.56 to 0.61) |
| Bermuda | 1551.27 (906.74 to 2406.16) | 1541.38 (993.18 to 2215.37) | -0.05 (-0.06 to -0.03) | 1419.00 (489.99 to 3383.54) | 1481.76 (587.30 to 3330.90) | 0.14 (0.12 to 0.15) |
| Bhutan | 1238.26 (797.12 to 1776.79) | 1314.46 (909.02 to 1801.56) | 0.19 (0.18 to 0.20) | 880.67 (331.89 to 2004.96) | 1036.73 (443.59 to 2203.75) | 0.57 (0.54 to 0.59) |
| Bolivia (Plurinational State of) | 1458.84 (1074.99 to 1915.15) | 1571.39 (1184.60 to 2022.82) | 0.19 (0.17 to 0.21) | 1132.95 (521.61 to 2367.50) | 1290.84 (610.07 to 2663.58) | 0.44 (0.43 to 0.45) |
| Bosnia and Herzegovina | 1299.83 (970.33 to 1707.06) | 1374.38 (1025.87 to 1784.93) | 0.12 (0.07 to 0.16) | 1025.31 (472.38 to 2132.70) | 1213.73 (568.87 to 2494.28) | 0.70 (0.62 to 0.78) |
| Botswana | 1350.92 (944.94 to 1848.21) | 1470.70 (1068.23 to 1942.95) | 0.22 (0.20 to 0.25) | 1041.71 (438.29 to 2277.54) | 1246.73 (562.42 to 2642.63) | 0.57 (0.55 to 0.60) |
| Brazil | 1559.96 (1181.68 to 1990.28) | 1638.89 (1250.29 to 2099.51) | 0.16 (0.16 to 0.17) | 1216.58 (585.40 to 2496.04) | 1395.54 (677.73 to 2847.18) | 0.47 (0.45 to 0.49) |
| Brunei Darussalam | 1692.91 (1052.98 to 2531.23) | 1896.14 (1309.51 to 2601.09) | 0.32 (0.28 to 0.35) | 1567.30 (567.49 to 3618.52) | 1667.03 (699.21 to 3627.06) | 0.22 (0.19 to 0.24) |
| Bulgaria | 1375.12 (1039.18 to 1773.48) | 1394.88 (1046.31 to 1797.90) | 0.03 (0.01 to 0.05) | 1172.59 (549.80 to 2408.39) | 1327.71 (636.31 to 2726.38) | 0.43 (0.40 to 0.46) |
| Burkina Faso | 1215.47 (897.12 to 1598.91) | 1283.41 (957.01 to 1667.70) | 0.18 (0.18 to 0.19) | 854.38 (392.64 to 1763.35) | 951.49 (446.12 to 1944.32) | 0.34 (0.34 to 0.35) |
| Burundi | 1225.40 (895.99 to 1609.33) | 1259.74 (937.10 to 1639.61) | 0.08 (0.07 to 0.09) | 893.10 (406.42 to 1886.35) | 910.37 (419.77 to 1874.82) | 0.10 (0.09 to 0.12) |
| Cabo Verde | 1219.19 (798.84 to 1731.84) | 1363.69 (928.55 to 1892.69) | 0.28 (0.24 to 0.32) | 980.95 (382.09 to 2168.67) | 1168.57 (493.41 to 2555.32) | 0.68 (0.64 to 0.73) |
| Cambodia | 986.46 (719.50 to 1306.18) | 1101.38 (816.56 to 1431.48) | 0.38 (0.37 to 0.40) | 714.76 (323.99 to 1482.04) | 848.48 (389.67 to 1755.75) | 0.62 (0.60 to 0.65) |
| Cameroon | 1298.79 (963.34 to 1696.40) | 1391.00 (1031.48 to 1801.03) | 0.18 (0.17 to 0.20) | 979.92 (450.55 to 2022.08) | 1115.51 (528.17 to 2321.07) | 0.42 (0.39 to 0.45) |
| Canada | 1095.94 (838.48 to 1402.44) | 1180.66 (895.93 to 1504.72) | 0.02 (-0.12 to 0.16) | 1076.58 (520.65 to 2215.97) | 1171.74 (567.84 to 2386.30) | 0.13 (0.06 to 0.20) |
| Central African Republic | 1283.03 (918.74 to 1729.32) | 1331.14 (964.42 to 1756.86) | 0.11 (0.10 to 0.12) | 905.03 (392.56 to 1940.86) | 950.92 (430.41 to 2002.91) | 0.16 (0.15 to 0.17) |
| Chad | 1193.74 (870.80 to 1563.18) | 1246.75 (931.24 to 1614.04) | 0.14 (0.13 to 0.14) | 865.46 (391.33 to 1786.17) | 909.75 (424.37 to 1886.79) | 0.15 (0.13 to 0.16) |
| Chile | 1450.19 (1090.47 to 1874.21) | 1546.84 (1169.20 to 2005.45) | 0.08 (0.04 to 0.12) | 1301.51 (615.76 to 2682.46) | 1460.05 (700.96 to 2994.13) | 0.36 (0.32 to 0.39) |
| China | 1228.33 (918.19 to 1587.67) | 1373.56 (1032.90 to 1763.32) | 0.24 (0.12 to 0.36) | 989.54 (470.29 to 2004.58) | 1174.05 (560.21 to 2383.22) | 0.66 (0.56 to 0.76) |
| Colombia | 1429.91 (1082.74 to 1847.91) | 1478.98 (1124.34 to 1898.72) | 0.10 (0.09 to 0.12) | 1170.44 (556.91 to 2412.91) | 1336.35 (639.65 to 2749.02) | 0.48 (0.46 to 0.49) |
| Comoros | 1261.06 (808.03 to 1829.80) | 1335.23 (918.78 to 1830.93) | 0.19 (0.18 to 0.19) | 903.13 (338.70 to 2082.26) | 1037.93 (432.56 to 2259.24) | 0.50 (0.48 to 0.53) |
| Congo | 1350.75 (963.74 to 1810.75) | 1418.05 (1035.79 to 1873.96) | 0.13 (0.12 to 0.14) | 1071.21 (466.98 to 2294.00) | 1184.45 (538.05 to 2476.86) | 0.32 (0.30 to 0.34) |
| Cook Islands | 1253.87 (434.25 to 2637.79) | 1349.94 (648.07 to 2346.59) | 0.24 (0.22 to 0.27) | 1148.12 (192.06 to 3478.49) | 1324.80 (359.90 to 3451.04) | 0.45 (0.42 to 0.49) |
| Costa Rica | 1423.30 (1030.81 to 1884.41) | 1498.44 (1111.26 to 1949.66) | 0.14 (0.13 to 0.16) | 1198.05 (541.03 to 2530.02) | 1338.17 (627.00 to 2761.04) | 0.37 (0.35 to 0.39) |
| Côte d'Ivoire | 1279.21 (939.31 to 1676.77) | 1347.75 (1002.51 to 1750.76) | 0.15 (0.14 to 0.17) | 920.38 (417.55 to 1931.93) | 1061.81 (503.52 to 2188.91) | 0.43 (0.42 to 0.45) |
| Croatia | 1368.36 (1023.26 to 1774.67) | 1383.75 (1031.64 to 1790.76) | 0.01 (-0.01 to 0.02) | 1181.58 (555.70 to 2454.00) | 1299.02 (616.75 to 2698.33) | 0.41 (0.38 to 0.45) |
| Cuba | 1434.87 (1076.87 to 1851.91) | 1481.55 (1109.80 to 1912.35) | 0.15 (0.14 to 0.17) | 1206.85 (568.54 to 2506.43) | 1335.85 (637.31 to 2761.39) | 0.35 (0.34 to 0.36) |
| Cyprus | 1474.68 (1060.50 to 1964.17) | 1518.42 (1123.26 to 1984.10) | 0.02 (0.00 to 0.04) | 1202.85 (531.83 to 2557.45) | 1341.99 (619.69 to 2790.68) | 0.33 (0.30 to 0.36) |
| Czechia | 1363.82 (1022.34 to 1758.62) | 1398.72 (1053.17 to 1796.20) | 0.08 (0.06 to 0.10) | 1244.75 (597.42 to 2558.49) | 1338.34 (644.54 to 2759.63) | 0.21 (0.18 to 0.24) |
| Democratic People's Republic of Korea | 1186.67 (868.27 to 1555.20) | 1274.69 (948.43 to 1656.57) | 0.23 (0.21 to 0.25) | 979.67 (451.59 to 1992.82) | 1090.72 (512.74 to 2237.49) | 0.35 (0.30 to 0.40) |
| Democratic Republic of the Congo | 1309.96 (980.01 to 1681.03) | 1336.56 (1014.04 to 1706.36) | 0.01 (-0.02 to 0.05) | 955.75 (447.77 to 1969.16) | 1004.81 (472.01 to 2060.63) | 0.11 (0.05 to 0.17) |
| Denmark | 1411.72 (1059.11 to 1812.07) | 1482.30 (1107.76 to 1903.38) | 0.15 (0.12 to 0.17) | 1419.42 (677.29 to 2942.43) | 1376.34 (655.77 to 2846.23) | -0.07 (-0.10 to -0.05) |
| Djibouti | 1262.34 (764.34 to 1908.21) | 1377.75 (951.22 to 1904.50) | 0.33 (0.31 to 0.35) | 908.53 (304.73 to 2139.84) | 1089.79 (461.22 to 2374.05) | 0.69 (0.64 to 0.74) |
| Dominica | 1459.00 (856.07 to 2249.82) | 1532.37 (934.45 to 2306.29) | 0.15 (0.13 to 0.17) | 1220.22 (409.48 to 2939.74) | 1323.71 (470.38 to 3098.44) | 0.28 (0.22 to 0.34) |
| Dominican Republic | 1440.71 (1062.11 to 1892.25) | 1509.37 (1127.57 to 1951.29) | 0.18 (0.16 to 0.20) | 1209.53 (558.80 to 2543.16) | 1350.87 (636.90 to 2769.34) | 0.38 (0.37 to 0.39) |
| Ecuador | 1494.73 (1112.42 to 1951.75) | 1577.04 (1182.84 to 2044.86) | 0.21 (0.19 to 0.23) | 1260.55 (588.30 to 2598.31) | 1394.94 (662.80 to 2872.26) | 0.37 (0.35 to 0.39) |
| Egypt | 1283.20 (970.13 to 1658.42) | 1397.91 (1051.24 to 1787.72) | 0.22 (0.20 to 0.25) | 938.11 (442.95 to 1929.82) | 1066.38 (512.11 to 2203.37) | 0.32 (0.28 to 0.36) |
| El Salvador | 1414.49 (1037.21 to 1868.10) | 1471.36 (1092.09 to 1933.68) | 0.12 (0.11 to 0.13) | 1171.40 (540.07 to 2433.12) | 1337.10 (627.42 to 2753.39) | 0.45 (0.42 to 0.48) |
| Equatorial Guinea | 1256.83 (801.21 to 1826.41) | 1447.27 (994.76 to 1990.14) | 0.52 (0.48 to 0.56) | 878.86 (321.73 to 2000.89) | 1220.99 (517.54 to 2643.12) | 1.29 (1.21 to 1.37) |
| Eritrea | 1250.72 (886.19 to 1686.22) | 1315.05 (960.13 to 1725.22) | 0.15 (0.14 to 0.17) | 842.78 (348.75 to 1818.52) | 955.05 (428.31 to 1980.85) | 0.42 (0.40 to 0.43) |
| Estonia | 1380.75 (1009.68 to 1816.31) | 1421.30 (1041.05 to 1859.57) | 0.06 (0.04 to 0.08) | 1344.06 (616.34 to 2801.40) | 1514.11 (708.44 to 3139.69) | 0.46 (0.43 to 0.49) |
| Eswatini | 1371.54 (917.79 to 1935.22) | 1498.18 (1053.26 to 2018.94) | 0.27 (0.23 to 0.31) | 1068.68 (416.84 to 2367.67) | 1222.84 (525.12 to 2646.20) | 0.38 (0.34 to 0.42) |
| Ethiopia | 1314.99 (996.19 to 1692.91) | 1443.85 (1092.89 to 1845.24) | 0.33 (0.31 to 0.35) | 908.62 (430.91 to 1868.77) | 1179.68 (567.03 to 2409.24) | 0.97 (0.93 to 1.01) |
| Fiji | 1222.79 (800.96 to 1733.09) | 1355.71 (940.90 to 1860.41) | 0.34 (0.32 to 0.36) | 1043.13 (413.08 to 2305.10) | 1220.32 (536.76 to 2609.47) | 0.50 (0.47 to 0.52) |
| Finland | 1468.59 (1096.79 to 1897.96) | 1496.46 (1128.10 to 1924.47) | -0.01 (-0.05 to 0.03) | 1314.22 (626.32 to 2718.56) | 1392.28 (665.52 to 2852.19) | 0.16 (0.14 to 0.18) |
| France | 1409.12 (1071.46 to 1809.37) | 1462.80 (1118.56 to 1855.07) | -0.05 (-0.12 to 0.01) | 1307.96 (636.92 to 2666.10) | 1388.72 (678.82 to 2829.60) | 0.20 (0.15 to 0.25) |
| Gabon | 1311.83 (910.00 to 1798.05) | 1447.35 (1025.58 to 1941.29) | 0.26 (0.23 to 0.29) | 1031.49 (432.75 to 2240.49) | 1201.45 (530.78 to 2574.82) | 0.51 (0.46 to 0.55) |
| Gambia | 1258.67 (848.41 to 1759.44) | 1354.99 (969.55 to 1813.00) | 0.21 (0.19 to 0.23) | 950.63 (382.82 to 2106.41) | 1127.88 (501.14 to 2415.24) | 0.57 (0.56 to 0.58) |
| Georgia | 1256.83 (938.50 to 1624.43) | 1285.19 (952.16 to 1666.15) | 0.02 (-0.02 to 0.07) | 1194.32 (559.49 to 2471.61) | 1280.34 (603.66 to 2612.90) | 0.28 (0.22 to 0.32) |
| Germany | 1449.88 (1097.69 to 1846.70) | 1460.33 (1111.69 to 1869.53) | -0.14 (-0.21 to -0.08) | 1361.69 (666.21 to 2762.87) | 1410.66 (680.54 to 2859.44) | 0.08 (0.07 to 0.09) |
| Ghana | 1319.39 (980.97 to 1726.21) | 1415.98 (1059.60 to 1809.51) | 0.20 (0.17 to 0.23) | 1122.95 (522.70 to 2341.19) | 1183.84 (559.21 to 2443.97) | 0.24 (0.12 to 0.37) |
| Greece | 1431.82 (1080.94 to 1834.09) | 1423.19 (1073.29 to 1838.58) | -0.13 (-0.22 to -0.05) | 1225.17 (581.27 to 2510.91) | 1366.61 (652.47 to 2811.24) | 0.75 (0.59 to 0.91) |
| Greenland | 1182.68 (550.12 to 2109.81) | 1288.52 (743.28 to 2003.46) | 0.14 (0.08 to 0.19) | 1064.10 (257.40 to 2909.36) | 1155.10 (375.24 to 2799.31) | 0.29 (0.26 to 0.32) |
| Grenada | 1387.55 (825.20 to 2103.95) | 1535.71 (956.54 to 2260.91) | 0.43 (0.38 to 0.49) | 1225.64 (424.03 to 2893.99) | 1319.57 (485.67 to 3036.11) | 0.19 (0.14 to 0.24) |
| Guam | 1276.27 (719.16 to 2016.69) | 1339.35 (871.53 to 1936.95) | 0.15 (0.14 to 0.17) | 1166.29 (376.48 to 2835.45) | 1331.15 (537.53 to 2949.63) | 0.46 (0.42 to 0.50) |
| Guatemala | 1408.10 (1043.88 to 1842.48) | 1456.55 (1090.63 to 1886.62) | 0.07 (0.05 to 0.08) | 1061.21 (483.52 to 2212.33) | 1197.44 (566.69 to 2469.23) | 0.40 (0.39 to 0.42) |
| Guinea | 1217.49 (896.94 to 1597.97) | 1279.44 (955.22 to 1667.60) | 0.10 (0.07 to 0.12) | 883.12 (401.94 to 1838.05) | 968.80 (446.69 to 1998.17) | 0.30 (0.28 to 0.31) |
| Guinea-Bissau | 1244.01 (841.95 to 1733.12) | 1330.66 (928.42 to 1813.35) | 0.20 (0.20 to 0.21) | 890.88 (359.01 to 1946.67) | 988.01 (414.47 to 2147.69) | 0.31 (0.29 to 0.32) |
| Guyana | 1455.01 (994.38 to 2022.64) | 1556.47 (1094.89 to 2110.91) | 0.18 (0.15 to 0.20) | 1150.39 (476.71 to 2533.20) | 1286.01 (557.21 to 2761.37) | 0.38 (0.35 to 0.42) |
| Haiti | 1387.51 (1026.22 to 1822.58) | 1452.91 (1094.74 to 1871.53) | 0.14 (0.12 to 0.15) | 976.11 (446.79 to 2023.14) | 1070.94 (497.80 to 2210.14) | 0.36 (0.34 to 0.38) |
| Honduras | 1402.85 (1027.69 to 1849.79) | 1497.57 (1121.15 to 1937.84) | 0.22 (0.22 to 0.23) | 1105.12 (499.56 to 2289.71) | 1217.09 (575.23 to 2500.69) | 0.32 (0.32 to 0.33) |
| Hungary | 1379.30 (1033.91 to 1776.25) | 1418.90 (1072.15 to 1823.97) | 0.06 (0.04 to 0.07) | 1234.74 (592.06 to 2542.52) | 1360.47 (654.04 to 2781.15) | 0.32 (0.27 to 0.36) |
| Iceland | 1444.60 (987.06 to 2002.12) | 1527.46 (1082.53 to 2059.15) | 0.03 (-0.03 to 0.09) | 1418.60 (596.49 to 3095.20) | 1436.62 (637.32 to 3062.01) | 0.00 (-0.09 to 0.09) |
| India | 1321.28 (1003.55 to 1687.97) | 1436.06 (1092.59 to 1830.80) | 0.26 (0.23 to 0.28) | 934.53 (451.92 to 1897.00) | 1121.57 (543.54 to 2273.38) | 0.60 (0.56 to 0.64) |
| Indonesia | 1105.59 (832.62 to 1421.97) | 1238.30 (935.95 to 1593.00) | 0.38 (0.37 to 0.38) | 836.64 (399.91 to 1712.51) | 997.77 (481.67 to 2032.12) | 0.59 (0.58 to 0.61) |
| Iran (Islamic Republic of) | 1339.52 (1011.36 to 1723.88) | 1418.42 (1075.37 to 1818.03) | 0.14 (0.13 to 0.16) | 963.35 (458.59 to 1981.91) | 1135.92 (546.88 to 2324.11) | 0.60 (0.54 to 0.67) |
| Iraq | 1275.21 (943.14 to 1663.47) | 1369.89 (1022.30 to 1773.67) | 0.21 (0.20 to 0.22) | 1025.48 (478.55 to 2140.87) | 1082.78 (516.29 to 2221.96) | 0.14 (0.11 to 0.16) |
| Ireland | 1459.77 (1096.09 to 1894.10) | 1525.56 (1134.14 to 1976.90) | 0.07 (0.04 to 0.09) | 1286.62 (601.21 to 2654.94) | 1384.31 (657.40 to 2850.07) | 0.19 (0.16 to 0.22) |
| Israel | 1459.28 (1099.13 to 1894.43) | 1526.22 (1159.77 to 1954.72) | 0.03 (-0.03 to 0.08) | 1285.24 (602.11 to 2664.89) | 1384.13 (661.19 to 2847.16) | -0.30 (-0.53 to -0.07) |
| Italy | 1527.79 (1168.15 to 1937.45) | 1522.75 (1156.04 to 1941.18) | -0.10 (-0.12 to -0.07) | 1340.24 (652.50 to 2734.55) | 1445.54 (703.59 to 2955.17) | 0.36 (0.27 to 0.44) |
| Jamaica | 1427.77 (1040.39 to 1884.38) | 1476.63 (1088.18 to 1937.26) | 0.10 (0.08 to 0.12) | 1225.14 (553.32 to 2586.86) | 1331.85 (616.98 to 2762.34) | 0.29 (0.26 to 0.33) |
| Japan | 1820.88 (1394.99 to 2318.43) | 1687.21 (1281.13 to 2158.79) | -0.37 (-0.42 to -0.32) | 1619.34 (780.29 to 3312.25) | 1763.82 (851.02 to 3602.53) | 0.56 (0.32 to 0.79) |
| Jordan | 1303.77 (929.52 to 1756.53) | 1401.85 (1037.41 to 1836.67) | 0.24 (0.22 to 0.27) | 1032.18 (454.63 to 2201.05) | 1171.04 (542.43 to 2417.73) | 0.43 (0.42 to 0.45) |
| Kazakhstan | 1272.42 (943.93 to 1656.75) | 1346.39 (1012.62 to 1740.76) | 0.10 (0.07 to 0.14) | 1273.44 (598.96 to 2616.93) | 1466.66 (705.70 to 2998.71) | 0.57 (0.50 to 0.65) |
| Kenya | 1358.69 (1012.98 to 1760.29) | 1508.54 (1140.87 to 1936.11) | 0.35 (0.33 to 0.37) | 1024.65 (481.02 to 2119.97) | 1207.60 (575.03 to 2489.31) | 0.57 (0.55 to 0.59) |
| Kiribati | 1256.35 (617.82 to 2180.52) | 1355.32 (757.00 to 2165.80) | 0.22 (0.20 to 0.24) | 1074.84 (279.53 to 2841.05) | 1181.65 (371.58 to 2893.37) | 0.25 (0.19 to 0.30) |
| Kuwait | 1311.69 (898.82 to 1820.69) | 1398.63 (1004.32 to 1878.57) | 0.14 (0.11 to 0.18) | 1105.69 (458.83 to 2435.82) | 1196.91 (547.49 to 2493.60) | 0.39 (0.35 to 0.43) |
| Kyrgyzstan | 1220.73 (898.51 to 1610.10) | 1277.12 (948.49 to 1660.77) | 0.06 (0.01 to 0.10) | 1201.05 (552.69 to 2495.97) | 1303.21 (601.04 to 2673.83) | 0.46 (0.36 to 0.57) |
| Lao People's Democratic Republic | 988.68 (703.40 to 1324.33) | 1091.18 (797.79 to 1435.17) | 0.36 (0.34 to 0.38) | 732.41 (320.42 to 1528.30) | 870.68 (396.02 to 1806.02) | 0.62 (0.60 to 0.64) |
| Latvia | 1385.18 (1021.81 to 1804.57) | 1417.11 (1047.84 to 1846.77) | 0.03 (0.01 to 0.05) | 1308.58 (604.01 to 2723.67) | 1485.71 (702.49 to 3096.12) | 0.51 (0.47 to 0.54) |
| Lebanon | 1268.40 (918.82 to 1689.33) | 1330.43 (974.50 to 1733.82) | 0.10 (0.07 to 0.14) | 984.95 (443.17 to 2061.42) | 1180.48 (553.75 to 2426.24) | 0.62 (0.58 to 0.67) |
| Lesotho | 1286.98 (922.12 to 1744.60) | 1444.44 (1041.53 to 1936.30) | 0.41 (0.39 to 0.42) | 988.16 (430.32 to 2113.25) | 1154.03 (517.99 to 2467.58) | 0.54 (0.50 to 0.58) |
| Liberia | 1259.80 (910.81 to 1679.79) | 1359.20 (981.22 to 1798.41) | 0.29 (0.26 to 0.32) | 935.40 (410.33 to 1974.76) | 1073.80 (488.54 to 2255.69) | 0.56 (0.51 to 0.61) |
| Libya | 1283.72 (919.30 to 1704.50) | 1365.86 (1006.63 to 1783.22) | 0.18 (0.17 to 0.20) | 1054.26 (477.78 to 2220.72) | 1152.88 (538.56 to 2390.60) | 0.30 (0.27 to 0.33) |
| Lithuania | 1373.67 (1018.31 to 1782.36) | 1402.48 (1047.09 to 1815.94) | 0.02 (-0.00 to 0.05) | 1282.77 (600.75 to 2669.53) | 1469.08 (692.89 to 3074.16) | 0.54 (0.50 to 0.58) |
| Luxembourg | 1453.30 (1026.04 to 1963.07) | 1496.45 (1079.59 to 1999.53) | -0.11 (-0.17 to -0.04) | 1343.33 (585.29 to 2900.77) | 1396.04 (638.06 to 2912.35) | 0.14 (0.11 to 0.16) |
| Madagascar | 1206.69 (901.53 to 1572.77) | 1275.70 (958.52 to 1649.86) | 0.17 (0.16 to 0.18) | 845.66 (388.27 to 1753.69) | 893.14 (415.45 to 1863.76) | 0.21 (0.20 to 0.22) |
| Malawi | 1256.40 (920.18 to 1636.93) | 1331.79 (991.59 to 1727.47) | 0.23 (0.22 to 0.25) | 878.22 (400.54 to 1821.64) | 993.01 (462.86 to 2056.08) | 0.43 (0.42 to 0.45) |
| Malaysia | 1076.61 (792.21 to 1411.70) | 1183.80 (882.95 to 1539.39) | 0.32 (0.30 to 0.33) | 919.24 (427.79 to 1899.26) | 1060.56 (498.52 to 2172.60) | 0.47 (0.46 to 0.49) |
| Maldives | 1024.30 (562.64 to 1645.15) | 1151.10 (746.42 to 1651.96) | 0.40 (0.39 to 0.42) | 857.19 (260.52 to 2125.54) | 1070.73 (428.46 to 2362.34) | 0.78 (0.75 to 0.81) |
| Mali | 1225.11 (898.18 to 1610.75) | 1297.62 (972.38 to 1684.46) | 0.18 (0.16 to 0.19) | 866.21 (389.43 to 1789.35) | 974.43 (454.80 to 2002.29) | 0.41 (0.40 to 0.43) |
| Malta | 1465.84 (1020.88 to 1984.83) | 1512.50 (1097.49 to 2006.76) | 0.01 (-0.03 to 0.06) | 1295.13 (554.70 to 2781.59) | 1402.47 (634.12 to 2928.27) | 0.20 (0.15 to 0.26) |
| Marshall Islands | 1214.81 (468.53 to 2424.46) | 1309.91 (629.14 to 2298.46) | 0.24 (0.23 to 0.24) | 1062.11 (193.93 to 3146.70) | 1156.31 (292.51 to 3091.52) | 0.21 (0.16 to 0.25) |
| Mauritania | 1290.96 (912.57 to 1731.09) | 1375.91 (1000.15 to 1827.79) | 0.18 (0.15 to 0.20) | 985.70 (429.87 to 2093.20) | 1133.47 (515.13 to 2392.38) | 0.42 (0.41 to 0.44) |
| Mauritius | 1110.57 (759.17 to 1540.79) | 1201.78 (866.59 to 1623.78) | 0.26 (0.26 to 0.27) | 949.99 (407.64 to 2061.99) | 1091.39 (497.32 to 2289.06) | 0.47 (0.44 to 0.51) |
| Mexico | 1562.89 (1186.34 to 1998.18) | 1634.10 (1248.03 to 2082.13) | 0.14 (0.12 to 0.15) | 1269.40 (612.55 to 2599.49) | 1460.64 (709.26 to 2985.51) | 0.51 (0.48 to 0.54) |
| Micronesia (Federated States of) | 1232.51 (658.03 to 2031.79) | 1345.07 (758.40 to 2142.80) | 0.28 (0.27 to 0.30) | 1059.19 (312.18 to 2651.42) | 1197.35 (387.29 to 2896.66) | 0.40 (0.32 to 0.48) |
| Monaco | 1428.23 (867.67 to 2163.86) | 1503.28 (939.07 to 2225.03) | 0.11 (0.06 to 0.16) | 1392.55 (502.14 to 3259.13) | 1450.58 (545.63 to 3314.61) | 0.10 (0.07 to 0.13) |
| Mongolia | 1202.94 (860.65 to 1610.23) | 1280.31 (931.00 to 1701.93) | 0.15 (0.12 to 0.18) | 1091.97 (478.72 to 2308.73) | 1385.64 (625.05 to 2885.02) | 0.84 (0.77 to 0.90) |
| Montenegro | 1359.93 (952.86 to 1856.25) | 1414.70 (1011.82 to 1886.60) | 0.08 (0.06 to 0.10) | 1234.77 (530.32 to 2651.33) | 1289.69 (574.49 to 2746.76) | 0.26 (0.22 to 0.31) |
| Morocco | 1236.17 (929.55 to 1600.78) | 1332.01 (1005.95 to 1707.88) | 0.20 (0.19 to 0.22) | 979.22 (463.43 to 2039.96) | 1061.07 (508.04 to 2174.87) | 0.25 (0.22 to 0.28) |
| Mozambique | 1230.43 (916.36 to 1606.02) | 1320.80 (992.13 to 1708.68) | 0.26 (0.24 to 0.28) | 852.97 (390.07 to 1770.40) | 956.28 (449.02 to 1968.67) | 0.40 (0.39 to 0.41) |
| Myanmar | 1002.27 (745.17 to 1304.25) | 1129.01 (843.68 to 1460.76) | 0.43 (0.42 to 0.45) | 752.46 (355.50 to 1524.47) | 929.08 (444.67 to 1879.00) | 0.81 (0.77 to 0.85) |
| Namibia | 1322.54 (930.20 to 1791.33) | 1404.12 (1019.33 to 1865.35) | 0.17 (0.15 to 0.19) | 978.76 (419.56 to 2142.34) | 1138.84 (508.16 to 2407.64) | 0.45 (0.42 to 0.48) |
| Nauru | 1228.13 (178.27 to 3757.67) | 1369.29 (288.60 to 3619.06) | 0.35 (0.32 to 0.37) | 1064.45 (41.30 to 4565.35) | 1233.86 (93.01 to 4578.05) | 0.45 (0.42 to 0.47) |
| Nepal | 1183.87 (879.48 to 1546.26) | 1300.94 (973.54 to 1686.17) | 0.30 (0.28 to 0.33) | 799.35 (366.17 to 1651.29) | 945.62 (445.11 to 1928.29) | 0.56 (0.54 to 0.58) |
| Netherlands | 1454.72 (1090.62 to 1870.05) | 1536.00 (1163.65 to 1969.91) | 0.08 (0.02 to 0.14) | 1388.06 (671.24 to 2774.06) | 1413.37 (679.76 to 2905.26) | -0.07 (-0.16 to 0.02) |
| New Zealand | 1548.25 (1142.75 to 2020.89) | 1636.96 (1222.92 to 2115.43) | 0.11 (0.08 to 0.14) | 1397.80 (655.82 to 2900.70) | 1534.76 (726.86 to 3176.66) | 0.27 (0.23 to 0.30) |
| Nicaragua | 1398.68 (1008.61 to 1856.76) | 1483.09 (1106.08 to 1925.32) | 0.20 (0.19 to 0.20) | 1094.57 (493.03 to 2299.96) | 1249.30 (586.23 to 2604.66) | 0.43 (0.41 to 0.46) |
| Niger | 1216.80 (887.18 to 1612.10) | 1265.39 (941.77 to 1635.83) | 0.14 (0.13 to 0.15) | 858.47 (383.72 to 1809.11) | 920.67 (431.74 to 1871.06) | 0.22 (0.21 to 0.24) |
| Nigeria | 1362.29 (1027.27 to 1746.08) | 1438.97 (1091.89 to 1840.88) | 0.18 (0.16 to 0.21) | 1029.61 (493.35 to 2100.88) | 1147.14 (553.72 to 2349.70) | 0.40 (0.37 to 0.42) |
| Niue | 1249.61 (107.38 to 4488.49) | 1364.94 (113.66 to 4944.18) | 0.29 (0.27 to 0.31) | 1185.37 (23.54 to 5648.26) | 1312.05 (26.59 to 6240.60) | 0.38 (0.36 to 0.41) |
| North Macedonia | 1325.44 (960.12 to 1747.77) | 1394.06 (1039.58 to 1801.26) | 0.12 (0.09 to 0.14) | 1092.76 (498.19 to 2312.52) | 1202.89 (560.29 to 2518.14) | 0.42 (0.39 to 0.46) |
| Northern Mariana Islands | 1266.82 (422.51 to 2745.76) | 1347.98 (714.91 to 2219.17) | 0.19 (0.17 to 0.21) | 1175.46 (182.68 to 3670.92) | 1258.80 (381.52 to 3123.58) | 0.14 (0.08 to 0.20) |
| Norway | 1496.63 (1120.70 to 1924.43) | 1577.36 (1190.26 to 2035.42) | 0.14 (0.08 to 0.20) | 1364.29 (647.80 to 2817.53) | 1443.36 (689.88 to 2967.87) | 0.23 (0.09 to 0.37) |
| Oman | 1243.51 (854.14 to 1721.07) | 1408.43 (1013.61 to 1877.17) | 0.42 (0.41 to 0.43) | 989.71 (419.54 to 2157.51) | 1183.04 (535.24 to 2497.14) | 0.59 (0.57 to 0.60) |
| Pakistan | 1202.49 (907.20 to 1547.56) | 1357.02 (1023.10 to 1757.54) | 0.43 (0.41 to 0.46) | 870.64 (412.85 to 1775.53) | 1033.83 (496.21 to 2096.96) | 0.57 (0.56 to 0.59) |
| Palau | 1264.30 (399.94 to 2789.81) | 1363.79 (597.73 to 2517.88) | 0.25 (0.24 to 0.26) | 1143.53 (160.00 to 3694.23) | 1257.64 (291.71 to 3454.29) | 0.27 (0.20 to 0.34) |
| Palestine | 1257.96 (891.74 to 1705.07) | 1351.89 (984.70 to 1785.31) | 0.18 (0.16 to 0.21) | 967.71 (421.10 to 2084.66) | 1098.50 (499.42 to 2326.44) | 0.42 (0.40 to 0.43) |
| Panama | 1384.71 (999.64 to 1845.35) | 1464.34 (1082.78 to 1918.12) | 0.19 (0.17 to 0.20) | 1137.66 (507.17 to 2412.43) | 1311.62 (610.38 to 2720.75) | 0.43 (0.41 to 0.44) |
| Papua New Guinea | 1112.73 (803.81 to 1500.90) | 1186.24 (867.51 to 1565.37) | 0.18 (0.16 to 0.19) | 841.83 (372.23 to 1803.44) | 941.26 (430.05 to 1951.17) | 0.32 (0.29 to 0.36) |
| Paraguay | 1449.70 (1055.77 to 1913.19) | 1504.31 (1120.98 to 1967.47) | 0.12 (0.08 to 0.15) | 1237.41 (568.66 to 2603.28) | 1291.81 (602.39 to 2683.20) | 0.14 (0.09 to 0.18) |
| Peru | 1455.10 (1090.79 to 1882.69) | 1528.59 (1155.50 to 1959.18) | 0.11 (0.09 to 0.13) | 1238.70 (585.06 to 2585.20) | 1380.79 (662.35 to 2836.45) | 0.36 (0.33 to 0.38) |
| Philippines | 1089.57 (813.32 to 1412.23) | 1224.92 (923.47 to 1581.49) | 0.37 (0.35 to 0.40) | 863.26 (409.75 to 1765.41) | 1013.12 (485.93 to 2071.37) | 0.48 (0.44 to 0.52) |
| Poland | 1458.73 (1106.80 to 1861.68) | 1548.95 (1184.36 to 1973.87) | 0.14 (0.12 to 0.17) | 1214.72 (584.98 to 2487.65) | 1396.99 (675.66 to 2858.76) | 0.54 (0.49 to 0.59) |
| Portugal | 1457.26 (1103.40 to 1860.51) | 1465.96 (1106.85 to 1892.38) | -0.19 (-0.26 to -0.12) | 1248.14 (593.47 to 2549.98) | 1388.04 (660.92 to 2843.35) | 0.48 (0.36 to 0.60) |
| Puerto Rico | 1527.19 (1132.72 to 2009.16) | 1528.93 (1131.41 to 1985.54) | 0.06 (0.04 to 0.09) | 1382.57 (646.73 to 2871.28) | 1506.56 (713.73 to 3130.33) | 0.29 (0.27 to 0.30) |
| Qatar | 1350.70 (752.91 to 2140.52) | 1463.02 (1011.98 to 2032.65) | 0.20 (0.18 to 0.23) | 1087.65 (344.78 to 2684.20) | 1178.17 (501.95 to 2544.76) | 0.24 (0.21 to 0.28) |
| Republic of Korea | 1872.96 (1417.57 to 2416.82) | 1801.62 (1368.27 to 2307.32) | -0.19 (-0.33 to -0.06) | 1672.16 (809.73 to 3381.21) | 1766.13 (848.92 to 3607.12) | 0.30 (0.17 to 0.43) |
| Republic of Moldova | 1352.64 (1005.65 to 1764.32) | 1430.27 (1063.06 to 1858.63) | 0.17 (0.13 to 0.20) | 1121.60 (522.33 to 2353.12) | 1309.26 (615.11 to 2700.77) | 0.67 (0.59 to 0.76) |
| Romania | 1327.07 (1004.75 to 1697.08) | 1384.81 (1043.52 to 1783.64) | 0.10 (0.08 to 0.12) | 1100.77 (526.55 to 2287.29) | 1240.23 (596.26 to 2550.38) | 0.50 (0.46 to 0.54) |
| Russian Federation | 1488.27 (1137.31 to 1888.75) | 1585.63 (1212.78 to 2014.91) | 0.12 (0.08 to 0.15) | 1470.10 (708.35 to 3026.62) | 1519.16 (737.07 to 3105.08) | 0.34 (0.26 to 0.41) |
| Rwanda | 1238.43 (913.24 to 1623.24) | 1313.24 (983.28 to 1704.15) | 0.20 (0.18 to 0.22) | 850.65 (382.93 to 1776.11) | 946.13 (442.19 to 1957.15) | 0.40 (0.37 to 0.43) |
| Saint Kitts and Nevis | 1494.12 (837.50 to 2401.89) | 1629.44 (968.42 to 2501.74) | 0.25 (0.19 to 0.32) | 1287.39 (404.71 to 3171.77) | 1371.95 (468.65 to 3280.63) | 0.21 (0.16 to 0.27) |
| Saint Lucia | 1461.90 (898.78 to 2181.40) | 1506.89 (1008.88 to 2107.67) | 0.07 (0.06 to 0.08) | 1176.81 (424.14 to 2752.00) | 1338.40 (547.03 to 2970.11) | 0.39 (0.34 to 0.44) |
| Saint Vincent and the Grenadines | 1449.15 (870.53 to 2203.92) | 1511.12 (978.67 to 2179.43) | 0.13 (0.11 to 0.14) | 1187.27 (404.94 to 2815.26) | 1300.62 (503.37 to 2968.54) | 0.33 (0.30 to 0.36) |
| Samoa | 1239.11 (717.85 to 1941.79) | 1325.31 (819.97 to 1963.30) | 0.22 (0.21 to 0.23) | 1077.45 (359.84 to 2557.15) | 1221.25 (461.50 to 2804.85) | 0.45 (0.43 to 0.48) |
| San Marino | 1442.16 (776.82 to 2366.43) | 1448.28 (875.03 to 2191.88) | -0.11 (-0.15 to -0.07) | 1338.63 (408.30 to 3343.93) | 1411.17 (508.85 to 3292.15) | 0.15 (0.12 to 0.18) |
| Sao Tome and Principe | 1280.23 (731.63 to 2014.27) | 1399.59 (848.89 to 2109.91) | 0.24 (0.23 to 0.26) | 1023.73 (325.73 to 2518.08) | 1226.10 (436.71 to 2868.30) | 0.66 (0.63 to 0.69) |
| Saudi Arabia | 1263.95 (929.23 to 1661.26) | 1428.05 (1062.78 to 1873.14) | 0.37 (0.35 to 0.40) | 995.08 (458.43 to 2059.78) | 1146.45 (550.67 to 2352.49) | 0.22 (0.11 to 0.33) |
| Senegal | 1263.17 (925.79 to 1654.78) | 1336.11 (989.33 to 1735.53) | 0.15 (0.14 to 0.16) | 951.88 (431.44 to 1968.53) | 1065.41 (494.41 to 2210.84) | 0.35 (0.33 to 0.36) |
| Serbia | 1350.67 (1019.26 to 1745.48) | 1393.16 (1045.92 to 1790.41) | 0.07 (0.05 to 0.08) | 1080.52 (509.82 to 2235.50) | 1244.70 (592.58 to 2546.17) | 0.58 (0.54 to 0.61) |
| Seychelles | 1112.26 (602.77 to 1798.83) | 1200.53 (719.08 to 1821.05) | 0.24 (0.22 to 0.26) | 974.41 (301.66 to 2393.55) | 1104.59 (400.79 to 2572.93) | 0.42 (0.36 to 0.48) |
| Sierra Leone | 1214.49 (881.94 to 1604.91) | 1307.50 (964.13 to 1710.53) | 0.25 (0.23 to 0.27) | 897.20 (405.10 to 1861.21) | 1026.63 (468.33 to 2127.51) | 0.44 (0.40 to 0.47) |
| Singapore | 1753.58 (1295.26 to 2291.67) | 1800.20 (1349.50 to 2312.02) | -0.02 (-0.06 to 0.02) | 1668.20 (761.68 to 3468.88) | 1737.65 (823.89 to 3574.93) | 0.14 (0.11 to 0.18) |
| Slovakia | 1364.00 (1016.66 to 1769.04) | 1419.43 (1072.19 to 1823.59) | 0.10 (0.07 to 0.12) | 1225.38 (576.08 to 2537.17) | 1338.60 (636.99 to 2766.28) | 0.29 (0.24 to 0.33) |
| Slovenia | 1357.63 (990.37 to 1777.61) | 1385.12 (1030.95 to 1810.33) | 0.01 (-0.01 to 0.03) | 1242.28 (574.79 to 2585.84) | 1335.58 (628.69 to 2771.76) | 0.26 (0.23 to 0.29) |
| Solomon Islands | 1140.13 (684.51 to 1732.30) | 1263.02 (830.34 to 1796.00) | 0.33 (0.31 to 0.35) | 919.45 (316.40 to 2180.87) | 1085.53 (433.23 to 2399.90) | 0.54 (0.51 to 0.56) |
| Somalia | 1238.23 (889.36 to 1643.66) | 1309.92 (977.93 to 1698.07) | 0.18 (0.16 to 0.19) | 874.45 (386.30 to 1852.71) | 900.38 (411.96 to 1858.27) | 0.15 (0.13 to 0.17) |
| South Africa | 1502.79 (1134.18 to 1931.67) | 1594.29 (1212.26 to 2043.38) | 0.19 (0.18 to 0.19) | 1290.07 (616.12 to 2656.85) | 1377.96 (666.62 to 2812.99) | 0.27 (0.24 to 0.29) |
| South Sudan | 1209.25 (886.49 to 1592.66) | 1277.05 (938.03 to 1674.42) | 0.21 (0.18 to 0.25) | 840.00 (379.88 to 1738.09) | 901.08 (411.94 to 1857.56) | 0.25 (0.24 to 0.26) |
| Spain | 1457.25 (1117.28 to 1850.74) | 1452.63 (1105.17 to 1851.57) | -0.17 (-0.22 to -0.12) | 1295.92 (622.35 to 2662.64) | 1415.58 (685.28 to 2885.45) | 0.24 (0.19 to 0.29) |
| Sri Lanka | 1029.68 (760.05 to 1337.92) | 1147.76 (855.37 to 1500.39) | 0.37 (0.36 to 0.38) | 819.11 (380.85 to 1684.65) | 965.82 (459.84 to 1983.79) | 0.54 (0.53 to 0.55) |
| Sudan | 1167.32 (863.09 to 1524.05) | 1295.74 (967.79 to 1667.03) | 0.35 (0.32 to 0.39) | 814.69 (376.08 to 1681.77) | 1000.79 (468.12 to 2059.73) | 0.68 (0.64 to 0.71) |
| Suriname | 1464.68 (978.27 to 2067.84) | 1540.71 (1090.18 to 2095.64) | 0.14 (0.12 to 0.15) | 1278.40 (510.05 to 2831.36) | 1382.99 (606.23 to 2950.48) | 0.32 (0.30 to 0.34) |
| Sweden | 1366.68 (1033.02 to 1742.86) | 1427.41 (1074.96 to 1836.30) | 0.17 (0.04 to 0.30) | 1161.70 (547.99 to 2442.29) | 1258.82 (597.48 to 2591.16) | 0.34 (0.12 to 0.57) |
| Switzerland | 1402.00 (1054.31 to 1804.06) | 1443.82 (1087.07 to 1850.65) | 0.00 (-0.02 to 0.03) | 1314.62 (627.95 to 2721.35) | 1360.12 (656.90 to 2788.25) | 0.09 (0.08 to 0.10) |
| Syrian Arab Republic | 1244.57 (919.78 to 1632.44) | 1358.78 (1015.58 to 1770.82) | 0.25 (0.22 to 0.27) | 937.69 (433.36 to 1957.39) | 1083.14 (504.66 to 2208.07) | 0.55 (0.52 to 0.58) |
| Taiwan (Province of China) | 1189.15 (868.47 to 1565.25) | 1315.69 (974.13 to 1728.43) | 0.38 (0.36 to 0.40) | 1070.18 (504.97 to 2209.67) | 1283.09 (608.58 to 2625.40) | 0.68 (0.64 to 0.73) |
| Tajikistan | 1170.74 (855.07 to 1544.96) | 1211.90 (909.47 to 1581.74) | 0.05 (0.01 to 0.09) | 1052.84 (479.01 to 2188.25) | 1096.37 (508.96 to 2257.69) | 0.24 (0.15 to 0.33) |
| Thailand | 1042.19 (775.06 to 1356.30) | 1174.33 (869.40 to 1526.00) | 0.41 (0.40 to 0.42) | 818.04 (388.61 to 1668.72) | 1024.82 (492.58 to 2082.61) | 0.80 (0.78 to 0.82) |
| Timor-Leste | 970.61 (611.95 to 1417.35) | 1086.38 (760.45 to 1482.67) | 0.41 (0.39 to 0.44) | 721.94 (265.79 to 1664.34) | 873.04 (373.63 to 1871.14) | 0.75 (0.69 to 0.80) |
| Togo | 1245.41 (889.86 to 1668.54) | 1359.12 (1005.26 to 1790.86) | 0.30 (0.29 to 0.31) | 921.58 (401.67 to 1955.79) | 1062.31 (487.99 to 2200.17) | 0.45 (0.42 to 0.48) |
| Tokelau | 1202.73 (26.53 to 5970.30) | 1321.16 (58.61 to 5651.98) | 0.31 (0.30 to 0.33) | 1050.95 (1.24 to 6897.20) | 1251.25 (8.36 to 6949.77) | 0.66 (0.61 to 0.71) |
| Tonga | 1217.62 (653.40 to 1981.57) | 1308.66 (767.20 to 2036.71) | 0.21 (0.18 to 0.24) | 1057.03 (317.08 to 2634.19) | 1208.49 (413.75 to 2864.45) | 0.39 (0.34 to 0.44) |
| Trinidad and Tobago | 1498.26 (1063.29 to 2008.40) | 1563.81 (1140.01 to 2056.87) | 0.20 (0.17 to 0.22) | 1281.59 (566.39 to 2690.54) | 1375.47 (635.89 to 2897.08) | 0.25 (0.22 to 0.28) |
| Tunisia | 1257.80 (925.23 to 1654.91) | 1352.56 (1016.93 to 1744.08) | 0.21 (0.20 to 0.22) | 963.76 (446.01 to 2014.57) | 1125.87 (537.09 to 2317.95) | 0.54 (0.51 to 0.56) |
| Turkey | 1280.02 (955.74 to 1642.48) | 1393.28 (1053.06 to 1781.07) | 0.22 (0.18 to 0.27) | 974.71 (460.13 to 2000.05) | 1159.74 (562.64 to 2384.10) | 0.62 (0.54 to 0.70) |
| Turkmenistan | 1239.00 (900.24 to 1634.68) | 1316.74 (970.90 to 1725.68) | 0.14 (0.11 to 0.17) | 1145.29 (514.44 to 2379.12) | 1341.74 (612.52 to 2729.78) | 0.66 (0.57 to 0.75) |
| Tuvalu | 1229.69 (303.31 to 3055.81) | 1334.41 (455.41 to 2844.78) | 0.27 (0.26 to 0.28) | 1018.06 (86.60 to 3712.75) | 1211.53 (192.07 to 3781.62) | 0.57 (0.51 to 0.62) |
| Uganda | 1219.03 (904.84 to 1584.79) | 1306.89 (985.86 to 1681.29) | 0.24 (0.23 to 0.25) | 865.56 (399.58 to 1780.49) | 976.28 (459.87 to 1994.48) | 0.41 (0.40 to 0.42) |
| Ukraine | 1503.33 (1140.09 to 1927.31) | 1541.76 (1178.93 to 1979.13) | 0.04 (0.02 to 0.06) | 1318.75 (634.94 to 2707.51) | 1438.33 (691.13 to 2901.02) | 0.40 (0.35 to 0.45) |
| United Arab Emirates | 1305.25 (857.71 to 1845.37) | 1445.89 (1058.82 to 1902.52) | 0.35 (0.34 to 0.36) | 1045.73 (412.58 to 2320.52) | 1059.77 (483.31 to 2188.63) | 0.06 (-0.00 to 0.13) |
| United Kingdom | 1578.09 (1209.21 to 2005.36) | 1620.29 (1238.94 to 2063.15) | -0.02 (-0.05 to 0.01) | 1432.32 (694.28 to 2935.04) | 1519.69 (739.09 to 3086.41) | 0.04 (-0.11 to 0.19) |
| United Republic of Tanzania | 1277.04 (954.68 to 1657.03) | 1330.20 (1011.30 to 1704.14) | 0.10 (0.08 to 0.12) | 950.87 (446.20 to 1964.49) | 1030.66 (487.68 to 2115.15) | 0.16 (0.12 to 0.21) |
| United States of America | 1510.14 (1140.72 to 1937.55) | 1619.01 (1229.37 to 2075.88) | 0.02 (-0.12 to 0.16) | 1570.42 (767.31 to 3201.15) | 1637.64 (805.42 to 3317.74) | -0.08 (-0.23 to 0.07) |
| United States Virgin Islands | 1557.27 (938.86 to 2362.35) | 1577.14 (1045.78 to 2216.53) | 0.12 (0.07 to 0.16) | 1347.57 (473.09 to 3167.07) | 1458.95 (591.18 to 3221.35) | 0.22 (0.20 to 0.24) |
| Uruguay | 1420.92 (1060.27 to 1843.03) | 1489.62 (1103.63 to 1938.68) | -0.00 (-0.04 to 0.04) | 1340.90 (622.98 to 2779.07) | 1480.25 (698.90 to 3020.46) | 0.33 (0.30 to 0.35) |
| Uzbekistan | 1210.74 (901.73 to 1561.52) | 1299.15 (984.41 to 1664.07) | 0.15 (0.12 to 0.18) | 1183.91 (561.44 to 2435.82) | 1290.33 (608.70 to 2600.17) | 0.35 (0.28 to 0.42) |
| Vanuatu | 1124.30 (603.63 to 1833.47) | 1234.76 (769.64 to 1813.44) | 0.30 (0.30 to 0.31) | 874.57 (248.90 to 2207.91) | 995.23 (369.11 to 2295.99) | 0.41 (0.40 to 0.42) |
| Venezuela (Bolivarian Republic of) | 1447.66 (1080.88 to 1870.25) | 1527.50 (1147.91 to 1961.06) | 0.12 (0.08 to 0.17) | 1233.15 (577.37 to 2533.95) | 1333.34 (634.37 to 2717.01) | 0.12 (0.05 to 0.19) |
| Viet Nam | 995.22 (745.03 to 1290.93) | 1101.22 (826.85 to 1423.54) | 0.36 (0.35 to 0.38) | 752.48 (358.44 to 1527.28) | 890.26 (424.74 to 1812.07) | 0.66 (0.62 to 0.69) |
| Yemen | 1166.48 (864.62 to 1515.96) | 1256.53 (946.13 to 1617.59) | 0.28 (0.26 to 0.30) | 786.83 (359.55 to 1633.01) | 933.93 (434.25 to 1906.79) | 0.65 (0.61 to 0.69) |
| Zambia | 1271.22 (926.40 to 1677.56) | 1346.73 (1000.60 to 1746.61) | 0.20 (0.17 to 0.22) | 961.37 (431.77 to 2030.46) | 1030.79 (479.14 to 2135.50) | 0.29 (0.23 to 0.35) |
| Zimbabwe | 1298.60 (966.09 to 1702.36) | 1361.37 (1019.18 to 1752.48) | 0.11 (0.07 to 0.14) | 960.20 (440.29 to 2011.64) | 989.37 (464.31 to 2028.03) | -0.01 (-0.06 to 0.04) |

**Abbreviations: DALY=disability-adjusted life-years. EAPC=estimated annual percentage change. CI=confidence interval**

**Table S3.** **Age-standardised incidence rates and DALY rates in 1990 and 2021, and estimated annual percentage changes for hand osteoarthritis in older adults, by country.**

| **Countries** | **Age-standardised incidence rate, 1990** | **Age-standardised incidence rate, 2021** | **EAPC of incidence rate No. (95% CI)** | **Age-standardised DALYs rate, 1990** | **Age-standardised DALYs rate, 2021** | **EAPC of DALYs rate No. (95% CI)** |
| --- | --- | --- | --- | --- | --- | --- |
| Afghanistan | 139.58 (61.26 to 262.52) | 162.87 (74.34 to 300.18) | 0.46 (0.38 to 0.54) | 150.84 (61.24 to 323.97) | 187.27 (78.03 to 403.62) | 0.67 (0.55 to 0.80) |
| Albania | 197.78 (83.18 to 380.09) | 221.79 (100.78 to 412.03) | 0.42 (0.39 to 0.46) | 261.43 (102.38 to 574.83) | 348.07 (149.05 to 741.97) | 1.18 (1.09 to 1.26) |
| Algeria | 184.91 (82.52 to 349.15) | 210.27 (96.68 to 384.01) | 0.39 (0.35 to 0.44) | 245.23 (104.34 to 517.57) | 335.94 (149.48 to 697.88) | 1.03 (0.95 to 1.11) |
| American Samoa | 258.43 (14.82 to 936.20) | 270.35 (42.94 to 761.39) | 0.13 (0.11 to 0.15) | 381.85 (32.43 to 1373.09) | 416.95 (77.95 to 1209.24) | 0.28 (0.27 to 0.30) |
| Andorra | 186.03 (26.92 to 531.07) | 193.78 (48.77 to 468.69) | 0.15 (0.11 to 0.19) | 353.51 (71.05 to 1001.50) | 372.69 (108.97 to 927.24) | 0.20 (0.15 to 0.24) |
| Angola | 247.44 (105.34 to 469.46) | 271.68 (123.40 to 497.24) | 0.26 (0.23 to 0.30) | 416.00 (173.44 to 899.70) | 469.74 (204.50 to 997.08) | 0.42 (0.40 to 0.44) |
| Antigua and Barbuda | 240.75 (42.81 to 654.76) | 242.70 (58.28 to 593.26) | 0.05 (0.03 to 0.08) | 435.99 (96.43 to 1192.26) | 444.83 (121.19 to 1143.22) | 0.02 (-0.00 to 0.05) |
| Argentina | 236.23 (111.16 to 422.47) | 241.57 (113.40 to 440.70) | 0.03 (0.01 to 0.05) | 431.52 (190.46 to 885.05) | 448.11 (201.42 to 925.24) | 0.10 (0.08 to 0.12) |
| Armenia | 229.64 (96.89 to 435.42) | 246.23 (107.92 to 461.34) | 0.08 (-0.03 to 0.19) | 474.23 (193.43 to 1025.48) | 635.07 (271.64 to 1334.24) | 1.33 (1.16 to 1.50) |
| Australia | 231.94 (107.69 to 422.88) | 232.75 (109.22 to 418.83) | 0.05 (0.03 to 0.07) | 411.36 (181.90 to 861.87) | 429.32 (194.26 to 886.11) | 0.11 (0.09 to 0.14) |
| Austria | 195.71 (87.01 to 363.63) | 194.19 (87.41 to 361.22) | -0.06 (-0.08 to -0.03) | 372.30 (161.56 to 783.95) | 380.59 (167.13 to 797.33) | 0.04 (0.02 to 0.06) |
| Azerbaijan | 243.20 (103.11 to 459.85) | 252.01 (111.79 to 457.36) | -0.07 (-0.19 to 0.04) | 621.56 (268.10 to 1312.64) | 649.51 (282.84 to 1365.75) | 0.40 (0.18 to 0.62) |
| Bahamas | 242.38 (62.74 to 575.49) | 250.38 (85.78 to 529.35) | 0.08 (0.06 to 0.10) | 435.49 (128.76 to 1077.52) | 452.52 (161.39 to 1045.24) | 0.11 (0.09 to 0.13) |
| Bahrain | 210.08 (49.03 to 516.31) | 216.69 (79.96 to 440.57) | 0.11 (0.06 to 0.15) | 338.43 (88.14 to 884.76) | 342.24 (126.94 to 769.68) | 0.09 (0.01 to 0.16) |
| Bangladesh | 181.08 (85.83 to 336.51) | 212.04 (100.89 to 381.21) | 0.52 (0.46 to 0.59) | 224.66 (100.40 to 468.74) | 298.29 (133.20 to 617.86) | 0.95 (0.81 to 1.09) |
| Barbados | 236.04 (77.63 to 504.26) | 244.04 (87.70 to 495.74) | 0.11 (0.08 to 0.13) | 425.06 (146.96 to 995.30) | 453.94 (170.94 to 1022.56) | 0.21 (0.16 to 0.26) |
| Belarus | 254.59 (116.47 to 459.99) | 260.75 (119.76 to 472.44) | 0.04 (-0.01 to 0.09) | 537.80 (241.32 to 1104.00) | 636.27 (285.99 to 1318.70) | 0.73 (0.66 to 0.79) |
| Belgium | 195.52 (86.72 to 362.90) | 195.79 (88.18 to 364.29) | -0.04 (-0.08 to -0.00) | 380.42 (165.72 to 802.89) | 388.03 (173.28 to 819.63) | 0.08 (0.06 to 0.09) |
| Belize | 213.22 (45.41 to 546.17) | 229.89 (73.11 to 503.39) | 0.19 (0.16 to 0.23) | 321.86 (74.37 to 862.66) | 388.28 (128.39 to 929.58) | 0.54 (0.49 to 0.59) |
| Benin | 182.85 (75.85 to 355.28) | 224.11 (98.72 to 420.50) | 0.69 (0.67 to 0.71) | 213.28 (82.42 to 473.13) | 310.37 (129.32 to 662.24) | 1.24 (1.22 to 1.26) |
| Bermuda | 244.74 (44.63 to 652.18) | 246.44 (65.79 to 572.45) | 0.00 (-0.02 to 0.02) | 443.57 (100.45 to 1210.99) | 469.41 (144.66 to 1152.18) | 0.18 (0.17 to 0.20) |
| Bhutan | 179.04 (47.57 to 422.67) | 203.93 (73.29 to 420.03) | 0.47 (0.44 to 0.49) | 204.58 (51.81 to 531.60) | 269.24 (94.66 to 621.56) | 0.88 (0.86 to 0.91) |
| Bolivia (Plurinational State of) | 212.20 (90.69 to 405.51) | 232.68 (107.44 to 421.43) | 0.26 (0.21 to 0.31) | 312.32 (129.21 to 672.58) | 374.15 (163.51 to 788.03) | 0.59 (0.50 to 0.68) |
| Bosnia and Herzegovina | 235.76 (103.79 to 435.58) | 255.61 (114.32 to 477.30) | 0.21 (0.15 to 0.26) | 350.09 (147.46 to 757.57) | 475.73 (206.55 to 1007.45) | 1.22 (1.07 to 1.38) |
| Botswana | 224.46 (82.64 to 462.41) | 267.47 (111.66 to 516.47) | 0.47 (0.42 to 0.52) | 308.75 (105.76 to 723.11) | 417.85 (167.19 to 927.24) | 0.91 (0.82 to 1.00) |
| Brazil | 297.68 (146.22 to 527.77) | 330.59 (161.90 to 577.95) | 0.33 (0.30 to 0.35) | 375.15 (169.73 to 784.06) | 461.91 (212.25 to 955.58) | 0.68 (0.65 to 0.70) |
| Brunei Darussalam | 243.62 (52.53 to 611.46) | 266.51 (89.50 to 568.49) | 0.26 (0.24 to 0.29) | 468.42 (121.64 to 1213.80) | 490.42 (170.03 to 1145.09) | 0.15 (0.12 to 0.17) |
| Bulgaria | 253.64 (116.02 to 460.97) | 263.04 (121.83 to 472.70) | 0.14 (0.11 to 0.16) | 450.43 (198.06 to 951.41) | 555.15 (247.90 to 1163.52) | 0.74 (0.68 to 0.80) |
| Burkina Faso | 152.03 (66.11 to 287.43) | 164.94 (75.07 to 302.07) | 0.28 (0.26 to 0.31) | 154.89 (61.09 to 336.10) | 178.43 (73.94 to 382.10) | 0.45 (0.42 to 0.48) |
| Burundi | 177.63 (73.25 to 345.09) | 184.31 (79.86 to 346.10) | 0.15 (0.13 to 0.17) | 205.70 (79.81 to 451.34) | 216.15 (87.28 to 462.20) | 0.27 (0.21 to 0.32) |
| Cabo Verde | 187.69 (53.63 to 428.50) | 241.44 (82.98 to 503.44) | 0.86 (0.83 to 0.89) | 221.30 (61.69 to 555.65) | 341.18 (117.97 to 802.47) | 1.66 (1.57 to 1.75) |
| Cambodia | 158.43 (69.51 to 293.83) | 210.33 (97.17 to 378.26) | 1.00 (0.96 to 1.03) | 155.04 (61.34 to 339.89) | 242.79 (103.89 to 518.84) | 1.60 (1.53 to 1.66) |
| Cameroon | 176.74 (76.44 to 336.91) | 210.30 (95.28 to 386.79) | 0.57 (0.52 to 0.62) | 197.79 (78.95 to 427.11) | 275.65 (117.78 to 590.17) | 1.07 (0.99 to 1.16) |
| Canada | 229.24 (105.01 to 417.53) | 230.26 (106.93 to 419.03) | 0.03 (-0.00 to 0.05) | 465.32 (207.93 to 983.95) | 471.90 (212.89 to 977.26) | 0.04 (0.02 to 0.06) |
| Central African Republic | 199.98 (79.14 to 396.62) | 213.26 (88.16 to 414.02) | 0.16 (0.15 to 0.18) | 243.57 (87.42 to 557.51) | 261.03 (100.06 to 580.68) | 0.16 (0.12 to 0.21) |
| Chad | 164.01 (69.55 to 312.74) | 169.32 (75.55 to 316.44) | 0.08 (0.05 to 0.11) | 176.79 (68.90 to 392.40) | 189.71 (76.84 to 411.57) | 0.20 (0.15 to 0.26) |
| Chile | 223.44 (101.27 to 408.25) | 233.89 (107.86 to 422.33) | 0.11 (0.09 to 0.13) | 364.82 (157.18 to 770.20) | 413.59 (184.76 to 865.68) | 0.39 (0.37 to 0.41) |
| China | 203.25 (98.87 to 360.36) | 282.15 (141.17 to 495.25) | 0.83 (0.65 to 1.02) | 180.31 (79.90 to 376.28) | 293.28 (132.88 to 610.03) | 1.55 (1.36 to 1.75) |
| Colombia | 209.77 (95.93 to 385.98) | 230.75 (111.16 to 421.27) | 0.37 (0.35 to 0.38) | 337.53 (145.93 to 709.38) | 419.92 (190.75 to 878.90) | 0.83 (0.78 to 0.88) |
| Comoros | 177.23 (47.16 to 422.14) | 217.75 (76.07 to 454.60) | 0.74 (0.70 to 0.78) | 201.32 (48.52 to 532.11) | 287.20 (96.64 to 682.98) | 1.29 (1.21 to 1.37) |
| Congo | 237.27 (92.37 to 477.66) | 255.75 (108.03 to 489.49) | 0.16 (0.13 to 0.19) | 351.77 (134.12 to 804.61) | 409.29 (164.53 to 897.60) | 0.44 (0.38 to 0.50) |
| Cook Islands | 245.72 (4.69 to 1092.53) | 265.31 (27.72 to 837.37) | 0.23 (0.19 to 0.27) | 359.94 (13.43 to 1517.97) | 428.45 (60.26 to 1345.32) | 0.57 (0.55 to 0.59) |
| Costa Rica | 206.15 (83.89 to 401.10) | 230.40 (103.63 to 426.96) | 0.34 (0.33 to 0.36) | 334.40 (133.56 to 728.75) | 406.30 (175.18 to 866.55) | 0.65 (0.62 to 0.68) |
| Côte d'Ivoire | 180.02 (77.09 to 345.90) | 208.91 (94.21 to 382.20) | 0.51 (0.46 to 0.55) | 203.48 (81.47 to 442.49) | 274.85 (117.10 to 586.78) | 0.95 (0.90 to 1.00) |
| Croatia | 271.47 (123.45 to 498.55) | 259.17 (117.75 to 469.70) | -0.14 (-0.19 to -0.10) | 476.71 (206.37 to 1008.74) | 526.65 (233.87 to 1104.89) | 0.45 (0.41 to 0.49) |
| Cuba | 215.58 (98.93 to 398.57) | 231.08 (106.05 to 421.10) | 0.22 (0.21 to 0.23) | 338.62 (145.89 to 711.98) | 394.45 (175.90 to 820.66) | 0.48 (0.45 to 0.50) |
| Cyprus | 180.96 (67.77 to 360.45) | 191.26 (78.07 to 372.69) | 0.15 (0.11 to 0.20) | 325.44 (121.92 to 735.07) | 370.78 (153.31 to 803.09) | 0.38 (0.31 to 0.46) |
| Czechia | 269.20 (121.71 to 492.32) | 259.17 (120.77 to 471.88) | -0.07 (-0.13 to -0.02) | 530.95 (238.24 to 1122.90) | 562.80 (253.94 to 1185.38) | 0.16 (0.11 to 0.20) |
| Democratic People's Republic of Korea | 181.45 (82.19 to 332.44) | 216.67 (101.12 to 388.79) | 0.56 (0.54 to 0.58) | 189.89 (80.49 to 408.87) | 256.66 (111.88 to 539.05) | 0.97 (0.88 to 1.07) |
| Democratic Republic of the Congo | 209.00 (97.69 to 381.93) | 223.76 (107.06 to 404.49) | 0.17 (0.09 to 0.26) | 267.49 (114.37 to 566.37) | 293.29 (129.03 to 621.20) | 0.17 (0.00 to 0.34) |
| Denmark | 192.93 (83.88 to 358.93) | 189.69 (83.45 to 354.26) | -0.06 (-0.14 to 0.02) | 470.25 (204.25 to 994.51) | 378.69 (164.38 to 793.79) | -0.47 (-0.58 to -0.37) |
| Djibouti | 189.64 (38.16 to 498.63) | 228.70 (82.52 to 475.52) | 0.78 (0.71 to 0.84) | 226.90 (44.65 to 644.77) | 332.27 (115.47 to 775.25) | 1.50 (1.37 to 1.64) |
| Dominica | 220.90 (40.29 to 592.77) | 234.52 (51.18 to 592.32) | 0.20 (0.12 to 0.27) | 334.53 (68.41 to 936.92) | 392.67 (96.50 to 1029.91) | 0.57 (0.47 to 0.68) |
| Dominican Republic | 222.45 (96.33 to 417.78) | 235.20 (108.53 to 431.52) | 0.17 (0.15 to 0.20) | 367.52 (153.94 to 797.53) | 418.29 (183.80 to 885.97) | 0.39 (0.38 to 0.41) |
| Ecuador | 223.30 (97.89 to 423.30) | 234.63 (108.10 to 430.74) | 0.16 (0.14 to 0.17) | 360.84 (152.08 to 769.49) | 408.62 (178.69 to 861.73) | 0.43 (0.40 to 0.46) |
| Egypt | 174.44 (81.07 to 318.40) | 194.81 (92.68 to 357.59) | 0.24 (0.20 to 0.28) | 218.09 (94.51 to 455.41) | 272.18 (120.28 to 576.45) | 0.54 (0.48 to 0.60) |
| El Salvador | 207.89 (88.80 to 401.85) | 230.52 (101.97 to 434.34) | 0.36 (0.34 to 0.37) | 328.89 (135.23 to 708.01) | 404.90 (175.91 to 851.58) | 0.75 (0.72 to 0.77) |
| Equatorial Guinea | 182.93 (47.11 to 435.13) | 258.26 (90.14 to 542.64) | 1.29 (1.20 to 1.39) | 206.19 (48.99 to 554.86) | 410.35 (147.71 to 952.28) | 2.67 (2.50 to 2.84) |
| Eritrea | 178.64 (66.83 to 361.93) | 213.74 (94.38 to 402.67) | 0.58 (0.54 to 0.61) | 188.86 (63.24 to 440.20) | 257.87 (101.35 to 571.00) | 1.03 (0.99 to 1.08) |
| Estonia | 256.64 (107.69 to 491.15) | 253.50 (107.85 to 474.16) | -0.07 (-0.11 to -0.04) | 584.57 (246.19 to 1240.26) | 679.34 (296.40 to 1438.13) | 0.52 (0.46 to 0.57) |
| Eswatini | 210.77 (64.87 to 473.40) | 258.59 (96.19 to 516.87) | 0.69 (0.65 to 0.72) | 268.30 (78.58 to 663.03) | 376.89 (134.63 to 861.30) | 1.10 (1.07 to 1.12) |
| Ethiopia | 230.65 (111.08 to 412.51) | 335.63 (164.34 to 595.42) | 1.37 (1.31 to 1.43) | 233.66 (100.80 to 494.83) | 441.84 (199.17 to 928.61) | 2.41 (2.31 to 2.51) |
| Fiji | 230.86 (73.43 to 502.22) | 269.25 (105.33 to 526.97) | 0.52 (0.48 to 0.57) | 304.81 (94.48 to 734.77) | 392.44 (146.16 to 895.38) | 0.87 (0.84 to 0.90) |
| Finland | 193.89 (85.11 to 367.88) | 192.76 (86.49 to 359.30) | 0.03 (0.00 to 0.06) | 358.85 (155.28 to 750.19) | 376.73 (165.26 to 791.05) | 0.13 (0.11 to 0.16) |
| France | 194.65 (88.74 to 352.07) | 195.54 (89.89 to 355.19) | 0.02 (-0.02 to 0.06) | 375.38 (168.98 to 776.71) | 386.14 (175.99 to 800.03) | 0.10 (0.09 to 0.12) |
| Gabon | 214.79 (77.31 to 441.29) | 247.02 (97.74 to 478.98) | 0.37 (0.31 to 0.42) | 291.72 (101.40 to 676.30) | 376.79 (143.87 to 842.60) | 0.84 (0.77 to 0.92) |
| Gambia | 186.14 (58.91 to 414.97) | 231.16 (89.96 to 457.76) | 0.70 (0.69 to 0.72) | 225.61 (66.29 to 554.39) | 336.27 (127.22 to 764.91) | 1.37 (1.33 to 1.40) |
| Georgia | 239.38 (106.80 to 443.85) | 245.23 (111.33 to 450.91) | 0.04 (-0.13 to 0.20) | 527.48 (227.71 to 1103.71) | 594.46 (260.78 to 1235.54) | 0.37 (0.23 to 0.51) |
| Germany | 197.15 (91.75 to 357.73) | 193.93 (90.14 to 354.17) | -0.22 (-0.28 to -0.16) | 388.01 (174.50 to 797.68) | 387.63 (174.73 to 794.81) | -0.16 (-0.22 to -0.10) |
| Ghana | 241.94 (105.45 to 456.11) | 243.50 (111.96 to 447.58) | 0.07 (-0.13 to 0.27) | 400.30 (169.26 to 855.08) | 363.64 (160.35 to 757.17) | -0.12 (-0.55 to 0.31) |
| Greece | 173.40 (78.01 to 322.14) | 185.85 (82.97 to 342.61) | 0.82 (0.59 to 1.06) | 295.20 (128.52 to 617.01) | 354.69 (156.15 to 737.20) | 2.09 (1.51 to 2.68) |
| Greenland | 224.84 (16.51 to 771.51) | 232.50 (41.48 to 615.61) | -0.02 (-0.08 to 0.03) | 419.92 (51.77 to 1367.79) | 441.74 (99.35 to 1222.50) | 0.18 (0.16 to 0.20) |
| Grenada | 227.54 (45.45 to 585.32) | 235.47 (56.71 to 574.79) | 0.15 (0.04 to 0.25) | 364.84 (85.01 to 983.40) | 410.47 (109.39 to 1052.26) | 0.29 (0.19 to 0.39) |
| Guam | 257.50 (49.43 to 677.78) | 276.08 (88.10 to 602.72) | 0.23 (0.21 to 0.26) | 378.07 (79.32 to 1062.73) | 445.30 (145.47 to 1060.41) | 0.55 (0.51 to 0.60) |
| Guatemala | 192.07 (80.60 to 363.31) | 210.72 (98.19 to 382.94) | 0.29 (0.27 to 0.31) | 278.43 (113.98 to 605.24) | 334.66 (144.54 to 710.01) | 0.60 (0.58 to 0.61) |
| Guinea | 161.13 (69.42 to 306.30) | 180.91 (78.28 to 339.00) | 0.39 (0.38 to 0.40) | 171.34 (67.10 to 379.53) | 211.13 (86.85 to 453.93) | 0.69 (0.68 to 0.71) |
| Guinea-Bissau | 169.97 (52.88 to 375.69) | 200.03 (72.92 to 416.17) | 0.54 (0.53 to 0.55) | 187.08 (53.62 to 464.28) | 241.04 (81.48 to 558.88) | 0.80 (0.76 to 0.83) |
| Guyana | 219.03 (71.92 to 466.35) | 239.67 (89.35 to 483.64) | 0.30 (0.27 to 0.32) | 322.82 (107.18 to 762.51) | 394.25 (145.07 to 902.83) | 0.67 (0.63 to 0.72) |
| Haiti | 175.82 (76.79 to 330.40) | 201.82 (91.31 to 373.89) | 0.48 (0.46 to 0.50) | 213.00 (85.38 to 461.05) | 275.79 (116.93 to 585.98) | 0.91 (0.89 to 0.93) |
| Honduras | 195.48 (78.98 to 375.81) | 213.82 (96.69 to 398.18) | 0.27 (0.24 to 0.31) | 294.03 (115.12 to 644.91) | 338.87 (144.69 to 717.66) | 0.48 (0.43 to 0.53) |
| Hungary | 265.29 (121.93 to 488.44) | 269.75 (124.88 to 497.41) | 0.04 (0.03 to 0.06) | 503.47 (224.25 to 1051.88) | 573.97 (256.10 to 1189.05) | 0.40 (0.31 to 0.48) |
| Iceland | 184.29 (55.54 to 407.57) | 189.42 (65.91 to 399.23) | 0.00 (-0.32 to 0.32) | 432.66 (147.32 to 1016.21) | 393.55 (146.57 to 893.08) | -0.38 (-0.65 to -0.12) |
| India | 238.16 (119.20 to 416.82) | 288.55 (144.01 to 498.68) | 0.66 (0.63 to 0.69) | 252.02 (115.09 to 520.41) | 360.15 (166.06 to 742.20) | 1.22 (1.17 to 1.26) |
| Indonesia | 255.60 (124.58 to 452.18) | 318.68 (159.87 to 552.32) | 0.65 (0.61 to 0.69) | 251.75 (112.64 to 527.27) | 358.58 (161.89 to 746.58) | 1.09 (1.05 to 1.13) |
| Iran (Islamic Republic of) | 243.50 (117.13 to 439.53) | 285.46 (139.38 to 507.99) | 0.62 (0.56 to 0.68) | 275.95 (121.42 to 583.31) | 367.05 (165.97 to 761.54) | 1.13 (0.87 to 1.38) |
| Iraq | 193.81 (85.60 to 365.65) | 207.57 (96.38 to 377.31) | 0.12 (0.08 to 0.16) | 269.13 (114.81 to 570.35) | 304.47 (134.22 to 635.33) | 0.20 (0.13 to 0.27) |
| Ireland | 185.31 (79.98 to 348.54) | 193.12 (84.65 to 365.44) | 0.11 (0.09 to 0.14) | 345.59 (144.16 to 735.81) | 372.51 (160.29 to 780.19) | 0.19 (0.14 to 0.24) |
| Israel | 188.86 (83.00 to 363.30) | 195.83 (88.10 to 362.22) | 0.32 (0.22 to 0.42) | 364.83 (153.33 to 777.80) | 385.42 (165.76 to 813.58) | -1.35 (-1.97 to -0.73) |
| Italy | 265.23 (127.52 to 472.91) | 272.72 (130.53 to 484.36) | 0.31 (0.15 to 0.47) | 407.64 (184.28 to 845.72) | 445.11 (203.43 to 918.10) | 0.66 (0.37 to 0.94) |
| Jamaica | 221.22 (91.88 to 428.11) | 233.58 (102.19 to 442.10) | 0.18 (0.17 to 0.20) | 351.34 (140.18 to 763.39) | 398.49 (167.07 to 857.94) | 0.40 (0.36 to 0.45) |
| Japan | 360.01 (179.15 to 627.84) | 337.77 (165.82 to 598.37) | -0.45 (-0.59 to -0.31) | 478.29 (217.48 to 992.44) | 585.08 (268.41 to 1197.93) | 1.31 (0.68 to 1.95) |
| Jordan | 204.71 (79.99 to 414.84) | 215.02 (95.68 to 400.30) | 0.16 (0.15 to 0.18) | 303.22 (114.57 to 674.75) | 352.62 (150.69 to 744.91) | 0.49 (0.47 to 0.51) |
| Kazakhstan | 249.58 (110.12 to 464.32) | 259.20 (119.23 to 473.00) | -0.04 (-0.15 to 0.06) | 609.73 (268.56 to 1288.45) | 773.41 (345.14 to 1609.24) | 0.91 (0.80 to 1.03) |
| Kenya | 270.01 (124.76 to 489.95) | 337.78 (163.58 to 599.08) | 0.77 (0.74 to 0.79) | 304.00 (130.19 to 649.88) | 433.66 (193.45 to 914.72) | 1.21 (1.18 to 1.23) |
| Kiribati | 250.89 (28.36 to 770.62) | 271.43 (50.28 to 719.42) | 0.18 (0.15 to 0.21) | 344.55 (44.35 to 1122.71) | 381.18 (77.27 to 1099.26) | 0.22 (0.17 to 0.28) |
| Kuwait | 199.45 (63.75 to 436.10) | 208.18 (84.94 to 400.60) | 0.14 (0.07 to 0.21) | 331.34 (112.02 to 785.68) | 347.07 (141.06 to 740.59) | 0.31 (0.25 to 0.38) |
| Kyrgyzstan | 244.85 (103.21 to 464.59) | 254.90 (111.35 to 474.90) | 0.01 (-0.13 to 0.15) | 575.41 (244.09 to 1218.98) | 661.74 (284.51 to 1416.13) | 0.76 (0.56 to 0.95) |
| Lao People's Democratic Republic | 167.42 (69.02 to 324.51) | 211.32 (91.99 to 395.39) | 0.81 (0.76 to 0.87) | 171.41 (63.25 to 390.32) | 253.08 (103.24 to 550.67) | 1.30 (1.24 to 1.37) |
| Latvia | 255.74 (111.05 to 477.56) | 251.84 (110.16 to 473.36) | -0.07 (-0.09 to -0.05) | 545.11 (233.75 to 1159.80) | 655.85 (289.52 to 1374.13) | 0.71 (0.65 to 0.76) |
| Lebanon | 197.97 (80.33 to 386.39) | 220.11 (96.74 to 411.30) | 0.35 (0.32 to 0.37) | 277.51 (109.92 to 616.03) | 371.07 (159.51 to 783.15) | 1.02 (0.98 to 1.05) |
| Lesotho | 205.94 (80.43 to 410.80) | 258.63 (105.68 to 504.57) | 0.81 (0.72 to 0.90) | 258.27 (93.87 to 586.40) | 372.62 (142.95 to 829.17) | 1.35 (1.21 to 1.49) |
| Liberia | 175.06 (67.64 to 355.03) | 211.50 (88.57 to 409.39) | 0.71 (0.67 to 0.76) | 204.50 (74.35 to 460.37) | 276.17 (108.34 to 612.44) | 1.17 (1.10 to 1.25) |
| Libya | 198.94 (79.98 to 396.99) | 214.64 (92.73 to 408.21) | 0.20 (0.17 to 0.22) | 298.03 (118.19 to 663.01) | 348.00 (144.87 to 745.51) | 0.47 (0.38 to 0.55) |
| Lithuania | 250.30 (110.79 to 466.88) | 251.29 (111.27 to 468.87) | 0.00 (-0.01 to 0.02) | 525.15 (225.22 to 1117.09) | 648.22 (286.14 to 1358.29) | 0.82 (0.77 to 0.88) |
| Luxembourg | 192.55 (66.68 to 403.61) | 197.21 (74.77 to 394.18) | -0.03 (-0.06 to 0.01) | 380.63 (138.09 to 859.10) | 386.97 (152.59 to 855.51) | 0.05 (0.02 to 0.07) |
| Madagascar | 157.71 (69.55 to 293.42) | 173.52 (79.88 to 314.00) | 0.33 (0.32 to 0.34) | 167.88 (67.36 to 368.52) | 190.93 (78.43 to 410.98) | 0.43 (0.41 to 0.44) |
| Malawi | 172.65 (75.58 to 325.21) | 202.26 (92.52 to 373.77) | 0.59 (0.57 to 0.62) | 188.17 (74.85 to 414.56) | 245.17 (103.27 to 530.00) | 0.91 (0.88 to 0.94) |
| Malaysia | 229.16 (100.82 to 429.75) | 260.23 (121.13 to 464.31) | 0.34 (0.30 to 0.39) | 307.64 (131.72 to 648.75) | 387.92 (172.73 to 814.44) | 0.69 (0.63 to 0.75) |
| Maldives | 228.82 (41.57 to 608.24) | 262.39 (84.78 to 564.85) | 0.29 (0.23 to 0.36) | 309.62 (60.81 to 895.07) | 416.56 (137.40 to 994.69) | 0.87 (0.79 to 0.95) |
| Mali | 164.86 (71.13 to 311.59) | 191.46 (86.21 to 354.22) | 0.52 (0.49 to 0.55) | 177.98 (69.72 to 392.66) | 233.19 (97.61 to 507.13) | 0.93 (0.88 to 0.98) |
| Malta | 193.36 (65.04 to 412.15) | 193.76 (72.93 to 389.32) | 0.03 (-0.01 to 0.07) | 374.17 (130.88 to 851.88) | 388.45 (151.40 to 852.53) | 0.08 (0.04 to 0.12) |
| Marshall Islands | 251.28 (9.25 to 999.45) | 281.74 (31.88 to 877.97) | 0.41 (0.40 to 0.43) | 367.36 (21.94 to 1430.90) | 415.13 (55.86 to 1340.26) | 0.35 (0.29 to 0.40) |
| Mauritania | 183.99 (71.43 to 375.68) | 213.34 (90.01 to 407.77) | 0.47 (0.41 to 0.53) | 215.93 (77.40 to 491.75) | 290.44 (116.17 to 637.99) | 0.96 (0.86 to 1.05) |
| Mauritius | 244.58 (90.62 to 482.57) | 272.33 (116.59 to 519.89) | 0.37 (0.36 to 0.39) | 336.39 (124.63 to 770.15) | 417.00 (171.93 to 913.00) | 0.71 (0.66 to 0.75) |
| Mexico | 281.73 (134.48 to 501.76) | 310.81 (151.04 to 547.34) | 0.34 (0.30 to 0.37) | 384.61 (173.02 to 804.10) | 508.30 (232.82 to 1050.03) | 1.08 (0.99 to 1.18) |
| Micronesia (Federated States of) | 236.04 (35.03 to 676.62) | 274.62 (52.37 to 726.50) | 0.54 (0.51 to 0.56) | 313.26 (50.43 to 955.50) | 391.60 (80.35 to 1106.13) | 0.79 (0.70 to 0.88) |
| Monaco | 192.26 (34.54 to 513.75) | 193.30 (40.91 to 491.12) | 0.05 (0.01 to 0.10) | 385.43 (92.18 to 1033.37) | 388.71 (103.59 to 988.47) | 0.01 (-0.01 to 0.04) |
| Mongolia | 227.07 (89.09 to 453.67) | 255.55 (103.47 to 489.63) | 0.34 (0.32 to 0.36) | 483.21 (190.05 to 1065.65) | 746.08 (310.66 to 1623.17) | 1.59 (1.49 to 1.70) |
| Montenegro | 257.40 (94.29 to 527.97) | 264.28 (106.02 to 508.37) | 0.00 (-0.04 to 0.04) | 493.94 (187.13 to 1127.49) | 532.57 (214.26 to 1171.96) | 0.39 (0.34 to 0.44) |
| Morocco | 196.66 (88.95 to 366.09) | 201.54 (96.61 to 362.58) | -0.01 (-0.05 to 0.04) | 285.14 (123.07 to 605.58) | 295.96 (130.81 to 620.92) | -0.02 (-0.14 to 0.10) |
| Mozambique | 167.83 (74.86 to 313.12) | 198.05 (92.37 to 360.83) | 0.59 (0.56 to 0.61) | 179.27 (72.93 to 386.30) | 233.53 (98.97 to 500.40) | 0.88 (0.84 to 0.91) |
| Myanmar | 180.98 (83.95 to 328.37) | 240.18 (114.74 to 431.27) | 1.03 (0.98 to 1.08) | 193.51 (83.79 to 412.90) | 310.21 (138.80 to 642.95) | 1.76 (1.68 to 1.84) |
| Namibia | 204.12 (74.21 to 419.86) | 247.64 (101.96 to 477.08) | 0.56 (0.52 to 0.60) | 253.90 (87.04 to 582.67) | 353.13 (137.19 to 777.75) | 0.97 (0.88 to 1.05) |
| Nauru | 240.77 (0.01 to 2000.24) | 274.92 (0.21 to 1777.09) | 0.48 (0.43 to 0.54) | 331.72 (0.09 to 2415.37) | 406.74 (1.49 to 2321.22) | 0.70 (0.65 to 0.76) |
| Nepal | 157.16 (70.74 to 289.38) | 190.57 (89.59 to 346.69) | 0.59 (0.56 to 0.63) | 164.75 (68.23 to 349.28) | 223.96 (97.04 to 467.71) | 0.94 (0.89 to 0.99) |
| Netherlands | 171.26 (78.89 to 312.89) | 192.56 (87.85 to 355.29) | 0.34 (0.14 to 0.54) | 399.81 (183.74 to 819.05) | 379.00 (169.06 to 795.01) | -0.54 (-0.92 to -0.17) |
| New Zealand | 315.09 (143.22 to 576.77) | 323.99 (151.38 to 586.43) | 0.11 (0.08 to 0.13) | 444.61 (189.90 to 949.88) | 476.71 (212.34 to 999.75) | 0.18 (0.15 to 0.22) |
| Nicaragua | 191.27 (76.04 to 374.90) | 217.03 (96.56 to 407.48) | 0.41 (0.40 to 0.41) | 271.43 (104.37 to 600.57) | 349.66 (147.70 to 746.30) | 0.84 (0.82 to 0.86) |
| Niger | 159.07 (66.77 to 307.48) | 170.06 (76.18 to 316.97) | 0.29 (0.25 to 0.34) | 168.26 (64.20 to 373.56) | 189.17 (77.81 to 403.55) | 0.41 (0.37 to 0.46) |
| Nigeria | 262.56 (128.10 to 467.91) | 294.37 (144.91 to 520.00) | 0.53 (0.40 to 0.67) | 281.61 (125.35 to 588.38) | 346.11 (156.15 to 721.37) | 0.90 (0.72 to 1.08) |
| Niue | 248.60 (0.00 to 2637.22) | 274.44 (0.00 to 2911.01) | 0.27 (0.25 to 0.29) | 379.48 (0.01 to 3216.62) | 428.87 (0.02 to 3588.01) | 0.48 (0.45 to 0.52) |
| North Macedonia | 239.66 (99.91 to 462.81) | 253.73 (112.22 to 477.02) | 0.14 (0.11 to 0.16) | 393.52 (158.68 to 861.00) | 467.76 (197.70 to 988.07) | 0.72 (0.66 to 0.78) |
| Northern Mariana Islands | 254.55 (4.02 to 1167.17) | 270.71 (42.26 to 766.02) | 0.28 (0.21 to 0.34) | 385.48 (13.11 to 1662.15) | 413.16 (75.88 to 1216.63) | 0.14 (0.10 to 0.18) |
| Norway | 271.12 (124.03 to 495.64) | 274.63 (127.58 to 505.29) | 0.12 (0.08 to 0.17) | 441.85 (193.34 to 929.79) | 452.88 (200.20 to 947.07) | 0.29 (-0.08 to 0.66) |
| Oman | 202.61 (70.13 to 428.31) | 220.77 (89.12 to 426.16) | 0.25 (0.23 to 0.28) | 314.28 (110.51 to 726.17) | 368.29 (146.26 to 816.87) | 0.45 (0.42 to 0.48) |
| Pakistan | 244.71 (118.12 to 436.78) | 292.46 (143.61 to 513.90) | 0.54 (0.53 to 0.56) | 270.27 (121.30 to 564.50) | 359.77 (163.08 to 746.62) | 0.86 (0.81 to 0.90) |
| Palau | 246.20 (2.45 to 1203.89) | 272.27 (19.68 to 941.99) | 0.38 (0.34 to 0.43) | 360.99 (8.80 to 1645.21) | 410.10 (39.63 to 1426.86) | 0.41 (0.34 to 0.47) |
| Palestine | 188.61 (69.65 to 384.17) | 214.73 (89.45 to 414.56) | 0.42 (0.38 to 0.46) | 249.43 (90.15 to 570.09) | 339.64 (137.08 to 743.49) | 1.04 (1.00 to 1.08) |
| Panama | 205.84 (82.38 to 403.26) | 224.55 (98.23 to 421.36) | 0.29 (0.28 to 0.31) | 334.18 (130.72 to 740.43) | 403.95 (172.29 to 849.91) | 0.58 (0.56 to 0.60) |
| Papua New Guinea | 174.72 (72.04 to 335.94) | 206.39 (91.92 to 376.19) | 0.57 (0.54 to 0.61) | 183.16 (66.76 to 417.63) | 241.77 (98.86 to 528.74) | 0.89 (0.87 to 0.91) |
| Paraguay | 232.25 (94.24 to 449.48) | 232.51 (103.65 to 428.93) | 0.01 (-0.07 to 0.09) | 388.47 (158.98 to 846.53) | 386.20 (164.77 to 832.80) | -0.05 (-0.23 to 0.14) |
| Peru | 230.44 (104.90 to 421.63) | 238.09 (110.28 to 433.77) | 0.00 (-0.08 to 0.09) | 390.98 (169.62 to 824.62) | 432.98 (193.18 to 900.71) | 0.23 (0.11 to 0.35) |
| Philippines | 287.06 (139.14 to 509.12) | 335.17 (166.95 to 584.93) | 0.41 (0.37 to 0.45) | 316.87 (140.44 to 667.71) | 398.80 (180.55 to 832.90) | 0.60 (0.53 to 0.67) |
| Poland | 351.82 (171.12 to 623.11) | 371.28 (183.40 to 647.91) | 0.17 (0.14 to 0.19) | 495.63 (224.35 to 1028.57) | 604.04 (275.87 to 1250.87) | 0.76 (0.68 to 0.83) |
| Portugal | 184.62 (84.75 to 344.90) | 192.17 (87.27 to 356.41) | -0.03 (-0.12 to 0.05) | 331.22 (143.84 to 697.46) | 371.81 (165.68 to 765.98) | 0.98 (0.59 to 1.38) |
| Puerto Rico | 236.12 (101.38 to 449.15) | 243.31 (108.29 to 455.94) | 0.12 (0.09 to 0.15) | 423.49 (177.91 to 915.53) | 464.92 (203.46 to 985.16) | 0.28 (0.27 to 0.29) |
| Qatar | 197.18 (27.87 to 567.74) | 208.79 (71.50 to 432.42) | 0.13 (0.06 to 0.19) | 316.10 (58.01 to 925.98) | 335.17 (115.14 to 775.87) | 0.16 (0.10 to 0.22) |
| Republic of Korea | 248.65 (117.09 to 449.50) | 253.19 (120.90 to 455.01) | -0.04 (-0.19 to 0.10) | 421.18 (186.72 to 872.82) | 489.89 (220.78 to 1026.93) | 0.46 (0.29 to 0.64) |
| Republic of Moldova | 230.95 (102.73 to 423.43) | 248.36 (113.08 to 463.46) | 0.31 (0.24 to 0.38) | 409.15 (173.20 to 876.06) | 511.34 (223.06 to 1072.90) | 0.99 (0.84 to 1.14) |
| Romania | 243.85 (112.83 to 441.17) | 252.93 (119.48 to 457.90) | 0.11 (0.07 to 0.16) | 399.53 (177.49 to 844.06) | 461.85 (205.24 to 971.90) | 0.62 (0.56 to 0.69) |
| Russian Federation | 354.47 (174.40 to 619.76) | 369.96 (184.13 to 645.70) | 0.03 (-0.04 to 0.10) | 727.05 (333.91 to 1504.04) | 710.76 (328.37 to 1469.62) | 0.27 (0.15 to 0.39) |
| Rwanda | 158.59 (66.29 to 304.58) | 186.54 (85.08 to 344.34) | 0.59 (0.56 to 0.61) | 162.78 (61.87 to 360.66) | 207.87 (84.68 to 448.32) | 0.88 (0.83 to 0.93) |
| Saint Kitts and Nevis | 230.27 (33.39 to 658.30) | 252.80 (47.36 to 665.14) | 0.31 (0.28 to 0.34) | 395.59 (73.77 to 1146.25) | 430.66 (98.02 to 1193.75) | 0.30 (0.23 to 0.36) |
| Saint Lucia | 216.50 (47.88 to 545.74) | 233.51 (70.45 to 515.91) | 0.22 (0.19 to 0.25) | 321.27 (75.37 to 861.75) | 405.57 (131.22 to 983.20) | 0.69 (0.60 to 0.77) |
| Saint Vincent and the Grenadines | 221.79 (44.42 to 577.55) | 227.98 (60.71 to 531.11) | 0.07 (0.05 to 0.09) | 340.17 (74.36 to 937.54) | 385.51 (111.54 to 968.29) | 0.40 (0.36 to 0.45) |
| Samoa | 232.08 (46.97 to 594.38) | 260.81 (71.06 to 601.26) | 0.48 (0.42 to 0.53) | 300.63 (62.26 to 840.37) | 391.67 (108.59 to 994.61) | 1.07 (0.98 to 1.16) |
| San Marino | 189.03 (18.76 to 599.19) | 191.52 (33.94 to 519.56) | 0.04 (0.02 to 0.05) | 371.93 (60.82 to 1120.52) | 383.08 (91.97 to 1013.09) | 0.08 (0.05 to 0.12) |
| Sao Tome and Principe | 209.08 (34.40 to 579.65) | 250.31 (55.54 to 625.60) | 0.63 (0.61 to 0.65) | 271.37 (48.73 to 794.79) | 408.91 (104.20 to 1082.75) | 1.50 (1.40 to 1.59) |
| Saudi Arabia | 190.01 (81.43 to 366.73) | 204.76 (94.03 to 381.68) | 0.37 (0.26 to 0.48) | 278.14 (117.20 to 590.89) | 330.70 (144.44 to 693.15) | -0.25 (-0.57 to 0.07) |
| Senegal | 182.70 (78.33 to 343.95) | 212.79 (94.40 to 391.02) | 0.51 (0.49 to 0.52) | 216.30 (86.74 to 468.22) | 282.77 (120.76 to 597.58) | 0.86 (0.83 to 0.89) |
| Serbia | 242.31 (113.31 to 439.10) | 253.67 (118.25 to 460.71) | 0.16 (0.14 to 0.19) | 381.54 (165.68 to 806.92) | 475.03 (211.77 to 993.05) | 0.88 (0.83 to 0.94) |
| Seychelles | 233.14 (38.68 to 640.56) | 271.47 (67.59 to 639.96) | 0.44 (0.39 to 0.49) | 319.45 (60.89 to 916.80) | 422.49 (115.60 to 1086.29) | 0.88 (0.74 to 1.02) |
| Sierra Leone | 167.54 (67.56 to 329.06) | 204.30 (87.55 to 395.04) | 0.73 (0.67 to 0.78) | 185.75 (71.04 to 408.43) | 264.45 (108.70 to 575.78) | 1.21 (1.12 to 1.31) |
| Singapore | 256.12 (105.51 to 491.59) | 260.49 (118.63 to 476.44) | 0.03 (-0.00 to 0.06) | 515.08 (211.27 to 1110.45) | 515.97 (226.12 to 1076.30) | 0.02 (-0.01 to 0.05) |
| Slovakia | 265.20 (120.11 to 493.48) | 270.41 (123.93 to 495.54) | 0.08 (0.06 to 0.10) | 507.59 (220.43 to 1076.38) | 568.65 (253.32 to 1183.80) | 0.39 (0.31 to 0.46) |
| Slovenia | 268.94 (115.28 to 513.36) | 259.76 (114.17 to 471.89) | -0.21 (-0.24 to -0.17) | 521.75 (221.17 to 1117.56) | 553.68 (241.32 to 1171.33) | 0.18 (0.13 to 0.23) |
| Solomon Islands | 205.23 (46.99 to 511.16) | 245.48 (81.12 to 530.33) | 0.59 (0.56 to 0.61) | 247.23 (54.95 to 686.90) | 333.11 (105.52 to 810.55) | 1.04 (0.98 to 1.09) |
| Somalia | 176.44 (72.17 to 345.04) | 189.92 (86.93 to 344.39) | 0.27 (0.26 to 0.29) | 193.51 (70.85 to 433.85) | 209.33 (84.54 to 452.00) | 0.35 (0.29 to 0.40) |
| South Africa | 350.40 (169.85 to 618.76) | 376.27 (185.54 to 657.95) | 0.22 (0.19 to 0.25) | 477.33 (212.99 to 998.94) | 520.69 (236.32 to 1082.28) | 0.35 (0.29 to 0.40) |
| South Sudan | 150.50 (62.87 to 294.11) | 168.88 (74.17 to 315.76) | 0.47 (0.43 to 0.51) | 160.81 (61.26 to 353.49) | 183.69 (71.53 to 400.19) | 0.52 (0.49 to 0.56) |
| Spain | 186.37 (85.88 to 342.76) | 194.56 (89.59 to 353.89) | 0.18 (0.14 to 0.21) | 340.31 (149.64 to 711.97) | 382.85 (172.39 to 794.73) | 0.39 (0.33 to 0.45) |
| Sri Lanka | 202.95 (92.48 to 373.63) | 247.54 (118.16 to 440.82) | 0.66 (0.64 to 0.68) | 242.93 (104.92 to 516.08) | 337.21 (148.54 to 710.65) | 1.01 (0.97 to 1.05) |
| Sudan | 147.15 (65.82 to 274.49) | 182.06 (84.29 to 343.33) | 0.72 (0.69 to 0.75) | 168.31 (70.15 to 356.36) | 251.24 (108.75 to 530.51) | 1.35 (1.25 to 1.44) |
| Suriname | 238.34 (72.40 to 525.87) | 245.65 (90.50 to 499.56) | 0.10 (0.09 to 0.12) | 417.05 (135.29 to 1007.41) | 445.87 (168.44 to 1005.84) | 0.29 (0.26 to 0.32) |
| Sweden | 259.79 (120.41 to 468.63) | 262.58 (122.66 to 477.73) | 0.49 (-0.14 to 1.13) | 373.62 (160.51 to 791.69) | 384.16 (167.42 to 810.67) | 0.71 (-0.36 to 1.80) |
| Switzerland | 193.10 (85.83 to 362.43) | 193.69 (86.62 to 362.94) | -0.02 (-0.04 to -0.00) | 379.93 (165.81 to 792.71) | 381.36 (167.91 to 794.98) | -0.01 (-0.03 to 0.01) |
| Syrian Arab Republic | 176.86 (75.74 to 340.83) | 205.23 (93.83 to 376.99) | 0.60 (0.55 to 0.65) | 225.72 (92.07 to 485.02) | 312.02 (134.78 to 650.51) | 1.31 (1.20 to 1.42) |
| Taiwan (Province of China) | 227.67 (101.86 to 412.85) | 261.84 (122.28 to 477.70) | 0.44 (0.40 to 0.48) | 294.61 (127.37 to 632.39) | 387.13 (173.55 to 816.68) | 0.91 (0.84 to 0.98) |
| Tajikistan | 228.66 (97.29 to 434.61) | 234.31 (102.79 to 437.31) | -0.02 (-0.15 to 0.11) | 454.74 (187.66 to 977.26) | 499.38 (211.75 to 1063.42) | 0.43 (0.27 to 0.59) |
| Thailand | 188.60 (87.57 to 342.79) | 233.46 (111.64 to 421.99) | 0.68 (0.65 to 0.70) | 208.86 (91.21 to 438.57) | 308.01 (138.50 to 633.79) | 1.30 (1.26 to 1.34) |
| Timor-Leste | 171.53 (46.08 to 400.05) | 229.37 (90.14 to 453.25) | 1.15 (1.05 to 1.25) | 175.16 (41.98 to 467.05) | 295.67 (108.82 to 665.91) | 1.99 (1.83 to 2.16) |
| Togo | 180.66 (71.07 to 359.72) | 221.96 (97.10 to 415.22) | 0.76 (0.71 to 0.81) | 207.51 (74.86 to 475.16) | 291.12 (118.05 to 637.53) | 1.13 (1.03 to 1.23) |
| Tokelau | 226.28 (0.00 to 3999.31) | 262.09 (0.00 to 3584.75) | 0.48 (0.43 to 0.53) | 309.39 (0.00 to 4440.87) | 409.71 (0.00 to 4268.85) | 1.09 (1.02 to 1.17) |
| Tonga | 224.36 (35.08 to 637.07) | 249.87 (53.62 to 631.42) | 0.34 (0.33 to 0.36) | 290.33 (45.83 to 867.44) | 358.95 (80.86 to 989.50) | 0.69 (0.68 to 0.71) |
| Trinidad and Tobago | 231.02 (87.81 to 456.61) | 241.98 (102.43 to 459.50) | 0.15 (0.13 to 0.18) | 393.23 (151.78 to 885.03) | 428.95 (178.01 to 933.26) | 0.27 (0.23 to 0.31) |
| Tunisia | 187.06 (80.53 to 357.26) | 216.71 (97.96 to 400.20) | 0.46 (0.42 to 0.50) | 259.42 (107.28 to 567.75) | 348.71 (152.34 to 735.42) | 0.98 (0.92 to 1.05) |
| Turkey | 176.28 (83.67 to 323.16) | 211.11 (100.41 to 376.46) | 0.55 (0.15 to 0.94) | 215.19 (93.84 to 451.30) | 318.71 (141.38 to 663.49) | 1.33 (0.85 to 1.81) |
| Turkmenistan | 241.32 (99.32 to 470.10) | 255.81 (109.01 to 476.04) | 0.07 (-0.04 to 0.17) | 514.22 (209.94 to 1101.46) | 663.87 (281.35 to 1402.41) | 1.01 (0.85 to 1.17) |
| Tuvalu | 235.61 (0.46 to 1420.31) | 269.44 (5.00 to 1207.60) | 0.50 (0.45 to 0.54) | 295.57 (0.98 to 1716.51) | 401.03 (14.96 to 1700.10) | 1.03 (0.96 to 1.10) |
| Uganda | 166.30 (72.67 to 313.01) | 198.87 (93.28 to 363.22) | 0.50 (0.47 to 0.53) | 181.52 (74.80 to 389.67) | 237.31 (100.54 to 506.78) | 0.80 (0.77 to 0.82) |
| Ukraine | 355.82 (172.44 to 627.19) | 359.15 (172.53 to 623.68) | 0.06 (0.00 to 0.12) | 564.52 (254.59 to 1158.41) | 646.46 (292.64 to 1342.53) | 0.57 (0.51 to 0.63) |
| United Arab Emirates | 202.41 (58.87 to 463.31) | 188.72 (79.21 to 362.75) | -0.24 (-0.31 to -0.18) | 313.87 (95.75 to 764.33) | 282.35 (110.49 to 628.66) | -0.36 (-0.49 to -0.24) |
| United Kingdom | 264.02 (126.75 to 470.32) | 260.33 (123.90 to 465.83) | -0.35 (-0.51 to -0.19) | 412.53 (186.42 to 856.60) | 432.78 (197.21 to 892.96) | -0.40 (-0.80 to 0.01) |
| United Republic of Tanzania | 195.22 (88.56 to 364.41) | 207.06 (96.43 to 377.15) | -0.07 (-0.18 to 0.04) | 244.77 (103.89 to 528.50) | 262.57 (114.46 to 548.59) | -0.26 (-0.46 to -0.05) |
| United States of America | 305.79 (148.40 to 537.96) | 332.30 (164.52 to 584.77) | 0.04 (-0.07 to 0.16) | 525.86 (241.39 to 1088.17) | 554.39 (255.22 to 1142.94) | 0.06 (-0.27 to 0.40) |
| United States Virgin Islands | 235.50 (47.47 to 602.19) | 238.05 (71.68 to 537.18) | 0.04 (-0.01 to 0.10) | 400.41 (94.07 to 1081.71) | 442.67 (144.35 to 1062.11) | 0.26 (0.24 to 0.29) |
| Uruguay | 232.17 (102.92 to 428.62) | 236.89 (104.05 to 443.83) | 0.03 (0.01 to 0.05) | 417.69 (176.07 to 882.51) | 435.36 (189.30 to 915.60) | 0.16 (0.13 to 0.18) |
| Uzbekistan | 239.55 (105.44 to 444.45) | 252.13 (117.60 to 453.16) | -0.01 (-0.11 to 0.09) | 552.25 (244.06 to 1153.42) | 635.46 (283.01 to 1315.81) | 0.47 (0.36 to 0.58) |
| Vanuatu | 169.89 (21.31 to 507.22) | 200.55 (51.63 to 469.83) | 0.55 (0.53 to 0.58) | 178.99 (19.08 to 597.30) | 230.34 (55.84 to 619.01) | 0.79 (0.76 to 0.81) |
| Venezuela (Bolivarian Republic of) | 215.10 (95.17 to 398.23) | 234.83 (110.96 to 427.76) | -0.02 (-0.18 to 0.14) | 369.32 (160.24 to 780.85) | 414.80 (185.39 to 863.65) | -0.10 (-0.35 to 0.16) |
| Viet Nam | 173.27 (82.05 to 312.12) | 226.38 (109.85 to 403.01) | 0.90 (0.87 to 0.94) | 180.86 (79.14 to 383.24) | 278.36 (124.11 to 574.35) | 1.56 (1.51 to 1.61) |
| Yemen | 148.57 (64.00 to 280.35) | 187.19 (86.84 to 347.33) | 0.94 (0.86 to 1.02) | 159.93 (62.66 to 342.89) | 248.59 (106.84 to 524.94) | 1.77 (1.62 to 1.92) |
| Zambia | 196.66 (82.16 to 377.38) | 214.76 (97.34 to 393.92) | 0.36 (0.28 to 0.44) | 254.70 (101.67 to 557.51) | 277.69 (116.33 to 594.50) | 0.41 (0.24 to 0.59) |
| Zimbabwe | 185.53 (81.18 to 349.02) | 203.20 (92.61 to 373.97) | 0.14 (0.08 to 0.20) | 221.51 (89.67 to 479.35) | 238.93 (100.53 to 511.37) | -0.05 (-0.16 to 0.07) |

**Abbreviations: DALY=disability-adjusted life-years. EAPC=estimated annual percentage change. CI=confidence interval**

**Table S4.** **Age-standardised incidence rates and DALY rates in 1990 and 2021, and estimated annual percentage changes for hip osteoarthritis in older adults, by country.**

| **Countries** | **Age-standardised incidence rate, 1990** | **Age-standardised incidence rate, 2021** | **EAPC of incidence rate No. (95% CI)** | **Age-standardised DALYs rate, 1990** | **Age-standardised DALYs rate, 2021** | **EAPC of DALYs rate No. (95% CI)** |
| --- | --- | --- | --- | --- | --- | --- |
| Afghanistan | 39.71 (15.93 to 78.41) | 47.25 (19.76 to 91.27) | 0.70 (0.59 to 0.80) | 29.66 (10.50 to 68.22) | 35.93 (13.30 to 79.35) | 0.79 (0.68 to 0.90) |
| Albania | 113.97 (44.87 to 227.53) | 127.88 (54.80 to 245.46) | 0.50 (0.43 to 0.57) | 74.07 (25.63 to 176.15) | 86.56 (33.44 to 192.77) | 0.67 (0.61 to 0.72) |
| Algeria | 43.57 (18.67 to 85.06) | 57.68 (26.02 to 109.23) | 0.96 (0.92 to 1.00) | 35.27 (13.48 to 77.14) | 47.11 (20.05 to 101.00) | 1.01 (0.98 to 1.05) |
| American Samoa | 71.30 (0.07 to 462.99) | 77.02 (2.40 to 321.11) | 0.18 (0.08 to 0.27) | 65.23 (0.02 to 470.80) | 71.26 (1.49 to 332.90) | 0.18 (0.01 to 0.35) |
| Andorra | 90.32 (9.15 to 293.73) | 95.68 (22.46 to 238.53) | 0.11 (-0.01 to 0.24) | 117.74 (10.68 to 415.67) | 139.94 (31.11 to 383.90) | 0.57 (0.47 to 0.66) |
| Angola | 64.60 (25.83 to 128.06) | 73.26 (31.94 to 139.66) | 0.47 (0.43 to 0.50) | 49.99 (17.54 to 115.50) | 58.29 (22.87 to 129.61) | 0.58 (0.53 to 0.63) |
| Antigua and Barbuda | 63.30 (2.65 to 246.15) | 70.55 (7.77 to 222.71) | 0.35 (0.32 to 0.38) | 56.93 (1.52 to 253.80) | 61.58 (4.91 to 225.65) | 0.23 (0.21 to 0.26) |
| Argentina | 63.77 (34.13 to 108.06) | 75.33 (40.33 to 128.38) | 0.55 (0.53 to 0.57) | 90.73 (39.99 to 188.16) | 117.82 (53.24 to 245.84) | 0.88 (0.78 to 0.98) |
| Armenia | 117.05 (45.75 to 232.20) | 133.53 (57.36 to 254.28) | 0.81 (0.58 to 1.04) | 75.30 (26.95 to 176.89) | 87.84 (34.84 to 193.32) | 0.93 (0.77 to 1.09) |
| Australia | 67.58 (35.67 to 115.32) | 80.84 (42.79 to 137.43) | 0.64 (0.57 to 0.72) | 102.60 (44.93 to 212.40) | 136.50 (62.22 to 282.74) | 0.91 (0.82 to 1.00) |
| Austria | 80.42 (42.79 to 137.42) | 87.02 (44.36 to 146.11) | 0.20 (0.14 to 0.26) | 117.21 (50.57 to 247.09) | 137.50 (60.83 to 288.82) | 0.50 (0.47 to 0.54) |
| Azerbaijan | 116.27 (48.14 to 225.88) | 127.14 (57.00 to 238.68) | 0.49 (0.36 to 0.62) | 77.11 (29.28 to 172.93) | 86.75 (35.62 to 186.98) | 0.69 (0.54 to 0.83) |
| Bahamas | 67.78 (9.22 to 202.22) | 75.05 (19.43 to 182.99) | 0.35 (0.30 to 0.40) | 62.10 (6.84 to 209.73) | 66.02 (14.29 to 182.44) | 0.24 (0.21 to 0.27) |
| Bahrain | 49.38 (4.11 to 169.13) | 58.95 (16.77 to 138.09) | 0.59 (0.57 to 0.62) | 41.03 (2.18 to 160.29) | 49.19 (12.01 to 131.15) | 0.61 (0.59 to 0.63) |
| Bangladesh | 32.17 (14.36 to 60.06) | 39.67 (18.92 to 72.43) | 0.71 (0.69 to 0.73) | 32.11 (13.29 to 68.22) | 40.54 (17.40 to 84.69) | 0.86 (0.80 to 0.91) |
| Barbados | 67.73 (16.11 to 168.65) | 76.52 (22.49 to 173.76) | 0.35 (0.31 to 0.40) | 63.60 (13.24 to 178.76) | 68.52 (17.83 to 176.80) | 0.23 (0.19 to 0.27) |
| Belarus | 121.67 (54.61 to 228.57) | 136.69 (62.07 to 252.95) | 0.60 (0.47 to 0.74) | 79.80 (33.30 to 174.92) | 90.80 (38.86 to 193.31) | 0.68 (0.58 to 0.79) |
| Belgium | 80.06 (42.14 to 135.51) | 87.03 (44.64 to 147.51) | 0.02 (-0.09 to 0.14) | 114.49 (49.89 to 239.59) | 133.70 (59.77 to 279.07) | 0.50 (0.44 to 0.55) |
| Belize | 67.02 (5.87 to 224.17) | 78.46 (17.43 to 199.67) | 0.49 (0.40 to 0.59) | 60.55 (3.95 to 230.76) | 70.41 (13.79 to 204.27) | 0.50 (0.38 to 0.62) |
| Benin | 65.86 (23.81 to 137.27) | 76.13 (31.75 to 150.01) | 0.48 (0.44 to 0.52) | 52.15 (16.74 to 126.67) | 62.57 (23.52 to 140.48) | 0.62 (0.57 to 0.66) |
| Bermuda | 74.20 (4.26 to 274.08) | 82.08 (13.26 to 232.77) | 0.33 (0.30 to 0.36) | 70.03 (3.02 to 287.40) | 76.32 (10.79 to 241.17) | 0.28 (0.26 to 0.30) |
| Bhutan | 35.32 (3.32 to 115.36) | 46.14 (11.44 to 112.40) | 0.86 (0.83 to 0.88) | 35.81 (2.76 to 130.44) | 49.67 (11.51 to 135.10) | 1.17 (1.12 to 1.22) |
| Bolivia (Plurinational State of) | 57.38 (22.78 to 115.49) | 67.89 (30.16 to 128.59) | 0.58 (0.55 to 0.61) | 48.54 (17.25 to 112.70) | 58.48 (23.09 to 129.53) | 0.68 (0.64 to 0.72) |
| Bosnia and Herzegovina | 111.99 (46.50 to 216.98) | 132.99 (58.69 to 250.74) | 0.70 (0.66 to 0.74) | 73.04 (26.98 to 166.17) | 87.65 (35.60 to 191.42) | 0.82 (0.74 to 0.89) |
| Botswana | 71.08 (20.02 to 166.04) | 89.11 (32.39 to 185.50) | 0.75 (0.72 to 0.78) | 59.33 (13.82 to 162.25) | 76.39 (25.08 to 185.19) | 0.87 (0.82 to 0.92) |
| Brazil | 67.06 (32.38 to 121.12) | 81.39 (39.55 to 146.24) | 0.68 (0.64 to 0.72) | 55.01 (24.58 to 115.16) | 67.58 (30.66 to 140.18) | 0.75 (0.71 to 0.79) |
| Brunei Darussalam | 54.80 (4.17 to 190.78) | 71.85 (19.02 to 171.34) | 0.96 (0.89 to 1.02) | 73.29 (5.31 to 270.22) | 85.15 (19.19 to 237.13) | 0.58 (0.52 to 0.64) |
| Bulgaria | 116.09 (52.39 to 218.72) | 135.25 (61.70 to 252.05) | 0.53 (0.47 to 0.59) | 82.05 (34.11 to 176.64) | 93.20 (39.62 to 198.81) | 0.45 (0.42 to 0.49) |
| Burkina Faso | 59.49 (23.61 to 118.00) | 70.03 (30.39 to 134.67) | 0.49 (0.46 to 0.53) | 47.02 (16.70 to 107.72) | 56.30 (22.08 to 125.70) | 0.59 (0.56 to 0.62) |
| Burundi | 65.49 (24.75 to 134.07) | 68.75 (28.27 to 135.46) | 0.19 (0.17 to 0.20) | 52.12 (17.35 to 125.74) | 53.61 (19.55 to 123.41) | 0.13 (0.11 to 0.14) |
| Cabo Verde | 69.67 (13.65 to 183.78) | 83.50 (21.63 to 199.08) | 0.66 (0.62 to 0.70) | 56.67 (8.93 to 172.78) | 67.14 (15.44 to 182.51) | 0.71 (0.65 to 0.77) |
| Cambodia | 44.21 (17.23 to 88.51) | 47.21 (20.60 to 89.63) | 0.25 (0.22 to 0.28) | 36.35 (12.39 to 85.57) | 38.72 (15.32 to 85.60) | 0.29 (0.26 to 0.32) |
| Cameroon | 72.58 (29.33 to 143.26) | 79.94 (35.31 to 152.59) | 0.30 (0.26 to 0.33) | 60.24 (22.30 to 136.67) | 67.25 (27.30 to 145.31) | 0.37 (0.34 to 0.41) |
| Canada | 60.15 (32.09 to 99.47) | 75.87 (41.80 to 122.97) | 0.44 (0.19 to 0.69) | 82.88 (36.62 to 174.75) | 109.36 (49.67 to 225.35) | 0.54 (0.35 to 0.73) |
| Central African Republic | 63.08 (20.04 to 139.02) | 65.79 (24.01 to 135.80) | 0.18 (0.16 to 0.21) | 48.32 (12.45 to 125.95) | 51.01 (15.57 to 125.11) | 0.23 (0.21 to 0.25) |
| Chad | 59.22 (22.94 to 119.38) | 64.43 (26.85 to 125.42) | 0.23 (0.20 to 0.26) | 46.28 (15.68 to 109.11) | 50.11 (18.47 to 114.63) | 0.26 (0.23 to 0.28) |
| Chile | 67.18 (33.33 to 118.07) | 79.57 (41.26 to 136.84) | 0.51 (0.44 to 0.58) | 97.84 (40.93 to 209.95) | 125.22 (56.20 to 257.33) | 0.81 (0.71 to 0.90) |
| China | 38.24 (18.71 to 68.89) | 52.30 (25.70 to 93.85) | 1.26 (1.16 to 1.37) | 32.11 (14.53 to 66.11) | 43.56 (19.93 to 89.60) | 1.21 (1.11 to 1.30) |
| Colombia | 54.97 (24.38 to 103.40) | 65.07 (30.27 to 119.44) | 0.55 (0.53 to 0.57) | 47.84 (19.41 to 104.37) | 57.17 (24.96 to 121.01) | 0.58 (0.56 to 0.60) |
| Comoros | 69.64 (11.12 to 196.98) | 78.08 (21.25 to 185.59) | 0.42 (0.40 to 0.44) | 55.20 (6.12 to 183.73) | 62.49 (13.74 to 172.57) | 0.46 (0.44 to 0.47) |
| Congo | 69.71 (23.06 to 152.96) | 80.51 (30.91 to 163.64) | 0.51 (0.50 to 0.53) | 56.16 (15.61 to 142.16) | 65.87 (22.92 to 154.24) | 0.58 (0.56 to 0.60) |
| Cook Islands | 67.85 (0.00 to 609.94) | 77.33 (0.65 to 395.42) | 0.36 (0.30 to 0.42) | 62.74 (0.00 to 619.36) | 74.55 (0.39 to 410.51) | 0.48 (0.38 to 0.57) |
| Costa Rica | 58.40 (20.95 to 122.97) | 67.56 (27.89 to 132.02) | 0.49 (0.46 to 0.52) | 52.01 (16.76 to 125.73) | 59.69 (22.61 to 133.64) | 0.47 (0.44 to 0.49) |
| Côte d'Ivoire | 65.18 (25.47 to 132.39) | 72.97 (32.10 to 139.63) | 0.39 (0.37 to 0.41) | 51.21 (18.05 to 118.57) | 59.39 (23.79 to 130.37) | 0.48 (0.47 to 0.49) |
| Croatia | 120.43 (51.96 to 231.82) | 144.25 (63.77 to 269.24) | 0.77 (0.67 to 0.87) | 79.29 (31.56 to 171.61) | 96.00 (40.69 to 207.35) | 0.82 (0.76 to 0.89) |
| Cuba | 63.38 (28.04 to 120.04) | 74.90 (33.97 to 139.25) | 0.60 (0.56 to 0.64) | 57.16 (23.08 to 124.94) | 66.59 (28.49 to 143.44) | 0.57 (0.54 to 0.61) |
| Cyprus | 85.53 (34.47 to 166.98) | 92.49 (42.59 to 167.14) | 0.42 (0.37 to 0.48) | 103.76 (34.37 to 246.37) | 127.48 (49.20 to 287.50) | 0.68 (0.63 to 0.73) |
| Czechia | 124.97 (56.05 to 237.36) | 144.93 (66.14 to 267.34) | 0.49 (0.47 to 0.51) | 82.16 (34.56 to 173.56) | 97.30 (41.81 to 205.38) | 0.55 (0.51 to 0.58) |
| Democratic People's Republic of Korea | 38.48 (16.54 to 73.46) | 40.23 (18.24 to 74.75) | 0.13 (0.07 to 0.20) | 32.87 (12.55 to 72.89) | 34.40 (14.12 to 73.55) | 0.18 (0.08 to 0.28) |
| Democratic Republic of the Congo | 67.03 (30.87 to 126.31) | 70.09 (32.95 to 130.18) | 0.15 (0.12 to 0.18) | 52.69 (20.78 to 114.97) | 54.68 (22.99 to 117.69) | 0.11 (0.07 to 0.16) |
| Denmark | 91.46 (47.90 to 161.09) | 94.25 (47.87 to 162.45) | 0.37 (0.21 to 0.54) | 156.85 (69.35 to 329.37) | 147.75 (64.86 to 310.84) | -0.30 (-0.49 to -0.11) |
| Djibouti | 65.33 (5.69 to 221.52) | 77.53 (22.03 to 181.35) | 0.64 (0.61 to 0.67) | 51.49 (2.66 to 204.22) | 63.68 (14.61 to 171.68) | 0.78 (0.74 to 0.82) |
| Dominica | 63.19 (3.12 to 240.80) | 72.66 (6.43 to 243.51) | 0.46 (0.43 to 0.50) | 56.51 (1.83 to 244.47) | 63.56 (3.89 to 243.65) | 0.38 (0.33 to 0.42) |
| Dominican Republic | 60.58 (24.71 to 120.25) | 73.12 (32.17 to 138.98) | 0.68 (0.64 to 0.72) | 53.50 (19.53 to 121.75) | 64.71 (26.29 to 143.41) | 0.72 (0.68 to 0.76) |
| Ecuador | 67.47 (27.74 to 132.55) | 77.34 (34.80 to 145.34) | 0.40 (0.38 to 0.43) | 60.39 (22.74 to 136.95) | 69.80 (29.58 to 149.21) | 0.42 (0.38 to 0.46) |
| Egypt | 47.55 (20.89 to 90.51) | 57.25 (26.30 to 107.18) | 0.53 (0.47 to 0.59) | 38.24 (15.33 to 81.03) | 47.37 (19.94 to 100.08) | 0.58 (0.51 to 0.66) |
| El Salvador | 56.58 (21.89 to 115.27) | 66.84 (27.95 to 131.13) | 0.56 (0.49 to 0.64) | 49.50 (16.65 to 114.85) | 59.19 (23.07 to 129.32) | 0.59 (0.51 to 0.67) |
| Equatorial Guinea | 65.13 (9.68 to 189.99) | 87.37 (23.91 to 206.78) | 1.18 (1.11 to 1.25) | 50.18 (4.98 to 174.99) | 72.34 (17.11 to 195.38) | 1.49 (1.38 to 1.59) |
| Eritrea | 59.06 (17.54 to 134.01) | 65.20 (24.80 to 131.23) | 0.35 (0.32 to 0.38) | 45.45 (10.84 to 122.84) | 51.23 (16.97 to 122.95) | 0.42 (0.40 to 0.44) |
| Estonia | 122.38 (47.98 to 242.96) | 153.69 (64.29 to 298.87) | 0.84 (0.72 to 0.96) | 81.52 (28.64 to 188.84) | 100.88 (39.11 to 225.03) | 0.90 (0.81 to 0.98) |
| Eswatini | 81.83 (17.87 to 206.81) | 87.99 (25.88 to 201.09) | 0.11 (0.01 to 0.21) | 70.79 (12.56 to 211.42) | 77.28 (19.74 to 204.89) | 0.13 (0.01 to 0.24) |
| Ethiopia | 70.92 (32.76 to 132.22) | 83.77 (39.15 to 154.85) | 0.59 (0.56 to 0.62) | 52.86 (21.66 to 115.38) | 63.28 (27.70 to 131.47) | 0.66 (0.62 to 0.69) |
| Fiji | 60.07 (12.18 to 159.63) | 67.41 (20.43 to 151.58) | 0.33 (0.29 to 0.38) | 52.83 (8.94 to 160.58) | 59.62 (15.95 to 154.80) | 0.32 (0.26 to 0.38) |
| Finland | 84.96 (44.12 to 147.21) | 92.11 (47.33 to 158.56) | 0.36 (0.30 to 0.42) | 120.69 (50.70 to 258.98) | 139.83 (60.82 to 287.51) | 0.47 (0.42 to 0.52) |
| France | 79.39 (43.79 to 132.14) | 90.20 (49.53 to 150.82) | 0.21 (0.09 to 0.33) | 121.30 (55.21 to 250.32) | 142.95 (65.52 to 292.78) | 0.55 (0.32 to 0.78) |
| Gabon | 75.43 (22.00 to 174.73) | 90.39 (30.77 to 192.82) | 0.61 (0.58 to 0.64) | 61.09 (14.86 to 163.97) | 75.55 (22.55 to 186.75) | 0.74 (0.70 to 0.78) |
| Gambia | 62.99 (13.44 to 162.02) | 73.91 (23.92 to 159.91) | 0.50 (0.48 to 0.52) | 50.91 (8.54 to 152.31) | 59.92 (16.45 to 152.45) | 0.55 (0.52 to 0.57) |
| Georgia | 123.92 (53.18 to 236.39) | 142.17 (61.70 to 270.32) | 0.58 (0.50 to 0.65) | 83.59 (32.83 to 185.22) | 91.24 (37.12 to 200.17) | 0.48 (0.41 to 0.55) |
| Germany | 80.81 (44.78 to 134.47) | 85.77 (47.51 to 142.92) | 0.05 (-0.10 to 0.20) | 120.47 (55.28 to 249.47) | 140.93 (65.52 to 288.16) | 0.51 (0.47 to 0.54) |
| Ghana | 62.40 (26.39 to 121.92) | 75.58 (34.13 to 143.93) | 0.64 (0.61 to 0.68) | 50.27 (18.82 to 114.27) | 62.62 (25.23 to 136.82) | 0.76 (0.74 to 0.79) |
| Greece | 79.20 (41.65 to 133.93) | 77.00 (40.60 to 131.10) | 0.05 (-0.16 to 0.26) | 107.69 (46.59 to 225.12) | 127.16 (56.48 to 269.16) | 1.29 (0.98 to 1.60) |
| Greenland | 69.75 (0.49 to 373.73) | 91.38 (9.93 to 290.51) | 0.71 (0.56 to 0.88) | 86.71 (0.65 to 469.81) | 109.05 (9.50 to 391.73) | 0.81 (0.74 to 0.89) |
| Grenada | 61.66 (3.87 to 222.89) | 68.52 (7.60 to 215.41) | 0.30 (0.23 to 0.37) | 54.91 (2.44 to 223.94) | 59.50 (4.61 to 218.60) | 0.14 (0.08 to 0.20) |
| Guam | 65.11 (3.15 to 251.13) | 76.57 (15.37 to 201.34) | 0.58 (0.56 to 0.61) | 59.24 (1.72 to 259.12) | 70.90 (12.55 to 210.03) | 0.67 (0.59 to 0.75) |
| Guatemala | 50.08 (19.34 to 100.30) | 58.56 (25.90 to 109.93) | 0.51 (0.49 to 0.54) | 41.87 (13.83 to 99.72) | 49.99 (20.31 to 108.59) | 0.62 (0.58 to 0.65) |
| Guinea | 63.07 (25.33 to 126.78) | 70.09 (29.29 to 136.56) | 0.42 (0.40 to 0.44) | 49.81 (17.65 to 115.79) | 55.62 (20.80 to 125.88) | 0.43 (0.41 to 0.46) |
| Guinea-Bissau | 60.10 (13.47 to 152.32) | 64.87 (18.20 to 151.43) | 0.20 (0.16 to 0.23) | 47.73 (8.16 to 142.68) | 51.77 (11.95 to 141.41) | 0.21 (0.17 to 0.24) |
| Guyana | 61.09 (13.95 to 154.06) | 69.06 (20.44 to 157.82) | 0.42 (0.39 to 0.45) | 52.00 (9.86 to 150.33) | 58.27 (14.62 to 153.60) | 0.42 (0.37 to 0.47) |
| Haiti | 52.30 (20.28 to 104.68) | 55.95 (23.88 to 106.35) | 0.29 (0.26 to 0.32) | 42.78 (14.15 to 100.63) | 45.42 (16.94 to 101.99) | 0.32 (0.28 to 0.36) |
| Honduras | 52.11 (18.97 to 107.66) | 58.68 (25.05 to 112.56) | 0.39 (0.34 to 0.44) | 44.87 (14.00 to 108.14) | 50.71 (19.50 to 113.97) | 0.41 (0.36 to 0.46) |
| Hungary | 126.77 (56.83 to 238.38) | 147.05 (66.74 to 274.25) | 0.52 (0.50 to 0.55) | 85.33 (36.36 to 182.37) | 98.53 (42.86 to 207.08) | 0.54 (0.50 to 0.57) |
| Iceland | 99.04 (32.24 to 217.63) | 102.98 (39.81 to 207.64) | -0.03 (-0.21 to 0.15) | 152.52 (43.73 to 381.37) | 157.02 (52.82 to 375.27) | -0.05 (-0.14 to 0.04) |
| India | 35.80 (17.14 to 65.11) | 45.80 (22.40 to 82.45) | 0.75 (0.58 to 0.91) | 34.15 (15.38 to 70.26) | 44.86 (20.63 to 91.51) | 1.00 (0.86 to 1.13) |
| Indonesia | 51.95 (25.07 to 94.52) | 58.18 (28.60 to 103.90) | 0.45 (0.42 to 0.49) | 41.40 (18.17 to 86.73) | 47.00 (21.10 to 97.55) | 0.51 (0.47 to 0.55) |
| Iran (Islamic Republic of) | 50.53 (22.52 to 94.84) | 66.03 (30.33 to 121.92) | 1.08 (0.95 to 1.21) | 38.12 (15.87 to 82.62) | 49.25 (21.60 to 102.68) | 0.93 (0.86 to 0.99) |
| Iraq | 56.86 (23.11 to 112.30) | 57.09 (25.24 to 108.62) | 0.09 (0.05 to 0.13) | 43.85 (16.64 to 97.52) | 45.60 (19.10 to 98.83) | 0.18 (0.14 to 0.23) |
| Ireland | 86.18 (41.96 to 152.41) | 96.00 (48.40 to 165.95) | 0.49 (0.44 to 0.53) | 117.91 (47.94 to 252.61) | 139.18 (59.16 to 292.55) | 0.49 (0.44 to 0.53) |
| Israel | 81.84 (40.26 to 144.86) | 92.58 (47.30 to 157.34) | 0.35 (0.25 to 0.46) | 109.46 (45.04 to 237.21) | 128.51 (55.41 to 270.06) | 0.45 (0.37 to 0.53) |
| Italy | 77.92 (42.34 to 130.63) | 78.12 (41.82 to 132.97) | 0.06 (0.01 to 0.12) | 111.08 (51.19 to 227.91) | 127.89 (59.13 to 262.20) | 0.63 (0.53 to 0.73) |
| Jamaica | 63.96 (23.32 to 132.24) | 76.24 (29.99 to 154.35) | 0.67 (0.59 to 0.75) | 57.39 (19.39 to 136.51) | 67.01 (24.58 to 151.62) | 0.60 (0.51 to 0.68) |
| Japan | 52.42 (27.86 to 89.60) | 48.75 (25.11 to 84.81) | -0.23 (-0.29 to -0.17) | 74.70 (34.64 to 154.52) | 84.52 (39.61 to 173.02) | 0.45 (0.35 to 0.56) |
| Jordan | 50.89 (15.40 to 115.25) | 61.57 (25.20 to 122.40) | 0.68 (0.65 to 0.70) | 39.73 (10.26 to 103.72) | 50.88 (19.27 to 112.86) | 0.85 (0.83 to 0.87) |
| Kazakhstan | 123.28 (54.28 to 235.64) | 127.05 (59.13 to 234.59) | 0.20 (0.09 to 0.31) | 82.95 (34.60 to 179.80) | 89.23 (38.52 to 191.51) | 0.39 (0.31 to 0.47) |
| Kenya | 80.47 (35.00 to 153.36) | 88.07 (40.64 to 163.04) | 0.34 (0.31 to 0.36) | 60.92 (24.16 to 134.40) | 67.88 (28.74 to 145.23) | 0.42 (0.37 to 0.46) |
| Kiribati | 56.89 (0.26 to 313.65) | 60.64 (1.94 to 250.11) | 0.14 (0.06 to 0.23) | 48.40 (0.06 to 311.90) | 52.01 (0.86 to 248.93) | 0.15 (0.00 to 0.30) |
| Kuwait | 57.83 (12.88 to 145.23) | 73.14 (26.71 to 153.56) | 0.99 (0.92 to 1.06) | 46.91 (9.09 to 135.29) | 58.97 (19.68 to 138.48) | 0.98 (0.90 to 1.06) |
| Kyrgyzstan | 113.27 (45.35 to 222.96) | 117.35 (50.11 to 224.43) | 0.27 (0.17 to 0.37) | 74.06 (27.20 to 168.83) | 78.12 (29.72 to 172.91) | 0.36 (0.28 to 0.44) |
| Lao People's Democratic Republic | 45.34 (16.01 to 95.08) | 51.15 (20.61 to 101.36) | 0.48 (0.45 to 0.51) | 37.45 (11.44 to 92.42) | 43.03 (15.35 to 100.67) | 0.54 (0.51 to 0.56) |
| Latvia | 126.17 (52.51 to 246.17) | 150.77 (63.97 to 290.13) | 0.63 (0.53 to 0.74) | 83.25 (32.07 to 187.10) | 99.47 (39.66 to 222.09) | 0.72 (0.65 to 0.80) |
| Lebanon | 49.56 (17.16 to 104.89) | 68.14 (27.43 to 134.50) | 1.04 (0.74 to 1.34) | 38.13 (11.57 to 92.84) | 52.21 (20.27 to 115.06) | 1.01 (0.73 to 1.30) |
| Lesotho | 70.70 (23.00 to 154.75) | 78.61 (27.54 to 166.95) | 0.32 (0.29 to 0.35) | 58.19 (16.12 to 149.05) | 65.60 (19.96 to 162.54) | 0.36 (0.33 to 0.38) |
| Liberia | 66.57 (22.65 to 143.59) | 77.09 (28.35 to 160.17) | 0.62 (0.56 to 0.67) | 53.43 (15.65 to 131.91) | 62.26 (20.56 to 147.78) | 0.70 (0.62 to 0.78) |
| Libya | 56.69 (18.86 to 122.73) | 63.41 (24.82 to 126.62) | 0.43 (0.40 to 0.46) | 44.09 (13.55 to 108.64) | 50.26 (18.57 to 114.60) | 0.49 (0.45 to 0.54) |
| Lithuania | 128.14 (53.87 to 245.21) | 149.67 (65.30 to 282.13) | 0.57 (0.46 to 0.68) | 83.40 (32.79 to 186.90) | 97.52 (40.10 to 213.32) | 0.64 (0.58 to 0.70) |
| Luxembourg | 81.78 (29.73 to 169.02) | 91.46 (38.10 to 177.52) | 0.14 (0.04 to 0.24) | 119.68 (37.86 to 292.37) | 138.03 (49.24 to 313.85) | 0.48 (0.44 to 0.52) |
| Madagascar | 67.01 (27.60 to 131.69) | 68.67 (30.10 to 131.67) | 0.15 (0.12 to 0.18) | 52.33 (19.20 to 118.43) | 53.80 (21.11 to 119.00) | 0.19 (0.15 to 0.22) |
| Malawi | 67.34 (26.73 to 134.76) | 74.74 (32.32 to 144.46) | 0.44 (0.39 to 0.49) | 52.66 (18.71 to 122.34) | 60.29 (24.12 to 134.30) | 0.52 (0.47 to 0.57) |
| Malaysia | 53.62 (22.40 to 104.54) | 60.68 (27.75 to 113.59) | 0.43 (0.40 to 0.45) | 46.19 (17.86 to 104.48) | 52.89 (22.23 to 114.27) | 0.52 (0.48 to 0.55) |
| Maldives | 47.76 (1.46 to 200.47) | 58.39 (11.01 to 156.62) | 0.75 (0.67 to 0.82) | 39.57 (0.49 to 198.50) | 50.91 (7.73 to 157.22) | 1.08 (0.96 to 1.20) |
| Mali | 58.68 (23.51 to 116.02) | 66.41 (28.83 to 127.80) | 0.41 (0.40 to 0.43) | 45.81 (15.93 to 107.61) | 52.87 (20.93 to 116.70) | 0.51 (0.49 to 0.52) |
| Malta | 85.56 (28.99 to 179.79) | 94.04 (39.23 to 181.17) | 0.39 (0.32 to 0.46) | 113.32 (32.72 to 284.99) | 138.81 (49.68 to 316.35) | 0.57 (0.46 to 0.67) |
| Marshall Islands | 52.64 (0.00 to 467.86) | 59.53 (0.19 to 340.04) | 0.37 (0.31 to 0.43) | 45.14 (0.00 to 468.95) | 49.53 (0.03 to 341.37) | 0.18 (0.07 to 0.29) |
| Mauritania | 68.78 (22.83 to 149.87) | 81.41 (31.14 to 167.63) | 0.52 (0.47 to 0.57) | 56.92 (16.53 to 143.75) | 68.72 (23.59 to 160.62) | 0.58 (0.52 to 0.63) |
| Mauritius | 51.61 (14.44 to 122.36) | 60.53 (22.51 to 126.75) | 0.56 (0.53 to 0.59) | 44.15 (10.01 to 118.59) | 51.91 (16.98 to 123.91) | 0.57 (0.51 to 0.62) |
| Mexico | 66.80 (31.30 to 122.72) | 75.19 (35.99 to 136.70) | 0.17 (0.04 to 0.30) | 57.29 (24.97 to 119.78) | 64.46 (29.05 to 133.63) | 0.18 (0.04 to 0.31) |
| Micronesia (Federated States of) | 59.87 (1.31 to 264.53) | 65.01 (2.66 to 258.80) | 0.21 (0.14 to 0.28) | 53.12 (0.61 to 272.48) | 56.35 (1.24 to 260.90) | 0.09 (-0.07 to 0.26) |
| Monaco | 82.39 (10.23 to 248.97) | 93.39 (16.91 to 255.02) | 0.54 (0.43 to 0.65) | 132.72 (18.17 to 418.46) | 150.73 (27.57 to 436.84) | 0.36 (0.31 to 0.42) |
| Mongolia | 106.35 (37.90 to 223.29) | 113.34 (43.50 to 227.93) | 0.13 (0.04 to 0.22) | 70.67 (21.04 to 175.38) | 76.91 (26.71 to 178.90) | 0.29 (0.23 to 0.36) |
| Montenegro | 135.38 (44.51 to 292.89) | 128.44 (48.07 to 262.94) | 0.01 (-0.08 to 0.10) | 89.31 (25.68 to 226.26) | 89.46 (29.44 to 214.17) | 0.24 (0.15 to 0.34) |
| Morocco | 47.07 (20.09 to 90.39) | 56.83 (25.66 to 106.56) | 0.65 (0.63 to 0.67) | 36.07 (13.84 to 79.68) | 44.66 (18.73 to 97.14) | 0.74 (0.72 to 0.76) |
| Mozambique | 66.20 (28.01 to 129.29) | 72.54 (31.78 to 138.46) | 0.36 (0.32 to 0.40) | 51.00 (19.16 to 116.05) | 57.08 (22.98 to 128.25) | 0.41 (0.39 to 0.44) |
| Myanmar | 45.00 (20.62 to 83.27) | 50.62 (23.91 to 93.06) | 0.47 (0.42 to 0.53) | 37.20 (15.10 to 81.12) | 41.98 (17.89 to 89.89) | 0.56 (0.50 to 0.62) |
| Namibia | 70.37 (21.00 to 161.71) | 79.13 (28.52 to 165.62) | 0.42 (0.38 to 0.45) | 57.99 (14.22 to 155.26) | 67.10 (20.89 to 163.92) | 0.48 (0.45 to 0.52) |
| Nauru | 63.14 (0.00 to 1433.75) | 65.41 (0.00 to 1154.96) | 0.06 (0.01 to 0.11) | 54.92 (0.00 to 1439.58) | 57.91 (0.00 to 1162.27) | 0.06 (-0.00 to 0.11) |
| Nepal | 32.67 (13.12 to 64.19) | 42.66 (19.29 to 80.48) | 0.91 (0.87 to 0.96) | 31.67 (11.73 to 71.76) | 43.87 (18.05 to 95.03) | 1.14 (1.10 to 1.18) |
| Netherlands | 89.84 (48.13 to 151.04) | 93.76 (49.65 to 157.28) | 0.23 (0.09 to 0.37) | 129.48 (57.42 to 267.58) | 137.81 (62.18 to 282.49) | 0.14 (0.06 to 0.23) |
| New Zealand | 66.15 (31.10 to 121.83) | 79.32 (39.06 to 142.40) | 0.70 (0.65 to 0.74) | 102.75 (42.03 to 222.20) | 131.13 (56.73 to 279.46) | 0.79 (0.71 to 0.86) |
| Nicaragua | 53.74 (18.27 to 114.27) | 61.86 (25.48 to 121.28) | 0.43 (0.37 to 0.50) | 46.38 (13.75 to 114.31) | 53.99 (20.15 to 121.47) | 0.48 (0.40 to 0.56) |
| Niger | 58.58 (22.31 to 118.25) | 63.36 (26.93 to 121.40) | 0.23 (0.18 to 0.28) | 46.09 (15.10 to 111.13) | 50.25 (19.13 to 112.05) | 0.25 (0.20 to 0.30) |
| Nigeria | 72.04 (33.68 to 132.68) | 75.07 (35.39 to 137.18) | -0.06 (-0.20 to 0.09) | 55.21 (24.01 to 117.15) | 58.45 (25.80 to 122.75) | 0.01 (-0.14 to 0.15) |
| Niue | 64.00 (0.00 to 2034.10) | 72.73 (0.00 to 2262.62) | 0.34 (0.28 to 0.40) | 60.12 (0.00 to 2048.77) | 68.06 (0.00 to 2275.35) | 0.41 (0.32 to 0.50) |
| North Macedonia | 125.60 (48.77 to 252.15) | 118.56 (50.34 to 229.03) | 0.05 (-0.11 to 0.21) | 81.24 (28.18 to 192.06) | 83.82 (32.18 to 187.15) | 0.33 (0.24 to 0.42) |
| Northern Mariana Islands | 69.30 (0.00 to 661.99) | 72.27 (2.03 to 308.23) | 0.09 (0.03 to 0.16) | 62.74 (0.00 to 669.53) | 64.85 (1.01 to 319.90) | -0.06 (-0.21 to 0.10) |
| Norway | 76.63 (37.88 to 135.54) | 88.42 (44.64 to 154.45) | 0.69 (0.52 to 0.86) | 114.64 (49.02 to 244.34) | 134.17 (58.58 to 281.58) | 0.51 (0.47 to 0.55) |
| Oman | 44.90 (10.42 to 110.78) | 59.98 (20.29 to 129.23) | 0.98 (0.88 to 1.09) | 34.07 (6.33 to 99.81) | 49.64 (15.01 to 121.41) | 1.28 (1.22 to 1.35) |
| Pakistan | 38.52 (17.83 to 71.12) | 48.77 (23.17 to 88.91) | 0.81 (0.78 to 0.83) | 37.89 (16.13 to 79.56) | 48.52 (21.37 to 102.27) | 0.88 (0.84 to 0.91) |
| Palau | 64.83 (0.00 to 690.43) | 73.22 (0.12 to 452.75) | 0.35 (0.27 to 0.42) | 59.26 (0.00 to 702.80) | 65.82 (0.04 to 458.82) | 0.20 (0.03 to 0.38) |
| Palestine | 48.64 (13.46 to 113.93) | 54.09 (19.32 to 114.01) | 0.38 (0.31 to 0.46) | 38.09 (8.79 to 102.36) | 42.88 (13.53 to 103.95) | 0.38 (0.33 to 0.43) |
| Panama | 52.28 (17.79 to 110.51) | 64.59 (26.30 to 126.98) | 0.64 (0.61 to 0.68) | 44.95 (13.41 to 109.79) | 56.64 (21.26 to 130.67) | 0.69 (0.66 to 0.72) |
| Papua New Guinea | 51.18 (17.80 to 109.32) | 54.63 (22.06 to 106.49) | 0.20 (0.16 to 0.24) | 42.22 (12.32 to 104.72) | 45.48 (15.93 to 104.55) | 0.16 (0.07 to 0.25) |
| Paraguay | 62.70 (23.67 to 128.12) | 70.69 (30.08 to 136.08) | 0.45 (0.41 to 0.48) | 54.23 (18.27 to 129.18) | 60.89 (23.79 to 136.79) | 0.42 (0.38 to 0.46) |
| Peru | 61.66 (26.86 to 117.33) | 73.29 (33.57 to 135.50) | 0.66 (0.62 to 0.70) | 53.42 (21.24 to 117.90) | 64.93 (28.14 to 135.28) | 0.78 (0.73 to 0.82) |
| Philippines | 52.86 (24.47 to 98.13) | 58.29 (27.89 to 105.98) | 0.31 (0.30 to 0.33) | 43.29 (18.12 to 92.99) | 47.48 (20.93 to 98.98) | 0.31 (0.30 to 0.32) |
| Poland | 136.34 (63.46 to 249.55) | 163.95 (77.15 to 295.99) | 0.76 (0.69 to 0.83) | 86.08 (38.17 to 181.13) | 104.46 (47.23 to 218.36) | 0.81 (0.75 to 0.87) |
| Portugal | 83.73 (43.96 to 140.58) | 84.21 (43.94 to 142.11) | -0.30 (-0.46 to -0.14) | 108.87 (47.14 to 228.17) | 132.32 (58.94 to 271.30) | 0.38 (0.26 to 0.51) |
| Puerto Rico | 73.67 (30.29 to 147.91) | 87.97 (37.63 to 172.21) | 0.64 (0.58 to 0.69) | 69.59 (26.40 to 156.89) | 83.39 (34.99 to 179.37) | 0.64 (0.59 to 0.69) |
| Qatar | 55.90 (1.37 to 242.18) | 65.40 (16.11 to 160.99) | 0.53 (0.50 to 0.55) | 46.32 (0.49 to 232.32) | 56.15 (11.87 to 155.08) | 0.60 (0.55 to 0.66) |
| Republic of Korea | 62.37 (32.26 to 107.14) | 61.96 (33.23 to 105.55) | -0.16 (-0.29 to -0.03) | 77.22 (33.44 to 164.04) | 87.73 (39.76 to 184.79) | 0.46 (0.40 to 0.51) |
| Republic of Moldova | 107.66 (45.15 to 211.11) | 140.05 (60.83 to 266.07) | 1.11 (0.98 to 1.25) | 72.07 (27.63 to 161.60) | 92.15 (37.61 to 199.63) | 1.07 (0.95 to 1.20) |
| Romania | 114.45 (52.74 to 211.05) | 143.53 (66.69 to 262.21) | 0.86 (0.77 to 0.95) | 77.76 (33.70 to 164.70) | 96.30 (42.91 to 203.71) | 0.87 (0.80 to 0.94) |
| Russian Federation | 125.12 (59.23 to 226.69) | 153.72 (74.03 to 274.28) | 0.88 (0.76 to 1.00) | 80.05 (36.38 to 166.62) | 97.53 (44.49 to 202.03) | 0.91 (0.80 to 1.01) |
| Rwanda | 65.88 (25.64 to 133.01) | 73.33 (31.23 to 142.14) | 0.43 (0.39 to 0.47) | 51.99 (17.90 to 121.31) | 58.45 (22.09 to 130.93) | 0.47 (0.42 to 0.52) |
| Saint Kitts and Nevis | 64.23 (1.60 to 277.63) | 75.05 (4.64 to 275.10) | 0.54 (0.47 to 0.60) | 58.39 (0.84 to 286.82) | 64.52 (2.45 to 275.89) | 0.36 (0.25 to 0.47) |
| Saint Lucia | 61.71 (5.03 to 208.87) | 73.58 (15.08 to 191.93) | 0.62 (0.58 to 0.67) | 54.15 (2.96 to 211.41) | 65.03 (11.00 to 190.72) | 0.61 (0.56 to 0.67) |
| Saint Vincent and the Grenadines | 59.76 (3.67 to 218.84) | 71.59 (10.67 to 205.78) | 0.66 (0.61 to 0.72) | 52.39 (1.96 to 220.41) | 62.84 (7.32 to 209.78) | 0.69 (0.63 to 0.76) |
| Samoa | 65.33 (4.44 to 233.12) | 68.48 (8.90 to 205.86) | 0.12 (0.07 to 0.16) | 58.25 (2.63 to 237.60) | 62.09 (6.22 to 213.11) | 0.14 (0.04 to 0.24) |
| San Marino | 84.65 (4.11 to 330.92) | 87.01 (11.44 to 263.57) | 0.10 (0.05 to 0.15) | 125.52 (7.07 to 495.13) | 143.91 (20.90 to 442.83) | 0.43 (0.37 to 0.49) |
| Sao Tome and Principe | 67.86 (3.26 to 257.84) | 78.00 (7.63 to 251.39) | 0.47 (0.45 to 0.49) | 54.85 (1.55 to 245.06) | 63.90 (4.22 to 242.13) | 0.57 (0.54 to 0.59) |
| Saudi Arabia | 51.10 (20.07 to 103.04) | 62.62 (27.36 to 123.74) | 0.75 (0.72 to 0.78) | 39.84 (14.26 to 91.17) | 52.32 (21.02 to 113.72) | 0.97 (0.93 to 1.00) |
| Senegal | 66.05 (25.84 to 134.06) | 72.96 (30.52 to 142.36) | 0.30 (0.28 to 0.32) | 53.48 (18.58 to 124.43) | 58.86 (23.11 to 131.17) | 0.31 (0.30 to 0.32) |
| Serbia | 118.59 (53.94 to 221.44) | 141.14 (63.96 to 262.94) | 0.94 (0.77 to 1.10) | 78.93 (32.74 to 173.33) | 95.08 (40.89 to 202.55) | 0.89 (0.80 to 0.98) |
| Seychelles | 55.80 (1.73 to 232.33) | 62.09 (5.90 to 204.44) | 0.32 (0.30 to 0.35) | 50.21 (0.92 to 233.46) | 53.88 (3.64 to 205.41) | 0.24 (0.22 to 0.25) |
| Sierra Leone | 63.20 (23.21 to 130.55) | 69.28 (27.70 to 138.95) | 0.25 (0.22 to 0.28) | 50.17 (16.42 to 119.39) | 55.85 (20.06 to 126.94) | 0.31 (0.27 to 0.34) |
| Singapore | 56.57 (23.77 to 109.03) | 66.11 (31.56 to 117.49) | 0.45 (0.39 to 0.52) | 74.60 (26.39 to 172.65) | 89.67 (37.19 to 192.15) | 0.64 (0.59 to 0.68) |
| Slovakia | 125.24 (54.53 to 240.73) | 137.74 (61.76 to 255.53) | 0.34 (0.29 to 0.38) | 83.18 (33.55 to 184.10) | 93.81 (39.09 to 202.13) | 0.40 (0.36 to 0.44) |
| Slovenia | 128.86 (52.11 to 256.83) | 155.98 (66.92 to 294.13) | 0.68 (0.60 to 0.75) | 83.85 (30.97 to 191.44) | 101.38 (40.81 to 223.54) | 0.73 (0.69 to 0.77) |
| Solomon Islands | 54.86 (4.76 to 184.11) | 58.82 (11.67 to 155.80) | 0.18 (0.11 to 0.24) | 46.29 (2.40 to 183.72) | 50.96 (7.94 to 155.69) | 0.25 (0.15 to 0.35) |
| Somalia | 65.78 (23.61 to 137.84) | 64.33 (27.13 to 124.92) | -0.01 (-0.03 to 0.02) | 51.44 (16.30 to 125.71) | 50.23 (18.20 to 115.10) | 0.01 (-0.02 to 0.04) |
| South Africa | 94.76 (43.28 to 176.47) | 102.67 (48.26 to 189.26) | 0.34 (0.31 to 0.38) | 77.20 (32.85 to 164.14) | 83.82 (36.84 to 174.55) | 0.39 (0.34 to 0.44) |
| South Sudan | 69.88 (27.33 to 142.60) | 76.57 (30.44 to 153.07) | 0.35 (0.28 to 0.42) | 54.27 (19.01 to 125.75) | 59.42 (21.26 to 135.28) | 0.30 (0.25 to 0.35) |
| Spain | 87.17 (47.87 to 145.88) | 88.27 (48.15 to 146.61) | -0.13 (-0.22 to -0.04) | 122.99 (54.62 to 253.82) | 145.62 (66.80 to 301.62) | 0.46 (0.34 to 0.59) |
| Sri Lanka | 48.34 (20.82 to 93.33) | 52.61 (24.54 to 97.63) | 0.32 (0.30 to 0.33) | 40.92 (15.95 to 89.20) | 44.30 (18.45 to 94.43) | 0.27 (0.26 to 0.29) |
| Sudan | 40.42 (16.92 to 79.07) | 54.67 (23.99 to 104.04) | 1.06 (1.02 to 1.09) | 30.91 (11.43 to 69.14) | 43.03 (17.81 to 93.75) | 1.11 (1.09 to 1.13) |
| Suriname | 65.27 (12.14 to 175.91) | 73.56 (22.31 to 167.45) | 0.43 (0.41 to 0.45) | 57.33 (8.87 to 178.28) | 64.79 (16.94 to 169.21) | 0.51 (0.47 to 0.55) |
| Sweden | 67.90 (34.31 to 118.01) | 81.81 (41.86 to 141.98) | 0.80 (0.29 to 1.32) | 87.57 (37.93 to 187.84) | 130.41 (57.67 to 269.69) | 1.08 (0.57 to 1.59) |
| Switzerland | 76.52 (38.87 to 131.37) | 82.36 (42.88 to 141.09) | 0.32 (0.28 to 0.35) | 115.61 (49.11 to 241.49) | 128.62 (57.21 to 266.86) | 0.34 (0.34 to 0.35) |
| Syrian Arab Republic | 51.18 (20.01 to 102.71) | 54.50 (23.44 to 105.70) | 0.34 (0.28 to 0.39) | 38.97 (13.82 to 89.92) | 44.19 (17.64 to 96.61) | 0.47 (0.44 to 0.50) |
| Taiwan (Province of China) | 44.86 (19.73 to 86.15) | 52.29 (23.93 to 98.20) | 0.59 (0.54 to 0.64) | 39.40 (15.62 to 86.64) | 45.65 (19.16 to 96.35) | 0.67 (0.58 to 0.76) |
| Tajikistan | 109.45 (43.45 to 215.48) | 105.55 (45.26 to 203.42) | 0.07 (-0.05 to 0.20) | 69.61 (24.76 to 161.03) | 69.28 (26.34 to 156.18) | 0.20 (0.11 to 0.29) |
| Thailand | 48.70 (22.38 to 90.88) | 60.15 (28.46 to 110.61) | 0.72 (0.69 to 0.76) | 41.08 (17.27 to 87.46) | 52.93 (23.28 to 111.01) | 0.94 (0.89 to 0.98) |
| Timor-Leste | 46.30 (5.88 to 139.08) | 46.38 (13.71 to 106.07) | 0.06 (0.01 to 0.10) | 37.21 (2.99 to 132.97) | 37.94 (9.26 to 101.30) | 0.13 (0.08 to 0.18) |
| Togo | 62.06 (20.08 to 136.35) | 67.15 (26.74 to 135.75) | 0.19 (0.15 to 0.23) | 49.50 (13.58 to 124.84) | 54.38 (19.35 to 125.36) | 0.26 (0.22 to 0.30) |
| Tokelau | 58.49 (0.00 to 3426.15) | 69.17 (0.00 to 2945.63) | 0.50 (0.47 to 0.53) | 52.92 (0.00 to 3433.36) | 64.03 (0.00 to 2960.77) | 0.65 (0.54 to 0.76) |
| Tonga | 63.16 (1.87 to 264.56) | 70.26 (5.20 to 245.03) | 0.21 (0.10 to 0.31) | 57.11 (0.93 to 273.09) | 64.51 (3.72 to 254.57) | 0.24 (0.08 to 0.39) |
| Trinidad and Tobago | 65.97 (21.05 to 146.11) | 76.22 (29.29 to 154.67) | 0.54 (0.48 to 0.60) | 59.23 (16.70 to 149.00) | 66.79 (23.17 to 155.56) | 0.48 (0.42 to 0.53) |
| Tunisia | 46.00 (18.01 to 93.05) | 58.73 (25.28 to 112.26) | 0.86 (0.83 to 0.89) | 36.57 (12.60 to 83.75) | 45.98 (18.13 to 99.26) | 0.80 (0.77 to 0.83) |
| Turkey | 51.62 (22.84 to 99.02) | 62.75 (28.92 to 116.61) | 0.79 (0.73 to 0.84) | 40.29 (16.72 to 87.03) | 50.49 (22.35 to 105.61) | 0.83 (0.79 to 0.87) |
| Turkmenistan | 112.22 (42.31 to 228.56) | 131.03 (54.03 to 255.66) | 0.74 (0.61 to 0.87) | 75.13 (24.75 to 176.45) | 88.39 (33.76 to 199.46) | 0.76 (0.67 to 0.85) |
| Tuvalu | 55.57 (0.00 to 879.34) | 63.77 (0.00 to 639.36) | 0.40 (0.35 to 0.44) | 46.91 (0.00 to 882.18) | 56.55 (0.00 to 643.66) | 0.49 (0.34 to 0.64) |
| Uganda | 67.18 (28.22 to 130.24) | 75.02 (33.27 to 140.88) | 0.41 (0.38 to 0.44) | 52.52 (19.44 to 118.57) | 59.74 (24.38 to 130.81) | 0.48 (0.44 to 0.53) |
| Ukraine | 130.26 (62.17 to 238.78) | 147.08 (69.98 to 266.47) | 0.57 (0.44 to 0.70) | 83.07 (36.46 to 173.19) | 93.39 (41.77 to 192.24) | 0.59 (0.50 to 0.68) |
| United Arab Emirates | 51.02 (8.34 to 143.80) | 57.58 (21.38 to 122.38) | 0.40 (0.33 to 0.46) | 40.63 (4.85 to 133.38) | 48.77 (15.42 to 117.04) | 0.64 (0.57 to 0.71) |
| United Kingdom | 83.42 (45.14 to 140.12) | 93.10 (50.07 to 156.95) | 0.46 (0.41 to 0.50) | 127.51 (58.85 to 261.65) | 148.17 (68.92 to 303.96) | 0.49 (0.44 to 0.53) |
| United Republic of Tanzania | 69.88 (31.22 to 134.03) | 80.72 (36.83 to 152.46) | 0.51 (0.49 to 0.53) | 55.69 (22.06 to 123.60) | 65.53 (27.69 to 138.78) | 0.58 (0.55 to 0.61) |
| United States of America | 77.71 (42.94 to 131.10) | 96.57 (53.15 to 159.74) | 0.00 (-0.37 to 0.36) | 143.87 (68.36 to 292.91) | 159.75 (76.39 to 323.47) | 0.38 (0.27 to 0.50) |
| United States Virgin Islands | 71.77 (5.58 to 253.11) | 81.73 (16.79 to 216.29) | 0.42 (0.36 to 0.49) | 66.88 (3.83 to 260.75) | 76.40 (13.69 to 226.34) | 0.38 (0.35 to 0.40) |
| Uruguay | 62.40 (29.14 to 113.13) | 71.54 (34.42 to 128.15) | 0.37 (0.32 to 0.41) | 93.38 (36.63 to 207.21) | 122.60 (51.44 to 265.21) | 0.91 (0.82 to 0.99) |
| Uzbekistan | 120.49 (52.95 to 225.02) | 120.65 (54.88 to 224.54) | 0.12 (-0.01 to 0.25) | 77.10 (31.90 to 168.47) | 81.82 (35.23 to 176.33) | 0.39 (0.29 to 0.48) |
| Vanuatu | 56.34 (1.30 to 245.15) | 59.72 (7.46 to 180.69) | 0.22 (0.20 to 0.24) | 48.12 (0.50 to 245.82) | 51.51 (4.74 to 181.60) | 0.22 (0.18 to 0.26) |
| Venezuela (Bolivarian Republic of) | 57.24 (24.90 to 110.46) | 64.74 (29.68 to 119.87) | 0.42 (0.38 to 0.46) | 50.77 (20.11 to 112.23) | 56.58 (24.08 to 121.39) | 0.38 (0.35 to 0.42) |
| Viet Nam | 44.36 (20.63 to 82.56) | 48.69 (23.10 to 89.00) | 0.36 (0.31 to 0.41) | 37.13 (15.45 to 79.82) | 40.28 (17.34 to 84.52) | 0.43 (0.37 to 0.48) |
| Yemen | 37.38 (14.27 to 75.16) | 45.03 (19.50 to 86.16) | 0.70 (0.66 to 0.74) | 28.16 (9.10 to 67.45) | 34.36 (13.33 to 75.80) | 0.72 (0.68 to 0.76) |
| Zambia | 71.72 (27.93 to 145.38) | 76.97 (32.99 to 149.89) | 0.26 (0.22 to 0.31) | 56.99 (19.48 to 134.49) | 62.28 (24.23 to 139.31) | 0.33 (0.27 to 0.38) |
| Zimbabwe | 73.36 (30.31 to 143.63) | 73.57 (32.26 to 140.05) | -0.06 (-0.13 to 0.01) | 61.14 (22.59 to 141.60) | 61.02 (24.16 to 136.40) | -0.11 (-0.18 to -0.03) |

**Abbreviations: DALY=disability-adjusted life-years. EAPC=estimated annual percentage change. CI=confidence interval**

**Table S5. Age-standardised incidence rates and DALY rates in 1990 and 2021, and estimated annual percentage changes for knee osteoarthritis in older adults, by country.**

| **Countries** | **Age-standardised incidence rate, 1990** | **Age-standardised incidence rate, 2021** | **EAPC of incidence rate No. (95% CI)** | **Age-standardised DALYs rate, 1990** | **Age-standardised DALYs rate, 2021** | **EAPC of DALYs rate No. (95% CI)** |
| --- | --- | --- | --- | --- | --- | --- |
| Afghanistan | 851.50 (591.56 to 1172.35) | 907.93 (637.97 to 1251.41) | 0.26 (0.24 to 0.28) | 483.26 (218.40 to 990.10) | 534.45 (244.25 to 1097.33) | 0.42 (0.37 to 0.47) |
| Albania | 817.26 (552.97 to 1154.53) | 857.15 (587.39 to 1190.18) | 0.12 (0.10 to 0.14) | 476.39 (206.19 to 1004.44) | 517.34 (229.30 to 1083.15) | 0.33 (0.31 to 0.34) |
| Algeria | 913.72 (638.75 to 1268.07) | 967.49 (684.98 to 1320.22) | 0.18 (0.16 to 0.20) | 537.61 (245.84 to 1105.40) | 605.94 (285.35 to 1240.07) | 0.42 (0.40 to 0.43) |
| American Samoa | 843.04 (271.61 to 1852.91) | 890.28 (396.59 to 1654.39) | 0.13 (0.10 to 0.16) | 617.65 (92.19 to 1921.75) | 660.94 (163.18 to 1744.66) | 0.16 (0.09 to 0.23) |
| Andorra | 1072.37 (555.75 to 1808.52) | 1086.89 (647.87 to 1678.77) | -0.13 (-0.21 to -0.06) | 679.04 (188.98 to 1737.72) | 728.03 (257.18 to 1684.38) | 0.21 (0.17 to 0.26) |
| Angola | 879.75 (598.47 to 1227.35) | 939.03 (655.06 to 1299.62) | 0.20 (0.18 to 0.21) | 508.42 (222.98 to 1058.97) | 558.64 (257.61 to 1146.91) | 0.33 (0.31 to 0.34) |
| Antigua and Barbuda | 1001.10 (509.10 to 1702.26) | 1101.91 (613.16 to 1753.38) | 0.34 (0.32 to 0.36) | 684.05 (190.82 to 1726.51) | 719.98 (236.03 to 1706.44) | 0.16 (0.15 to 0.17) |
| Argentina | 1023.25 (733.55 to 1390.86) | 1090.50 (774.68 to 1474.58) | 0.03 (-0.02 to 0.09) | 697.82 (328.29 to 1406.36) | 773.18 (363.85 to 1568.91) | 0.31 (0.26 to 0.35) |
| Armenia | 731.07 (489.59 to 1046.44) | 779.72 (534.70 to 1100.80) | 0.07 (0.01 to 0.13) | 419.53 (178.88 to 895.53) | 452.96 (203.85 to 944.57) | 0.43 (0.37 to 0.49) |
| Australia | 1028.79 (732.69 to 1413.51) | 1094.69 (773.86 to 1495.57) | 0.06 (0.00 to 0.12) | 727.25 (343.33 to 1472.85) | 804.53 (384.18 to 1628.36) | 0.27 (0.24 to 0.30) |
| Austria | 1048.89 (737.65 to 1441.89) | 1065.78 (758.00 to 1447.84) | -0.13 (-0.17 to -0.08) | 708.70 (333.73 to 1460.36) | 742.23 (350.33 to 1521.00) | 0.11 (0.09 to 0.12) |
| Azerbaijan | 739.82 (500.62 to 1044.08) | 810.41 (562.72 to 1130.38) | 0.22 (0.19 to 0.25) | 433.00 (191.02 to 907.67) | 458.08 (209.83 to 933.92) | 0.33 (0.26 to 0.40) |
| Bahamas | 1078.12 (635.13 to 1691.37) | 1124.63 (708.12 to 1655.90) | 0.16 (0.14 to 0.18) | 722.20 (249.13 to 1690.83) | 752.77 (301.92 to 1661.11) | 0.13 (0.12 to 0.14) |
| Bahrain | 968.65 (538.19 to 1564.42) | 1049.29 (678.51 to 1512.95) | 0.22 (0.20 to 0.24) | 572.37 (181.26 to 1360.33) | 615.62 (252.22 to 1309.23) | 0.27 (0.25 to 0.29) |
| Bangladesh | 843.47 (597.96 to 1145.69) | 905.66 (642.53 to 1242.36) | 0.26 (0.24 to 0.29) | 495.26 (233.24 to 990.12) | 539.06 (256.98 to 1089.60) | 0.32 (0.30 to 0.35) |
| Barbados | 1035.64 (639.57 to 1552.43) | 1102.88 (714.18 to 1613.01) | 0.22 (0.20 to 0.24) | 728.91 (280.38 to 1617.41) | 755.61 (311.04 to 1642.33) | 0.13 (0.10 to 0.15) |
| Belarus | 867.48 (606.01 to 1199.40) | 916.84 (644.12 to 1256.58) | 0.01 (-0.04 to 0.07) | 535.88 (248.65 to 1091.43) | 571.56 (264.99 to 1155.42) | 0.30 (0.27 to 0.33) |
| Belgium | 1031.97 (727.46 to 1405.89) | 1061.07 (748.15 to 1436.79) | -0.17 (-0.25 to -0.09) | 693.69 (320.83 to 1410.39) | 727.46 (341.99 to 1491.27) | 0.13 (0.10 to 0.16) |
| Belize | 1024.14 (562.33 to 1665.80) | 1116.51 (686.04 to 1669.08) | 0.23 (0.19 to 0.27) | 689.61 (214.81 to 1661.93) | 751.22 (286.54 to 1682.35) | 0.27 (0.21 to 0.33) |
| Benin | 865.57 (581.67 to 1233.33) | 940.39 (643.41 to 1305.11) | 0.24 (0.22 to 0.26) | 550.87 (238.19 to 1166.76) | 617.71 (278.29 to 1264.03) | 0.37 (0.34 to 0.40) |
| Bermuda | 1104.19 (572.84 to 1870.92) | 1085.76 (632.03 to 1695.79) | -0.08 (-0.10 to -0.07) | 762.89 (223.81 to 1908.56) | 789.57 (283.10 to 1818.56) | 0.11 (0.09 to 0.12) |
| Bhutan | 897.90 (522.84 to 1388.87) | 938.16 (594.32 to 1374.54) | 0.13 (0.12 to 0.14) | 523.86 (179.60 to 1216.31) | 588.56 (235.37 to 1275.83) | 0.42 (0.40 to 0.45) |
| Bolivia (Plurinational State of) | 1064.90 (731.17 to 1496.64) | 1144.83 (809.22 to 1562.66) | 0.17 (0.15 to 0.20) | 652.65 (293.59 to 1368.33) | 729.64 (341.76 to 1468.14) | 0.37 (0.35 to 0.40) |
| Bosnia and Herzegovina | 826.97 (572.12 to 1165.81) | 860.20 (599.39 to 1195.17) | 0.02 (-0.04 to 0.08) | 478.98 (211.27 to 995.35) | 517.68 (236.95 to 1055.62) | 0.34 (0.30 to 0.38) |
| Botswana | 929.19 (592.76 to 1356.85) | 986.34 (671.64 to 1404.99) | 0.14 (0.10 to 0.18) | 553.86 (217.28 to 1211.42) | 620.45 (265.79 to 1322.02) | 0.38 (0.37 to 0.39) |
| Brazil | 1067.91 (770.47 to 1449.85) | 1099.87 (789.96 to 1493.35) | 0.10 (0.09 to 0.12) | 657.38 (314.32 to 1327.04) | 726.38 (348.63 to 1454.01) | 0.35 (0.34 to 0.37) |
| Brunei Darussalam | 1269.98 (706.65 to 2040.30) | 1431.55 (920.49 to 2087.78) | 0.32 (0.28 to 0.36) | 910.42 (300.38 to 2152.51) | 972.64 (385.10 to 2121.01) | 0.24 (0.21 to 0.26) |
| Bulgaria | 878.15 (616.96 to 1208.49) | 870.56 (611.08 to 1204.41) | -0.06 (-0.10 to -0.03) | 510.05 (233.26 to 1048.04) | 541.18 (249.22 to 1100.77) | 0.19 (0.18 to 0.20) |
| Burkina Faso | 879.26 (606.72 to 1230.23) | 922.57 (642.92 to 1273.22) | 0.16 (0.16 to 0.17) | 537.25 (238.51 to 1115.57) | 593.35 (271.66 to 1212.09) | 0.32 (0.32 to 0.33) |
| Burundi | 857.93 (584.10 to 1213.94) | 882.49 (614.15 to 1236.43) | 0.07 (0.05 to 0.08) | 519.01 (230.01 to 1089.28) | 524.89 (236.22 to 1095.19) | 0.05 (0.05 to 0.06) |
| Cabo Verde | 837.06 (491.40 to 1306.84) | 911.74 (564.19 to 1370.20) | 0.14 (0.07 to 0.21) | 574.09 (207.60 to 1302.05) | 627.51 (246.46 to 1390.50) | 0.34 (0.32 to 0.36) |
| Cambodia | 659.67 (441.24 to 953.09) | 719.93 (493.34 to 1013.33) | 0.30 (0.28 to 0.31) | 410.71 (181.73 to 840.11) | 451.77 (208.82 to 936.54) | 0.35 (0.33 to 0.37) |
| Cameroon | 921.61 (628.61 to 1295.14) | 972.42 (676.06 to 1341.90) | 0.12 (0.10 to 0.14) | 594.58 (269.39 to 1241.38) | 640.74 (294.75 to 1301.81) | 0.23 (0.23 to 0.24) |
| Canada | 681.74 (488.90 to 914.79) | 748.53 (531.66 to 1018.63) | -0.02 (-0.23 to 0.18) | 405.23 (189.98 to 826.55) | 455.40 (217.23 to 923.88) | 0.08 (-0.08 to 0.25) |
| Central African Republic | 894.62 (585.62 to 1286.22) | 927.75 (629.53 to 1308.68) | 0.10 (0.09 to 0.12) | 503.98 (209.37 to 1083.18) | 527.97 (228.55 to 1114.58) | 0.16 (0.15 to 0.17) |
| Chad | 846.34 (571.16 to 1186.14) | 888.08 (616.17 to 1232.67) | 0.15 (0.14 to 0.17) | 525.73 (233.86 to 1084.99) | 550.81 (250.31 to 1149.28) | 0.14 (0.13 to 0.15) |
| Chile | 1034.19 (737.14 to 1416.24) | 1107.33 (782.85 to 1518.53) | 0.05 (-0.00 to 0.10) | 716.00 (332.36 to 1455.48) | 786.62 (369.10 to 1616.45) | 0.29 (0.25 to 0.32) |
| China | 859.71 (601.08 to 1197.09) | 911.73 (639.02 to 1263.06) | 0.06 (-0.07 to 0.20) | 654.25 (315.31 to 1299.70) | 704.44 (339.39 to 1402.95) | 0.41 (0.30 to 0.52) |
| Colombia | 1039.60 (736.41 to 1424.54) | 1057.75 (746.34 to 1440.58) | 0.04 (0.02 to 0.05) | 657.82 (305.69 to 1337.94) | 723.25 (341.33 to 1464.95) | 0.32 (0.31 to 0.34) |
| Comoros | 888.73 (515.78 to 1393.69) | 913.96 (578.03 to 1360.16) | 0.07 (0.06 to 0.08) | 530.88 (176.45 to 1262.20) | 566.62 (221.13 to 1239.06) | 0.23 (0.23 to 0.24) |
| Congo | 917.62 (606.98 to 1323.15) | 954.77 (646.53 to 1358.85) | 0.11 (0.09 to 0.12) | 546.65 (227.13 to 1159.90) | 585.35 (261.86 to 1221.75) | 0.23 (0.22 to 0.23) |
| Cook Islands | 811.70 (194.68 to 2031.40) | 878.69 (337.78 to 1743.46) | 0.27 (0.25 to 0.30) | 587.67 (53.81 to 2083.29) | 672.00 (136.93 to 1908.41) | 0.43 (0.39 to 0.47) |
| Costa Rica | 1032.92 (691.38 to 1455.25) | 1074.64 (749.39 to 1483.27) | 0.10 (0.08 to 0.12) | 680.15 (300.02 to 1431.55) | 734.76 (334.68 to 1520.57) | 0.26 (0.24 to 0.27) |
| Côte d'Ivoire | 907.55 (620.66 to 1263.55) | 939.15 (654.46 to 1298.99) | 0.08 (0.07 to 0.10) | 548.31 (244.29 to 1137.33) | 602.46 (278.89 to 1224.72) | 0.27 (0.26 to 0.29) |
| Croatia | 850.13 (590.12 to 1189.02) | 854.35 (593.95 to 1187.75) | -0.06 (-0.10 to -0.01) | 498.06 (225.90 to 1026.28) | 537.03 (247.12 to 1109.60) | 0.31 (0.28 to 0.35) |
| Cuba | 1029.77 (725.32 to 1419.34) | 1049.98 (745.72 to 1441.51) | 0.13 (0.11 to 0.14) | 679.55 (312.44 to 1402.70) | 736.94 (346.84 to 1502.28) | 0.30 (0.29 to 0.31) |
| Cyprus | 1084.21 (726.60 to 1534.63) | 1109.54 (763.55 to 1544.98) | -0.04 (-0.07 to -0.01) | 658.35 (275.25 to 1416.53) | 713.71 (319.51 to 1476.51) | 0.24 (0.22 to 0.26) |
| Czechia | 843.23 (587.15 to 1164.88) | 868.47 (608.21 to 1187.95) | 0.08 (0.06 to 0.10) | 501.74 (232.66 to 1026.77) | 539.07 (252.36 to 1102.43) | 0.21 (0.19 to 0.23) |
| Democratic People's Republic of Korea | 841.96 (566.84 to 1189.08) | 894.73 (621.45 to 1245.11) | 0.20 (0.18 to 0.22) | 639.52 (296.91 to 1276.36) | 679.48 (319.15 to 1386.92) | 0.20 (0.17 to 0.23) |
| Democratic Republic of the Congo | 907.64 (642.52 to 1246.82) | 917.87 (651.80 to 1246.21) | -0.02 (-0.06 to 0.01) | 522.41 (241.18 to 1067.76) | 541.48 (249.35 to 1108.50) | 0.08 (0.07 to 0.10) |
| Denmark | 1003.08 (704.58 to 1380.92) | 1073.87 (750.73 to 1459.16) | 0.18 (0.15 to 0.21) | 667.40 (311.71 to 1376.61) | 716.07 (331.53 to 1479.28) | 0.17 (0.13 to 0.20) |
| Djibouti | 882.64 (469.36 to 1455.71) | 944.16 (602.61 to 1413.81) | 0.24 (0.23 to 0.25) | 516.99 (150.06 to 1269.95) | 568.74 (224.24 to 1247.59) | 0.36 (0.34 to 0.37) |
| Dominica | 1049.43 (548.14 to 1764.46) | 1099.24 (603.60 to 1794.97) | 0.14 (0.12 to 0.16) | 699.90 (201.22 to 1759.56) | 734.65 (229.17 to 1764.91) | 0.16 (0.11 to 0.21) |
| Dominican Republic | 1032.65 (711.46 to 1444.73) | 1074.73 (754.71 to 1477.17) | 0.17 (0.14 to 0.19) | 660.73 (296.07 to 1377.57) | 729.84 (339.55 to 1504.21) | 0.36 (0.35 to 0.38) |
| Ecuador | 1076.75 (749.31 to 1479.96) | 1137.96 (797.24 to 1565.10) | 0.23 (0.20 to 0.26) | 706.09 (320.51 to 1442.51) | 776.87 (363.43 to 1577.47) | 0.37 (0.34 to 0.40) |
| Egypt | 933.40 (659.57 to 1288.71) | 1016.78 (721.22 to 1387.33) | 0.23 (0.21 to 0.26) | 552.08 (255.71 to 1131.44) | 610.62 (286.77 to 1232.67) | 0.26 (0.23 to 0.29) |
| El Salvador | 1024.98 (697.33 to 1447.34) | 1048.55 (732.49 to 1454.86) | 0.06 (0.04 to 0.07) | 664.08 (298.64 to 1378.35) | 734.77 (339.99 to 1511.69) | 0.33 (0.29 to 0.37) |
| Equatorial Guinea | 884.25 (508.33 to 1403.78) | 973.90 (613.05 to 1439.66) | 0.34 (0.30 to 0.37) | 511.59 (169.29 to 1208.51) | 611.09 (239.00 to 1348.33) | 0.70 (0.65 to 0.74) |
| Eritrea | 888.40 (580.28 to 1282.65) | 911.36 (620.69 to 1302.41) | 0.07 (0.05 to 0.09) | 500.47 (200.16 to 1086.38) | 531.43 (227.19 to 1112.75) | 0.21 (0.19 to 0.22) |
| Estonia | 876.10 (591.24 to 1242.57) | 887.94 (603.55 to 1249.74) | 0.00 (-0.05 to 0.05) | 546.18 (238.57 to 1145.34) | 591.13 (264.02 to 1228.26) | 0.35 (0.32 to 0.38) |
| Eswatini | 952.06 (576.77 to 1459.56) | 1023.67 (658.34 to 1478.01) | 0.22 (0.18 to 0.27) | 601.17 (216.36 to 1376.12) | 638.37 (255.58 to 1407.62) | 0.13 (0.08 to 0.18) |
| Ethiopia | 886.71 (629.38 to 1212.14) | 897.92 (643.14 to 1222.80) | 0.03 (0.02 to 0.05) | 505.75 (233.33 to 1027.50) | 548.27 (259.56 to 1107.20) | 0.29 (0.27 to 0.32) |
| Fiji | 804.77 (472.19 to 1255.09) | 890.44 (563.51 to 1322.17) | 0.34 (0.32 to 0.36) | 558.02 (208.16 to 1252.41) | 634.91 (266.21 to 1348.51) | 0.39 (0.35 to 0.42) |
| Finland | 1064.75 (748.69 to 1452.45) | 1085.96 (767.00 to 1474.82) | -0.05 (-0.11 to -0.00) | 710.15 (328.32 to 1468.28) | 740.35 (346.47 to 1519.66) | 0.10 (0.08 to 0.12) |
| France | 1010.63 (718.13 to 1383.57) | 1052.60 (752.14 to 1409.66) | -0.10 (-0.18 to -0.01) | 686.36 (327.08 to 1406.69) | 725.09 (346.64 to 1477.35) | 0.17 (0.13 to 0.20) |
| Gabon | 894.93 (561.37 to 1322.90) | 981.34 (642.22 to 1414.27) | 0.23 (0.20 to 0.27) | 556.05 (221.46 to 1214.52) | 619.14 (261.61 to 1316.72) | 0.35 (0.32 to 0.38) |
| Gambia | 883.76 (537.09 to 1336.81) | 923.51 (607.50 to 1333.85) | 0.10 (0.07 to 0.13) | 554.23 (209.02 to 1259.43) | 604.83 (251.04 to 1311.59) | 0.28 (0.27 to 0.29) |
| Georgia | 767.09 (527.81 to 1074.57) | 771.63 (532.67 to 1063.27) | -0.06 (-0.10 to -0.03) | 449.20 (199.67 to 934.41) | 458.63 (208.14 to 955.82) | 0.15 (0.12 to 0.18) |
| Germany | 1047.27 (746.60 to 1416.42) | 1055.36 (750.93 to 1437.05) | -0.16 (-0.23 to -0.09) | 725.73 (345.67 to 1461.53) | 744.63 (355.97 to 1502.48) | 0.09 (0.08 to 0.11) |
| Ghana | 889.11 (608.45 to 1227.77) | 969.83 (674.41 to 1328.57) | 0.22 (0.20 to 0.25) | 553.91 (250.76 to 1146.17) | 630.49 (294.35 to 1280.38) | 0.42 (0.41 to 0.43) |
| Greece | 1054.08 (747.23 to 1424.65) | 1035.37 (729.67 to 1410.17) | -0.31 (-0.42 to -0.20) | 696.68 (324.97 to 1436.66) | 745.32 (353.18 to 1533.61) | 0.24 (0.20 to 0.27) |
| Greenland | 764.40 (279.45 to 1557.91) | 839.66 (422.19 to 1445.19) | 0.14 (0.08 to 0.20) | 444.14 (63.68 to 1410.97) | 483.88 (119.30 to 1274.06) | 0.30 (0.26 to 0.33) |
| Grenada | 974.16 (518.96 to 1628.05) | 1106.12 (630.13 to 1758.58) | 0.56 (0.49 to 0.61) | 674.08 (205.62 to 1651.82) | 719.99 (237.57 to 1709.37) | 0.20 (0.17 to 0.22) |
| Guam | 824.60 (394.57 to 1466.69) | 858.46 (491.46 to 1354.47) | 0.12 (0.10 to 0.14) | 595.71 (159.26 to 1522.69) | 667.66 (245.17 to 1504.93) | 0.40 (0.38 to 0.43) |
| Guatemala | 1042.19 (726.04 to 1424.59) | 1062.37 (745.40 to 1461.04) | 0.01 (-0.01 to 0.03) | 622.54 (276.62 to 1280.59) | 685.02 (318.29 to 1396.31) | 0.32 (0.29 to 0.34) |
| Guinea | 868.47 (589.54 to 1230.32) | 902.92 (615.03 to 1258.48) | 0.03 (-0.01 to 0.07) | 543.54 (240.72 to 1128.93) | 577.90 (260.27 to 1185.66) | 0.17 (0.15 to 0.19) |
| Guinea-Bissau | 889.09 (551.21 to 1328.39) | 939.88 (605.94 to 1375.59) | 0.16 (0.15 to 0.17) | 540.63 (205.39 to 1207.18) | 577.15 (229.52 to 1253.44) | 0.19 (0.17 to 0.20) |
| Guyana | 1049.43 (660.44 to 1559.69) | 1121.73 (727.93 to 1621.00) | 0.16 (0.13 to 0.19) | 651.56 (254.25 to 1461.48) | 706.25 (290.88 to 1517.30) | 0.28 (0.24 to 0.31) |
| Haiti | 1035.87 (715.69 to 1437.36) | 1071.88 (753.84 to 1475.21) | 0.08 (0.06 to 0.10) | 605.98 (267.15 to 1255.24) | 632.73 (290.21 to 1293.53) | 0.19 (0.17 to 0.20) |
| Honduras | 1030.73 (700.34 to 1448.31) | 1099.38 (769.79 to 1518.63) | 0.23 (0.22 to 0.24) | 642.28 (280.89 to 1332.48) | 699.19 (320.50 to 1429.74) | 0.28 (0.26 to 0.30) |
| Hungary | 860.00 (596.38 to 1181.60) | 875.43 (611.28 to 1216.00) | 0.00 (-0.03 to 0.02) | 513.25 (238.92 to 1047.87) | 547.72 (254.92 to 1121.76) | 0.22 (0.20 to 0.24) |
| Iceland | 1035.70 (647.69 to 1536.47) | 1109.22 (735.64 to 1598.59) | 0.04 (-0.02 to 0.11) | 704.22 (271.74 to 1562.15) | 747.95 (312.18 to 1610.13) | 0.19 (0.16 to 0.21) |
| India | 920.34 (661.85 to 1250.28) | 974.93 (703.77 to 1321.55) | 0.16 (0.13 to 0.19) | 529.95 (253.65 to 1058.82) | 589.70 (284.69 to 1176.45) | 0.32 (0.28 to 0.36) |
| Indonesia | 670.98 (471.03 to 935.11) | 734.09 (521.57 to 1004.41) | 0.33 (0.32 to 0.34) | 421.33 (199.07 to 845.55) | 465.85 (222.26 to 938.77) | 0.39 (0.37 to 0.41) |
| Iran (Islamic Republic of) | 917.13 (655.35 to 1245.52) | 939.75 (675.78 to 1281.91) | -0.02 (-0.06 to 0.01) | 519.08 (242.17 to 1049.10) | 580.47 (275.76 to 1166.49) | 0.37 (0.34 to 0.39) |
| Iraq | 896.98 (614.55 to 1265.28) | 977.34 (681.78 to 1345.14) | 0.26 (0.26 to 0.27) | 572.93 (260.31 to 1201.13) | 597.77 (278.68 to 1201.61) | 0.15 (0.13 to 0.16) |
| Ireland | 1063.23 (738.89 to 1470.15) | 1110.77 (780.26 to 1524.30) | 0.03 (-0.01 to 0.07) | 699.35 (315.90 to 1450.15) | 738.71 (337.54 to 1523.69) | 0.14 (0.12 to 0.16) |
| Israel | 1063.86 (749.11 to 1453.62) | 1112.58 (793.00 to 1508.26) | -0.04 (-0.12 to 0.03) | 687.56 (313.87 to 1417.80) | 736.91 (343.33 to 1519.31) | 0.16 (0.12 to 0.20) |
| Italy | 1058.42 (763.66 to 1424.16) | 1046.56 (749.59 to 1415.62) | -0.22 (-0.28 to -0.16) | 690.24 (328.97 to 1409.81) | 728.96 (350.95 to 1490.76) | 0.16 (0.14 to 0.17) |
| Jamaica | 1017.76 (697.08 to 1435.26) | 1041.25 (717.99 to 1465.83) | 0.06 (0.03 to 0.09) | 684.43 (304.07 to 1451.17) | 727.52 (331.03 to 1524.21) | 0.22 (0.20 to 0.25) |
| Japan | 1282.96 (927.54 to 1723.48) | 1175.69 (846.12 to 1588.46) | -0.39 (-0.44 to -0.34) | 940.95 (453.23 to 1889.19) | 955.00 (459.37 to 1926.42) | 0.07 (0.05 to 0.09) |
| Jordan | 920.14 (606.71 to 1322.71) | 996.58 (694.07 to 1385.96) | 0.27 (0.23 to 0.30) | 557.06 (231.53 to 1180.93) | 625.87 (287.06 to 1274.85) | 0.42 (0.40 to 0.43) |
| Kazakhstan | 772.87 (529.75 to 1079.14) | 831.98 (578.08 to 1143.91) | 0.15 (0.12 to 0.19) | 447.24 (204.18 to 923.15) | 469.93 (216.40 to 964.05) | 0.23 (0.20 to 0.27) |
| Kenya | 881.19 (617.79 to 1220.32) | 955.28 (683.24 to 1307.48) | 0.26 (0.22 to 0.29) | 535.31 (245.23 to 1098.82) | 578.53 (270.05 to 1174.92) | 0.28 (0.26 to 0.29) |
| Kiribati | 822.34 (327.06 to 1632.27) | 896.35 (428.51 to 1586.23) | 0.27 (0.25 to 0.30) | 561.52 (109.49 to 1602.19) | 622.87 (164.37 to 1587.27) | 0.31 (0.25 to 0.37) |
| Kuwait | 925.13 (578.14 to 1389.84) | 987.93 (659.33 to 1430.59) | 0.11 (0.06 to 0.15) | 583.78 (227.78 to 1307.54) | 638.76 (281.24 to 1333.91) | 0.39 (0.36 to 0.43) |
| Kyrgyzstan | 737.80 (498.67 to 1054.63) | 778.85 (532.08 to 1086.98) | 0.05 (0.00 to 0.10) | 424.29 (183.03 to 899.75) | 435.73 (191.92 to 902.89) | 0.17 (0.14 to 0.21) |
| Lao People's Democratic Republic | 651.14 (424.18 to 949.86) | 702.87 (468.20 to 997.15) | 0.29 (0.28 to 0.30) | 409.49 (171.54 to 871.37) | 452.33 (199.25 to 937.42) | 0.40 (0.37 to 0.42) |
| Latvia | 877.80 (596.18 to 1236.21) | 888.30 (608.80 to 1253.50) | -0.02 (-0.07 to 0.02) | 548.14 (245.58 to 1143.52) | 589.02 (268.32 to 1221.76) | 0.30 (0.28 to 0.33) |
| Lebanon | 893.35 (602.56 to 1269.30) | 914.57 (624.70 to 1267.04) | 0.00 (-0.03 to 0.04) | 537.12 (233.01 to 1120.47) | 608.07 (278.91 to 1241.07) | 0.41 (0.34 to 0.48) |
| Lesotho | 886.66 (579.15 to 1274.99) | 981.71 (660.28 to 1406.36) | 0.36 (0.35 to 0.37) | 552.01 (230.11 to 1185.24) | 594.35 (251.31 to 1292.09) | 0.22 (0.20 to 0.24) |
| Liberia | 892.37 (596.46 to 1278.67) | 943.20 (629.78 to 1342.48) | 0.21 (0.18 to 0.24) | 557.49 (234.83 to 1184.76) | 608.52 (265.74 to 1270.68) | 0.37 (0.33 to 0.41) |
| Libya | 899.91 (598.46 to 1277.70) | 959.76 (656.57 to 1342.71) | 0.19 (0.18 to 0.21) | 571.48 (246.52 to 1200.23) | 613.09 (276.90 to 1265.43) | 0.25 (0.23 to 0.26) |
| Lithuania | 869.80 (601.54 to 1216.46) | 876.01 (606.23 to 1218.40) | -0.05 (-0.10 to 0.01) | 541.86 (244.95 to 1149.78) | 583.04 (265.07 to 1198.64) | 0.29 (0.27 to 0.32) |
| Luxembourg | 1053.83 (686.54 to 1535.00) | 1081.89 (725.27 to 1545.88) | -0.16 (-0.23 to -0.08) | 716.07 (295.56 to 1568.01) | 734.13 (315.62 to 1553.43) | 0.09 (0.07 to 0.11) |
| Madagascar | 856.98 (594.85 to 1192.69) | 908.84 (637.56 to 1248.35) | 0.17 (0.15 to 0.19) | 509.38 (225.92 to 1059.40) | 532.11 (243.00 to 1094.35) | 0.18 (0.17 to 0.19) |
| Malawi | 891.41 (606.51 to 1236.50) | 929.30 (645.48 to 1279.18) | 0.17 (0.15 to 0.19) | 524.95 (233.55 to 1087.71) | 567.85 (260.62 to 1160.88) | 0.29 (0.27 to 0.31) |
| Malaysia | 667.44 (447.86 to 951.56) | 734.83 (501.41 to 1041.19) | 0.35 (0.33 to 0.36) | 439.23 (200.14 to 901.78) | 488.21 (227.07 to 991.22) | 0.39 (0.38 to 0.40) |
| Maldives | 622.02 (283.37 to 1132.50) | 703.51 (405.69 to 1113.38) | 0.49 (0.45 to 0.52) | 391.85 (91.34 to 1037.29) | 471.12 (167.35 to 1069.50) | 0.74 (0.69 to 0.79) |
| Mali | 876.86 (599.49 to 1229.21) | 914.39 (641.94 to 1261.91) | 0.12 (0.09 to 0.14) | 528.74 (234.01 to 1093.67) | 569.03 (262.58 to 1162.24) | 0.27 (0.26 to 0.28) |
| Malta | 1062.18 (675.93 to 1541.66) | 1099.23 (739.91 to 1552.62) | -0.02 (-0.08 to 0.03) | 684.09 (272.55 to 1506.86) | 738.79 (318.67 to 1543.28) | 0.19 (0.14 to 0.25) |
| Marshall Islands | 785.90 (222.37 to 1828.62) | 841.42 (325.95 to 1682.05) | 0.20 (0.18 to 0.21) | 530.85 (57.13 to 1802.24) | 569.67 (102.48 to 1664.55) | 0.16 (0.13 to 0.19) |
| Mauritania | 911.29 (596.96 to 1324.50) | 953.07 (644.47 to 1346.35) | 0.11 (0.08 to 0.13) | 587.00 (246.21 to 1261.04) | 639.69 (282.45 to 1360.35) | 0.24 (0.22 to 0.26) |
| Mauritius | 687.16 (423.45 to 1051.82) | 741.57 (484.22 to 1084.29) | 0.25 (0.24 to 0.26) | 446.54 (177.78 to 982.37) | 493.58 (215.07 to 1025.87) | 0.35 (0.33 to 0.38) |
| Mexico | 1087.02 (783.16 to 1481.34) | 1120.82 (807.05 to 1525.52) | 0.09 (0.08 to 0.11) | 690.62 (327.69 to 1399.87) | 745.32 (357.57 to 1500.45) | 0.25 (0.24 to 0.26) |
| Micronesia (Federated States of) | 809.33 (358.78 to 1493.01) | 877.59 (414.43 to 1563.73) | 0.26 (0.23 to 0.28) | 565.98 (134.65 to 1497.77) | 620.76 (167.66 to 1578.87) | 0.29 (0.22 to 0.36) |
| Monaco | 1027.45 (555.90 to 1694.39) | 1090.99 (614.30 to 1742.07) | 0.10 (0.04 to 0.16) | 740.19 (228.37 to 1823.39) | 770.82 (255.54 to 1836.85) | 0.09 (0.07 to 0.11) |
| Mongolia | 744.44 (486.40 to 1068.10) | 785.70 (522.67 to 1126.56) | 0.11 (0.07 to 0.15) | 413.20 (167.20 to 893.15) | 436.50 (183.12 to 921.35) | 0.09 (0.01 to 0.16) |
| Montenegro | 840.51 (534.07 to 1245.06) | 895.72 (590.04 to 1280.89) | 0.12 (0.09 to 0.16) | 516.56 (207.50 to 1133.83) | 534.05 (225.07 to 1157.35) | 0.19 (0.16 to 0.22) |
| Morocco | 866.44 (597.99 to 1195.85) | 946.32 (674.61 to 1301.71) | 0.25 (0.24 to 0.26) | 528.72 (243.35 to 1083.64) | 585.67 (276.84 to 1186.90) | 0.37 (0.35 to 0.38) |
| Mozambique | 871.64 (608.77 to 1208.29) | 925.04 (653.73 to 1277.30) | 0.22 (0.19 to 0.24) | 510.45 (227.54 to 1065.28) | 549.45 (249.81 to 1116.49) | 0.28 (0.26 to 0.29) |
| Myanmar | 651.68 (443.90 to 919.09) | 713.27 (491.59 to 995.45) | 0.33 (0.32 to 0.34) | 408.50 (189.30 to 820.52) | 457.75 (215.22 to 921.32) | 0.45 (0.42 to 0.48) |
| Namibia | 922.83 (596.59 to 1344.78) | 951.66 (640.98 to 1353.05) | 0.08 (0.06 to 0.10) | 548.20 (219.50 to 1221.53) | 592.49 (253.17 to 1262.65) | 0.23 (0.21 to 0.25) |
| Nauru | 796.20 (51.72 to 3035.93) | 900.53 (109.29 to 2913.91) | 0.38 (0.36 to 0.41) | 550.80 (3.15 to 3025.46) | 637.61 (13.61 to 2934.17) | 0.42 (0.39 to 0.45) |
| Nepal | 869.13 (607.82 to 1207.83) | 942.50 (661.96 to 1304.08) | 0.26 (0.24 to 0.29) | 490.44 (220.55 to 1007.56) | 557.85 (258.42 to 1130.19) | 0.45 (0.43 to 0.47) |
| Netherlands | 1068.64 (756.05 to 1463.07) | 1124.53 (800.08 to 1524.76) | 0.04 (-0.01 to 0.08) | 733.07 (342.54 to 1498.44) | 763.27 (362.65 to 1559.04) | 0.08 (0.06 to 0.11) |
| New Zealand | 1040.52 (715.62 to 1449.41) | 1107.07 (774.14 to 1516.71) | 0.09 (0.04 to 0.13) | 718.83 (328.95 to 1479.36) | 783.64 (360.64 to 1620.53) | 0.24 (0.21 to 0.27) |
| Nicaragua | 1029.03 (691.53 to 1449.21) | 1078.65 (744.14 to 1486.71) | 0.16 (0.15 to 0.18) | 651.77 (284.01 to 1363.88) | 714.01 (325.30 to 1468.89) | 0.30 (0.28 to 0.32) |
| Niger | 874.01 (593.12 to 1231.76) | 907.15 (629.60 to 1249.31) | 0.13 (0.12 to 0.14) | 529.17 (233.65 to 1112.80) | 563.25 (254.83 to 1147.10) | 0.19 (0.18 to 0.20) |
| Nigeria | 900.41 (639.43 to 1231.44) | 942.30 (674.06 to 1285.95) | 0.12 (0.11 to 0.14) | 566.65 (268.89 to 1144.03) | 610.87 (290.80 to 1230.51) | 0.25 (0.20 to 0.30) |
| Niue | 809.99 (22.37 to 3754.58) | 889.46 (24.07 to 4157.71) | 0.33 (0.31 to 0.36) | 606.14 (0.86 to 3907.68) | 671.06 (1.01 to 4276.31) | 0.37 (0.35 to 0.40) |
| North Macedonia | 833.87 (556.97 to 1199.47) | 895.63 (616.25 to 1236.36) | 0.14 (0.09 to 0.19) | 488.50 (209.94 to 1034.69) | 520.71 (232.70 to 1087.82) | 0.26 (0.24 to 0.28) |
| Northern Mariana Islands | 813.90 (184.89 to 2089.93) | 876.22 (389.86 to 1617.86) | 0.21 (0.18 to 0.23) | 593.55 (46.40 to 2161.84) | 643.84 (153.48 to 1701.78) | 0.20 (0.13 to 0.27) |
| Norway | 1023.02 (718.82 to 1403.66) | 1088.23 (774.87 to 1493.84) | 0.12 (0.04 to 0.19) | 676.01 (312.29 to 1400.74) | 716.10 (334.45 to 1479.45) | 0.14 (0.11 to 0.17) |
| Oman | 869.86 (554.49 to 1288.94) | 998.98 (666.14 to 1435.44) | 0.48 (0.46 to 0.50) | 516.99 (204.63 to 1148.81) | 624.79 (271.02 to 1311.41) | 0.66 (0.64 to 0.67) |
| Pakistan | 792.09 (558.75 to 1090.24) | 888.53 (630.06 to 1228.93) | 0.44 (0.40 to 0.48) | 437.13 (202.81 to 890.72) | 496.15 (231.54 to 994.64) | 0.49 (0.44 to 0.53) |
| Palau | 824.89 (173.99 to 2178.03) | 888.66 (302.59 to 1877.92) | 0.23 (0.22 to 0.25) | 588.72 (39.96 to 2200.92) | 643.59 (101.50 to 1968.78) | 0.26 (0.20 to 0.31) |
| Palestine | 893.33 (579.24 to 1302.83) | 956.15 (649.91 to 1348.19) | 0.15 (0.12 to 0.18) | 548.41 (226.04 to 1187.84) | 584.03 (254.19 to 1226.50) | 0.19 (0.18 to 0.20) |
| Panama | 1002.67 (677.23 to 1426.30) | 1049.30 (728.66 to 1454.58) | 0.15 (0.14 to 0.17) | 634.65 (273.34 to 1352.99) | 715.06 (325.04 to 1477.89) | 0.35 (0.34 to 0.37) |
| Papua New Guinea | 761.69 (499.65 to 1104.22) | 799.96 (540.46 to 1139.82) | 0.11 (0.09 to 0.13) | 502.34 (211.65 to 1060.83) | 534.47 (237.98 to 1101.80) | 0.16 (0.13 to 0.19) |
| Paraguay | 1029.17 (695.44 to 1445.44) | 1075.43 (748.15 to 1495.05) | 0.13 (0.10 to 0.16) | 666.74 (297.54 to 1404.39) | 712.36 (320.87 to 1471.81) | 0.22 (0.21 to 0.23) |
| Peru | 1038.17 (736.84 to 1422.46) | 1091.03 (774.42 to 1491.11) | 0.11 (0.08 to 0.13) | 667.17 (306.25 to 1387.44) | 745.38 (356.32 to 1512.12) | 0.40 (0.38 to 0.42) |
| Philippines | 622.52 (432.29 to 864.78) | 704.03 (495.57 to 972.49) | 0.43 (0.40 to 0.46) | 378.71 (175.91 to 767.87) | 437.58 (206.93 to 882.40) | 0.50 (0.46 to 0.54) |
| Poland | 843.69 (600.93 to 1153.39) | 886.49 (634.12 to 1201.31) | 0.05 (0.01 to 0.10) | 499.03 (234.41 to 1012.83) | 543.76 (258.32 to 1097.65) | 0.32 (0.29 to 0.34) |
| Portugal | 1064.74 (753.22 to 1425.81) | 1064.69 (752.60 to 1455.63) | -0.23 (-0.30 to -0.16) | 686.65 (321.18 to 1406.94) | 747.14 (354.05 to 1544.83) | 0.24 (0.20 to 0.27) |
| Puerto Rico | 1089.60 (746.84 to 1537.00) | 1070.09 (744.72 to 1481.27) | 0.02 (-0.01 to 0.04) | 747.17 (336.63 to 1543.27) | 804.95 (372.63 to 1655.64) | 0.27 (0.25 to 0.29) |
| Qatar | 967.22 (473.31 to 1686.86) | 1057.90 (671.87 to 1568.12) | 0.22 (0.20 to 0.25) | 582.97 (148.07 to 1524.93) | 640.16 (254.62 to 1386.52) | 0.29 (0.26 to 0.32) |
| Republic of Korea | 1438.16 (1035.44 to 1946.02) | 1362.20 (973.66 to 1835.65) | -0.24 (-0.39 to -0.09) | 1061.46 (502.49 to 2124.94) | 1066.33 (512.96 to 2153.77) | 0.22 (0.07 to 0.37) |
| Republic of Moldova | 889.07 (614.62 to 1237.41) | 915.78 (630.12 to 1272.30) | 0.02 (-0.01 to 0.06) | 517.67 (229.31 to 1073.43) | 570.56 (262.47 to 1164.84) | 0.40 (0.36 to 0.44) |
| Romania | 842.71 (594.06 to 1157.64) | 862.46 (603.24 to 1189.51) | 0.00 (-0.04 to 0.04) | 495.20 (230.19 to 1021.87) | 541.64 (254.95 to 1096.57) | 0.36 (0.34 to 0.39) |
| Russian Federation | 882.66 (633.33 to 1199.04) | 934.83 (675.17 to 1269.80) | 0.06 (0.00 to 0.11) | 530.96 (253.65 to 1075.32) | 571.45 (273.62 to 1148.64) | 0.34 (0.30 to 0.38) |
| Rwanda | 888.70 (607.54 to 1233.32) | 928.10 (640.73 to 1278.20) | 0.14 (0.12 to 0.16) | 522.42 (230.30 to 1093.47) | 561.49 (253.21 to 1167.67) | 0.28 (0.26 to 0.30) |
| Saint Kitts and Nevis | 1073.18 (525.81 to 1892.07) | 1173.81 (619.93 to 1957.56) | 0.25 (0.16 to 0.34) | 701.56 (182.74 to 1825.16) | 743.72 (217.80 to 1850.76) | 0.18 (0.15 to 0.22) |
| Saint Lucia | 1058.29 (581.84 to 1710.71) | 1073.92 (653.48 to 1627.32) | 0.01 (0.00 to 0.03) | 674.93 (215.14 to 1628.92) | 732.18 (276.45 to 1646.23) | 0.25 (0.22 to 0.28) |
| Saint Vincent and the Grenadines | 1042.95 (555.60 to 1731.04) | 1085.39 (634.46 to 1686.52) | 0.12 (0.10 to 0.15) | 668.86 (200.05 to 1659.76) | 718.77 (251.55 to 1680.53) | 0.28 (0.25 to 0.30) |
| Samoa | 813.40 (403.36 to 1418.67) | 867.90 (470.28 to 1417.61) | 0.19 (0.16 to 0.21) | 586.23 (169.79 to 1459.73) | 630.08 (214.41 to 1496.57) | 0.22 (0.17 to 0.26) |
| San Marino | 1043.00 (489.78 to 1851.55) | 1044.44 (560.45 to 1716.36) | -0.16 (-0.22 to -0.11) | 712.10 (175.42 to 1915.43) | 743.33 (230.93 to 1826.02) | 0.11 (0.08 to 0.13) |
| Sao Tome and Principe | 877.25 (430.91 to 1515.17) | 944.44 (503.70 to 1555.60) | 0.16 (0.13 to 0.18) | 574.10 (152.32 to 1513.07) | 624.23 (190.73 to 1519.00) | 0.30 (0.29 to 0.31) |
| Saudi Arabia | 895.13 (616.10 to 1250.77) | 1030.66 (722.50 to 1428.20) | 0.40 (0.36 to 0.43) | 543.68 (245.01 to 1120.52) | 620.77 (289.03 to 1275.47) | 0.43 (0.39 to 0.47) |
| Senegal | 888.38 (602.98 to 1248.95) | 924.34 (642.27 to 1282.92) | 0.08 (0.07 to 0.10) | 559.52 (245.57 to 1169.93) | 598.16 (272.99 to 1213.09) | 0.19 (0.17 to 0.20) |
| Serbia | 862.97 (609.05 to 1186.00) | 871.86 (615.62 to 1194.34) | -0.07 (-0.11 to -0.03) | 493.41 (224.59 to 1014.78) | 536.24 (245.53 to 1088.23) | 0.32 (0.30 to 0.34) |
| Seychelles | 696.13 (310.81 to 1270.46) | 739.15 (378.16 to 1276.39) | 0.20 (0.18 to 0.22) | 472.94 (115.94 to 1250.92) | 497.53 (150.75 to 1218.60) | 0.19 (0.16 to 0.22) |
| Sierra Leone | 858.81 (576.28 to 1211.07) | 908.78 (613.71 to 1271.79) | 0.18 (0.16 to 0.20) | 542.34 (238.18 to 1146.53) | 582.89 (263.23 to 1194.93) | 0.22 (0.21 to 0.24) |
| Singapore | 1316.59 (911.27 to 1829.25) | 1348.10 (956.32 to 1844.38) | -0.05 (-0.10 to -0.00) | 962.49 (429.64 to 2002.64) | 1006.14 (471.36 to 2056.01) | 0.15 (0.12 to 0.19) |
| Slovakia | 847.05 (587.07 to 1175.76) | 884.53 (622.05 to 1223.56) | 0.08 (0.04 to 0.11) | 502.63 (227.98 to 1030.36) | 538.33 (247.85 to 1113.83) | 0.20 (0.19 to 0.22) |
| Slovenia | 833.73 (556.48 to 1174.11) | 843.15 (581.42 to 1189.17) | -0.03 (-0.07 to 0.01) | 504.99 (222.96 to 1077.11) | 538.33 (242.99 to 1117.01) | 0.25 (0.23 to 0.27) |
| Solomon Islands | 754.22 (392.93 to 1258.98) | 832.98 (493.81 to 1290.08) | 0.33 (0.30 to 0.35) | 506.61 (154.29 to 1241.63) | 575.58 (211.07 to 1294.09) | 0.39 (0.34 to 0.44) |
| Somalia | 871.40 (586.96 to 1236.52) | 931.02 (647.52 to 1288.04) | 0.20 (0.17 to 0.22) | 515.74 (219.34 to 1108.02) | 530.28 (236.80 to 1098.18) | 0.12 (0.11 to 0.14) |
| South Africa | 930.86 (653.90 to 1284.83) | 988.38 (710.47 to 1348.43) | 0.18 (0.16 to 0.20) | 597.31 (279.81 to 1215.71) | 633.87 (300.86 to 1282.02) | 0.22 (0.21 to 0.23) |
| South Sudan | 864.28 (585.90 to 1217.52) | 906.96 (623.74 to 1275.27) | 0.19 (0.14 to 0.23) | 510.82 (222.22 to 1063.59) | 541.10 (239.35 to 1118.24) | 0.21 (0.20 to 0.21) |
| Spain | 1058.44 (764.47 to 1432.74) | 1044.52 (745.46 to 1408.89) | -0.25 (-0.32 to -0.19) | 706.43 (333.24 to 1452.65) | 748.06 (358.91 to 1526.09) | 0.11 (0.07 to 0.15) |
| Sri Lanka | 652.72 (442.79 to 926.83) | 722.00 (500.96 to 1010.66) | 0.35 (0.34 to 0.36) | 415.51 (188.70 to 841.64) | 461.53 (214.72 to 925.79) | 0.39 (0.37 to 0.40) |
| Sudan | 855.60 (591.50 to 1180.38) | 932.38 (649.17 to 1281.06) | 0.29 (0.24 to 0.33) | 495.53 (223.95 to 1011.25) | 571.75 (265.51 to 1156.91) | 0.47 (0.44 to 0.50) |
| Suriname | 1035.18 (638.80 to 1580.68) | 1094.88 (713.20 to 1599.70) | 0.14 (0.12 to 0.17) | 672.96 (245.80 to 1517.28) | 736.45 (310.20 to 1563.58) | 0.34 (0.33 to 0.36) |
| Sweden | 913.27 (646.25 to 1253.99) | 957.66 (668.82 to 1317.13) | 0.04 (-0.08 to 0.17) | 565.94 (260.84 to 1179.90) | 600.99 (276.81 to 1231.61) | 0.05 (-0.09 to 0.19) |
| Switzerland | 1008.16 (711.88 to 1372.76) | 1043.10 (734.29 to 1418.42) | -0.02 (-0.06 to 0.02) | 693.18 (322.93 to 1435.45) | 716.26 (337.89 to 1480.50) | 0.08 (0.07 to 0.10) |
| Syrian Arab Republic | 889.61 (607.05 to 1246.59) | 971.59 (678.27 to 1352.10) | 0.20 (0.17 to 0.24) | 540.53 (245.04 to 1130.79) | 593.01 (277.17 to 1188.01) | 0.31 (0.29 to 0.34) |
| Taiwan (Province of China) | 788.71 (534.23 to 1119.50) | 874.46 (605.46 to 1226.88) | 0.42 (0.38 to 0.45) | 610.67 (284.03 to 1236.58) | 715.60 (337.97 to 1432.25) | 0.64 (0.59 to 0.68) |
| Tajikistan | 708.61 (474.60 to 1006.71) | 746.95 (519.69 to 1047.71) | 0.08 (0.05 to 0.11) | 402.77 (171.61 to 867.09) | 404.71 (178.73 to 850.12) | 0.09 (0.06 to 0.12) |
| Thailand | 679.68 (466.58 to 961.40) | 753.53 (518.55 to 1053.74) | 0.37 (0.34 to 0.40) | 449.09 (210.47 to 918.80) | 530.40 (250.20 to 1080.50) | 0.62 (0.59 to 0.65) |
| Timor-Leste | 628.33 (343.19 to 1023.82) | 687.13 (434.25 to 1022.92) | 0.30 (0.29 to 0.31) | 395.89 (128.62 to 953.66) | 424.78 (173.28 to 920.66) | 0.29 (0.26 to 0.33) |
| Togo | 877.30 (573.96 to 1268.62) | 944.00 (647.61 to 1318.22) | 0.24 (0.23 to 0.26) | 546.43 (228.99 to 1160.56) | 596.27 (268.09 to 1243.20) | 0.28 (0.26 to 0.29) |
| Tokelau | 791.79 (2.36 to 5233.37) | 862.09 (8.02 to 4828.60) | 0.30 (0.27 to 0.32) | 558.66 (0.00 to 5282.80) | 636.16 (0.10 to 4986.57) | 0.48 (0.44 to 0.52) |
| Tonga | 802.73 (361.14 to 1465.41) | 861.45 (434.26 to 1486.80) | 0.20 (0.17 to 0.24) | 577.45 (142.02 to 1530.60) | 645.26 (192.49 to 1618.83) | 0.30 (0.24 to 0.37) |
| Trinidad and Tobago | 1074.76 (708.37 to 1535.38) | 1118.66 (763.24 to 1561.64) | 0.20 (0.17 to 0.23) | 698.44 (296.56 to 1470.20) | 744.73 (331.60 to 1568.28) | 0.25 (0.23 to 0.27) |
| Tunisia | 898.12 (621.49 to 1246.87) | 949.88 (665.79 to 1307.27) | 0.14 (0.13 to 0.16) | 538.81 (242.57 to 1119.25) | 595.46 (278.41 to 1198.98) | 0.36 (0.34 to 0.37) |
| Turkey | 924.58 (645.26 to 1276.45) | 991.57 (710.46 to 1356.41) | 0.15 (0.12 to 0.18) | 584.88 (273.48 to 1194.14) | 649.13 (309.32 to 1309.87) | 0.39 (0.34 to 0.44) |
| Turkmenistan | 760.12 (506.24 to 1085.82) | 803.14 (547.87 to 1132.26) | 0.10 (0.06 to 0.13) | 428.02 (179.83 to 914.66) | 457.59 (200.02 to 944.72) | 0.31 (0.28 to 0.34) |
| Tuvalu | 811.65 (121.58 to 2436.98) | 873.48 (211.37 to 2188.68) | 0.24 (0.22 to 0.26) | 554.87 (16.91 to 2433.05) | 621.76 (53.33 to 2251.57) | 0.38 (0.33 to 0.42) |
| Uganda | 861.38 (597.55 to 1193.27) | 907.89 (635.85 to 1254.55) | 0.20 (0.19 to 0.21) | 516.25 (235.29 to 1075.25) | 559.38 (260.96 to 1135.62) | 0.31 (0.29 to 0.33) |
| Ukraine | 890.84 (630.04 to 1220.37) | 909.02 (645.99 to 1249.44) | -0.03 (-0.07 to 0.01) | 537.76 (255.97 to 1100.92) | 560.83 (264.91 to 1113.00) | 0.22 (0.19 to 0.25) |
| United Arab Emirates | 924.26 (550.59 to 1417.32) | 1067.70 (726.62 to 1487.83) | 0.50 (0.47 to 0.52) | 559.69 (205.20 to 1269.39) | 593.99 (258.67 to 1226.93) | 0.24 (0.20 to 0.28) |
| United Kingdom | 1104.63 (797.67 to 1486.01) | 1140.82 (824.03 to 1537.31) | 0.02 (-0.02 to 0.05) | 757.92 (361.87 to 1549.57) | 794.93 (381.05 to 1614.93) | 0.17 (0.10 to 0.24) |
| United Republic of Tanzania | 886.36 (618.91 to 1221.07) | 915.97 (645.72 to 1257.96) | 0.12 (0.10 to 0.13) | 532.83 (242.71 to 1093.24) | 575.99 (267.00 to 1182.94) | 0.29 (0.28 to 0.31) |
| United States of America | 997.38 (707.07 to 1368.62) | 1062.23 (756.51 to 1448.21) | 0.03 (-0.17 to 0.23) | 772.35 (370.67 to 1551.48) | 791.33 (381.54 to 1582.53) | -0.31 (-0.52 to -0.09) |
| United States Virgin Islands | 1121.63 (598.27 to 1849.58) | 1129.75 (686.45 to 1717.12) | 0.12 (0.07 to 0.18) | 741.35 (224.25 to 1801.27) | 795.26 (301.54 to 1782.73) | 0.21 (0.20 to 0.22) |
| Uruguay | 1001.93 (699.75 to 1397.40) | 1055.85 (737.85 to 1453.99) | -0.04 (-0.09 to 0.02) | 706.33 (322.61 to 1466.15) | 784.26 (362.24 to 1599.08) | 0.33 (0.29 to 0.37) |
| Uzbekistan | 726.24 (503.57 to 1010.17) | 799.90 (563.93 to 1097.18) | 0.22 (0.19 to 0.26) | 425.31 (194.73 to 887.23) | 444.26 (206.12 to 901.15) | 0.24 (0.21 to 0.28) |
| Vanuatu | 771.84 (344.60 to 1410.11) | 847.86 (468.52 to 1370.09) | 0.30 (0.29 to 0.30) | 526.00 (124.95 to 1396.91) | 587.30 (195.10 to 1379.33) | 0.36 (0.34 to 0.37) |
| Venezuela (Bolivarian Republic of) | 1049.23 (734.67 to 1437.50) | 1101.20 (782.58 to 1491.85) | 0.15 (0.10 to 0.19) | 682.44 (311.23 to 1408.60) | 727.08 (343.89 to 1473.86) | 0.22 (0.21 to 0.23) |
| Viet Nam | 653.51 (451.23 to 920.66) | 701.81 (492.49 to 983.77) | 0.27 (0.26 to 0.29) | 417.39 (195.13 to 842.92) | 452.52 (212.69 to 914.36) | 0.34 (0.32 to 0.37) |
| Yemen | 856.41 (593.48 to 1185.26) | 899.36 (635.79 to 1231.64) | 0.17 (0.16 to 0.19) | 484.04 (217.71 to 1005.80) | 527.45 (241.60 to 1077.24) | 0.30 (0.29 to 0.31) |
| Zambia | 877.24 (598.53 to 1241.87) | 929.16 (641.07 to 1287.34) | 0.18 (0.16 to 0.20) | 531.13 (231.63 to 1127.51) | 569.65 (255.21 to 1176.51) | 0.27 (0.24 to 0.29) |
| Zimbabwe | 913.96 (631.82 to 1280.17) | 960.06 (670.02 to 1316.70) | 0.13 (0.09 to 0.18) | 555.34 (248.53 to 1150.57) | 570.55 (259.03 to 1159.42) | 0.04 (0.01 to 0.07) |

**Abbreviations: DALY=disability-adjusted life-years. EAPC=estimated annual percentage change. CI=confidence interval**

**Table S6. Age-standardised incidence rates and DALY rates in 1990 and 2021, and estimated annual percentage changes for other sites osteoarthritis in older adults, by country.**

| **Countries** | **Age-standardised incidence rate, 1990** | **Age-standardised incidence rate, 2021** | **EAPC of incidence rate No. (95% CI)** | **Age-standardised DALYs rate, 1990** | **Age-standardised DALYs rate, 2021** | **EAPC of DALYs rate No. (95% CI)** |
| --- | --- | --- | --- | --- | --- | --- |
| Afghanistan | 124.46 (64.70 to 182.17) | 124.50 (66.12 to 181.60) | 0.03 (0.02 to 0.05) | 116.52 (47.21 to 261.36) | 124.19 (51.88 to 281.86) | 0.30 (0.25 to 0.36) |
| Albania | 124.68 (60.15 to 192.93) | 125.14 (64.50 to 184.92) | 0.01 (-0.00 to 0.01) | 124.95 (46.29 to 289.76) | 133.76 (54.24 to 303.34) | 0.28 (0.26 to 0.30) |
| Algeria | 126.70 (68.43 to 180.41) | 127.89 (71.48 to 178.00) | 0.03 (0.02 to 0.04) | 127.05 (53.30 to 283.01) | 140.00 (61.78 to 306.36) | 0.36 (0.33 to 0.38) |
| American Samoa | 129.28 (3.31 to 533.47) | 128.96 (14.48 to 370.31) | -0.01 (-0.02 to -0.00) | 135.81 (1.40 to 705.00) | 142.56 (10.58 to 538.04) | 0.11 (0.02 to 0.20) |
| Andorra | 125.03 (17.89 to 327.82) | 125.79 (34.06 to 257.25) | 0.01 (0.00 to 0.01) | 122.12 (11.33 to 442.43) | 136.04 (28.01 to 392.09) | 0.35 (0.27 to 0.43) |
| Angola | 124.15 (61.92 to 188.37) | 125.92 (66.91 to 182.25) | 0.04 (0.03 to 0.04) | 111.33 (42.08 to 258.72) | 118.33 (49.15 to 263.83) | 0.25 (0.22 to 0.27) |
| Antigua and Barbuda | 125.32 (18.80 to 326.67) | 126.40 (28.44 to 282.18) | 0.03 (0.02 to 0.03) | 133.52 (13.54 to 471.76) | 132.86 (21.39 to 416.69) | -0.05 (-0.07 to -0.03) |
| Argentina | 124.31 (67.92 to 176.38) | 125.70 (69.40 to 175.68) | 0.03 (0.02 to 0.04) | 120.41 (52.80 to 261.48) | 133.97 (60.03 to 283.14) | 0.35 (0.29 to 0.40) |
| Armenia | 124.64 (60.42 to 190.73) | 125.98 (65.07 to 184.81) | 0.00 (-0.01 to 0.01) | 127.61 (48.62 to 299.79) | 133.01 (54.14 to 296.76) | 0.42 (0.33 to 0.51) |
| Australia | 125.95 (68.87 to 178.47) | 127.12 (71.06 to 176.10) | 0.02 (0.02 to 0.03) | 125.59 (54.28 to 272.13) | 140.90 (63.26 to 303.15) | 0.33 (0.27 to 0.39) |
| Austria | 124.80 (66.14 to 178.54) | 124.59 (66.67 to 177.76) | 0.01 (0.00 to 0.02) | 126.10 (54.85 to 276.21) | 136.39 (59.85 to 297.19) | 0.25 (0.23 to 0.27) |
| Azerbaijan | 124.88 (63.41 to 185.14) | 127.00 (68.54 to 182.73) | 0.02 (0.01 to 0.03) | 129.38 (52.16 to 294.02) | 131.71 (54.16 to 296.19) | 0.26 (0.18 to 0.33) |
| Bahamas | 126.56 (33.14 to 262.71) | 126.76 (47.36 to 223.24) | 0.00 (-0.01 to 0.00) | 135.83 (26.53 to 400.01) | 136.21 (38.80 to 349.98) | 0.02 (0.00 to 0.03) |
| Bahrain | 129.65 (33.43 to 273.05) | 130.60 (55.54 to 212.39) | 0.02 (0.01 to 0.03) | 134.01 (23.61 to 407.79) | 138.08 (43.92 to 349.95) | 0.12 (0.10 to 0.14) |
| Bangladesh | 124.56 (69.48 to 174.17) | 124.20 (69.79 to 174.59) | 0.00 (-0.00 to 0.00) | 118.54 (52.00 to 256.86) | 120.60 (53.86 to 260.93) | 0.09 (0.05 to 0.14) |
| Barbados | 126.44 (45.83 to 226.61) | 126.55 (51.26 to 213.44) | 0.01 (0.00 to 0.01) | 137.66 (38.37 to 360.25) | 138.95 (43.02 to 349.28) | 0.02 (-0.01 to 0.04) |
| Belarus | 125.36 (67.68 to 178.70) | 126.25 (69.24 to 178.58) | 0.00 (-0.00 to 0.01) | 129.99 (55.16 to 286.76) | 135.54 (58.63 to 294.19) | 0.31 (0.25 to 0.37) |
| Belgium | 124.55 (66.23 to 178.04) | 124.96 (67.78 to 176.97) | 0.00 (-0.00 to 0.01) | 124.96 (54.30 to 274.37) | 134.95 (59.10 to 290.23) | 0.28 (0.26 to 0.31) |
| Belize | 126.13 (26.27 to 289.10) | 127.31 (42.91 to 234.99) | 0.01 (0.00 to 0.02) | 135.97 (20.41 to 434.85) | 140.82 (36.01 to 380.11) | 0.12 (0.06 to 0.18) |
| Benin | 125.43 (60.30 to 193.63) | 126.93 (65.25 to 186.00) | 0.05 (0.04 to 0.06) | 121.04 (45.22 to 284.63) | 128.86 (51.93 to 288.60) | 0.21 (0.19 to 0.23) |
| Bermuda | 128.13 (20.95 to 324.39) | 127.10 (34.79 to 259.84) | -0.04 (-0.04 to -0.03) | 142.51 (15.83 to 492.96) | 146.45 (31.26 to 417.62) | 0.07 (0.06 to 0.09) |
| Bhutan | 126.00 (37.72 to 249.91) | 126.23 (51.92 to 212.85) | 0.01 (0.00 to 0.01) | 116.42 (23.51 to 339.10) | 129.27 (40.22 to 320.13) | 0.39 (0.36 to 0.43) |
| Bolivia (Plurinational State of) | 124.36 (60.99 to 188.99) | 125.99 (67.28 to 181.28) | 0.03 (0.02 to 0.03) | 119.44 (46.11 to 273.00) | 128.57 (53.85 to 286.33) | 0.29 (0.26 to 0.31) |
| Bosnia and Herzegovina | 125.12 (63.46 to 187.19) | 125.58 (65.64 to 182.21) | 0.00 (-0.01 to 0.01) | 123.20 (48.36 to 279.70) | 132.67 (55.37 to 296.24) | 0.36 (0.32 to 0.41) |
| Botswana | 126.18 (49.81 to 216.43) | 127.78 (59.38 to 199.15) | 0.03 (0.02 to 0.04) | 119.77 (34.67 to 309.10) | 132.04 (47.31 to 320.32) | 0.34 (0.32 to 0.36) |
| Brazil | 127.31 (72.15 to 174.09) | 127.04 (73.51 to 173.37) | -0.01 (-0.01 to -0.01) | 129.04 (57.75 to 281.30) | 139.67 (63.86 to 298.90) | 0.28 (0.25 to 0.30) |
| Brunei Darussalam | 124.52 (23.40 to 296.71) | 126.23 (43.38 to 233.57) | 0.04 (0.04 to 0.05) | 115.18 (14.20 to 390.05) | 118.81 (29.76 to 326.19) | 0.11 (0.07 to 0.14) |
| Bulgaria | 127.24 (70.49 to 179.45) | 126.04 (69.02 to 179.49) | -0.02 (-0.03 to -0.01) | 130.05 (55.52 to 289.71) | 138.17 (59.89 to 299.69) | 0.21 (0.19 to 0.23) |
| Burkina Faso | 124.68 (63.18 to 185.67) | 125.87 (66.61 to 182.47) | 0.04 (0.04 to 0.05) | 115.23 (44.91 to 262.58) | 123.42 (51.99 to 279.63) | 0.21 (0.19 to 0.22) |
| Burundi | 124.34 (59.75 to 189.38) | 124.18 (61.56 to 186.39) | 0.00 (-0.01 to 0.00) | 116.28 (42.64 to 272.06) | 115.71 (45.41 to 260.40) | 0.03 (0.00 to 0.05) |
| Cabo Verde | 124.77 (40.00 to 237.25) | 127.00 (47.75 to 222.30) | 0.04 (0.03 to 0.05) | 128.90 (31.52 to 352.62) | 132.75 (38.16 to 343.62) | 0.22 (0.16 to 0.27) |
| Cambodia | 124.14 (62.65 to 185.62) | 123.90 (65.56 to 179.39) | 0.00 (-0.00 to 0.01) | 112.65 (44.34 to 255.16) | 115.21 (48.51 to 252.45) | 0.12 (0.10 to 0.14) |
| Cameroon | 127.86 (65.52 to 188.49) | 128.35 (68.80 to 182.21) | 0.00 (-0.00 to 0.01) | 127.31 (50.75 to 289.04) | 131.87 (56.00 to 293.94) | 0.14 (0.12 to 0.16) |
| Canada | 124.81 (68.33 to 176.38) | 125.99 (70.22 to 175.75) | 0.03 (0.02 to 0.03) | 123.15 (55.14 to 267.67) | 135.08 (60.17 to 287.70) | 0.28 (0.23 to 0.34) |
| Central African Republic | 125.35 (55.05 to 203.42) | 124.34 (57.21 to 195.65) | -0.01 (-0.02 to -0.01) | 109.15 (34.52 to 269.55) | 110.91 (39.47 to 266.61) | 0.08 (0.07 to 0.10) |
| Chad | 124.16 (61.37 to 188.09) | 124.92 (63.79 to 183.37) | 0.03 (0.02 to 0.03) | 116.67 (44.74 to 270.09) | 119.12 (48.48 to 268.91) | 0.06 (0.04 to 0.07) |
| Chile | 125.38 (66.25 to 181.13) | 126.05 (69.47 to 177.55) | 0.02 (0.01 to 0.02) | 122.85 (52.26 to 270.29) | 134.62 (59.72 to 288.65) | 0.29 (0.25 to 0.32) |
| China | 127.14 (73.31 to 173.10) | 127.38 (74.05 to 172.38) | 0.00 (0.00 to 0.01) | 122.87 (55.69 to 264.35) | 132.77 (61.30 to 284.09) | 0.28 (0.26 to 0.29) |
| Colombia | 125.57 (68.55 to 179.01) | 125.41 (70.65 to 175.48) | 0.00 (-0.01 to 0.00) | 127.25 (55.23 to 276.26) | 136.02 (60.66 to 293.62) | 0.23 (0.22 to 0.24) |
| Comoros | 125.47 (35.88 to 254.29) | 125.44 (48.38 to 218.99) | 0.00 (-0.01 to 0.00) | 115.73 (22.24 to 341.61) | 121.62 (34.57 to 312.38) | 0.20 (0.18 to 0.21) |
| Congo | 126.15 (55.52 to 203.23) | 127.01 (61.97 to 192.94) | 0.02 (0.01 to 0.03) | 116.62 (38.43 to 284.96) | 123.94 (46.70 to 291.54) | 0.23 (0.21 to 0.24) |
| Cook Islands | 128.60 (0.58 to 694.27) | 128.61 (7.07 to 442.66) | 0.00 (-0.01 to 0.00) | 137.77 (0.23 to 882.43) | 149.79 (5.73 to 636.60) | 0.21 (0.17 to 0.25) |
| Costa Rica | 125.82 (59.92 to 193.98) | 125.84 (65.94 to 183.35) | 0.00 (-0.00 to 0.01) | 131.49 (48.82 to 310.95) | 137.43 (57.66 to 304.34) | 0.15 (0.14 to 0.16) |
| Côte d'Ivoire | 126.47 (63.78 to 189.21) | 126.73 (68.67 to 181.78) | 0.00 (-0.01 to 0.00) | 117.37 (46.12 to 272.84) | 125.12 (51.70 to 275.55) | 0.19 (0.17 to 0.20) |
| Croatia | 126.33 (66.28 to 183.44) | 125.98 (67.18 to 180.49) | -0.01 (-0.02 to -0.01) | 127.52 (53.00 to 288.03) | 139.34 (59.72 to 304.97) | 0.39 (0.35 to 0.43) |
| Cuba | 126.14 (67.22 to 179.84) | 125.58 (69.19 to 177.71) | 0.00 (-0.01 to 0.01) | 131.52 (55.73 to 291.99) | 137.87 (60.92 to 302.95) | 0.17 (0.15 to 0.19) |
| Cyprus | 123.97 (52.08 to 202.48) | 125.14 (60.89 to 190.37) | 0.04 (0.03 to 0.06) | 115.30 (38.05 to 283.19) | 130.01 (50.11 to 301.83) | 0.38 (0.35 to 0.41) |
| Czechia | 126.43 (69.10 to 179.05) | 126.15 (69.74 to 177.35) | 0.01 (0.00 to 0.01) | 129.89 (56.15 to 285.90) | 139.17 (61.11 to 301.71) | 0.21 (0.19 to 0.24) |
| Democratic People's Republic of Korea | 124.78 (66.90 to 179.62) | 123.06 (67.23 to 175.87) | -0.06 (-0.06 to -0.05) | 117.38 (49.34 to 260.64) | 120.19 (52.49 to 259.18) | 0.10 (0.03 to 0.16) |
| Democratic Republic of the Congo | 126.29 (67.91 to 180.57) | 124.83 (68.52 to 177.15) | -0.05 (-0.06 to -0.03) | 113.16 (47.51 to 249.83) | 115.36 (49.68 to 252.75) | 0.07 (0.05 to 0.08) |
| Denmark | 124.26 (65.57 to 180.65) | 124.49 (66.78 to 178.26) | 0.03 (0.02 to 0.04) | 124.91 (53.24 to 277.16) | 133.83 (58.58 to 291.15) | 0.16 (0.12 to 0.20) |
| Djibouti | 124.73 (26.04 to 289.01) | 127.37 (49.91 to 219.97) | 0.06 (0.06 to 0.07) | 113.14 (14.25 to 373.77) | 125.10 (36.18 to 324.25) | 0.34 (0.32 to 0.36) |
| Dominica | 125.48 (20.08 to 317.96) | 125.96 (24.68 to 296.14) | 0.01 (0.01 to 0.02) | 129.28 (14.19 to 450.11) | 132.84 (18.26 to 435.60) | 0.07 (0.05 to 0.10) |
| Dominican Republic | 125.04 (62.37 to 187.26) | 126.33 (68.13 to 180.45) | 0.03 (0.02 to 0.03) | 127.78 (50.71 to 295.11) | 138.03 (59.49 to 305.68) | 0.26 (0.24 to 0.28) |
| Ecuador | 127.21 (66.37 to 185.33) | 127.11 (69.76 to 179.43) | 0.00 (-0.01 to 0.00) | 133.23 (54.15 to 300.21) | 139.65 (60.54 to 307.71) | 0.13 (0.11 to 0.15) |
| Egypt | 127.81 (70.73 to 178.53) | 129.08 (74.01 to 177.77) | 0.02 (0.01 to 0.03) | 129.70 (56.46 to 285.80) | 136.21 (60.06 to 295.80) | 0.10 (0.06 to 0.14) |
| El Salvador | 125.05 (61.04 to 187.69) | 125.45 (65.13 to 181.57) | 0.00 (-0.01 to 0.01) | 128.94 (51.57 to 294.52) | 138.25 (57.91 to 307.52) | 0.22 (0.20 to 0.25) |
| Equatorial Guinea | 124.51 (33.27 to 257.39) | 127.73 (47.88 to 224.23) | 0.09 (0.08 to 0.10) | 110.89 (19.62 to 336.95) | 127.21 (35.51 to 334.68) | 0.58 (0.53 to 0.63) |
| Eritrea | 124.61 (51.42 to 208.11) | 124.75 (60.49 to 191.38) | 0.00 (-0.01 to 0.01) | 108.00 (32.67 to 269.77) | 114.53 (41.89 to 263.94) | 0.20 (0.18 to 0.21) |
| Estonia | 125.63 (60.86 to 192.22) | 126.17 (63.11 to 187.66) | 0.00 (-0.01 to 0.01) | 131.79 (50.06 to 300.61) | 142.75 (57.02 to 319.04) | 0.38 (0.33 to 0.42) |
| Eswatini | 126.89 (40.88 to 240.15) | 127.92 (51.08 to 218.27) | 0.01 (-0.01 to 0.03) | 128.43 (30.52 to 356.42) | 130.29 (38.51 to 331.26) | -0.04 (-0.10 to 0.01) |
| Ethiopia | 126.71 (69.69 to 179.27) | 126.53 (70.27 to 175.79) | -0.01 (-0.02 to -0.01) | 116.35 (49.92 to 256.01) | 126.29 (56.18 to 275.06) | 0.29 (0.27 to 0.31) |
| Fiji | 127.09 (44.13 to 234.95) | 128.61 (54.93 to 211.33) | 0.02 (0.02 to 0.03) | 127.47 (32.00 to 351.05) | 133.35 (43.44 to 330.20) | 0.10 (0.08 to 0.12) |
| Finland | 124.99 (64.53 to 182.92) | 125.63 (67.17 to 179.16) | 0.02 (0.02 to 0.03) | 124.53 (52.50 to 277.67) | 135.37 (59.70 to 294.03) | 0.25 (0.22 to 0.27) |
| France | 124.45 (69.87 to 174.35) | 124.46 (70.28 to 173.64) | -0.01 (-0.01 to -0.00) | 124.92 (56.35 to 267.60) | 134.53 (61.11 to 286.98) | 0.28 (0.24 to 0.31) |
| Gabon | 126.69 (51.04 to 214.87) | 128.60 (56.95 to 206.25) | 0.03 (0.02 to 0.04) | 122.63 (36.93 to 313.81) | 129.97 (42.37 to 313.79) | 0.22 (0.20 to 0.25) |
| Gambia | 125.77 (43.76 to 230.51) | 126.41 (54.88 to 204.61) | 0.02 (0.02 to 0.02) | 119.88 (30.15 to 328.77) | 126.86 (43.22 to 308.14) | 0.18 (0.16 to 0.21) |
| Georgia | 126.44 (65.98 to 184.19) | 126.16 (66.49 to 183.45) | -0.03 (-0.04 to -0.02) | 134.06 (55.01 to 297.90) | 136.02 (56.40 to 302.90) | 0.18 (0.14 to 0.23) |
| Germany | 124.65 (70.14 to 173.51) | 125.28 (70.59 to 174.26) | 0.00 (-0.00 to 0.01) | 127.47 (58.03 to 271.37) | 137.47 (63.77 to 288.76) | 0.26 (0.25 to 0.28) |
| Ghana | 125.95 (65.54 to 184.39) | 127.06 (69.52 to 180.18) | 0.03 (0.03 to 0.04) | 118.47 (48.25 to 265.79) | 127.08 (54.50 to 281.82) | 0.24 (0.23 to 0.26) |
| Greece | 125.14 (68.04 to 178.63) | 124.97 (68.51 to 176.84) | -0.01 (-0.02 to -0.01) | 125.59 (53.90 to 277.91) | 139.44 (62.22 to 297.82) | 0.39 (0.37 to 0.41) |
| Greenland | 123.69 (4.92 to 461.41) | 124.98 (18.85 to 324.59) | 0.03 (0.02 to 0.03) | 113.33 (1.73 to 566.76) | 120.43 (11.29 to 435.08) | 0.25 (0.23 to 0.28) |
| Grenada | 124.19 (22.95 to 299.13) | 125.60 (28.69 to 277.85) | 0.04 (0.04 to 0.05) | 131.82 (17.55 to 434.17) | 129.61 (20.84 to 407.34) | -0.15 (-0.20 to -0.11) |
| Guam | 129.06 (20.81 to 327.91) | 128.24 (41.31 to 242.01) | -0.03 (-0.04 to -0.02) | 133.26 (13.20 to 471.22) | 147.29 (37.03 to 399.12) | 0.36 (0.31 to 0.41) |
| Guatemala | 123.76 (60.50 to 186.91) | 124.90 (67.87 to 179.30) | 0.02 (0.02 to 0.03) | 118.37 (45.28 to 270.05) | 127.77 (54.39 to 282.61) | 0.28 (0.26 to 0.31) |
| Guinea | 124.82 (63.41 to 186.89) | 125.53 (64.85 to 185.23) | 0.02 (0.01 to 0.03) | 118.43 (45.78 to 272.21) | 124.15 (49.83 to 282.65) | 0.19 (0.17 to 0.21) |
| Guinea-Bissau | 124.85 (44.88 to 225.86) | 125.88 (51.20 to 213.78) | 0.01 (0.01 to 0.02) | 115.43 (30.13 to 310.88) | 118.04 (35.15 to 300.05) | 0.04 (0.03 to 0.06) |
| Guyana | 125.46 (44.87 to 225.15) | 126.00 (51.70 to 211.96) | -0.01 (-0.01 to 0.00) | 124.02 (32.97 to 328.22) | 127.23 (39.60 to 316.53) | 0.13 (0.10 to 0.17) |
| Haiti | 123.53 (60.69 to 187.54) | 123.27 (63.57 to 182.28) | 0.00 (-0.00 to 0.01) | 114.34 (43.49 to 265.07) | 117.00 (47.17 to 265.16) | 0.15 (0.12 to 0.18) |
| Honduras | 124.53 (59.31 to 192.98) | 125.69 (65.19 to 182.30) | 0.02 (0.02 to 0.03) | 123.94 (46.38 to 291.23) | 128.32 (52.67 to 286.13) | 0.11 (0.09 to 0.14) |
| Hungary | 127.24 (69.53 to 180.70) | 126.68 (69.98 to 178.43) | -0.03 (-0.03 to -0.02) | 132.68 (58.36 to 290.72) | 140.25 (62.23 to 300.69) | 0.21 (0.19 to 0.23) |
| Iceland | 125.56 (43.95 to 229.95) | 125.84 (52.07 to 210.80) | 0.02 (0.02 to 0.03) | 129.20 (34.67 to 341.05) | 138.10 (45.70 to 339.81) | 0.25 (0.22 to 0.28) |
| India | 126.97 (73.63 to 172.85) | 126.78 (73.57 to 172.21) | -0.01 (-0.01 to -0.00) | 118.41 (54.20 to 254.34) | 126.86 (58.36 to 273.56) | 0.26 (0.24 to 0.27) |
| Indonesia | 127.06 (71.64 to 175.00) | 127.35 (73.69 to 174.01) | 0.00 (-0.00 to 0.01) | 122.17 (54.71 to 263.96) | 126.34 (56.75 to 272.69) | 0.16 (0.14 to 0.17) |
| Iran (Islamic Republic of) | 128.36 (71.91 to 177.52) | 127.18 (72.80 to 174.99) | -0.04 (-0.05 to -0.03) | 130.21 (56.29 to 286.76) | 139.16 (62.68 to 299.84) | 0.28 (0.24 to 0.32) |
| Iraq | 127.56 (67.98 to 182.74) | 127.90 (70.65 to 179.80) | 0.01 (0.00 to 0.01) | 139.56 (57.30 to 312.93) | 134.95 (59.20 to 294.35) | -0.06 (-0.08 to -0.04) |
| Ireland | 125.04 (63.49 to 184.82) | 125.67 (66.95 to 181.45) | 0.01 (0.00 to 0.01) | 123.77 (49.48 to 279.69) | 133.90 (56.88 to 295.10) | 0.21 (0.18 to 0.24) |
| Israel | 124.71 (63.62 to 184.37) | 125.22 (66.73 to 178.96) | -0.01 (-0.02 to -0.00) | 123.39 (50.65 to 274.47) | 133.29 (58.00 to 293.20) | 0.23 (0.18 to 0.28) |
| Italy | 126.23 (71.28 to 174.45) | 125.35 (71.08 to 173.43) | -0.02 (-0.03 to -0.02) | 131.28 (59.69 to 281.17) | 143.59 (66.36 to 304.61) | 0.28 (0.27 to 0.30) |
| Jamaica | 124.82 (59.93 to 191.15) | 125.57 (63.32 to 187.07) | 0.02 (0.01 to 0.02) | 131.98 (49.75 to 305.61) | 138.83 (56.11 to 313.04) | 0.20 (0.16 to 0.25) |
| Japan | 125.49 (71.23 to 173.29) | 125.00 (70.77 to 171.79) | -0.01 (-0.02 to -0.01) | 125.39 (57.47 to 270.84) | 139.21 (64.90 to 296.48) | 0.34 (0.33 to 0.35) |
| Jordan | 128.03 (58.56 to 203.07) | 128.67 (68.55 to 184.91) | 0.01 (-0.00 to 0.02) | 132.17 (45.65 to 319.73) | 141.66 (58.60 to 314.00) | 0.25 (0.24 to 0.26) |
| Kazakhstan | 126.70 (67.92 to 181.34) | 128.16 (71.34 to 180.64) | 0.00 (-0.02 to 0.01) | 133.51 (56.67 to 292.62) | 134.10 (59.11 to 293.02) | 0.12 (0.08 to 0.16) |
| Kenya | 127.02 (67.26 to 182.46) | 127.41 (70.71 to 178.62) | 0.01 (0.01 to 0.02) | 124.43 (51.60 to 279.42) | 127.53 (55.20 to 280.75) | 0.11 (0.09 to 0.13) |
| Kiribati | 126.23 (8.58 to 417.54) | 126.90 (17.28 to 338.46) | 0.01 (0.00 to 0.02) | 120.37 (3.93 to 536.15) | 125.59 (10.37 to 459.47) | 0.08 (0.02 to 0.15) |
| Kuwait | 129.27 (49.76 to 223.85) | 129.36 (63.38 to 194.55) | -0.02 (-0.03 to -0.01) | 143.66 (41.91 to 371.39) | 152.12 (57.98 to 350.38) | 0.33 (0.27 to 0.38) |
| Kyrgyzstan | 124.81 (62.05 to 189.15) | 126.02 (63.94 to 186.01) | -0.02 (-0.04 to -0.01) | 127.28 (49.20 to 293.71) | 127.62 (50.10 to 293.25) | 0.14 (0.09 to 0.19) |
| Lao People's Democratic Republic | 124.78 (59.37 to 192.72) | 125.84 (64.49 to 186.30) | 0.03 (0.02 to 0.04) | 114.06 (41.33 to 268.13) | 122.24 (48.43 to 275.47) | 0.28 (0.25 to 0.30) |
| Latvia | 125.48 (63.99 to 186.62) | 126.20 (64.74 to 186.12) | 0.00 (-0.00 to 0.01) | 132.08 (53.59 to 296.54) | 141.36 (58.10 to 318.56) | 0.33 (0.29 to 0.38) |
| Lebanon | 127.53 (61.24 to 194.03) | 127.60 (68.32 to 183.61) | -0.01 (-0.01 to -0.00) | 132.20 (49.79 to 307.16) | 149.12 (62.54 to 328.00) | 0.45 (0.44 to 0.47) |
| Lesotho | 123.68 (53.34 to 204.68) | 125.49 (55.72 to 201.30) | 0.06 (0.05 to 0.06) | 119.70 (38.70 to 296.14) | 121.45 (40.58 to 291.95) | 0.01 (-0.00 to 0.03) |
| Liberia | 125.80 (57.06 to 200.08) | 127.41 (60.78 to 195.34) | 0.05 (0.04 to 0.07) | 119.98 (41.66 to 285.77) | 126.84 (46.50 to 296.21) | 0.25 (0.21 to 0.29) |
| Libya | 128.19 (60.85 to 196.63) | 128.06 (66.87 to 185.55) | -0.02 (-0.02 to -0.01) | 140.66 (52.19 to 330.01) | 141.54 (57.86 to 316.86) | 0.07 (0.05 to 0.09) |
| Lithuania | 125.44 (64.64 to 185.01) | 125.51 (65.44 to 183.21) | -0.02 (-0.03 to -0.01) | 132.36 (53.34 to 297.64) | 140.30 (59.33 to 311.17) | 0.29 (0.26 to 0.33) |
| Luxembourg | 125.14 (49.85 to 211.95) | 125.90 (56.93 to 199.96) | 0.03 (0.03 to 0.03) | 126.94 (40.59 to 320.07) | 136.91 (48.91 to 324.01) | 0.29 (0.27 to 0.32) |
| Madagascar | 124.99 (64.30 to 186.21) | 124.66 (64.79 to 180.41) | -0.01 (-0.02 to -0.01) | 116.07 (46.02 to 264.53) | 116.30 (47.21 to 260.79) | 0.04 (0.02 to 0.06) |
| Malawi | 125.00 (62.84 to 186.49) | 125.49 (65.23 to 183.05) | 0.03 (0.02 to 0.04) | 112.43 (43.48 to 256.18) | 119.70 (48.44 to 268.33) | 0.22 (0.18 to 0.26) |
| Malaysia | 126.39 (67.85 to 182.80) | 128.07 (71.16 to 179.60) | 0.06 (0.05 to 0.07) | 126.17 (52.98 to 281.40) | 131.54 (57.07 to 287.70) | 0.19 (0.16 to 0.21) |
| Maldives | 125.69 (20.45 to 317.65) | 126.82 (41.97 to 236.89) | 0.04 (0.03 to 0.05) | 116.15 (10.86 to 414.62) | 132.14 (33.38 to 362.34) | 0.59 (0.52 to 0.66) |
| Mali | 124.71 (63.52 to 186.51) | 125.36 (65.37 to 181.86) | 0.01 (0.00 to 0.01) | 113.68 (43.97 to 262.92) | 119.33 (49.18 to 266.15) | 0.19 (0.17 to 0.21) |
| Malta | 124.74 (46.50 to 217.43) | 125.46 (56.07 to 199.72) | 0.03 (0.02 to 0.04) | 123.54 (35.82 to 321.68) | 136.42 (49.16 to 323.36) | 0.27 (0.22 to 0.32) |
| Marshall Islands | 124.98 (1.23 to 598.74) | 127.22 (6.89 to 444.38) | 0.05 (0.05 to 0.05) | 118.75 (0.32 to 727.21) | 121.98 (2.77 to 568.17) | -0.02 (-0.09 to 0.04) |
| Mauritania | 126.90 (56.24 to 202.69) | 128.08 (62.52 to 194.49) | 0.02 (0.01 to 0.03) | 125.85 (42.14 to 303.59) | 134.62 (50.11 to 318.02) | 0.19 (0.16 to 0.23) |
| Mauritius | 127.21 (53.88 to 210.19) | 127.35 (61.23 to 194.75) | 0.01 (0.01 to 0.02) | 122.92 (39.13 to 303.53) | 128.91 (48.69 to 304.61) | 0.18 (0.14 to 0.22) |
| Mexico | 127.34 (71.76 to 176.24) | 127.28 (72.93 to 174.14) | -0.01 (-0.01 to -0.00) | 136.88 (61.07 to 298.56) | 142.56 (64.93 to 309.09) | 0.15 (0.14 to 0.16) |
| Micronesia (Federated States of) | 127.27 (14.63 to 357.72) | 127.86 (19.29 to 336.63) | 0.00 (-0.01 to 0.01) | 126.83 (8.57 to 486.15) | 128.64 (11.64 to 465.98) | -0.02 (-0.11 to 0.07) |
| Monaco | 126.14 (24.39 to 298.76) | 125.60 (28.85 to 276.35) | 0.00 (-0.00 to 0.01) | 134.22 (18.58 to 434.64) | 140.31 (25.03 to 420.11) | 0.12 (0.08 to 0.16) |
| Mongolia | 125.07 (55.58 to 200.93) | 125.73 (59.17 to 194.76) | 0.00 (-0.00 to 0.01) | 124.88 (42.40 to 303.59) | 126.15 (46.61 to 294.89) | 0.08 (0.05 to 0.11) |
| Montenegro | 126.64 (52.38 to 213.13) | 126.26 (57.21 to 199.77) | -0.02 (-0.03 to -0.01) | 134.96 (43.04 to 341.63) | 133.60 (46.75 to 318.06) | 0.09 (0.04 to 0.13) |
| Morocco | 126.01 (68.49 to 179.07) | 127.32 (70.87 to 177.08) | 0.03 (0.03 to 0.04) | 129.30 (55.01 to 287.22) | 134.77 (59.29 to 294.56) | 0.18 (0.17 to 0.20) |
| Mozambique | 124.76 (63.70 to 184.89) | 125.17 (66.16 to 182.56) | 0.01 (0.00 to 0.02) | 112.25 (44.43 to 251.66) | 116.21 (48.23 to 257.28) | 0.11 (0.09 to 0.13) |
| Myanmar | 124.61 (67.08 to 176.67) | 124.94 (70.05 to 176.01) | 0.02 (0.01 to 0.02) | 113.26 (48.81 to 248.63) | 119.14 (52.34 to 259.29) | 0.27 (0.23 to 0.31) |
| Namibia | 125.22 (51.57 to 212.09) | 125.68 (57.09 to 198.43) | 0.02 (0.01 to 0.02) | 118.67 (35.46 to 304.32) | 126.12 (44.62 to 304.62) | 0.20 (0.18 to 0.23) |
| Nauru | 128.02 (0.00 to 1550.31) | 128.44 (0.00 to 1260.18) | 0.00 (-0.01 to 0.00) | 127.01 (0.00 to 1729.09) | 131.59 (0.00 to 1442.47) | 0.04 (0.01 to 0.08) |
| Nepal | 124.91 (66.21 to 181.62) | 125.20 (68.37 to 177.82) | 0.03 (0.02 to 0.04) | 112.49 (46.33 to 248.78) | 119.95 (52.08 to 262.09) | 0.24 (0.23 to 0.26) |
| Netherlands | 124.98 (67.67 to 177.39) | 125.15 (68.98 to 175.95) | 0.00 (-0.01 to 0.00) | 125.70 (55.13 to 272.59) | 133.28 (60.22 to 286.05) | 0.15 (0.12 to 0.18) |
| New Zealand | 126.49 (64.92 to 185.55) | 126.58 (68.22 to 181.39) | 0.00 (-0.00 to 0.01) | 131.61 (53.37 to 295.06) | 143.28 (62.15 to 315.21) | 0.25 (0.20 to 0.29) |
| Nicaragua | 124.65 (57.28 to 196.16) | 125.56 (64.77 to 184.73) | 0.01 (0.00 to 0.02) | 125.00 (45.34 to 296.32) | 131.64 (53.60 to 295.83) | 0.14 (0.11 to 0.18) |
| Niger | 125.15 (61.22 to 190.39) | 124.83 (64.91 to 181.96) | 0.00 (-0.01 to 0.01) | 114.96 (42.45 to 271.43) | 118.00 (48.20 to 267.06) | 0.06 (0.03 to 0.09) |
| Nigeria | 127.28 (71.66 to 176.26) | 127.23 (72.45 to 175.00) | 0.00 (-0.00 to 0.00) | 126.15 (56.15 to 274.49) | 131.71 (59.17 to 285.98) | 0.09 (0.04 to 0.14) |
| Niue | 127.02 (0.00 to 2144.47) | 128.30 (0.00 to 2340.21) | 0.01 (0.00 to 0.02) | 139.63 (0.00 to 2371.87) | 144.07 (0.00 to 2588.78) | 0.12 (0.09 to 0.15) |
| North Macedonia | 126.31 (59.56 to 193.65) | 126.13 (63.95 to 186.39) | -0.02 (-0.03 to -0.01) | 129.50 (48.75 to 302.43) | 130.60 (52.26 to 296.00) | 0.13 (0.09 to 0.17) |
| Northern Mariana Islands | 129.06 (0.35 to 743.41) | 128.77 (14.02 to 372.65) | 0.00 (-0.01 to 0.01) | 133.70 (0.09 to 921.98) | 136.95 (9.03 to 529.82) | -0.03 (-0.12 to 0.06) |
| Norway | 125.86 (66.53 to 181.17) | 126.08 (68.18 to 180.08) | 0.02 (0.01 to 0.02) | 131.79 (56.23 to 290.97) | 140.22 (61.28 to 307.55) | 0.18 (0.14 to 0.23) |
| Oman | 126.14 (50.74 to 215.31) | 128.70 (61.06 to 198.82) | 0.08 (0.07 to 0.09) | 124.37 (37.74 to 318.68) | 140.33 (51.12 to 333.32) | 0.43 (0.40 to 0.46) |
| Pakistan | 127.16 (72.01 to 175.45) | 127.26 (72.26 to 173.75) | 0.01 (0.00 to 0.01) | 125.35 (55.78 to 271.31) | 129.39 (58.55 to 278.65) | 0.11 (0.10 to 0.12) |
| Palau | 128.39 (0.21 to 789.20) | 129.63 (3.58 to 519.00) | 0.02 (0.01 to 0.04) | 134.57 (0.05 to 979.46) | 138.13 (1.96 to 691.50) | 0.02 (-0.09 to 0.12) |
| Palestine | 127.38 (55.84 to 206.46) | 126.93 (61.89 to 192.38) | -0.01 (-0.02 to 0.00) | 131.78 (44.02 to 323.10) | 131.95 (49.17 to 304.82) | 0.03 (0.00 to 0.06) |
| Panama | 123.92 (57.23 to 195.08) | 125.90 (64.30 to 184.98) | 0.04 (0.04 to 0.05) | 123.88 (44.88 to 294.25) | 135.98 (55.90 to 306.01) | 0.26 (0.24 to 0.28) |
| Papua New Guinea | 125.15 (58.67 to 195.79) | 125.26 (64.37 to 185.81) | 0.00 (-0.02 to 0.01) | 114.11 (40.45 to 272.52) | 119.55 (46.73 to 272.14) | 0.10 (0.04 to 0.17) |
| Paraguay | 125.58 (61.24 to 191.49) | 125.69 (65.08 to 184.25) | 0.00 (-0.01 to 0.01) | 127.98 (48.52 to 292.84) | 132.36 (54.04 to 296.96) | 0.11 (0.08 to 0.14) |
| Peru | 124.83 (67.10 to 179.43) | 126.17 (70.05 to 177.19) | 0.02 (0.01 to 0.02) | 127.13 (53.91 to 279.74) | 137.50 (60.96 to 302.29) | 0.32 (0.29 to 0.34) |
| Philippines | 127.12 (70.44 to 177.66) | 127.44 (72.32 to 175.30) | 0.00 (-0.00 to 0.00) | 124.39 (54.24 to 274.57) | 129.27 (57.88 to 281.05) | 0.12 (0.11 to 0.14) |
| Poland | 126.88 (71.72 to 175.74) | 127.23 (72.31 to 174.50) | 0.00 (-0.01 to 0.00) | 133.98 (59.96 to 290.10) | 144.73 (65.55 to 310.52) | 0.35 (0.31 to 0.39) |
| Portugal | 124.18 (65.73 to 178.27) | 124.90 (67.83 to 176.09) | 0.00 (-0.01 to 0.00) | 121.41 (52.52 to 268.43) | 136.76 (60.99 to 295.70) | 0.37 (0.35 to 0.39) |
| Puerto Rico | 127.80 (65.70 to 188.52) | 127.55 (67.79 to 182.91) | -0.01 (-0.01 to -0.00) | 142.32 (58.30 to 322.85) | 153.30 (64.85 to 337.57) | 0.24 (0.22 to 0.27) |
| Qatar | 130.41 (19.61 to 340.30) | 130.92 (52.81 to 221.57) | 0.01 (0.01 to 0.02) | 142.26 (13.75 to 509.69) | 146.68 (42.90 to 373.39) | 0.10 (0.07 to 0.14) |
| Republic of Korea | 123.78 (67.64 to 176.74) | 124.28 (68.23 to 173.90) | 0.01 (0.01 to 0.02) | 112.30 (48.90 to 244.36) | 122.17 (55.85 to 258.57) | 0.29 (0.27 to 0.32) |
| Republic of Moldova | 124.95 (63.40 to 184.85) | 126.09 (66.62 to 182.19) | 0.01 (0.00 to 0.03) | 122.71 (49.28 to 277.68) | 135.20 (56.33 to 301.07) | 0.46 (0.40 to 0.52) |
| Romania | 126.06 (71.15 to 176.51) | 125.89 (69.69 to 175.76) | 0.00 (-0.01 to 0.00) | 128.28 (56.35 to 281.95) | 140.43 (62.22 to 305.43) | 0.38 (0.34 to 0.42) |
| Russian Federation | 126.02 (71.72 to 173.56) | 127.12 (73.30 to 173.04) | 0.00 (-0.01 to 0.01) | 132.04 (60.20 to 284.50) | 139.43 (64.27 to 300.04) | 0.34 (0.28 to 0.40) |
| Rwanda | 125.27 (61.74 to 190.66) | 125.27 (65.95 to 184.03) | 0.01 (0.01 to 0.02) | 113.46 (41.76 to 266.38) | 118.32 (47.60 to 265.29) | 0.19 (0.16 to 0.22) |
| Saint Kitts and Nevis | 126.44 (14.76 to 356.60) | 127.78 (20.69 to 324.00) | 0.03 (0.02 to 0.04) | 131.85 (9.60 to 497.13) | 133.06 (13.98 to 470.03) | 0.06 (-0.02 to 0.14) |
| Saint Lucia | 125.41 (25.84 to 289.51) | 125.88 (41.27 to 239.17) | 0.00 (-0.00 to 0.01) | 126.46 (18.29 to 409.31) | 135.63 (33.59 to 366.50) | 0.24 (0.20 to 0.27) |
| Saint Vincent and the Grenadines | 124.64 (22.08 to 302.89) | 126.16 (33.91 to 260.49) | 0.03 (0.02 to 0.03) | 125.85 (15.55 to 420.30) | 133.50 (26.39 to 394.73) | 0.24 (0.20 to 0.29) |
| Samoa | 128.28 (24.35 to 306.77) | 128.12 (33.01 to 269.86) | -0.02 (-0.03 to -0.01) | 132.35 (16.57 to 441.79) | 137.40 (25.13 to 414.38) | 0.10 (0.05 to 0.16) |
| San Marino | 125.48 (11.88 to 378.62) | 125.31 (23.88 to 299.50) | 0.00 (0.00 to 0.00) | 129.08 (7.50 to 511.13) | 140.85 (20.32 to 451.84) | 0.27 (0.22 to 0.31) |
| Sao Tome and Principe | 126.04 (19.85 to 324.16) | 126.85 (26.66 to 291.99) | 0.02 (0.01 to 0.02) | 123.41 (12.35 to 440.30) | 129.06 (18.33 to 414.56) | 0.19 (0.17 to 0.21) |
| Saudi Arabia | 127.72 (67.26 to 185.23) | 130.02 (71.55 to 182.64) | 0.06 (0.06 to 0.06) | 133.41 (53.33 to 298.62) | 142.66 (60.75 to 313.79) | 0.29 (0.27 to 0.31) |
| Senegal | 126.04 (62.36 to 189.02) | 126.02 (66.35 to 182.09) | 0.00 (-0.00 to 0.00) | 122.59 (46.84 to 282.86) | 125.62 (52.15 to 278.95) | 0.08 (0.07 to 0.10) |
| Serbia | 126.80 (69.01 to 180.31) | 126.49 (69.10 to 178.37) | -0.02 (-0.03 to -0.01) | 126.64 (53.17 to 281.14) | 138.34 (60.94 to 302.65) | 0.40 (0.37 to 0.43) |
| Seychelles | 127.19 (19.17 to 329.32) | 127.82 (29.47 to 282.59) | 0.00 (-0.01 to 0.01) | 131.81 (13.48 to 465.11) | 130.69 (20.93 to 408.91) | 0.03 (0.01 to 0.04) |
| Sierra Leone | 124.94 (59.94 to 191.64) | 125.14 (62.17 to 187.39) | 0.02 (0.01 to 0.04) | 118.94 (44.61 to 277.25) | 123.44 (48.78 to 280.22) | 0.08 (0.06 to 0.10) |
| Singapore | 124.30 (58.93 to 192.61) | 125.51 (66.32 to 181.55) | 0.04 (0.03 to 0.04) | 116.02 (43.01 to 270.41) | 125.87 (52.72 to 277.64) | 0.29 (0.26 to 0.32) |
| Slovakia | 126.51 (66.74 to 183.35) | 126.75 (68.42 to 180.74) | 0.00 (-0.00 to 0.01) | 131.98 (55.27 to 291.42) | 137.81 (58.28 to 303.57) | 0.13 (0.11 to 0.15) |
| Slovenia | 126.11 (61.97 to 190.64) | 126.23 (65.35 to 184.79) | -0.02 (-0.03 to -0.01) | 131.69 (51.33 to 304.89) | 142.18 (59.57 to 314.86) | 0.31 (0.28 to 0.33) |
| Solomon Islands | 125.82 (29.04 to 281.12) | 125.73 (41.99 to 234.52) | -0.02 (-0.03 to -0.01) | 119.32 (17.63 to 378.08) | 125.87 (31.53 to 341.84) | 0.14 (0.09 to 0.19) |
| Somalia | 124.60 (58.01 to 195.71) | 124.64 (63.58 to 184.51) | -0.01 (-0.02 to 0.00) | 113.75 (39.60 to 269.53) | 110.54 (43.84 to 247.27) | -0.04 (-0.06 to -0.01) |
| South Africa | 126.77 (70.35 to 177.48) | 126.97 (72.08 to 175.02) | 0.01 (0.01 to 0.01) | 138.22 (60.36 to 301.39) | 139.59 (61.98 to 302.99) | 0.11 (0.08 to 0.13) |
| South Sudan | 124.59 (60.65 to 189.80) | 124.64 (61.37 to 189.10) | 0.00 (-0.01 to 0.00) | 114.11 (43.40 to 264.03) | 116.87 (44.98 to 273.94) | 0.03 (-0.01 to 0.08) |
| Spain | 125.27 (70.16 to 175.28) | 125.28 (70.08 to 174.21) | -0.01 (-0.02 to -0.01) | 126.19 (56.33 to 269.29) | 139.06 (63.31 to 294.99) | 0.31 (0.27 to 0.34) |
| Sri Lanka | 125.67 (66.26 to 180.74) | 125.61 (69.61 to 176.28) | 0.01 (0.01 to 0.02) | 119.75 (49.91 to 269.50) | 122.78 (54.44 to 269.05) | 0.07 (0.05 to 0.09) |
| Sudan | 124.16 (65.06 to 180.77) | 126.63 (69.60 to 178.91) | 0.07 (0.06 to 0.08) | 119.94 (49.27 to 266.29) | 134.78 (57.86 to 294.50) | 0.37 (0.35 to 0.39) |
| Suriname | 125.89 (39.98 to 241.39) | 126.62 (52.03 to 211.39) | -0.01 (-0.01 to 0.00) | 131.07 (31.31 to 360.49) | 135.88 (43.46 to 341.53) | 0.18 (0.15 to 0.21) |
| Sweden | 125.71 (68.58 to 178.53) | 125.35 (69.05 to 177.17) | 0.01 (0.00 to 0.02) | 134.57 (58.27 to 294.78) | 143.27 (63.79 to 311.56) | 0.13 (0.09 to 0.18) |
| Switzerland | 124.22 (65.39 to 180.13) | 124.67 (67.73 to 177.84) | 0.00 (-0.00 to 0.01) | 125.91 (53.44 to 275.72) | 133.87 (59.17 to 288.92) | 0.18 (0.17 to 0.19) |
| Syrian Arab Republic | 126.92 (65.64 to 185.81) | 127.46 (70.45 to 180.16) | -0.01 (-0.02 to 0.00) | 132.46 (53.20 to 297.48) | 133.92 (57.46 to 298.22) | 0.08 (0.06 to 0.10) |
| Taiwan (Province of China) | 127.91 (70.27 to 180.80) | 127.11 (71.66 to 176.23) | -0.01 (-0.02 to 0.00) | 125.50 (54.44 to 272.01) | 134.71 (59.04 to 287.31) | 0.34 (0.27 to 0.41) |
| Tajikistan | 124.01 (60.01 to 189.76) | 125.08 (63.87 to 185.96) | -0.01 (-0.02 to 0.00) | 125.72 (47.55 to 291.62) | 123.00 (49.09 to 279.69) | 0.08 (0.03 to 0.14) |
| Thailand | 125.22 (68.93 to 176.11) | 127.19 (71.89 to 175.15) | 0.05 (0.04 to 0.05) | 119.00 (51.72 to 261.33) | 133.48 (59.74 to 287.42) | 0.46 (0.43 to 0.49) |
| Timor-Leste | 124.43 (37.13 to 248.39) | 123.50 (53.08 to 202.38) | -0.03 (-0.03 to -0.02) | 113.68 (22.97 to 334.13) | 114.66 (36.91 to 280.06) | 0.06 (0.03 to 0.10) |
| Togo | 125.39 (55.76 to 201.48) | 126.01 (62.90 to 188.79) | 0.00 (-0.00 to 0.01) | 118.14 (40.00 to 286.78) | 120.53 (46.09 to 278.52) | 0.01 (-0.01 to 0.04) |
| Tokelau | 126.17 (0.00 to 3558.22) | 127.81 (0.00 to 3042.41) | 0.01 (0.00 to 0.02) | 129.98 (0.00 to 3755.85) | 141.35 (0.00 to 3285.52) | 0.32 (0.25 to 0.39) |
| Tonga | 127.37 (16.35 to 347.53) | 127.08 (23.78 to 304.32) | -0.03 (-0.04 to -0.02) | 132.13 (10.64 to 488.36) | 139.78 (19.06 to 456.46) | 0.12 (0.06 to 0.19) |
| Trinidad and Tobago | 126.52 (55.28 to 204.59) | 126.94 (61.83 to 193.00) | 0.02 (0.01 to 0.02) | 130.68 (43.96 to 321.47) | 134.99 (51.91 to 310.53) | 0.12 (0.08 to 0.16) |
| Tunisia | 126.62 (65.22 to 185.03) | 127.24 (69.99 to 180.10) | 0.01 (0.00 to 0.01) | 128.97 (52.22 to 294.63) | 135.72 (58.51 to 296.25) | 0.22 (0.20 to 0.23) |
| Turkey | 127.54 (71.95 to 177.16) | 127.86 (73.58 to 175.79) | 0.00 (-0.01 to 0.01) | 134.35 (58.93 to 295.86) | 141.41 (64.15 to 304.33) | 0.26 (0.23 to 0.29) |
| Turkmenistan | 125.34 (58.58 to 195.66) | 126.76 (64.06 to 188.26) | 0.00 (-0.01 to 0.01) | 127.92 (47.11 to 295.34) | 131.89 (52.54 to 300.31) | 0.25 (0.21 to 0.30) |
| Tuvalu | 126.87 (0.01 to 1016.40) | 127.72 (0.32 to 739.09) | 0.00 (-0.01 to 0.00) | 120.71 (0.00 to 1161.52) | 132.20 (0.09 to 910.01) | 0.23 (0.14 to 0.31) |
| Uganda | 124.16 (63.65 to 182.84) | 125.11 (67.88 to 179.40) | 0.01 (0.01 to 0.02) | 115.27 (47.35 to 260.56) | 119.85 (50.91 to 266.00) | 0.15 (0.12 to 0.18) |
| Ukraine | 126.42 (71.85 to 175.08) | 126.52 (71.86 to 175.03) | -0.03 (-0.04 to -0.01) | 133.39 (59.60 to 287.84) | 137.64 (62.06 to 290.74) | 0.23 (0.19 to 0.28) |
| United Arab Emirates | 127.57 (42.81 to 238.47) | 131.89 (67.15 to 195.80) | 0.11 (0.10 to 0.11) | 131.55 (32.13 to 359.12) | 134.65 (48.60 to 315.62) | 0.10 (0.05 to 0.14) |
| United Kingdom | 126.03 (71.18 to 174.08) | 126.04 (71.26 to 173.40) | 0.01 (0.00 to 0.01) | 134.36 (61.51 to 287.77) | 143.81 (66.20 to 306.93) | 0.20 (0.17 to 0.23) |
| United Republic of Tanzania | 125.58 (66.73 to 180.69) | 126.45 (69.57 to 178.65) | 0.02 (0.02 to 0.02) | 117.59 (49.07 to 257.04) | 126.57 (54.75 to 278.73) | 0.27 (0.25 to 0.28) |
| United States of America | 129.27 (74.02 to 174.48) | 127.91 (73.53 to 172.88) | -0.03 (-0.04 to -0.02) | 128.35 (60.95 to 269.62) | 132.17 (63.44 to 277.36) | 0.08 (0.03 to 0.14) |
| United States Virgin Islands | 128.37 (24.20 to 307.50) | 127.61 (40.12 to 244.87) | -0.02 (-0.03 to -0.01) | 138.92 (18.46 to 460.93) | 144.61 (34.85 to 394.59) | 0.07 (0.05 to 0.09) |
| Uruguay | 124.42 (62.31 to 185.52) | 125.34 (64.15 to 183.64) | 0.01 (0.01 to 0.02) | 123.49 (50.02 to 277.66) | 138.03 (57.21 to 302.43) | 0.38 (0.33 to 0.42) |
| Uzbekistan | 124.46 (65.99 to 181.18) | 126.47 (70.00 to 178.16) | 0.04 (0.03 to 0.04) | 129.26 (54.56 to 288.25) | 128.79 (55.85 to 282.49) | 0.15 (0.09 to 0.20) |
| Vanuatu | 126.24 (16.37 to 347.02) | 126.62 (33.17 to 262.73) | 0.01 (-0.00 to 0.02) | 121.46 (8.83 to 463.87) | 126.08 (23.72 to 380.45) | 0.10 (0.07 to 0.12) |
| Venezuela (Bolivarian Republic of) | 126.09 (67.25 to 182.77) | 126.73 (70.52 to 177.07) | 0.00 (-0.01 to 0.01) | 130.63 (54.85 to 289.26) | 134.88 (59.32 to 294.23) | 0.12 (0.10 to 0.14) |
| Viet Nam | 124.08 (69.24 to 176.40) | 124.35 (68.70 to 174.18) | 0.01 (-0.00 to 0.02) | 117.10 (51.00 to 255.53) | 119.10 (52.78 to 260.08) | 0.18 (0.14 to 0.23) |
| Yemen | 124.12 (62.40 to 184.97) | 124.95 (67.42 to 179.52) | 0.02 (0.02 to 0.03) | 114.70 (44.97 to 262.43) | 123.53 (51.95 to 273.61) | 0.27 (0.25 to 0.30) |
| Zambia | 125.61 (60.77 to 190.60) | 125.83 (66.19 to 184.37) | 0.01 (-0.00 to 0.02) | 118.56 (44.85 to 274.46) | 121.17 (49.30 to 274.37) | 0.11 (0.09 to 0.14) |
| Zimbabwe | 125.75 (64.10 to 186.40) | 124.53 (64.80 to 182.96) | -0.05 (-0.06 to -0.04) | 122.21 (47.97 to 278.67) | 118.88 (47.97 to 270.99) | -0.13 (-0.17 to -0.08) |

**Abbreviations: DALY=disability-adjusted life-years. EAPC=estimated annual percentage change. CI=confidence interval**

**Table S7. Age-standardised DALYs attributable to high BMI for osteoarthritis, hip osteoarthritis, and knee osteoarthritis among older adults in 1990 and 2021, and their proportions.**

|  |  | **Age-standardised DALYs attributable to high BMI (Proportion %)** | |
| --- | --- | --- | --- |
|  | **Cause** | **1990** | **2021** |
| Global | Osteoarthritis | 186.73 (15.83) | 246.38 (19.22) |
|  | Hip Osteoarthritis | 21.24 (29.85) | 26.27 (35.09) |
|  | Knee Osteoarthritis | 165.49 (26.15) | 220.11 (32.60) |
| **GBD regions** | |  |  |
| East Asia | Osteoarthritis | 113.23 (11.43) | 219.65 (18.69) |
|  | Hip Osteoarthritis | 5.21 (16.14) | 12.37 (28.44) |
|  | Knee Osteoarthritis | 108.02 (16.54) | 207.28 (29.43) |
| South Asia | Osteoarthritis | 55.73 (6.06) | 117.46 (10.67) |
|  | Hip Osteoarthritis | 3.55 (10.32) | 8.50 (19.03) |
|  | Knee Osteoarthritis | 52.18 (10.14) | 108.96 (18.91) |
| Southeast Asia | Osteoarthritis | 55.21 (6.78) | 112.38 (11.45) |
|  | Hip Osteoarthritis | 4.62 (11.42) | 9.51 (20.34) |
|  | Knee Osteoarthritis | 50.59 (12.09) | 102.87 (21.77) |
| Central Asia | Osteoarthritis | 181.58 (15.10) | 216.99 (16.32) |
|  | Hip Osteoarthritis | 27.04 (34.38) | 33.51 (39.59) |
|  | Knee Osteoarthritis | 154.54 (35.64) | 183.48 (40.65) |
| High-income Asia Pacific | Osteoarthritis | 208.23 (12.80) | 263.38 (14.93) |
|  | Hip Osteoarthritis | 14.70 (19.59) | 20.74 (24.35) |
|  | Knee Osteoarthritis | 193.54 (20.24) | 242.64 (24.85) |
| Oceania | Osteoarthritis | 151.32 (16.57) | 191.79 (18.71) |
|  | Hip Osteoarthritis | 11.92 (25.81) | 15.28 (30.48) |
|  | Knee Osteoarthritis | 139.40 (26.67) | 176.50 (31.27) |
| Australasia | Osteoarthritis | 290.94 (21.21) | 406.08 (26.81) |
|  | Hip Osteoarthritis | 35.82 (34.90) | 58.64 (43.22) |
|  | Knee Osteoarthritis | 255.12 (35.15) | 347.45 (43.36) |
| Eastern Europe | Osteoarthritis | 229.25 (16.27) | 291.42 (19.52) |
|  | Hip Osteoarthritis | 29.31 (36.26) | 41.30 (42.90) |
|  | Knee Osteoarthritis | 199.94 (37.49) | 250.13 (43.91) |
| Western Europe | Osteoarthritis | 283.09 (21.11) | 347.35 (24.46) |
|  | Hip Osteoarthritis | 40.61 (34.03) | 54.64 (39.29) |
|  | Knee Osteoarthritis | 242.48 (34.24) | 292.71 (39.45) |
| Central Europe | Osteoarthritis | 222.65 (18.93) | 273.10 (20.64) |
|  | Hip Osteoarthritis | 30.99 (37.65) | 41.50 (42.21) |
|  | Knee Osteoarthritis | 191.66 (38.32) | 231.60 (42.86) |
| High-income North America | Osteoarthritis | 333.19 (21.83) | 410.23 (25.86) |
|  | Hip Osteoarthritis | 52.31 (37.79) | 69.33 (44.95) |
|  | Knee Osteoarthritis | 280.88 (37.98) | 340.90 (45.18) |
| Andean Latin America | Osteoarthritis | 217.34 (17.70) | 314.93 (22.97) |
|  | Hip Osteoarthritis | 15.81 (29.01) | 24.66 (37.73) |
|  | Knee Osteoarthritis | 201.53 (29.85) | 290.28 (38.60) |
| Central Latin America | Osteoarthritis | 246.49 (20.18) | 331.99 (23.94) |
|  | Hip Osteoarthritis | 17.35 (32.85) | 24.87 (41.23) |
|  | Knee Osteoarthritis | 229.15 (33.90) | 307.12 (41.92) |
| Caribbean | Osteoarthritis | 203.07 (16.68) | 291.37 (21.74) |
|  | Hip Osteoarthritis | 15.35 (26.83) | 23.87 (35.87) |
|  | Knee Osteoarthritis | 187.72 (27.55) | 267.50 (36.40) |
| Tropical Latin America | Osteoarthritis | 225.75 (18.55) | 317.33 (22.78) |
|  | Hip Osteoarthritis | 16.89 (30.71) | 26.60 (39.44) |
|  | Knee Osteoarthritis | 208.87 (31.76) | 290.74 (40.04) |
| Southern Latin America | Osteoarthritis | 296.96 (22.29) | 393.90 (26.80) |
|  | Hip Osteoarthritis | 34.13 (36.93) | 52.37 (43.54) |
|  | Knee Osteoarthritis | 262.82 (37.42) | 341.54 (43.91) |
| Eastern Sub-Saharan Africa | Osteoarthritis | 80.38 (8.84) | 132.30 (12.45) |
|  | Hip Osteoarthritis | 7.32 (13.55) | 12.80 (20.74) |
|  | Knee Osteoarthritis | 73.06 (14.10) | 119.51 (21.43) |
| Southern Sub-Saharan Africa | Osteoarthritis | 210.42 (17.29) | 298.00 (22.56) |
|  | Hip Osteoarthritis | 22.33 (30.45) | 33.10 (41.19) |
|  | Knee Osteoarthritis | 188.08 (32.01) | 264.90 (42.39) |
| Western Sub-Saharan Africa | Osteoarthritis | 129.33 (13.14) | 200.03 (18.28) |
|  | Hip Osteoarthritis | 10.90 (20.57) | 17.37 (29.67) |
|  | Knee Osteoarthritis | 118.43 (21.21) | 182.66 (30.17) |
| North Africa and Middle East | Osteoarthritis | 191.64 (20.26) | 289.12 (26.11) |
|  | Hip Osteoarthritis | 12.02 (32.06) | 20.72 (43.56) |
|  | Knee Osteoarthritis | 179.62 (33.14) | 268.40 (44.32) |
| Central Sub-Saharan Africa | Osteoarthritis | 96.86 (9.87) | 167.66 (15.81) |
|  | Hip Osteoarthritis | 8.56 (16.32) | 15.33 (27.14) |
|  | Knee Osteoarthritis | 88.30 (16.93) | 152.33 (27.74) |

**Abbreviations: DALY=disability-adjusted life-years.**

**Table S8. Age-standardised DALYs attributable to high BMI for osteoarthritis, hip osteoarthritis, and knee osteoarthritis among in 1990 and 2021, and their proportions, by country.**

|  | **Age-standardised DALYs attributable to high BMI for osteoarthritis (Proportion %)** | | **Age-standardised DALYs attributable to high BMI for Knee osteoarthritis (Proportion %)** | | **Age-standardised DALYs attributable to high BMI for Hip osteoarthritis (Proportion %)** | |
| --- | --- | --- | --- | --- | --- | --- |
| **Countries** | **1990** | **2021** | **1990** | **2021** | **1990** | **2021** |
| Afghanistan | 127.98(16.40) | 181.55(20.59) | 120.94(25.03) | 170.35(31.87) | 7.04(23.74) | 11.20(31.18) |
| Albania | 210.22(22.44) | 255.46(23.53) | 182.31(38.27) | 219.30(42.39) | 27.90(37.67) | 36.16(41.78) |
| Algeria | 173.85(18.39) | 271.22(24.02) | 163.56(30.42) | 252.03(41.59) | 10.29(29.16) | 19.18(40.72) |
| American Samoa | 279.19(23.26) | 337.33(26.12) | 252.68(40.91) | 304.72(46.10) | 26.51(40.65) | 32.62(45.77) |
| Andorra | 266.34(20.93) | 326.17(23.69) | 227.20(33.46) | 273.98(37.63) | 39.15(33.25) | 52.19(37.30) |
| Angola | 80.94(7.45) | 147.90(12.27) | 73.98(14.55) | 134.40(24.06) | 6.95(13.91) | 13.50(23.16) |
| Antigua and Barbuda | 223.04(17.02) | 307.01(22.59) | 206.44(30.18) | 283.36(39.36) | 16.60(29.15) | 23.65(38.40) |
| Argentina | 294.78(21.99) | 391.16(26.55) | 261.30(37.45) | 339.86(43.96) | 33.49(36.91) | 51.30(43.54) |
| Armenia | 185.21(16.89) | 223.43(17.07) | 158.13(37.69) | 188.28(41.57) | 27.08(35.96) | 35.15(40.01) |
| Australia | 290.33(21.24) | 410.31(27.15) | 254.68(35.02) | 350.97(43.62) | 35.65(34.74) | 59.33(43.47) |
| Austria | 285.69(21.57) | 338.19(24.21) | 245.25(34.61) | 285.59(38.48) | 40.44(34.50) | 52.60(38.25) |
| Azerbaijan | 178.85(14.18) | 224.98(16.97) | 152.42(35.20) | 189.78(41.43) | 26.43(34.28) | 35.20(40.58) |
| Bahamas | 264.50(19.51) | 345.46(24.54) | 244.11(33.80) | 318.09(42.26) | 20.40(32.85) | 27.36(41.45) |
| Bahrain | 221.42(20.39) | 316.57(27.65) | 207.00(36.16) | 293.44(47.67) | 14.42(35.16) | 23.13(47.03) |
| Bangladesh | 41.73(4.79) | 87.16(8.73) | 39.20(7.91) | 81.09(15.04) | 2.53(7.88) | 6.07(14.97) |
| Barbados | 263.26(19.43) | 348.66(24.61) | 242.69(33.29) | 320.21(42.38) | 20.57(32.35) | 28.45(41.52) |
| Belarus | 223.24(17.39) | 286.98(20.01) | 195.08(36.40) | 248.22(43.43) | 28.16(35.28) | 38.76(42.68) |
| Belgium | 253.07(19.27) | 325.34(23.51) | 217.55(31.36) | 275.13(37.82) | 35.52(31.03) | 50.21(37.55) |
| Belize | 266.47(22.06) | 353.20(26.15) | 245.71(35.63) | 323.50(43.06) | 20.75(34.27) | 29.71(42.19) |
| Benin | 124.02(13.23) | 192.92(17.23) | 114.05(20.70) | 175.90(28.48) | 9.97(19.12) | 17.02(27.21) |
| Bermuda | 306.72(21.62) | 390.43(26.35) | 281.36(36.88) | 356.42(45.14) | 25.36(36.21) | 34.01(44.56) |
| Bhutan | 125.79(14.28) | 184.63(17.81) | 117.86(22.50) | 170.53(28.97) | 7.93(22.15) | 14.09(28.38) |
| Bolivia (Plurinational State of) | 191.16(16.87) | 290.50(22.50) | 178.29(27.32) | 269.38(36.92) | 12.87(26.51) | 21.12(36.11) |
| Bosnia and Herzegovina | 189.37(18.47) | 243.52(20.06) | 165.14(34.48) | 208.97(40.37) | 24.23(33.17) | 34.55(39.42) |
| Botswana | 150.04(14.40) | 260.90(20.93) | 136.79(24.70) | 233.36(37.61) | 13.25(22.33) | 27.54(36.06) |
| Brazil | 225.41(18.53) | 317.42(22.75) | 208.54(31.72) | 290.77(40.03) | 16.87(30.67) | 26.64(39.43) |
| Brunei Darussalam | 165.14(10.54) | 307.38(18.44) | 153.05(16.81) | 283.33(29.13) | 12.10(16.51) | 24.05(28.24) |
| Bulgaria | 227.91(19.44) | 265.85(20.02) | 196.60(38.55) | 227.11(41.97) | 31.31(38.16) | 38.74(41.57) |
| Burkina Faso | 66.66(7.80) | 95.47(10.03) | 61.32(11.41) | 87.22(14.70) | 5.34(11.36) | 8.25(14.65) |
| Burundi | 64.03(7.17) | 87.69(9.63) | 58.47(11.27) | 79.96(15.23) | 5.56(10.67) | 7.72(14.41) |
| Cabo Verde | 129.36(13.19) | 225.00(19.25) | 118.40(20.62) | 203.97(32.51) | 10.96(19.35) | 21.02(31.31) |
| Cambodia | 46.48(6.50) | 75.50(8.90) | 42.98(10.46) | 69.93(15.48) | 3.50(9.64) | 5.57(14.38) |
| Cameroon | 196.12(20.01) | 280.44(25.14) | 178.85(30.08) | 254.27(39.68) | 17.27(28.67) | 26.17(38.91) |
| Canada | 181.94(16.90) | 236.88(20.22) | 151.24(37.32) | 191.19(41.98) | 30.71(37.05) | 45.70(41.78) |
| Central African Republic | 73.23(8.09) | 126.43(13.30) | 67.17(13.33) | 115.84(21.94) | 6.06(12.53) | 10.60(20.77) |
| Chad | 97.24(11.24) | 129.89(14.28) | 89.86(17.09) | 119.53(21.70) | 7.37(15.93) | 10.36(20.67) |
| Chile | 309.22(23.76) | 405.51(27.77) | 272.38(38.04) | 350.15(44.51) | 36.84(37.66) | 55.35(44.21) |
| China | 112.72(11.39) | 219.25(18.67) | 107.58(16.44) | 206.88(29.37) | 5.15(16.02) | 12.36(28.38) |
| Colombia | 214.32(18.31) | 310.25(23.22) | 200.34(30.45) | 287.96(39.81) | 13.98(29.23) | 22.29(38.99) |
| Comoros | 102.69(11.37) | 180.88(17.43) | 93.38(17.59) | 163.30(28.82) | 9.31(16.87) | 17.58(28.14) |
| Congo | 116.88(10.91) | 193.93(16.37) | 106.60(19.50) | 175.43(29.97) | 10.29(18.32) | 18.50(28.09) |
| Cook Islands | 260.96(22.73) | 340.06(25.67) | 236.32(40.21) | 306.68(45.64) | 24.64(39.27) | 33.39(44.78) |
| Costa Rica | 241.07(20.12) | 321.12(24.00) | 224.35(32.98) | 297.32(40.46) | 16.73(32.16) | 23.80(39.88) |
| Côte d'Ivoire | 121.65(13.22) | 191.97(18.08) | 111.51(20.34) | 175.05(29.06) | 10.13(19.79) | 16.91(28.48) |
| Croatia | 216.02(18.28) | 275.29(21.19) | 186.85(37.52) | 234.01(43.58) | 29.17(36.79) | 41.28(43.00) |
| Cuba | 192.18(15.92) | 292.18(21.87) | 178.05(26.20) | 268.72(36.46) | 14.13(24.71) | 23.46(35.23) |
| Cyprus | 225.68(18.76) | 321.59(23.96) | 195.20(29.65) | 273.10(38.27) | 30.48(29.38) | 48.49(38.03) |
| Czechia | 240.10(19.29) | 280.17(20.93) | 206.62(41.18) | 237.78(44.11) | 33.49(40.76) | 42.39(43.56) |
| Democratic People's Republic of Korea | 103.04(10.52) | 199.26(18.27) | 98.08(15.34) | 190.00(27.96) | 4.95(15.07) | 9.27(26.94) |
| Democratic Republic of the Congo | 97.20(10.17) | 169.79(16.90) | 88.56(16.95) | 154.43(28.52) | 8.64(16.39) | 15.36(28.08) |
| Denmark | 256.02(18.04) | 311.14(22.61) | 207.48(31.09) | 258.10(36.04) | 48.54(30.95) | 53.04(35.90) |
| Djibouti | 64.25(7.07) | 102.10(9.37) | 58.66(11.35) | 92.13(16.20) | 5.60(10.87) | 9.97(15.65) |
| Dominica | 291.97(23.93) | 365.77(27.63) | 270.55(38.66) | 336.94(45.86) | 21.42(37.90) | 28.83(45.36) |
| Dominican Republic | 173.38(14.33) | 272.12(20.14) | 160.60(24.31) | 250.47(34.32) | 12.78(23.88) | 21.65(33.46) |
| Ecuador | 236.46(18.76) | 356.24(25.54) | 218.34(30.92) | 327.37(42.14) | 18.12(30.00) | 28.87(41.36) |
| Egypt | 225.27(24.01) | 325.32(30.51) | 211.06(38.23) | 302.21(49.49) | 14.21(37.15) | 23.11(48.79) |
| El Salvador | 249.33(21.28) | 337.91(25.27) | 232.47(35.01) | 313.08(42.61) | 16.86(34.07) | 24.83(41.95) |
| Equatorial Guinea | 114.60(13.04) | 242.24(19.84) | 104.83(20.49) | 217.00(35.51) | 9.77(19.47) | 25.25(34.90) |
| Eritrea | 50.01(5.93) | 85.64(8.97) | 46.08(9.21) | 78.37(14.75) | 3.92(8.63) | 7.27(14.19) |
| Estonia | 241.27(17.95) | 294.58(19.46) | 210.49(38.54) | 252.29(42.68) | 30.78(37.76) | 42.29(41.92) |
| Eswatini | 238.22(22.29) | 332.92(27.22) | 214.49(35.68) | 297.79(46.65) | 23.74(33.53) | 35.12(45.45) |
| Ethiopia | 65.00(7.15) | 93.62(7.94) | 59.04(11.67) | 84.08(15.34) | 5.96(11.28) | 9.53(15.06) |
| Fiji | 229.15(21.97) | 321.13(26.32) | 209.99(37.63) | 294.06(46.32) | 19.16(36.26) | 27.07(45.40) |
| Finland | 288.83(21.98) | 351.25(25.23) | 247.10(34.80) | 295.63(39.93) | 41.73(34.57) | 55.63(39.78) |
| France | 241.92(18.50) | 325.85(23.46) | 205.71(29.97) | 272.28(37.55) | 36.22(29.86) | 53.56(37.47) |
| Gabon | 170.56(16.54) | 271.79(22.62) | 154.60(27.80) | 243.06(39.26) | 15.96(26.13) | 28.73(38.03) |
| Gambia | 135.15(14.22) | 196.05(17.38) | 124.43(22.45) | 179.09(29.61) | 10.72(21.05) | 16.96(28.31) |
| Georgia | 188.18(15.76) | 210.47(16.44) | 159.32(35.47) | 176.18(38.41) | 28.86(34.53) | 34.29(37.59) |
| Germany | 310.18(22.78) | 351.90(24.95) | 266.13(36.67) | 296.05(39.76) | 44.05(36.57) | 55.85(39.63) |
| Ghana | 105.24(9.37) | 209.75(17.72) | 96.75(17.47) | 191.19(30.32) | 8.49(16.89) | 18.56(29.65) |
| Greece | 269.37(21.99) | 353.31(25.85) | 233.53(33.52) | 302.05(40.53) | 35.84(33.28) | 51.26(40.31) |
| Greenland | 206.08(19.37) | 246.02(21.30) | 172.64(38.87) | 200.97(41.53) | 33.44(38.57) | 45.06(41.32) |
| Grenada | 191.14(15.59) | 284.39(21.55) | 177.51(26.33) | 263.37(36.58) | 13.63(24.82) | 21.02(35.33) |
| Guam | 240.90(20.65) | 301.91(22.68) | 219.53(36.85) | 273.47(40.96) | 21.37(36.07) | 28.43(40.10) |
| Guatemala | 211.50(19.93) | 289.74(24.20) | 198.80(31.93) | 270.64(39.51) | 12.70(30.32) | 19.09(38.20) |
| Guinea-Bissau | 108.31(12.16) | 163.73(16.57) | 99.97(18.49) | 150.69(26.11) | 8.34(17.47) | 13.04(25.19) |
| Guinea | 107.00(12.12) | 152.26(15.72) | 98.58(18.14) | 139.44(24.13) | 8.43(16.92) | 12.82(23.05) |
| Guyana | 202.29(17.58) | 279.96(21.77) | 187.89(28.84) | 259.17(36.70) | 14.40(27.69) | 20.79(35.68) |
| Haiti | 86.50(8.86) | 141.73(13.23) | 81.07(13.38) | 132.59(20.95) | 5.42(12.68) | 9.14(20.13) |
| Honduras | 218.57(19.78) | 291.71(23.97) | 204.82(31.89) | 272.41(38.96) | 13.75(30.64) | 19.30(38.06) |
| Hungary | 238.29(19.30) | 285.09(20.96) | 204.74(39.89) | 242.14(44.21) | 33.55(39.32) | 42.95(43.59) |
| Iceland | 309.93(21.85) | 368.35(25.64) | 254.89(36.19) | 304.71(40.74) | 55.04(36.09) | 63.64(40.53) |
| India | 54.10(5.79) | 118.77(10.59) | 50.82(9.59) | 110.36(18.71) | 3.28(9.61) | 8.41(18.75) |
| Indonesia | 47.41(5.67) | 97.21(9.74) | 43.41(10.30) | 89.00(19.11) | 4.00(9.65) | 8.21(17.46) |
| Iran (Islamic Republic of) | 158.42(16.44) | 267.47(23.55) | 148.15(28.54) | 247.08(42.57) | 10.26(26.93) | 20.39(41.41) |
| Iraq | 239.96(23.40) | 284.72(26.30) | 223.10(38.94) | 264.64(44.27) | 16.86(38.44) | 20.08(44.04) |
| Ireland | 266.53(20.72) | 345.48(24.96) | 228.31(32.65) | 290.78(39.36) | 38.22(32.42) | 54.70(39.30) |
| Israel | 287.13(22.34) | 347.76(25.12) | 248.11(36.09) | 296.60(40.25) | 39.02(35.65) | 51.16(39.81) |
| Italy | 256.89(19.17) | 326.34(22.58) | 221.51(32.09) | 277.79(38.11) | 35.39(31.86) | 48.55(37.96) |
| Jamaica | 226.14(18.46) | 315.53(23.69) | 209.59(30.62) | 289.87(39.84) | 16.54(28.83) | 25.66(38.30) |
| Japan | 209.23(12.92) | 252.06(14.29) | 194.36(20.66) | 231.88(24.28) | 14.87(19.90) | 20.17(23.87) |
| Jordan | 244.89(23.73) | 338.50(28.91) | 229.03(41.11) | 313.42(50.08) | 15.86(39.91) | 25.07(49.28) |
| Kazakhstan | 187.90(14.76) | 229.05(15.62) | 159.43(35.65) | 193.29(41.13) | 28.47(34.33) | 35.76(40.07) |
| Kenya | 91.25(8.91) | 164.01(13.58) | 82.20(15.36) | 147.23(25.45) | 9.05(14.86) | 16.78(24.72) |
| Kiribati | 197.89(18.41) | 279.63(23.66) | 182.77(32.55) | 258.66(41.53) | 15.12(31.24) | 20.96(40.31) |
| Kuwait | 248.38(22.46) | 357.17(29.84) | 230.28(39.45) | 327.28(51.24) | 18.11(38.60) | 29.89(50.70) |
| Kyrgyzstan | 177.87(14.81) | 214.15(16.43) | 152.39(35.92) | 182.47(41.88) | 25.48(34.40) | 31.69(40.56) |
| Lao People's Democratic Republic | 53.17(7.26) | 90.29(10.37) | 48.99(11.96) | 82.88(18.32) | 4.18(11.16) | 7.41(17.22) |
| Latvia | 252.28(19.28) | 302.67(20.37) | 219.74(40.09) | 259.59(44.07) | 32.53(39.08) | 43.08(43.31) |
| Lebanon | 214.57(21.78) | 304.25(25.77) | 200.64(37.36) | 280.45(46.12) | 13.93(36.53) | 23.79(45.57) |
| Lesotho | 181.37(18.35) | 252.60(21.89) | 165.42(29.97) | 228.71(38.48) | 15.95(27.41) | 23.88(36.41) |
| Liberia | 157.85(16.88) | 235.86(21.97) | 144.70(25.96) | 214.69(35.28) | 13.16(24.63) | 21.17(34.00) |
| Libya | 211.95(20.10) | 317.88(27.57) | 197.28(34.52) | 294.13(47.98) | 14.67(33.26) | 23.74(47.24) |
| Lithuania | 235.72(18.38) | 298.71(20.33) | 204.94(37.82) | 256.51(44.00) | 30.78(36.91) | 42.20(43.27) |
| Luxembourg | 284.16(21.15) | 346.84(24.84) | 243.64(34.02) | 292.10(39.79) | 40.53(33.86) | 54.74(39.66) |
| Madagascar | 73.88(8.74) | 124.90(13.98) | 67.34(13.22) | 113.95(21.42) | 6.54(12.50) | 10.95(20.35) |
| Malawi | 81.46(9.28) | 136.72(13.77) | 73.92(14.08) | 123.36(21.72) | 7.55(14.33) | 13.36(22.16) |
| Malaysia | 101.23(11.01) | 171.23(16.15) | 92.33(21.02) | 155.59(31.87) | 8.91(19.28) | 15.64(29.56) |
| Maldives | 60.27(7.03) | 124.52(11.63) | 55.23(14.09) | 113.36(24.06) | 5.04(12.75) | 11.15(21.91) |
| Mali | 99.33(11.47) | 129.75(13.32) | 91.90(17.38) | 119.25(20.96) | 7.42(16.21) | 10.51(19.87) |
| Malta | 243.80(18.82) | 338.68(24.15) | 209.59(30.64) | 285.60(38.66) | 34.21(30.19) | 53.08(38.24) |
| Marshall Islands | 215.36(20.28) | 273.73(23.67) | 199.01(37.49) | 252.23(44.28) | 16.35(36.21) | 21.50(43.41) |
| Mauritania | 191.57(19.43) | 268.13(23.66) | 174.94(29.80) | 242.20(37.86) | 16.62(29.21) | 25.93(37.73) |
| Mauritius | 121.24(12.76) | 186.48(17.09) | 110.89(24.83) | 169.57(34.36) | 10.34(23.43) | 16.91(32.57) |
| Mexico | 258.45(20.36) | 344.60(23.59) | 239.23(34.64) | 317.56(42.61) | 19.21(33.54) | 27.04(41.95) |
| Micronesia (Federated States of) | 224.81(21.23) | 301.30(25.16) | 206.00(36.40) | 276.65(44.57) | 18.81(35.41) | 24.65(43.75) |
| Monaco | 321.63(23.10) | 378.59(26.10) | 272.86(36.86) | 316.84(41.10) | 48.77(36.75) | 61.75(40.97) |
| Mongolia | 155.27(14.22) | 184.69(13.33) | 133.17(32.23) | 157.65(36.12) | 22.10(31.27) | 27.04(35.16) |
| Montenegro | 241.53(19.56) | 286.38(22.21) | 206.22(39.92) | 245.56(45.98) | 35.31(39.53) | 40.82(45.63) |
| Morocco | 162.46(16.59) | 241.66(22.78) | 152.59(28.86) | 224.99(38.42) | 9.87(27.37) | 16.67(37.33) |
| Mozambique | 79.04(9.27) | 130.56(13.65) | 72.19(14.14) | 118.78(21.62) | 6.85(13.43) | 11.78(20.63) |
| Myanmar | 55.39(7.36) | 90.08(9.70) | 51.13(12.52) | 83.13(18.16) | 4.26(11.45) | 6.95(16.56) |
| Namibia | 143.09(14.62) | 242.48(21.29) | 129.67(23.65) | 217.97(36.79) | 13.41(23.13) | 24.52(36.54) |
| Nauru | 236.91(22.26) | 320.23(25.95) | 216.33(39.28) | 294.21(46.14) | 20.58(37.47) | 26.02(44.94) |
| Nepal | 47.63(5.96) | 93.76(9.92) | 44.82(9.14) | 87.16(15.62) | 2.82(8.90) | 6.61(15.06) |
| Netherlands | 284.18(20.47) | 337.53(23.88) | 241.72(32.97) | 286.08(37.48) | 42.47(32.80) | 51.45(37.33) |
| New Zealand | 293.98(21.03) | 383.22(24.97) | 257.31(35.80) | 328.35(41.90) | 36.67(35.69) | 54.87(41.84) |
| Nicaragua | 251.76(23.00) | 331.25(26.51) | 235.63(36.15) | 308.53(43.21) | 16.12(34.77) | 22.72(42.09) |
| Niger | 93.52(10.89) | 120.96(13.14) | 86.52(16.35) | 111.62(19.82) | 7.00(15.19) | 9.34(18.59) |
| Nigeria | 140.56(13.65) | 223.20(19.46) | 128.40(22.66) | 203.93(33.38) | 12.16(22.03) | 19.27(32.97) |
| Niue | 224.16(18.91) | 317.30(24.18) | 204.30(33.70) | 288.49(42.99) | 19.86(33.04) | 28.82(42.34) |
| North Macedonia | 213.32(19.52) | 258.01(21.45) | 183.63(37.59) | 222.76(42.78) | 29.69(36.54) | 35.26(42.06) |
| Northern Mariana Islands | 251.97(21.44) | 323.18(25.67) | 228.08(38.43) | 293.88(45.65) | 23.89(38.08) | 29.30(45.18) |
| Norway | 249.03(18.25) | 299.99(20.78) | 213.14(31.53) | 252.97(35.33) | 35.88(31.30) | 47.03(35.05) |
| Oman | 156.38(15.80) | 309.46(26.16) | 147.03(28.44) | 286.97(45.93) | 9.36(27.47) | 22.49(45.30) |
| Pakistan | 78.91(9.06) | 148.48(14.36) | 72.62(16.61) | 135.29(27.27) | 6.29(16.60) | 13.19(27.19) |
| Palau | 255.26(22.32) | 326.95(26.00) | 232.23(39.45) | 297.07(46.16) | 23.03(38.87) | 29.88(45.40) |
| Palestine | 226.83(23.44) | 297.28(27.06) | 212.47(38.74) | 277.19(47.46) | 14.36(37.69) | 20.10(46.86) |
| Panama | 267.76(23.54) | 359.59(27.42) | 250.36(39.45) | 333.47(46.64) | 17.40(38.70) | 26.12(46.11) |
| Papua New Guinea | 114.22(13.57) | 143.88(15.29) | 105.94(21.09) | 133.24(24.93) | 8.28(19.60) | 10.64(23.39) |
| Paraguay | 238.61(19.28) | 313.54(24.27) | 221.15(33.17) | 289.17(40.59) | 17.46(32.19) | 24.37(40.02) |
| Peru | 215.65(17.41) | 300.77(21.78) | 200.11(29.99) | 277.28(37.20) | 15.54(29.10) | 23.49(36.17) |
| Philippines | 66.64(7.72) | 118.21(11.67) | 60.14(15.88) | 107.23(24.50) | 6.50(15.02) | 10.99(23.14) |
| Poland | 223.52(18.40) | 274.38(19.64) | 191.31(38.34) | 230.81(42.45) | 32.21(37.42) | 43.58(41.72) |
| Portugal | 256.36(20.54) | 342.58(24.68) | 221.69(32.29) | 291.41(39.00) | 34.67(31.84) | 51.17(38.67) |
| Puerto Rico | 306.33(22.16) | 388.35(25.78) | 280.74(37.57) | 352.43(43.78) | 25.59(36.77) | 35.91(43.07) |
| Qatar | 246.11(22.63) | 348.50(29.58) | 228.28(39.16) | 320.65(50.09) | 17.83(38.49) | 27.85(49.60) |
| Republic of Korea | 204.04(12.20) | 306.13(17.33) | 190.37(17.93) | 283.50(26.59) | 13.66(17.70) | 22.62(25.79) |
| Republic of Moldova | 228.04(20.33) | 299.15(22.85) | 200.79(38.79) | 258.23(45.26) | 27.25(37.82) | 40.92(44.40) |
| Romania | 207.57(18.86) | 265.32(21.39) | 179.68(36.28) | 225.64(41.66) | 27.88(35.86) | 39.68(41.20) |
| Russian Federation | 226.61(15.41) | 293.95(19.35) | 197.79(37.25) | 251.95(44.09) | 28.81(36.00) | 42.00(43.07) |
| Rwanda | 79.23(9.31) | 127.40(13.46) | 72.37(13.85) | 115.93(20.65) | 6.85(13.18) | 11.46(19.61) |
| Saint Kitts and Nevis | 236.19(18.35) | 331.43(24.16) | 218.53(31.15) | 305.41(41.07) | 17.66(30.24) | 26.02(40.34) |
| Saint Lucia | 197.95(16.82) | 282.60(21.11) | 183.78(27.23) | 260.24(35.54) | 14.16(26.16) | 22.36(34.39) |
| Saint Vincent and the Grenadines | 178.19(15.01) | 261.02(20.07) | 165.79(24.79) | 240.69(33.49) | 12.40(23.66) | 20.33(32.35) |
| Samoa | 253.08(23.49) | 312.63(25.60) | 230.84(39.38) | 285.23(45.27) | 22.24(38.18) | 27.39(44.12) |
| San Marino | 297.61(22.23) | 356.41(25.26) | 253.15(35.55) | 298.75(40.19) | 44.46(35.42) | 57.66(40.07) |
| Sao Tome and Principe | 160.01(15.63) | 236.71(19.31) | 146.80(25.57) | 215.47(34.52) | 13.21(24.08) | 21.24(33.24) |
| Saudi Arabia | 211.50(21.25) | 334.33(29.16) | 197.34(36.30) | 308.60(49.71) | 14.17(35.56) | 25.73(49.17) |
| Senegal | 129.22(13.58) | 183.21(17.20) | 118.35(21.15) | 167.25(27.96) | 10.87(20.33) | 15.96(27.11) |
| Serbia | 207.27(19.18) | 277.20(22.27) | 179.43(36.37) | 236.32(44.07) | 27.83(35.26) | 40.88(42.99) |
| Seychelles | 141.00(14.47) | 212.47(19.24) | 128.54(27.18) | 192.72(38.73) | 12.45(24.80) | 19.75(36.66) |
| Sierra Leone | 113.15(12.61) | 164.56(16.03) | 104.12(19.20) | 150.75(25.86) | 9.03(17.99) | 13.81(24.72) |
| Singapore | 175.06(10.49) | 332.51(19.14) | 162.31(16.86) | 305.49(30.36) | 12.75(17.10) | 27.02(30.14) |
| Slovakia | 240.66(19.64) | 281.62(21.04) | 207.10(41.20) | 240.41(44.66) | 33.56(40.35) | 41.21(43.93) |
| Slovenia | 233.04(18.76) | 280.09(20.97) | 200.22(39.65) | 236.07(43.85) | 32.82(39.14) | 44.01(43.41) |
| Solomon Islands | 141.70(15.41) | 201.00(18.52) | 130.31(25.72) | 185.02(32.14) | 11.39(24.61) | 15.98(31.36) |
| Somalia | 71.88(8.22) | 105.38(11.70) | 65.64(12.73) | 96.55(18.21) | 6.24(12.13) | 8.83(17.58) |
| South Africa | 232.19(18.00) | 312.77(22.70) | 206.99(34.65) | 277.28(43.74) | 25.20(32.65) | 35.49(42.34) |
| South Sudan | 59.48(7.08) | 83.23(9.24) | 53.96(10.56) | 75.25(13.91) | 5.51(10.16) | 7.98(13.43) |
| Spain | 304.65(23.51) | 381.68(26.96) | 259.94(36.80) | 319.88(42.76) | 44.71(36.35) | 61.80(42.44) |
| Sri Lanka | 76.86(9.38) | 132.73(13.74) | 70.48(16.96) | 121.76(26.38) | 6.38(15.60) | 10.97(24.76) |
| Sudan | 152.76(18.75) | 249.31(24.91) | 144.16(29.09) | 232.23(40.62) | 8.60(27.82) | 17.08(39.69) |
| Suriname | 179.55(14.04) | 263.80(19.07) | 165.92(24.66) | 243.09(33.01) | 13.63(23.77) | 20.71(31.96) |
| Sweden | 215.55(18.56) | 271.74(21.59) | 187.00(33.04) | 223.59(37.20) | 28.56(32.61) | 48.15(36.92) |
| Switzerland | 259.75(19.76) | 301.03(22.13) | 222.73(32.13) | 255.39(35.66) | 37.02(32.02) | 45.64(35.48) |
| Syrian Arab Republic | 208.87(22.28) | 314.18(29.01) | 195.26(36.12) | 292.62(49.34) | 13.61(34.93) | 21.56(48.79) |
| Taiwan (Province of China) | 148.08(13.84) | 253.00(19.72) | 139.43(22.83) | 238.35(33.31) | 8.65(21.95) | 14.65(32.10) |
| Tajikistan | 162.29(15.41) | 184.53(16.83) | 139.28(34.58) | 158.25(39.10) | 23.01(33.06) | 26.28(37.93) |
| Thailand | 75.93(9.28) | 164.88(16.09) | 69.96(15.58) | 150.99(28.47) | 5.96(14.51) | 13.89(26.24) |
| Timor-Leste | 30.07(4.17) | 47.34(5.42) | 27.56(6.96) | 43.56(10.25) | 2.51(6.74) | 3.79(9.98) |
| Togo | 122.15(13.25) | 195.42(18.40) | 112.54(20.59) | 179.52(30.11) | 9.61(19.42) | 15.90(29.24) |
| Tokelau | 203.65(19.38) | 293.76(23.48) | 186.21(33.33) | 267.05(41.98) | 17.43(32.94) | 26.71(41.72) |
| Tonga | 257.86(24.39) | 335.92(27.80) | 235.37(40.76) | 306.07(47.43) | 22.49(39.38) | 29.85(46.28) |
| Trinidad and Tobago | 246.48(19.23) | 327.81(23.83) | 227.80(32.62) | 301.49(40.48) | 18.68(31.54) | 26.32(39.40) |
| Tunisia | 166.83(17.31) | 265.16(23.55) | 156.59(29.06) | 246.43(41.39) | 10.24(28.00) | 18.73(40.73) |
| Turkey | 230.79(23.68) | 323.20(27.87) | 216.40(37.00) | 300.32(46.27) | 14.39(35.72) | 22.88(45.32) |
| Turkmenistan | 177.20(15.47) | 210.65(15.70) | 151.42(35.38) | 177.31(38.75) | 25.78(34.31) | 33.34(37.72) |
| Tuvalu | 206.11(20.25) | 287.96(23.77) | 190.26(34.29) | 264.24(42.50) | 15.86(33.80) | 23.73(41.96) |
| Uganda | 79.16(9.15) | 129.21(13.23) | 72.24(13.99) | 117.32(20.97) | 6.92(13.18) | 11.89(19.90) |
| Ukraine | 234.71(17.80) | 282.76(19.66) | 204.20(37.97) | 243.28(43.38) | 30.51(36.73) | 39.48(42.27) |
| United Arab Emirates | 199.64(19.09) | 312.92(29.53) | 186.39(33.30) | 289.30(48.70) | 13.25(32.62) | 23.61(48.42) |
| United Kingdom | 326.15(22.77) | 396.69(26.10) | 279.27(36.85) | 334.47(42.08) | 46.88(36.77) | 62.22(41.99) |
| United Republic of Tanzania | 110.82(11.65) | 189.83(18.42) | 100.76(18.91) | 171.18(29.72) | 10.06(18.07) | 18.64(28.45) |
| United States of America | 348.03(22.16) | 431.60(26.35) | 293.60(38.01) | 359.35(45.41) | 54.43(37.83) | 72.24(45.22) |
| United States Virgin Islands | 321.93(23.89) | 381.28(26.13) | 295.78(39.90) | 348.27(43.79) | 26.15(39.09) | 33.01(43.21) |
| Uruguay | 286.13(21.34) | 370.17(25.01) | 253.06(35.83) | 320.38(40.85) | 33.07(35.41) | 49.79(40.61) |
| Uzbekistan | 179.93(15.20) | 215.89(16.73) | 153.24(36.03) | 183.03(41.20) | 26.69(34.62) | 32.86(40.16) |
| Vanuatu | 152.20(17.40) | 214.52(21.56) | 140.15(26.64) | 198.06(33.72) | 12.05(25.05) | 16.46(31.96) |
| Venezuela (Bolivarian Republic of) | 264.97(21.49) | 340.78(25.56) | 247.05(36.20) | 316.50(43.53) | 17.92(35.30) | 24.28(42.91) |
| Viet Nam | 33.11(4.40) | 67.15(7.54) | 30.52(7.31) | 61.97(13.69) | 2.59(6.97) | 5.18(12.86) |
| Yemen | 101.49(12.90) | 183.49(19.65) | 96.12(19.86) | 172.39(32.68) | 5.37(19.07) | 11.11(32.32) |
| Zambia | 95.27(9.91) | 176.26(17.10) | 86.48(16.28) | 159.72(28.04) | 8.78(15.41) | 16.54(26.56) |
| Zimbabwe | 121.04(12.61) | 212.52(21.48) | 109.61(19.74) | 192.84(33.80) | 11.43(18.69) | 19.68(32.25) |

**Abbreviations: DALY=disability-adjusted life-years.**

**Table S9. Projected age-standardised incidence and DALY rates for osteoarthritis and its four anatomical sites by sex from 2022 to 2040 based on the BAPC model.**

|  |  | **Age-standardised incidence rate** | | | **Age-standardised DALY rate** | | |
| --- | --- | --- | --- | --- | --- | --- | --- |
| **Year** | **Cause** | **Both** | **Male** | **Female** | **Both** | **Male** | **Female** |
| 2022 | OA | 1438.50 (1425.90 to 1451.10) | 1247.42 (1235.12 to 1259.72) | 1602.27 (1587.77 to 1616.77) | 1295.40 (1285.00 to 1305.80) | 1063.97 (1054.44 to 1073.50) | 1485.16 (1472.76 to 1497.56) |
| 2023 | OA | 1444.01 (1425.21 to 1462.81) | 1253.26 (1235.26 to 1271.26) | 1606.84 (1585.34 to 1628.34) | 1298.90 (1283.10 to 1314.70) | 1069.62 (1055.02 to 1084.22) | 1486.70 (1467.50 to 1505.90) |
| 2024 | OA | 1449.26 (1422.56 to 1475.96) | 1259.06 (1233.56 to 1284.56) | 1610.97 (1580.47 to 1641.47) | 1302.38 (1279.68 to 1325.08) | 1075.14 (1054.14 to 1096.14) | 1488.21 (1460.41 to 1516.01) |
| 2025 | OA | 1454.29 (1418.19 to 1490.39) | 1264.84 (1230.64 to 1299.04) | 1614.68 (1573.58 to 1655.78) | 1305.79 (1274.89 to 1336.69) | 1080.55 (1052.05 to 1109.05) | 1489.62 (1451.72 to 1527.52) |
| 2026 | OA | 1459.30 (1412.50 to 1506.10) | 1270.73 (1226.53 to 1314.93) | 1618.22 (1565.02 to 1671.42) | 1309.16 (1269.16 to 1349.16) | 1085.93 (1049.03 to 1122.83) | 1490.91 (1441.71 to 1540.11) |
| 2027 | OA | 1464.41 (1405.71 to 1523.11) | 1276.76 (1221.46 to 1332.06) | 1621.80 (1555.30 to 1688.30) | 1312.60 (1262.30 to 1362.90) | 1091.42 (1045.02 to 1137.82) | 1492.17 (1430.47 to 1553.87) |
| 2028 | OA | 1469.50 (1397.80 to 1541.20) | 1282.86 (1215.36 to 1350.36) | 1625.26 (1544.16 to 1706.36) | 1316.09 (1254.69 to 1377.49) | 1096.91 (1040.21 to 1153.62) | 1493.44 (1418.14 to 1568.74) |
| 2029 | OA | 1474.43 (1388.73 to 1560.13) | 1288.94 (1208.34 to 1369.54) | 1628.45 (1531.65 to 1725.25) | 1319.56 (1246.26 to 1392.86) | 1102.32 (1034.52 to 1170.12) | 1494.69 (1404.89 to 1584.49) |
| 2030 | OA | 1479.21 (1378.47 to 1579.95) | 1295.02 (1200.32 to 1389.72) | 1631.36 (1517.89 to 1744.82) | 1322.97 (1236.87 to 1409.07) | 1107.64 (1027.94 to 1187.34) | 1495.86 (1390.56 to 1601.16) |
| 2031 | OA | 1483.97 (1367.26 to 1600.67) | 1301.17 (1191.44 to 1410.89) | 1634.15 (1502.94 to 1765.37) | 1326.33 (1226.63 to 1426.03) | 1112.92 (1020.52 to 1205.32) | 1496.93 (1375.23 to 1618.62) |
| 2032 | OA | 1488.78 (1355.11 to 1622.45) | 1307.40 (1181.67 to 1433.12) | 1636.97 (1486.96 to 1786.98) | 1329.70 (1215.57 to 1443.84) | 1118.27 (1012.39 to 1224.14) | 1497.94 (1358.92 to 1636.96) |
| 2033 | OA | 1493.58 (1342.02 to 1645.14) | 1313.68 (1171.03 to 1456.33) | 1639.71 (1469.92 to 1809.50) | 1333.10 (1203.75 to 1462.44) | 1123.61 (1003.47 to 1243.75) | 1498.93 (1341.71 to 1656.15) |
| 2034 | OA | 1498.28 (1327.92 to 1668.65) | 1319.96 (1159.48 to 1480.44) | 1642.29 (1451.78 to 1832.81) | 1336.47 (1191.15 to 1481.79) | 1128.90 (993.74 to 1264.05) | 1499.91 (1323.65 to 1676.16) |
| 2035 | OA | 1502.89 (1312.83 to 1692.94) | 1326.23 (1147.03 to 1505.43) | 1644.71 (1432.56 to 1856.86) | 1339.81 (1177.76 to 1501.85) | 1134.13 (983.21 to 1285.05) | 1500.84 (1304.73 to 1696.94) |
| 2036 | OA | 1507.47 (1296.84 to 1718.11) | 1332.54 (1133.72 to 1531.37) | 1647.05 (1412.35 to 1881.76) | 1343.10 (1163.58 to 1522.61) | 1139.33 (971.90 to 1306.76) | 1501.70 (1284.93 to 1718.47) |
| 2037 | OA | 1512.09 (1279.97 to 1744.21) | 1338.91 (1119.55 to 1558.26) | 1649.41 (1391.23 to 1907.59) | 1346.38 (1148.63 to 1544.13) | 1144.56 (959.85 to 1329.28) | 1502.52 (1264.27 to 1740.77) |
| 2038 | OA | 1516.70 (1262.24 to 1771.17) | 1345.31 (1104.54 to 1586.08) | 1651.73 (1369.20 to 1934.26) | 1349.67 (1132.94 to 1566.39) | 1149.79 (947.05 to 1352.54) | 1503.31 (1242.81 to 1763.82) |
| 2039 | OA | 1521.28 (1243.61 to 1798.94) | 1351.72 (1088.66 to 1614.79) | 1653.98 (1346.24 to 1961.72) | 1352.94 (1116.51 to 1589.37) | 1155.01 (933.50 to 1376.52) | 1504.09 (1220.56 to 1787.62) |
| 2040 | OA | 1525.81 (1224.12 to 1827.50) | 1358.15 (1071.92 to 1644.37) | 1656.16 (1322.39 to 1989.93) | 1356.20 (1099.35 to 1613.04) | 1160.20 (919.19 to 1401.21) | 1504.86 (1197.56 to 1812.16) |
| 2022 | hand | 280.07 (277.71 to 282.43) | 212.34 (209.99 to 214.69) | 335.20 (332.06 to 338.34) | 403.34 (399.25 to 407.43) | 292.63 (289.65 to 295.61) | 494.62 (489.22 to 500.02) |
| 2023 | hand | 281.91 (278.19 to 285.63) | 214.51 (210.90 to 218.12) | 336.82 (332.10 to 341.54) | 405.44 (398.62 to 412.26) | 295.58 (290.71 to 300.45) | 496.43 (486.66 to 506.20) |
| 2024 | hand | 283.73 (278.25 to 289.21) | 216.74 (211.49 to 221.99) | 338.37 (331.60 to 345.14) | 407.63 (397.33 to 417.93) | 298.54 (291.24 to 305.84) | 498.32 (483.05 to 513.59) |
| 2025 | hand | 285.55 (278.01 to 293.09) | 219.02 (211.83 to 226.21) | 339.86 (330.68 to 349.04) | 409.85 (395.48 to 424.23) | 301.51 (291.36 to 311.66) | 500.23 (478.57 to 521.88) |
| 2026 | hand | 287.41 (277.52 to 297.30) | 221.37 (211.97 to 230.77) | 341.35 (329.44 to 353.26) | 412.10 (393.13 to 431.07) | 304.51 (291.12 to 317.91) | 502.09 (473.26 to 530.93) |
| 2027 | hand | 289.31 (276.81 to 301.81) | 223.79 (211.91 to 235.67) | 342.85 (327.90 to 357.81) | 414.38 (390.30 to 438.46) | 307.60 (290.58 to 324.62) | 503.95 (467.19 to 540.72) |
| 2028 | hand | 291.24 (275.88 to 306.60) | 226.29 (211.67 to 240.90) | 344.33 (326.07 to 362.60) | 416.76 (387.10 to 446.42) | 310.73 (289.73 to 331.74) | 505.89 (460.50 to 551.29) |
| 2029 | hand | 293.17 (274.71 to 311.63) | 228.83 (211.23 to 246.43) | 345.76 (323.93 to 367.60) | 419.21 (383.52 to 454.90) | 313.88 (288.55 to 339.21) | 507.91 (453.22 to 562.60) |
| 2030 | hand | 295.09 (273.31 to 316.88) | 231.43 (210.61 to 252.26) | 347.15 (321.50 to 372.81) | 421.70 (379.54 to 463.85) | 317.03 (287.04 to 347.02) | 509.94 (445.33 to 574.55) |
| 2031 | hand | 297.04 (271.70 to 322.38) | 234.09 (209.79 to 258.38) | 348.53 (318.81 to 378.25) | 424.19 (375.14 to 473.24) | 320.21 (285.21 to 355.21) | 511.94 (436.80 to 587.08) |
| 2032 | hand | 299.03 (269.90 to 328.15) | 236.80 (208.78 to 264.82) | 349.91 (315.88 to 383.94) | 426.70 (370.32 to 483.08) | 323.44 (283.10 to 363.79) | 513.92 (427.63 to 600.20) |
| 2033 | hand | 301.02 (267.88 to 334.17) | 239.57 (207.58 to 271.57) | 351.28 (312.70 to 389.85) | 429.28 (365.14 to 493.41) | 326.72 (280.67 to 372.77) | 515.94 (417.92 to 613.96) |
| 2034 | hand | 303.02 (265.64 to 340.40) | 242.39 (206.17 to 278.61) | 352.61 (309.25 to 395.96) | 431.90 (359.58 to 504.22) | 330.01 (277.92 to 382.09) | 518.02 (407.67 to 628.36) |
| 2035 | hand | 305.02 (263.19 to 346.86) | 245.25 (204.55 to 285.95) | 353.91 (305.55 to 402.26) | 434.55 (353.64 to 515.46) | 333.30 (274.84 to 391.76) | 520.10 (396.86 to 643.35) |
| 2036 | hand | 307.04 (260.52 to 353.55) | 248.16 (202.72 to 293.60) | 355.20 (301.62 to 408.78) | 437.20 (347.27 to 527.12) | 336.61 (271.43 to 401.79) | 522.17 (385.48 to 658.86) |
| 2037 | hand | 309.07 (257.65 to 360.48) | 251.11 (200.67 to 301.56) | 356.49 (297.46 to 415.53) | 439.85 (340.49 to 539.20) | 339.96 (267.70 to 412.22) | 524.20 (373.51 to 674.90) |
| 2038 | hand | 311.11 (254.56 to 367.65) | 254.11 (198.40 to 309.83) | 357.77 (293.06 to 422.48) | 442.53 (333.32 to 551.73) | 343.34 (263.65 to 423.03) | 526.25 (361.00 to 691.51) |
| 2039 | hand | 313.16 (251.26 to 375.05) | 257.16 (195.90 to 318.42) | 359.03 (288.42 to 429.63) | 445.23 (325.76 to 564.71) | 346.74 (259.27 to 434.21) | 528.33 (347.96 to 708.70) |
| 2040 | hand | 315.21 (247.75 to 382.68) | 260.24 (193.16 to 327.32) | 360.28 (283.56 to 436.99) | 447.96 (317.80 to 578.13) | 350.16 (254.54 to 445.77) | 530.42 (334.40 to 726.45) |
| 2022 | hip | 70.55 (69.63 to 71.47) | 72.24 (71.44 to 73.05) | 69.44 (68.36 to 70.52) | 75.76 (75.09 to 76.44) | 76.15 (75.42 to 76.88) | 75.65 (74.92 to 76.37) |
| 2023 | hip | 70.34 (69.05 to 71.63) | 72.30 (71.14 to 73.46) | 69.01 (67.53 to 70.49) | 75.19 (74.13 to 76.25) | 75.96 (74.83 to 77.09) | 74.82 (73.69 to 75.95) |
| 2024 | hip | 70.08 (68.33 to 71.83) | 72.33 (70.72 to 73.94) | 68.53 (66.53 to 70.53) | 74.57 (73.03 to 76.11) | 75.72 (74.09 to 77.35) | 73.96 (72.34 to 75.58) |
| 2025 | hip | 69.78 (67.48 to 72.08) | 72.33 (70.20 to 74.46) | 68.01 (65.40 to 70.62) | 73.92 (71.83 to 76.01) | 75.45 (73.25 to 77.65) | 73.07 (70.90 to 75.24) |
| 2026 | hip | 69.47 (66.54 to 72.40) | 72.32 (69.59 to 75.05) | 67.47 (64.18 to 70.76) | 73.28 (70.59 to 75.97) | 75.19 (72.36 to 78.02) | 72.19 (69.40 to 74.98) |
| 2027 | hip | 69.16 (65.54 to 72.78) | 72.30 (68.90 to 75.70) | 66.93 (62.89 to 70.97) | 72.66 (69.31 to 76.01) | 74.93 (71.39 to 78.47) | 71.32 (67.87 to 74.77) |
| 2028 | hip | 68.83 (64.47 to 73.19) | 72.28 (68.15 to 76.41) | 66.38 (61.53 to 71.23) | 72.02 (67.96 to 76.08) | 74.66 (70.37 to 78.95) | 70.46 (66.31 to 74.61) |
| 2029 | hip | 68.47 (63.31 to 73.63) | 72.23 (67.32 to 77.14) | 65.79 (60.09 to 71.49) | 71.36 (66.56 to 76.16) | 74.36 (69.27 to 79.45) | 69.56 (64.67 to 74.45) |
| 2030 | hip | 68.10 (62.10 to 74.10) | 72.18 (66.43 to 77.93) | 65.20 (58.61 to 71.79) | 70.67 (65.10 to 76.24) | 74.03 (68.10 to 79.96) | 68.64 (62.99 to 74.29) |
| 2031 | hip | 67.73 (60.85 to 74.61) | 72.12 (65.49 to 78.75) | 64.60 (57.08 to 72.12) | 69.99 (63.62 to 76.36) | 73.70 (66.89 to 80.52) | 67.73 (61.29 to 74.17) |
| 2032 | hip | 67.36 (59.55 to 75.17) | 72.06 (64.49 to 79.63) | 64.00 (55.51 to 72.49) | 69.32 (62.11 to 76.53) | 73.38 (65.64 to 81.12) | 66.84 (59.59 to 74.09) |
| 2033 | hip | 66.97 (58.19 to 75.75) | 71.99 (63.43 to 80.55) | 63.40 (53.91 to 72.89) | 68.64 (60.56 to 76.72) | 73.05 (64.34 to 81.76) | 65.94 (57.85 to 74.03) |
| 2034 | hip | 66.57 (56.79 to 76.35) | 71.91 (62.32 to 81.50) | 62.78 (52.25 to 73.30) | 67.94 (58.98 to 76.90) | 72.69 (62.98 to 82.40) | 65.02 (56.08 to 73.96) |
| 2035 | hip | 66.17 (55.35 to 76.98) | 71.82 (61.16 to 82.49) | 62.15 (50.57 to 73.73) | 67.23 (57.36 to 77.10) | 72.32 (61.58 to 83.05) | 64.10 (54.30 to 73.90) |
| 2036 | hip | 65.75 (53.87 to 77.63) | 71.73 (59.94 to 83.52) | 61.52 (48.86 to 74.18) | 66.53 (55.73 to 77.32) | 71.94 (60.14 to 83.74) | 63.19 (52.51 to 73.86) |
| 2037 | hip | 65.34 (52.36 to 78.32) | 71.63 (58.68 to 84.59) | 60.90 (47.14 to 74.66) | 65.82 (54.08 to 77.57) | 71.56 (58.66 to 84.46) | 62.28 (50.71 to 73.85) |
| 2038 | hip | 64.93 (50.82 to 79.03) | 71.53 (57.37 to 85.69) | 60.27 (45.39 to 75.15) | 65.12 (52.41 to 77.83) | 71.18 (57.15 to 85.21) | 61.38 (48.91 to 73.85) |
| 2039 | hip | 64.50 (49.24 to 79.76) | 71.42 (56.01 to 86.83) | 59.63 (43.61 to 75.65) | 64.41 (50.73 to 78.10) | 70.78 (55.60 to 85.97) | 60.48 (47.11 to 73.85) |
| 2040 | hip | 64.07 (47.63 to 80.50) | 71.31 (54.61 to 88.00) | 59.00 (41.83 to 76.16) | 63.70 (49.03 to 78.38) | 70.38 (54.02 to 86.74) | 59.58 (45.30 to 73.86) |
| 2022 | knee | 961.23 (951.59 to 970.87) | 834.81 (825.86 to 843.76) | 1072.46 (1061.26 to 1083.66) | 682.40 (676.54 to 688.26) | 552.96 (547.57 to 558.35) | 788.45 (781.47 to 795.43) |
| 2023 | knee | 965.32 (951.42 to 979.22) | 838.18 (825.28 to 851.08) | 1076.38 (1060.28 to 1092.48) | 684.78 (676.02 to 693.54) | 555.37 (547.32 to 563.42) | 790.50 (780.20 to 800.80) |
| 2024 | knee | 969.25 (949.75 to 988.75) | 841.49 (823.49 to 859.49) | 1080.04 (1057.64 to 1102.44) | 687.07 (674.57 to 699.57) | 557.72 (546.32 to 569.12) | 792.42 (777.82 to 807.02) |
| 2025 | knee | 973.02 (947.02 to 999.02) | 844.76 (820.66 to 868.86) | 1083.40 (1053.40 to 1113.40) | 689.27 (672.47 to 706.07) | 559.99 (544.59 to 575.39) | 794.22 (774.52 to 813.92) |
| 2026 | knee | 976.78 (943.28 to 1010.28) | 848.09 (817.19 to 878.99) | 1086.64 (1048.14 to 1125.14) | 691.41 (669.61 to 713.21) | 562.23 (542.33 to 582.13) | 795.92 (770.52 to 821.32) |
| 2027 | knee | 980.60 (938.70 to 1022.50) | 851.48 (812.88 to 890.08) | 1089.91 (1041.81 to 1138.01) | 693.59 (666.29 to 720.89) | 564.47 (539.67 to 589.27) | 797.62 (765.92 to 829.32) |
| 2028 | knee | 984.42 (933.42 to 1035.42) | 854.89 (807.99 to 901.79) | 1093.14 (1034.64 to 1151.64) | 695.73 (662.53 to 728.93) | 566.69 (536.39 to 596.99) | 799.26 (760.66 to 837.86) |
| 2029 | knee | 988.14 (927.24 to 1049.04) | 858.28 (802.28 to 914.28) | 1096.20 (1026.40 to 1166.00) | 697.81 (658.11 to 737.51) | 568.87 (532.77 to 604.97) | 800.81 (754.81 to 846.81) |
| 2030 | knee | 991.75 (920.35 to 1063.15) | 861.63 (796.03 to 927.23) | 1099.07 (1017.27 to 1180.87) | 699.81 (653.21 to 746.41) | 571.00 (528.70 to 613.30) | 802.26 (748.36 to 856.16) |
| 2031 | knee | 995.34 (912.64 to 1078.04) | 865.02 (789.12 to 940.92) | 1101.84 (1007.34 to 1196.34) | 701.77 (647.87 to 755.67) | 573.10 (524.10 to 622.10) | 803.64 (741.34 to 865.94) |
| 2032 | knee | 998.97 (904.27 to 1093.67) | 868.44 (781.54 to 955.34) | 1104.65 (996.54 to 1212.75) | 703.74 (642.04 to 765.44) | 575.20 (519.20 to 631.20) | 805.00 (733.80 to 876.20) |
| 2033 | knee | 1002.59 (895.27 to 1109.92) | 871.88 (773.48 to 970.28) | 1107.42 (985.02 to 1229.82) | 705.69 (635.79 to 775.59) | 577.29 (513.79 to 640.79) | 806.33 (725.83 to 886.83) |
| 2034 | knee | 1006.17 (885.55 to 1126.79) | 875.30 (764.73 to 985.87) | 1110.11 (972.71 to 1247.50) | 707.59 (629.09 to 786.09) | 579.35 (508.05 to 650.65) | 807.60 (717.20 to 898.00) |
| 2035 | knee | 1009.68 (875.12 to 1144.23) | 878.70 (755.38 to 1002.03) | 1112.69 (959.60 to 1265.77) | 709.46 (621.96 to 796.96) | 581.39 (501.89 to 660.89) | 808.81 (708.19 to 909.43) |
| 2036 | knee | 1013.17 (864.05 to 1162.30) | 882.13 (745.46 to 1018.79) | 1115.22 (945.76 to 1284.68) | 711.30 (614.40 to 808.20) | 583.41 (495.41 to 671.41) | 809.99 (698.65 to 921.32) |
| 2037 | knee | 1016.69 (852.35 to 1181.03) | 885.57 (734.96 to 1036.18) | 1117.77 (931.25 to 1304.29) | 713.14 (606.39 to 819.88) | 585.43 (488.43 to 682.43) | 811.15 (688.66 to 933.65) |
| 2038 | knee | 1020.21 (840.03 to 1200.39) | 889.03 (723.90 to 1054.16) | 1120.31 (916.06 to 1324.55) | 714.97 (597.98 to 831.95) | 587.44 (481.17 to 693.72) | 812.30 (678.20 to 946.40) |
| 2039 | knee | 1023.72 (827.08 to 1220.35) | 892.49 (712.27 to 1072.71) | 1122.81 (900.20 to 1345.43) | 716.78 (589.17 to 844.39) | 589.45 (473.50 to 705.40) | 813.42 (667.31 to 959.54) |
| 2040 | knee | 1027.20 (813.52 to 1240.89) | 895.94 (700.07 to 1091.82) | 1125.29 (883.67 to 1366.90) | 718.58 (579.95 to 857.20) | 591.45 (465.47 to 717.44) | 814.53 (655.97 to 973.08) |
| 2022 | other | 126.50 (125.51 to 127.49) | 127.36 (126.26 to 128.46) | 125.73 (124.70 to 126.76) | 134.83 (133.84 to 135.82) | 141.31 (140.18 to 142.44) | 129.48 (128.49 to 130.47) |
| 2023 | other | 126.53 (125.04 to 128.02) | 127.38 (125.75 to 129.01) | 125.75 (124.21 to 127.29) | 134.95 (133.43 to 136.47) | 141.50 (139.78 to 143.22) | 129.54 (128.04 to 131.04) |
| 2024 | other | 126.56 (124.42 to 128.70) | 127.41 (125.10 to 129.72) | 125.78 (123.59 to 127.97) | 135.07 (132.88 to 137.26) | 141.69 (139.23 to 144.15) | 129.61 (127.45 to 131.77) |
| 2025 | other | 126.58 (123.69 to 129.47) | 127.43 (124.33 to 130.53) | 125.80 (122.84 to 128.76) | 135.19 (132.21 to 138.17) | 141.90 (138.58 to 145.22) | 129.67 (126.74 to 132.60) |
| 2026 | other | 126.61 (122.87 to 130.35) | 127.47 (123.47 to 131.47) | 125.82 (122.00 to 129.64) | 135.32 (131.46 to 139.18) | 142.11 (137.82 to 146.40) | 129.74 (125.95 to 133.53) |
| 2027 | other | 126.64 (121.95 to 131.33) | 127.50 (122.51 to 132.49) | 125.85 (121.08 to 130.62) | 135.45 (130.61 to 140.29) | 142.33 (136.96 to 147.70) | 129.81 (125.07 to 134.55) |
| 2028 | other | 126.68 (120.97 to 132.39) | 127.53 (121.46 to 133.60) | 125.87 (120.06 to 131.68) | 135.59 (129.69 to 141.49) | 142.56 (136.02 to 149.10) | 129.88 (124.10 to 135.66) |
| 2029 | other | 126.71 (119.90 to 133.52) | 127.57 (120.35 to 134.79) | 125.89 (118.97 to 132.81) | 135.73 (128.68 to 142.78) | 142.80 (135.00 to 150.60) | 129.96 (123.08 to 136.84) |
| 2030 | other | 126.74 (118.77 to 134.71) | 127.61 (119.16 to 136.06) | 125.92 (117.82 to 134.02) | 135.87 (127.61 to 144.13) | 143.04 (133.89 to 152.19) | 130.03 (121.97 to 138.09) |
| 2031 | other | 126.77 (117.56 to 135.98) | 127.65 (117.90 to 137.40) | 125.94 (116.58 to 135.30) | 136.02 (126.47 to 145.57) | 143.29 (132.72 to 153.86) | 130.10 (120.79 to 139.41) |
| 2032 | other | 126.81 (116.28 to 137.33) | 127.69 (116.56 to 138.82) | 125.97 (115.28 to 136.65) | 136.17 (125.25 to 147.09) | 143.54 (131.46 to 155.63) | 130.17 (119.54 to 140.81) |
| 2033 | other | 126.84 (114.94 to 138.74) | 127.73 (115.15 to 140.31) | 125.99 (113.91 to 138.07) | 136.32 (123.96 to 148.68) | 143.80 (130.13 to 157.48) | 130.25 (118.23 to 142.27) |
| 2034 | other | 126.87 (113.53 to 140.22) | 127.77 (113.68 to 141.87) | 126.02 (112.48 to 139.55) | 136.47 (122.61 to 150.34) | 144.07 (128.72 to 159.42) | 130.32 (116.85 to 143.80) |
| 2035 | other | 126.91 (112.06 to 141.75) | 127.81 (112.14 to 143.49) | 126.04 (110.98 to 141.10) | 136.63 (121.19 to 152.07) | 144.33 (127.24 to 161.43) | 130.40 (115.40 to 145.40) |
| 2036 | other | 126.94 (110.53 to 143.35) | 127.86 (110.53 to 145.18) | 126.07 (109.43 to 142.71) | 136.78 (119.70 to 153.87) | 144.60 (125.68 to 163.53) | 130.47 (113.89 to 147.05) |
| 2037 | other | 126.98 (108.94 to 145.01) | 127.90 (108.86 to 146.94) | 126.09 (107.80 to 144.38) | 136.94 (118.14 to 155.74) | 144.88 (124.05 to 165.70) | 130.55 (112.32 to 148.78) |
| 2038 | other | 127.01 (107.29 to 146.74) | 127.95 (107.13 to 148.76) | 126.11 (106.12 to 146.11) | 137.10 (116.52 to 157.68) | 145.15 (122.35 to 167.96) | 130.62 (110.68 to 150.56) |
| 2039 | other | 127.05 (105.57 to 148.52) | 127.99 (105.34 to 150.64) | 126.14 (104.38 to 147.89) | 137.26 (114.84 to 159.69) | 145.43 (120.57 to 170.30) | 130.70 (108.99 to 152.41) |
| 2040 | other | 127.08 (103.81 to 150.35) | 128.04 (103.49 to 152.58) | 126.16 (102.59 to 149.74) | 137.42 (113.09 to 161.75) | 145.71 (118.72 to 172.71) | 130.77 (107.23 to 154.31) |

**Abbreviations: DALY=disability-adjusted life-years.**

**
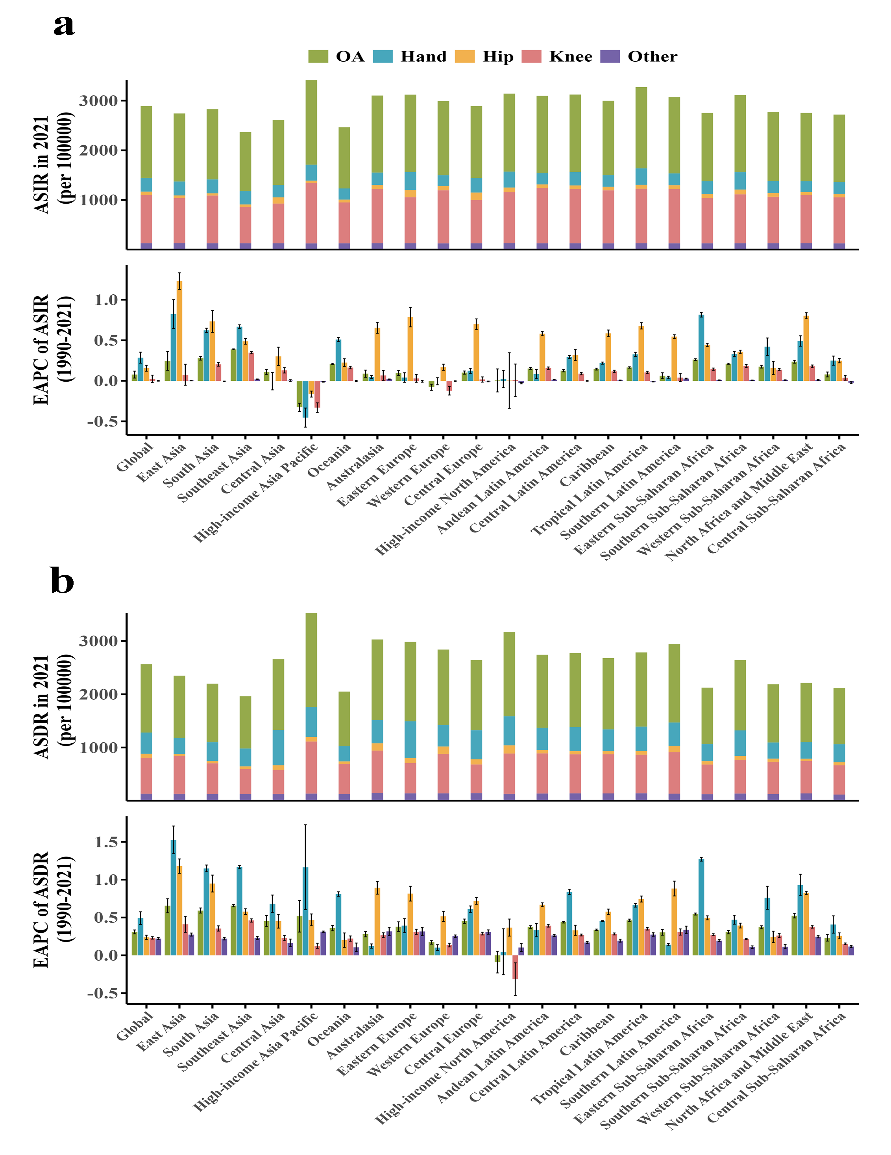
Figure S1. Age-standardised incidence and DALY rates (per 100,000 population) and estimated annual percentage change.**

**
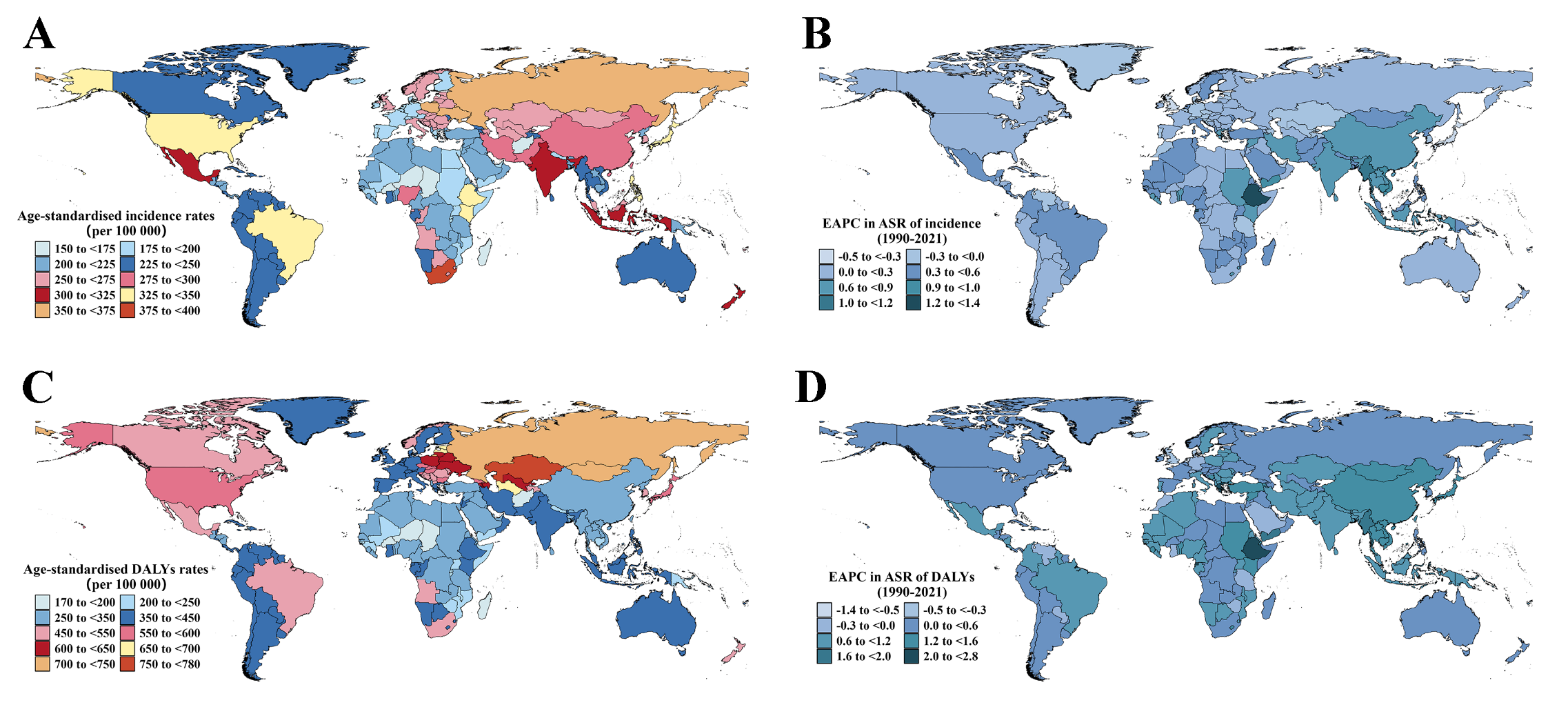
Figure S2.** **Age-standardised incidence and DALY rates and estimated annual percentage change in hand osteoarthritis**

**
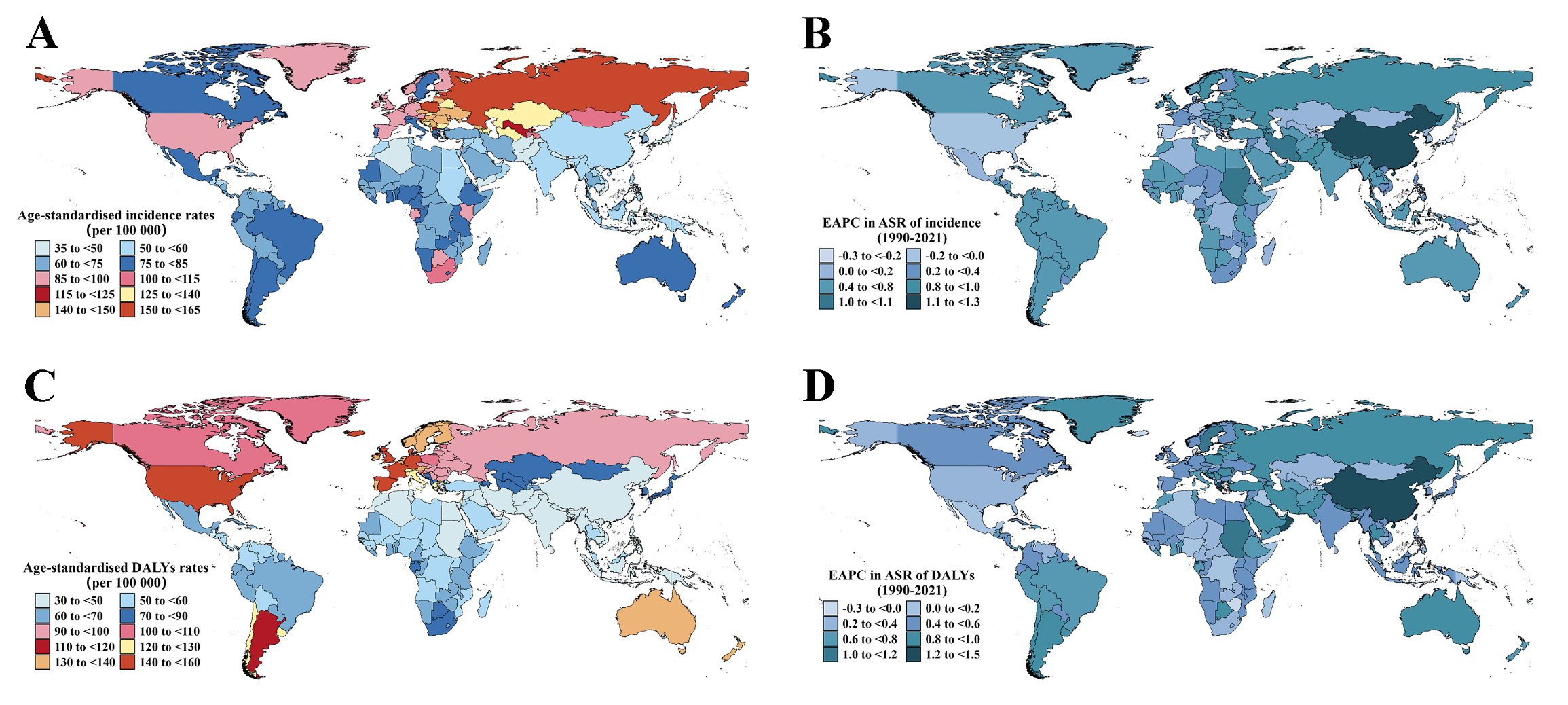
Figure S3. Age-standardised incidence and DALY rates and estimated annual percentage change in hip osteoarthritis**

**
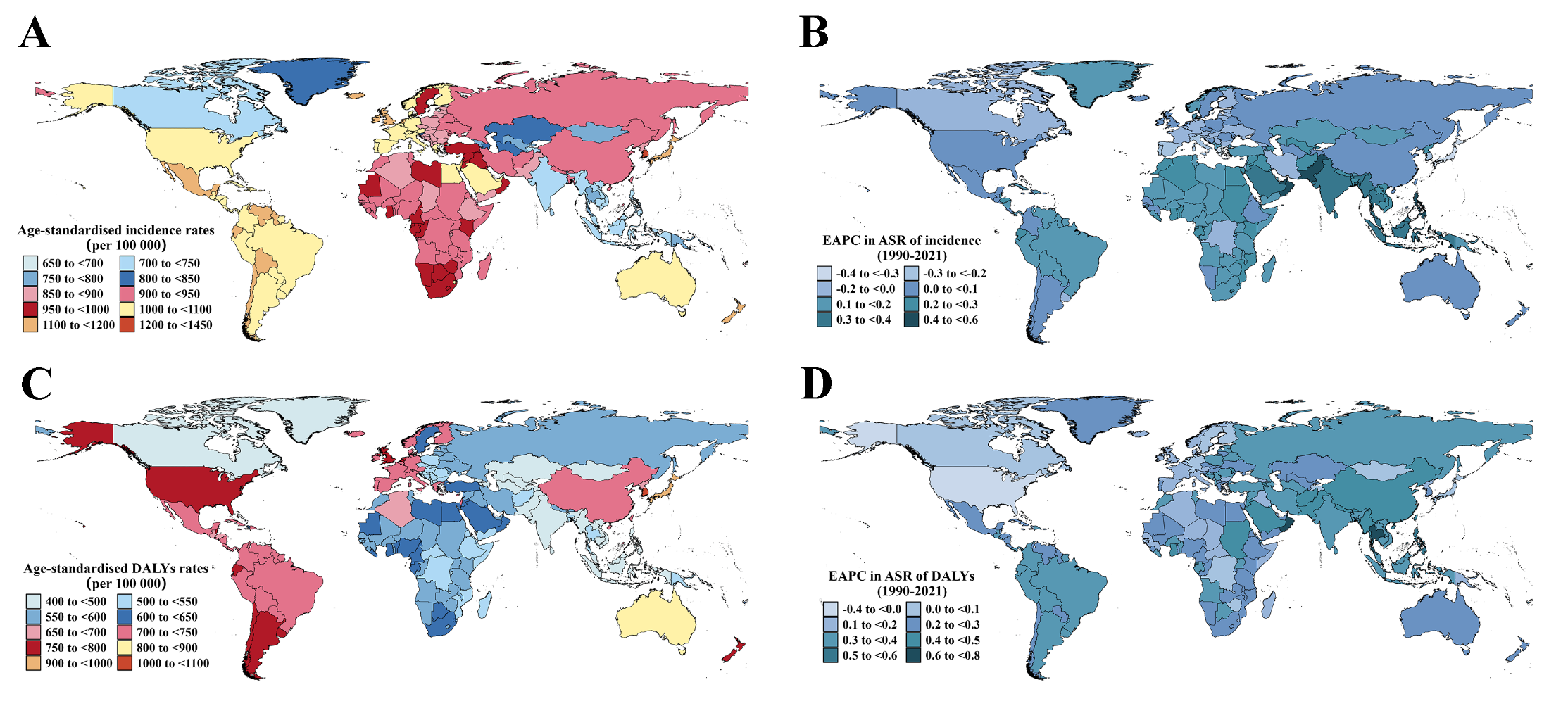
Figure S4. Age-standardised incidence and DALY rates and estimated annual percentage change in knee osteoarthritis**

**
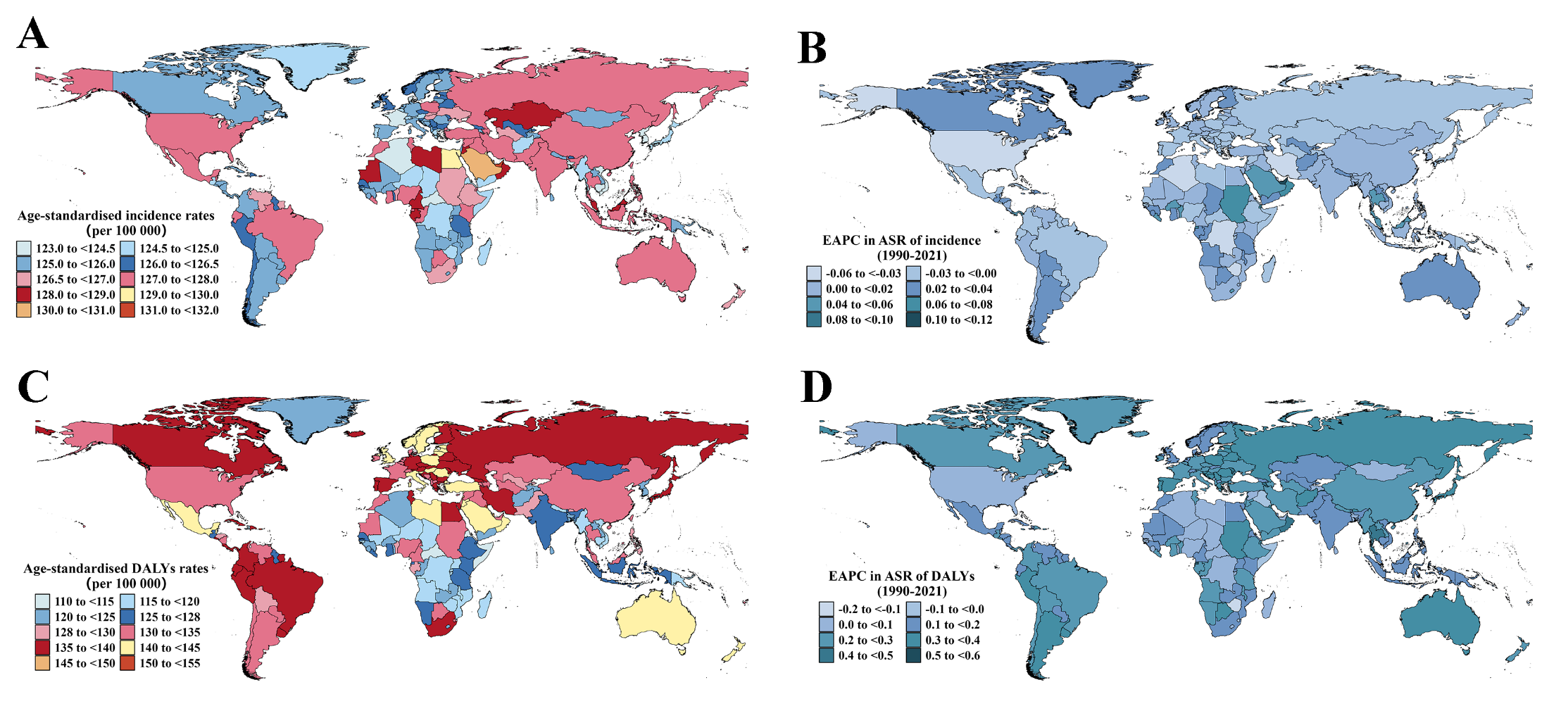
****Figure S5. Age-standardised incidence and DALY rates and estimated annual percentage change in other sites osteoarthritis**

**Figure S6. EAPCs in age-standardised incidence and DALY rates for osteoarthritis and its four anatomical sites across different age groups of older adults globally and in 21 GBD regions from 1990 to 2021.
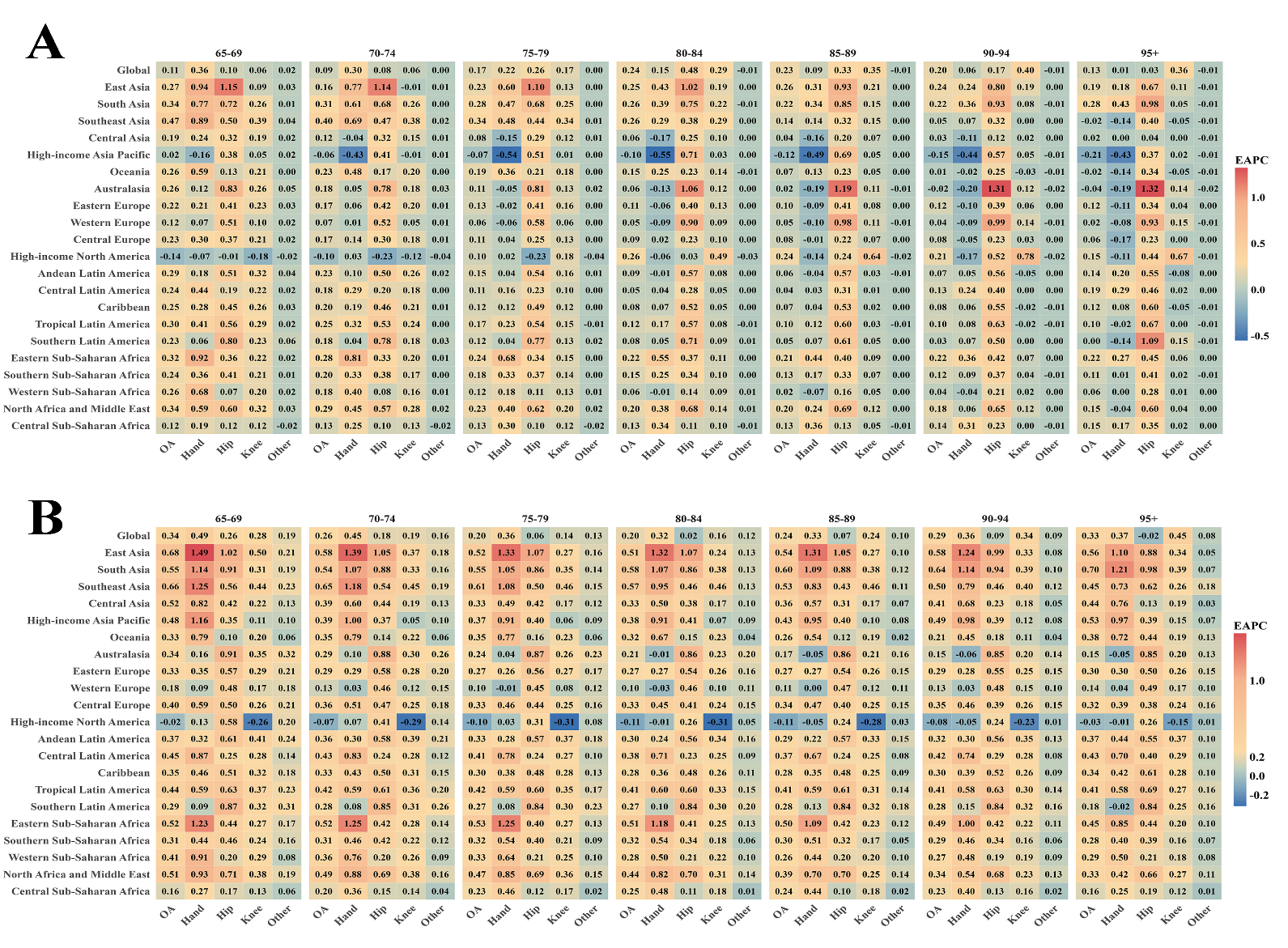
**

**Figure S7. Age-standardised incidence and DALY rates of OA and its four anatomical sites by SDI
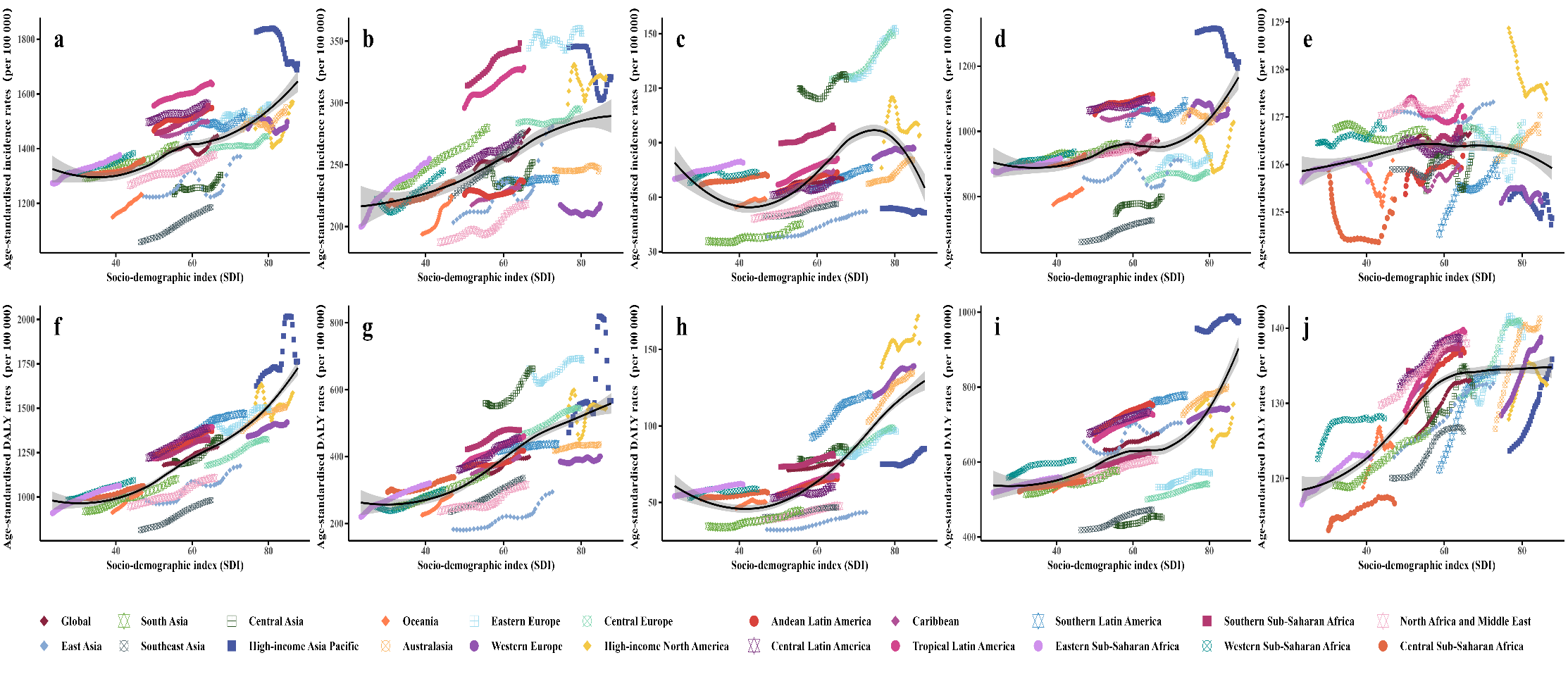
**

**
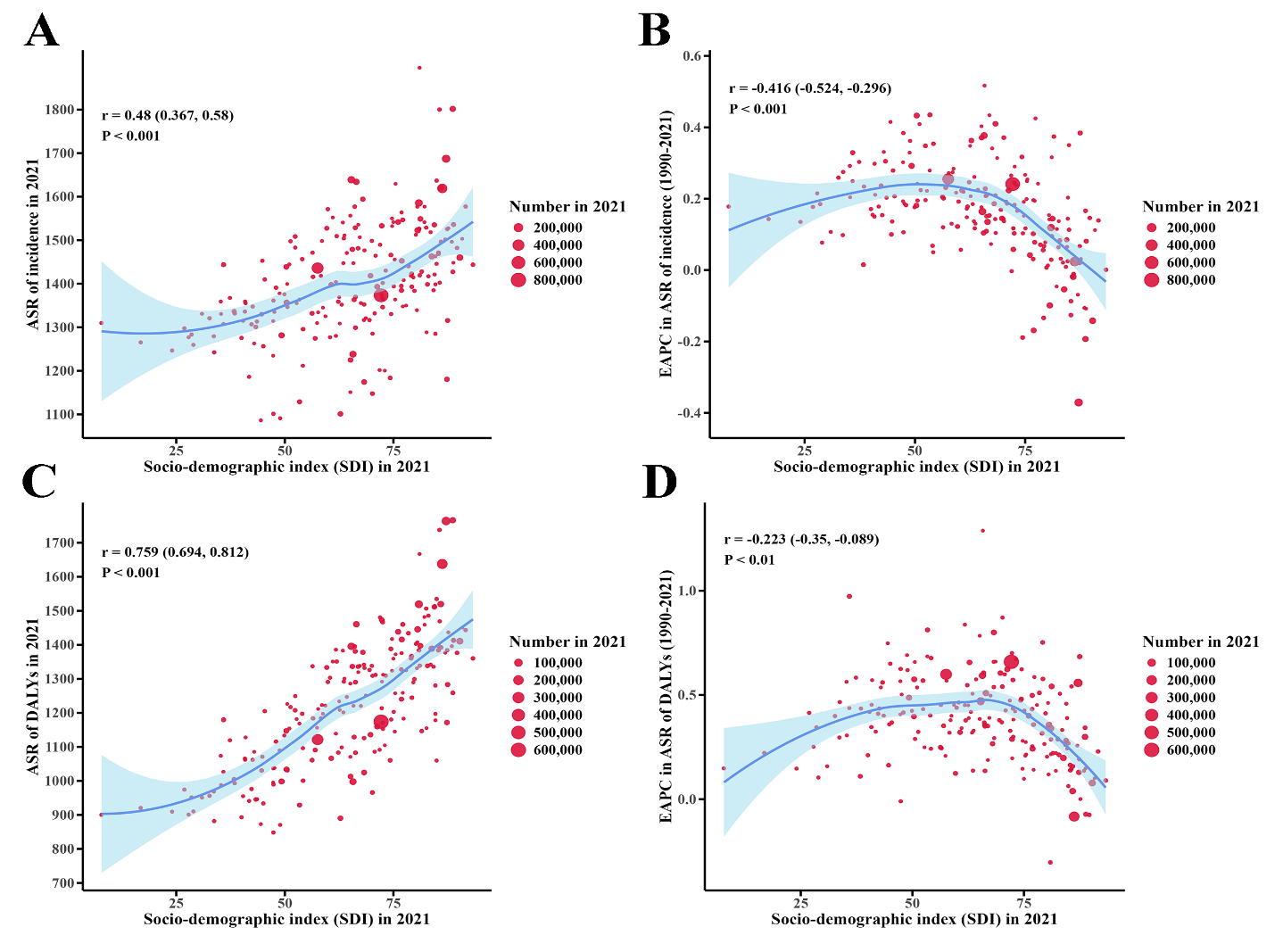
Figure S8. Age-standardised incidence and DALY rates for osteoarthritis among older adults in 204 countries and territories in 2021, and their estimated annual percentage changes (1990-2021), by SDI in 2021.**

**
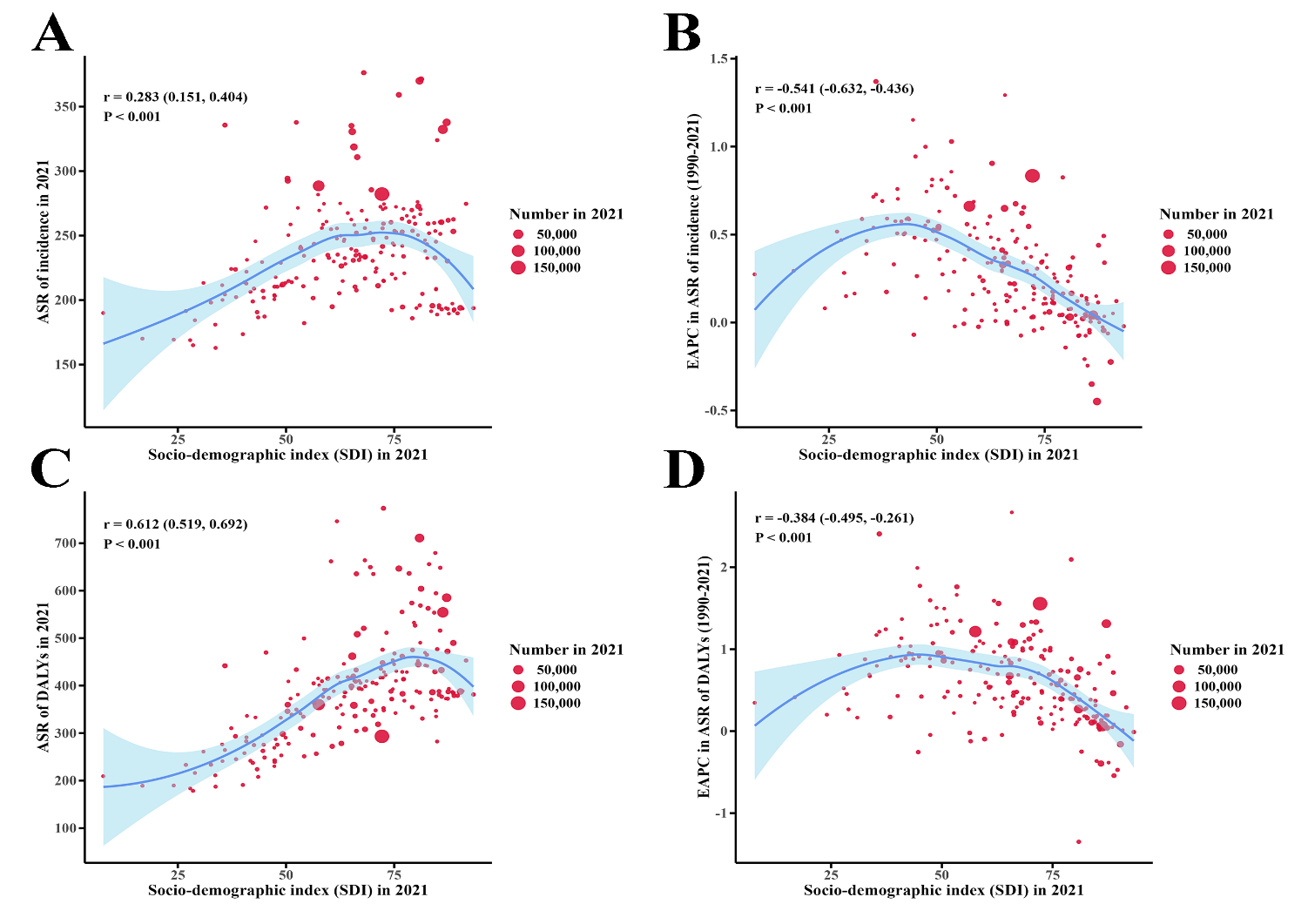
Figure S9. Age-standardised incidence and DALY rates for hand osteoarthritis among older adults in 204 countries and territories in 2021, and their estimated annual percentage changes (1990-2021), by SDI in 2021.**

**
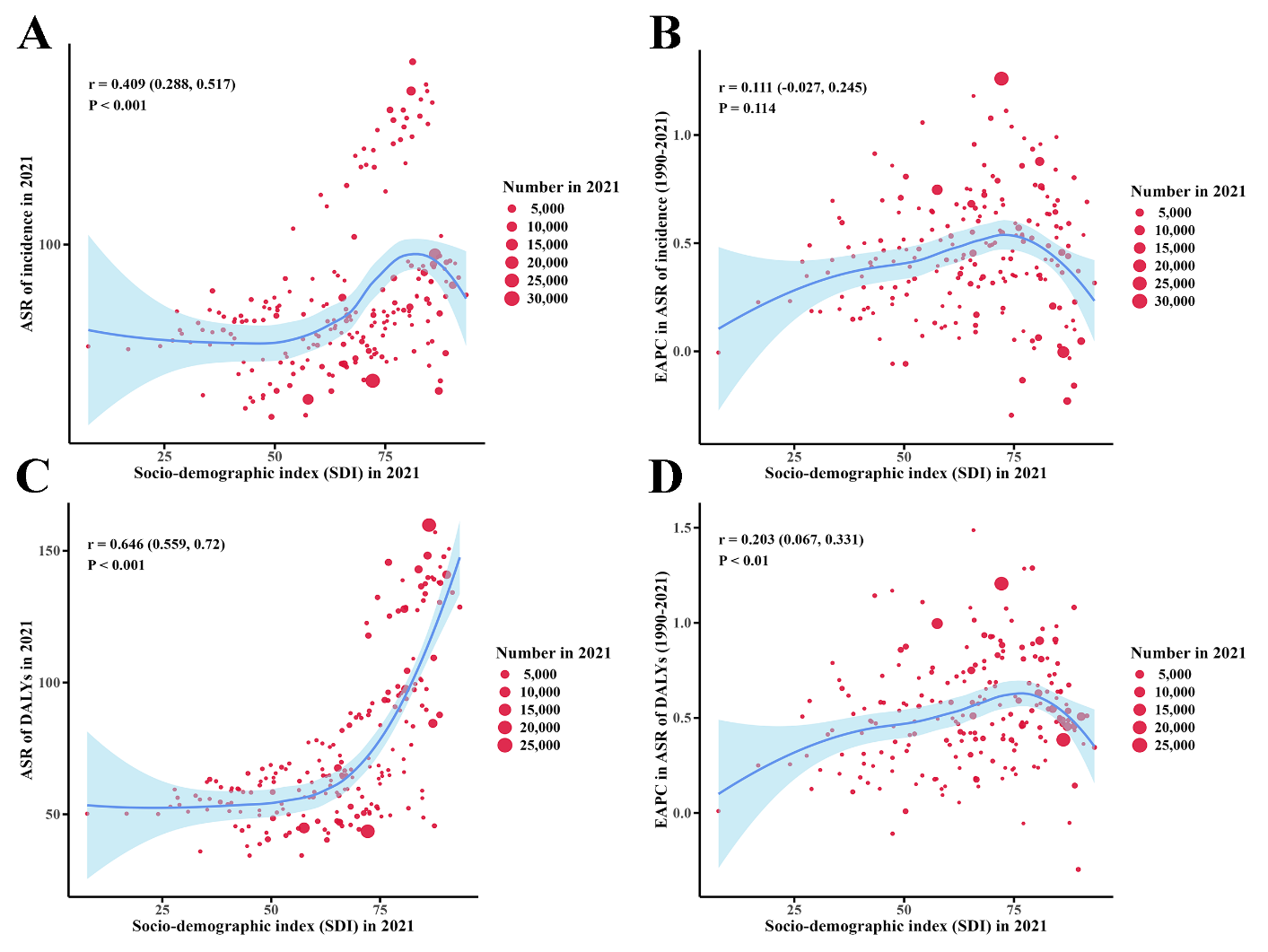
Figure S10. Age-standardised incidence and DALY rates for hip osteoarthritis among older adults in 204 countries and territories in 2021, and their estimated annual percentage changes (1990-2021), by SDI in 2021.**

**
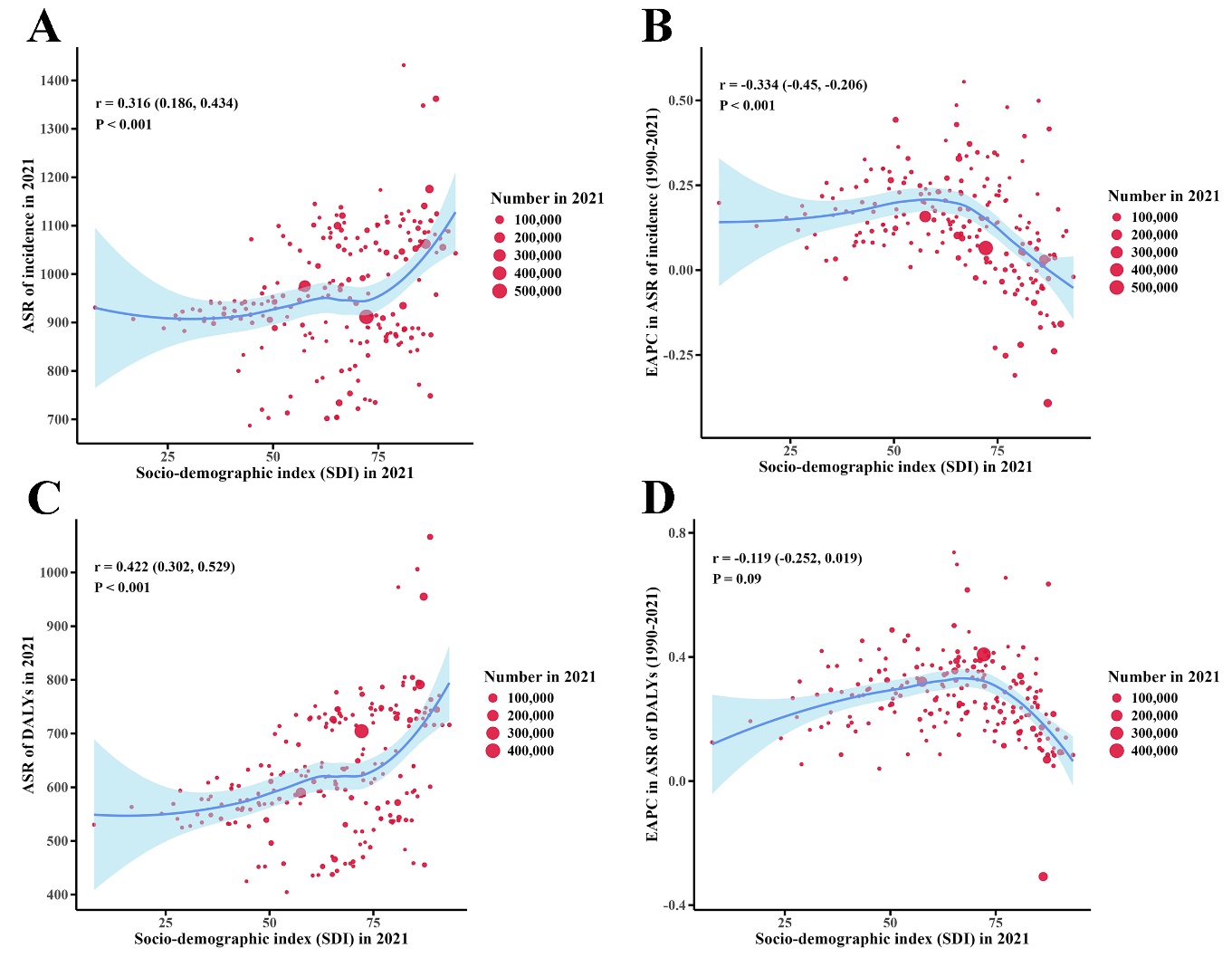
Figure S11. Age-standardised incidence and DALY rates for knee osteoarthritis among older adults in 204 countries and territories in 2021, and their estimated annual percentage changes (1990-2021), by SDI in 2021.**

**
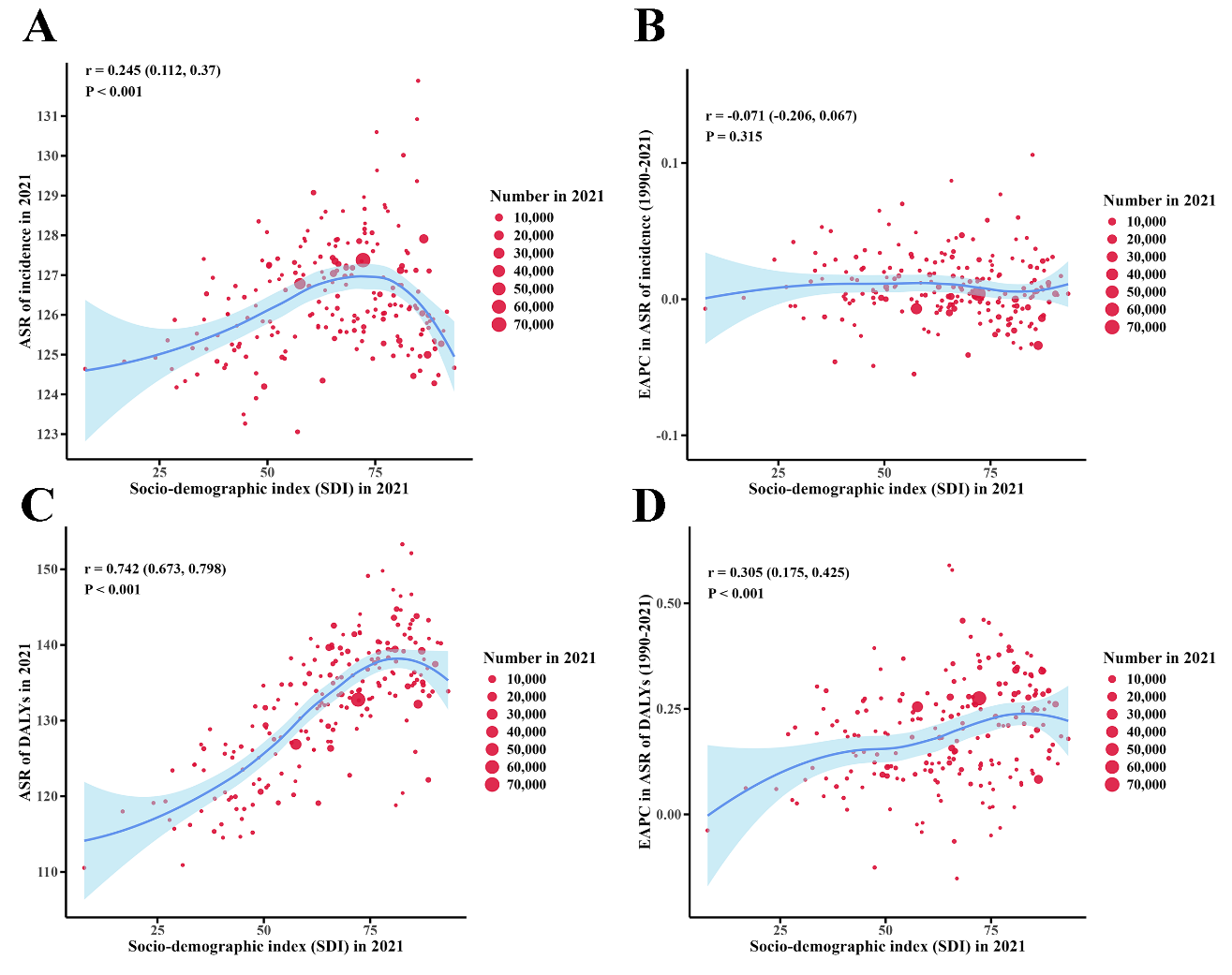
Figure S12. Age-standardised incidence and DALY rates for other sites osteoarthritis among older adults in 204 countries and territories in 2021, and their estimated annual percentage changes (1990-2021), by SDI in 2021.**

**
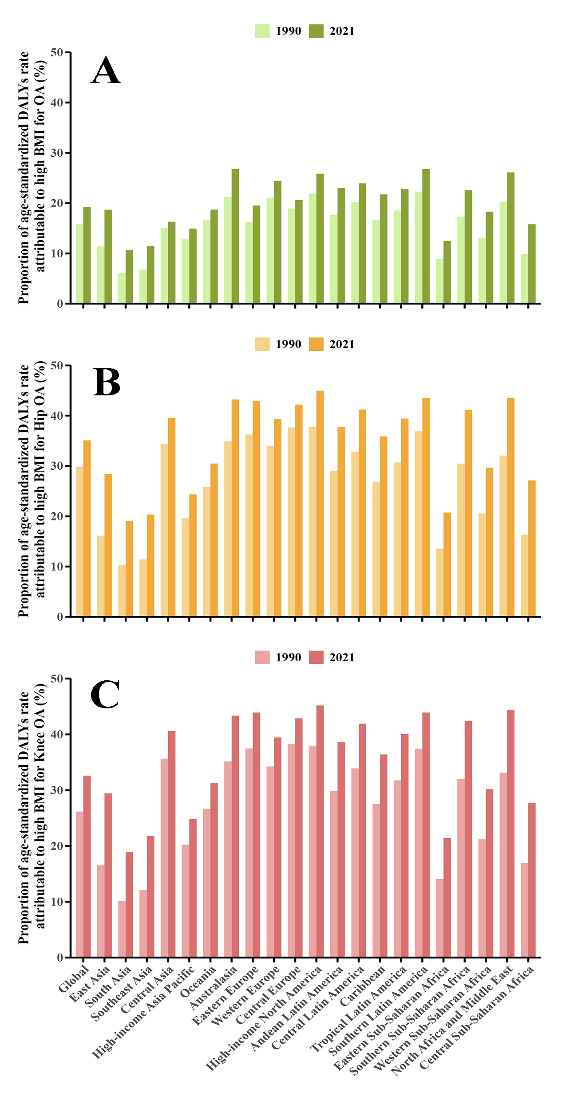
Figure S13. Proportion and trend of the osteoarthritis burden attributable to high BMI among older adults in 1990 and 2021, globally and across 21 GBD regions.**

**
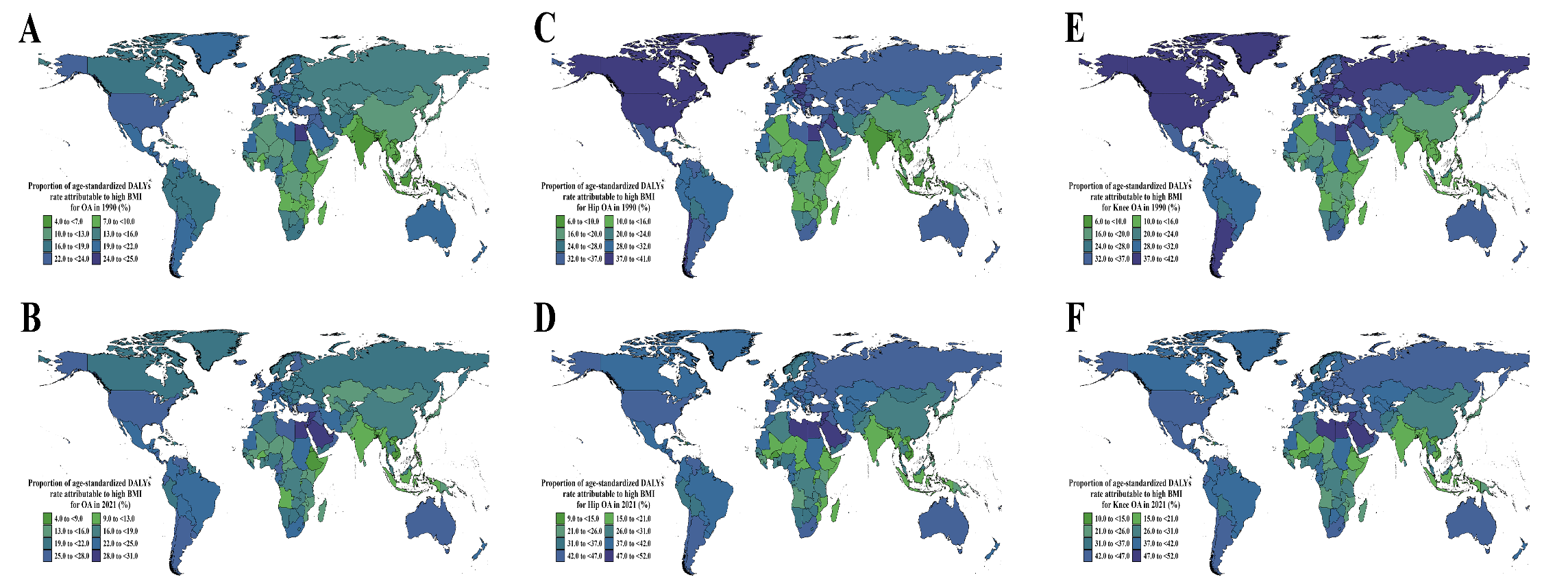
Figure S14.** **Proportion and trend of the osteoarthritis burden attributable to high BMI among older adults in 1990 and 2021, at the national level.**
